# Structure–activity relationship, *in vitro* and *in vivo* evaluation of novel dienyl sulphonyl fluorides as selective BuChE inhibitors for the treatment of Alzheimer's disease

**DOI:** 10.1080/14756366.2021.1959571

**Published:** 2021-08-23

**Authors:** Chengyao Wu, Guijuan Zhang, Zai-Wei Zhang, Xia Jiang, Ziwen Zhang, Huanhuan Li, Hua-Li Qin, Wenjian Tang

**Affiliations:** a School of Pharmacy, Anhui Province Key Laboratory of Major Autoimmune Diseases, Anhui Medical University, Hefei, China; b Management Center of Anhui Continuing Education Network Park, Anhui Open University, Hefei, China; c School of Chemistry, Chemical Engineering and Life Science, Wuhan University of Technology, Wuhan, China

**Keywords:** Acetylcholinesterase, butyrylcholinesterase, sulphonyl fluoride, anti-amyloid, Alzheimer’s disease

## Abstract

To discover novel scaffolds as leads against dementia, a series of δ-aryl-1,3-dienesulfonyl fluorides with α-halo, α-aryl and α-alkynyl were assayed for ChE inhibitory activity, in which compound **A10** was identified as a selective BuChE inhibitor (IC_50 _= 0.021 μM for eqBChE, 3.62 μM for hBuChE). SAR of BuChE inhibition showed: (i) *o*- > *m*- > *p*-; –OCH_3_ > –CH_3_ > –Cl (–Br) for *δ*-aryl; (ii) α-Br > α-Cl, α-I. Compound **A10** exhibited neuroprotective, BBB penetration, mixed competitive inhibitory effect on BuChE (*K*_i_ = 29 nM), and benign neural and hepatic safety. Treatment with **A10** could almost entirely recover the A*β*_1-42_-induced cognitive dysfunction to the normal level, and the assessment of total amount of A*β*_1-42_ confirmed its anti-amyloidogenic profile. Therefore, the potential BuChE inhibitor **A10** is a promising effective lead for the treatment of AD.

## Introduction

Alzheimer’s disease (AD) is the most prevalent chronic neurodegenerative disorder and the main cause of dementia. Treatment of AD remains one of the most urgent medical needs. With the acceleration of ageing, more and more people suffer from dementia. Currently, around 50 million people worldwide suffer from dementia, and the number is estimated to increase to more than 150 million by 2050. The huge incidence and prevalence of AD make it the seventh leading cause of death. However, AD cannot be cured, prevented, or even slowed down. Further, the economic burden of AD is really a major issue for health systems, with a total estimated cost of around one trillion U.S. dollars, which is expected to double in next 10 years^1^^,^^2^. Development and application of novel scaffolds for potential anti-AD agents have attracted significant attention for medicinal chemists.

AD has neuropathological features of extensive deposition of A*β* plaques in the neocortex and hierarchical neurofibrillary tangles in limbic and cortical association areas^3‒5^. The biological mechanisms of AD involve cholinergic dysfunction, amyloid plaques of A*β*, tau aggregation, deposition of neurofibrillary tangles (NFT), inflammatory response, and disturbances in the brain microenvironment^6‒8^. The “cholinergic hypothesis” is one of the most effective strategies to improve disease symptoms, involving cognitive and behavioural function. AD is mainly caused by cholinergic neuron loss and progressive decline in acetylcholine (ACh) in the forebrain^9^^,^^10^, the activity of ACh in the brain is terminated by the hydrolysis of two cholinesterase (ChEs), namely acetylcholinesterase (AChE) and butyrylcholinesterase (BuChE). Inhibition of AChE or/and BuChE is an efficient anti-AD strategy.

AChE, the major ChE, is mainly derived from regions of the neural synaptic junction and adult cerebral cortex that express intense activity^11^^,^^12^, while BuChE is mainly derived from glial cells of the brain, maintaining a close spatial relationship of BuChE in glial cells and facilitating BuChE-mediated hydrolysis, thereby regulating local ACh levels, which in turn maintain normal cholinergic function^13^^,^^14^. As AD progresses, the activity of AChE decreases, while the activity of BuChE increases in the hippocampus and temporal cortex, thus, BuChE can compensate the reduction of AChE activity^15^. In AChE knockout mice, no cholinergic hyperactivation was observed since BuChE can take over the hydrolysis of ACh^16^. More studies on the role of the BuChE in AD brains showed a positive correlation between selective BuChE inhibition and improved cognitive performance and memory^14^^,^^17–20^.

At present, the drugs used to treat AD are mainly aimed at the cholinergic system to improve the symptoms, of which there are four AChE inhibitors approved by the FDA in clinical drugs, including tacrine, donepezil, rivastigmine and galantamine^21‒23^, which can significantly relieve memory loss and improve cognitive function in mild-to-moderate patients but cannot completely cure AD. In addition, studies have shown that AChE can cause amyloid plaques^24^, and the expression of BuChE is related to A*β* plaques, NFT and cerebral amyloid angiopathy^25‒27^. The use of AChE and/or BuChE inhibitors can reduce these plaques. Therefore, AChE and BuChE are still the most valuable and predominating targets for the discovery of new anti-AD agents^28‒30^.

**Figure 1.**
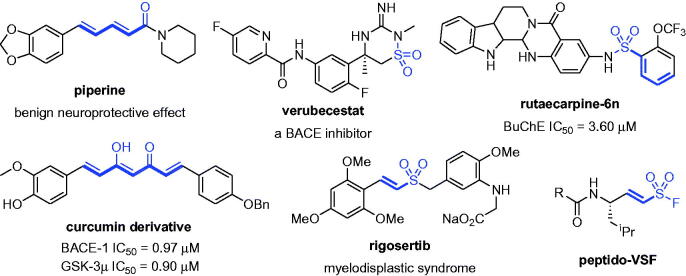
The structure characteristics of active compounds.

Molecular hybridisation is an approach for drug development in which two different active pharmacophores are clubbed together. Piperine is a kind of conjugated diene derivative that can improve oxidative nitrosation stress, restore neurotransmission and reduce neuro-inflammation^32^^,^^33^. Verubecestat with sulphonyl is a BACE-1 inhibitor which was evaluated for the treatment of AD in mild to moderate AD and prodromal AD^34^. Rutaecarpine-6n with dienyl sulphonyl was identified as the most potent BuChE inhibitor with IC_50_=3.60 µM and demonstrated remarkable neuroprotective effects as a potential drug candidate for AD^35^. A curcumin derivative with conjugated diene exerted neuroprotective activity through the balanced concurrent inhibitory activity against BACE-1 and GSK-3β, making it a promising drug candidate for AD^36^^,^^37^. When piperidine-1-carbonyl of piperine was substituted with sulphonyl fluoride, molecular docking showed that the sulphonyl fluoride unit can form more interactions with amino acids in the hBuChE target (Figure S1). Therefore, this vinyl/sulphonyl molecule in new chemical entities may be developed as ChE inhibitors with neuroprotective activity (Figure 1).

With the development of sulphur fluoride exchange (SuFEx), sulphonyl fluorides containing substances are currently attracting enormous attention among practitioners of both chemical biology and synthetic organic chemistry, but the chemistry of these compounds is quite unexplored^38^. Vinyl sulphonyl fluoride (VSF) is a class of novel scaffolds containing both olefin and sulphonyl fluoride. The exploration of biological activity of diverse vinyl sulphonyl fluoride scaffolds is highly desirable and of great significance, such as peptide-VSF, rigosertib, etc^44^. Sulphonyl fluorides have emerged as promising scaffolds for drug discovery^47^^,^^48^, and vinyl/sulphonyl hybrids may possess neuro-protection, therefore, it is rational to evaluate ChE activity of this novel dienylsulphonyl fluoride scaffold. In this work, a series of α-substituted *δ*-aryl-1,3-dienylsulphonyl fluorides were evaluated for their ChE inhibitory activity, analysed for their structure–activity relationship (SAR), and explained possible preliminary mechanism.

## Materials and methods

### Chemistry

All reactions were carried out under air atmosphere, unless otherwise specified. Reactions were checked by TLC on precoated silica gel plates, and spots were visualised by UV at 254 nm. Melting points of the products were measured on a micro melting point apparatus (SGW X-4) and uncorrected. ^1^H NMR and ^13 ^C NMR spectra were recorded in CDCl_3_ on a 500 MHz (for ^1^H), 471 MHz (for ^19 ^F), and 126 MHz (for ^13 ^C) spectrometer. All chemical shifts are reported in parts per million (*δ*) downfield from the signal of TMS as internal standards. Coupling constants are reported in Hz. The multiplicity is defined by *s* (singlet), *d* (doublet), *t* (triplet), or *m* (multiplet). MS experiments were performed on a TOF-Q ESI or CI/EI instrument. Reagents and solvents used in the reactions were all purchased from commercial sources and used without further purification, unless otherwise noticed. The purity (relative content) of active compounds was determined by HPLC on an Agilent 1200 instrument (column: Elite, RP-C18, 5 µm, 4.6 × 150 mm) through area normalisation method.

**Scheme 1.**
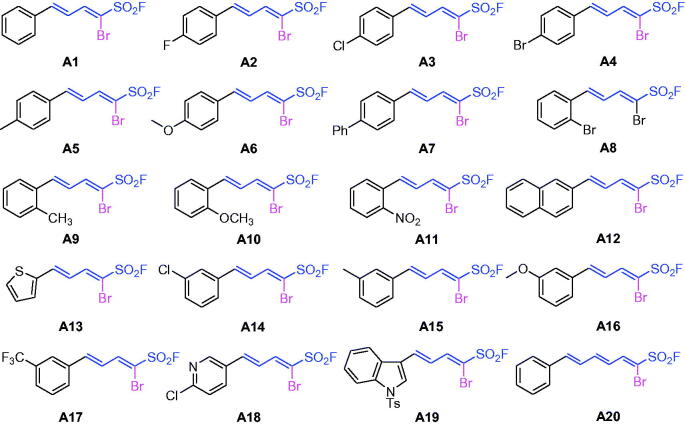
Chemical structures of series **A1–A20**.

**Scheme 2.**
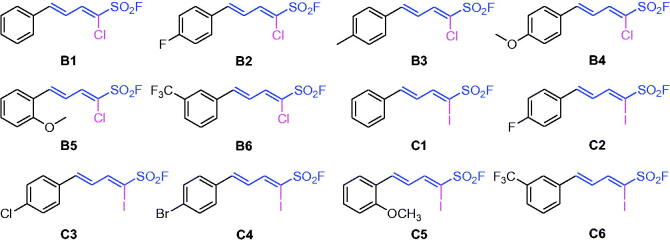
Chemical structures of series **B1–B6** and **C1–C6**.

**Scheme 3.**
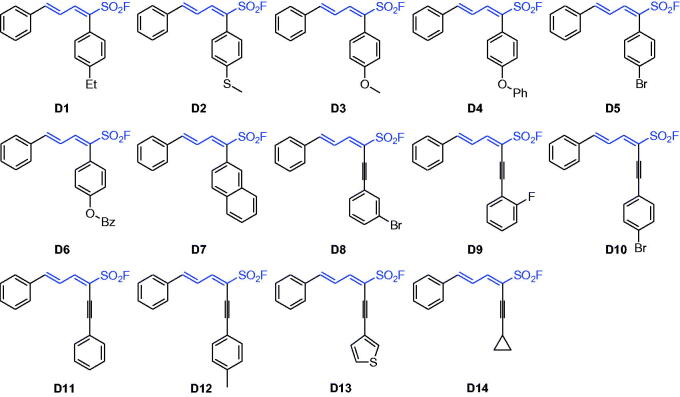
Chemical structures of series **D1**–**D14**.

In our recent work^45^, highly stereoselective δ-aryl-*α*-halo-1,3-dienylsulphonyl fluorides (**A1‒A20**, **B1‒B6** and **C1‒C6**, Schemes 1 and 2) were obtained in up to 100% *Z*-selectivity and high yields at room temperature from a pyrrolidine-mediated Knoevenagel-type condensation reaction of the readily available aldehydes and halomethanesulphonyl fluorides (3.0 eq.) in the presence of pyrrolidine catalyst (60 mol%) in good yields (56–96%). Suzuki coupling of arylboronic acids with *α*-bromo-1,3-dienylsulphonyl fluorides gave the *α*-aryl-1,3-dienylsulphonyl fluorides (**D1‒D7**, Scheme 3) under the catalysis of PdCl_2_(PPh_3_)_2_ in moderate to good yields (49–83%). Sonogashira reaction of alkynes with *α*-iodo-1,3-dienylsulphonyl fluorides gave the *α*-alkynyl-1,3-dienylsulphonyl fluorides (**D8‒D14**, Scheme 3) under the catalysis of CuI and PdCl_2_(Cy_3_P)_2_ in good yields (62–83%).

### EeAChE and eqBuChE inhibition assays

According to the Ellman’s method, assays were performed on AChE from electric eel (C3389-500UN; Sigma) and BuChE from equine serum (C4290-1KU; Sigma). The experiment was performed in 48-well plates in a final volume of 500 µL. Each well contained 0.036 U/mL of eeAChE or eqBuChE, and 0.1 M pH 8 PBS. They were pre-incubated for 20 min at different compound concentrations at 37 °C. Then, 0.35 mM acetylthiocholine iodide (ACh; A5751-1G; Sigma) or 0.5 mM butyrylthiocholine iodide (BuCh; 20820–1 G; Sigma) and 0.35 mM 5,5′-dithiobis(2-nitrobenzoic acid) (DTNB; D8130-1G; Sigma) were added. The DTNB produces the yellow anion 5-thio-2-nitrobenzoic acid along with the enzymatic degradation of ACh or BuCh. Changes in absorbance were measured at 410 nm after 20 min in a PerkinElmer VICTOR Nivo reader. The IC_50_ values were calculated graphically from inhibition curves (log inhibitor concentration vs percent of inhibition). A control experiment was performed under the same conditions without inhibitor and the blank contained buffer, DMSO, DTNB, and substrate.

### hAChE and hBuChE inhibition assays

AChE from human (C1682; Sigma), BuChE from human (B4186; Sigma), 5,5′-dithiobis(2-nitrobenzoic acid) (DTNB; D8130-1G; Sigma), acetylthiocholine iodide (ACh; A5751-1G; Sigma), and iodobutyrylthiocholine (BuCh; 20820–1 G; Sigma). The buffer is 0.1 M PBS (pH = 8), which is prepared into zymogen solution by gelatine (1% deionised water) and diluted to 0.125 units per ml with water. Solutions of ACh and BuCh iodide were prepared in deionised water to a final concentration of 3.75 µM. DTNB solution (5 µM) was prepared with 0.1 M pH 8 PBS. The test compound was dissolved at a concentration of 2 × 1 0^−1 ^M in DMSO and stored at temperatures ranging from −20 to 4 °C as a stock solution. The solution was diluted to 5 concentrations of 200, 100, 10, 1 and 0.1 µM in ethanol. Measurements were performed using 96-well plates. Buffer (40 µL), sample at a series of concentrations (10 µL), AChE (10 µL), and dibutyl succinate (20 µL) were successively added. The mixture was then incubated for 5 min at 37 °C. Then, 20 µL of ACh or BuCh were added and the reaction started. After incubation at 37 °C for 5 min, the absorbance was measured at 412 nm. For the blank value, 10 µL of water was used instead of the inhibitor solution. The activity of the inhibitor was expressed as a logarithmic plot of the percentage of enzyme activity (with 100% as the reference) versus the concentration of the compound. IC_50_ values were determined graphically from inhibition curves (log inhibitor concentration versus percent inhibition).

### Molecular docking study

A structure based *in silico* procedure was applied to discover the binding modes of the active compounds to AChE and BuChE enzyme active sites. The CDOCKER of Discovery Studio Client v18.1.0 (DS) was conducted to explain SAR of series compounds and further guide the design of more effective and concrete AChE and BuChE inhibitors. The ligand binding to the crystal structure of hAChE (PDB ID: 4eye) and hBuChE (PDB ID: 1p0i) were selected as template. The target enzyme was prepared with Prepare Protein of DS to ensure the integrity of target. The ligand was processed by Full Minimisation of the Small Molecular in DS. Then, title compounds were docked into the active site of protein using CDOCKER. The view results of molecular docking were extracted after the program running end, each docking result was analysed for interaction and their different pose. Those poses with the lowest -CDOCKER_INTERACTION_ENERGY values were regarded as the most stable and picked to analysis binding interactions with target enzyme visualised.

### Kinetic studies of eqBuChE inhibition

Kinetic studies were performed with the same test conditions, using six concentrations of substrate (0.1‒1 mM) and four concentrations of inhibitor (0‒0.08 mM). The apparent inhibition constants and kinetic parameters were calculated in the “Enzyme Kinetics” module of Prism8. The effect of compound **A10** concentration on the catalytic activity of BuChE at 37 °C was investigated. Assay conditions were same as described in the assay protocol except that the final concentration of enzyme was varied (0‒0.72 U/mL). Concentrations of compound **A10** were 0, 0.02, 0.04, 0.08 µM respectively, for the determination of reversible as well as irreversible binding of inhibitors at enzyme.

### Cytotoxicity assays

The cytotoxic effect was detected by MTT colorimetric assay. Human hepatoblastoma cells HepG2 and human normal hepatocyte L02 were maintained at 37 °C in a 5% humidified incubator containing 10% foetal bovine serum, 100 U/mL penicillin and 100 mg/mL streptomycin. HepG2 cells and L02 cells were seeded in 96-well plates at 1 × 10^4^ cells per well. After cell culture for 24 h, different compounds were diluted in DMEM for 24 h. 20 ml of 5 mg/mL MTT reagent was added for incubation for 4 h. After 4 h, the cell culture medium was removed and 150 ml of DMSO was added to dissolve formazan. The optical density was measured at 492 nm (OD492). Cell viability was calculated from three independent experiments. The density of formazan formed in blank group was set as 100% of viability. Cell viability (%) ¼ compound (OD492/blank (OD492) ×100%

Blank: cultured with fresh medium only.

Compound: treated with compounds or donepezil.

### Neuroprotection assay

PC12 cells were distributed into 96-well plates at a density of 1 × 10^4^ cells/well, and after the cells were incubated overnight, they were treated with different concentrations of compound **A10** (1 − 25 µM) for 3 h. Then, 100 µM H_2_O_2_ was added as a cytotoxic stimulus, and the culture was continued for 24 h in fresh medium containing this drug. Cell survival was measured by MTT assay after 24 h. The cells were incubated with 20 µL of 5 mg/mL MTT reagent for 4 h. After 4 h, the cell culture medium was removed, and 150 µL of DMSO was added to dissolve formazan. The optical density at 492 nm (OD492) was measured on a BiotekSynergy HTX multimode reader. Results were adjusted for OD measured in blank.

### PAMPA-BBB penetration assay

On the basis of previous work by Di et al.^^29^^, the penetration of drug into the brain was tested by a parallel artificial membrane permeation assay (PAMPA) of the blood–brain barrier (BBB). Six commercial drugs were used to validate the method, all of which were purchased from Aladdin reagent. DMSO and dodecane are produced by Energy Chemical Company. Porcine brain endothelial cells (PBL) are extracted from the polar lipids of Avanti. Polyvinylidene fluoride membranes (pore size 0.45 µM) were used at the late stage of donor 96-well filters, and acceptor recessed 96-well microplates were purchased from Microwell, USA. The 96-well UV plate (COSTAR) was manufactured by Corning Corporation, USA, and the commercially available drugs and test compounds were initially dissolved in DMSO at a concentration of 20 mg/mL. The solution was then diluted 200-fold with PBS (pH 7.4 ± 0.1)/EtOH (70/30, *v/v*) solution to a final concentration of 100 µg/mL.

The test compound was dissolved in DMSO at 5 mg/mL and diluted 200-fold with universal buffer (final concentration 25 µg/mL) to prepare the secondary stock solution; 200 µL of the secondary stock solution was added to the donor well; the acceptor plate was coated with 4 µL of 20 mg/mL dodecyl porcine polar brain, and then, 200 µL of pH 7.4 universal buffer was added to the acceptor well; the acceptor plate was carefully placed on the donor plate to form a “sandwich” and maintained for 18 h; the drug concentrations in the acceptor, donor and reference wells were determined using a UV microplate reader; the experiment was repeated three times, and the concentrations of the test compound in the donor and acceptor wells were measured using a UV plate spectral reader (PerkinElmer VICT or Nivo, Finland). In at least three independent experiments, each sample was analysed at three wavelengths in a four well.

### In vivo acute toxicity evaluation

A total of 20 mice (F: M = 1: 1) weighing 20‒25 g were randomly divided into control group (*n* = 10) and experimental group (*n* = 10). Compound **A10** was suspended in a mixture of DMSO, PEG 400 and physiological saline (10/50/40, *v/v/v*). After fasting for 8‒12 h, the mice were intragastrically administered with the preparation or test compound **A10** 1.0 g/kg on the first day. The behaviour, appearance and body weight changes of the mice were observed and recorded for 2 weeks. The body weights of the mice in the control group and experimental group were compared and summarised with GraphPadPrism8.0 software.

### In vivo hepatotoxicity evaluation

*In vivo* hepatotoxicity was assessed in male ICR mice (20‒25 g), also divided into blank experimental groups, mice were fasted for 24 h, and compound **A10** was suspended in a mixed solution of DMSO, PEG 400, and normal saline (10/50/40, *v/v/v*). The combination was administered intragastrically at a dose of 30 mg/kg body weight and the same amount of vehicle (po). Heparinised serum was collected from the retrobulbar plexus at 8, 22, and 36 h after administration for hepatotoxicity evaluation. The activities of alanine aminotransferase (ALT) and aspartate aminotransferase (AST), indicators of liver injury, were measured with corresponding kits (EF551 and EF550 for ALT, EH027 and EH548 for AST). Data were processed using a biochemical analyser (Hitachi 7020, Japan). After the last collection of post-globular blood, mice were sacrificed and livers were taken for immunohistochemical morphological observation. We isolated two 3-mm sections of each liver from the hilum to the edge of the left lateral lobe using an ultra-thin semi-automatic microtome (LeicaRM2245, Germany), immediately placed them in 10% buffered formaldehyde, fixed them for 2 days, and embedded them in paraffin blocks using a paraffin-embedding station (LeicaEG1150H, Germany). Subsequently, five µM sections were prepared from these paraffin sections, deparaffinized, stained with haematoxylin and eosin or using the periodic acid-Schiff glycogen staining method.

### Animal studies

All experiments were performed according to the National Institutes of Health Guide for the Care and Use of Laboratory Animals. The Measures for the Care and Treatment of Laboratory Animals were approved by the Animal Care and Use Committee of Anhui Medical University. Male ICR mice were used in the animal centre of Anhui Medical University (Hefei). Male mice (18‒24 g) aged 6‒8 weeks, 10 mice per cage, room temperature 22 ± 2 °C, light/dark (12:12 h) cycle. These animals had access to food and water prior to testing. The ambient temperature and relative humidity (50%) of the room remained consistent throughout all tests. Behavioural experiments: MWM was used for cognitive function, and mice were randomly selected for behavioural experiments. Each experimental group consisted of 8‒10 mice/dose. The experimental time was from 08:00 to 14:00, and the mice were sacrificed by cervical dislocation immediately after the end of the experiment. For A*β*_1-42_ oligomerization injury test, positive and test compounds were suspended in a mixture of DMSO and 0.5% sodium carboxymethylcellulose (1/99, *V/V*) before the experiment, and 40 male mice were randomly divided into five groups: (i) blank control group (po), (ii) saline (icv) + vehicle (appropriate amount, po), sham-operation group, (iii) oligomerized A*β*_1-42_ peptide (10 µg/mouse, icv) + vehicle (appropriate amount, po), model group, (iv) oligomerized A*β*_1-42_ peptide (10 µg/mouse, icv) + donepezil (15 mg/kg, po), donepezil group, (v) oligomerized A*β*_1-42_ peptide (10 µg/mouse, icv) + **A10** (10 mg/kg, po), **A10** group. A*β*_1-42_ aggregation was induced by dissolving the A*β*_1-42_ peptide in DMSO as a stock solution of 5 mM and incubating it in saline at a final concentration of 2 mg/mL for 24 h at 37 °C.

The behavioural study was a water maze experimental behavioural study on days 10 − 15, including a 5-day learning and memory training, and a test assessment on day 6. MWM consists of a water-filled pool (grey, circular, 1.20 m in diameter, 0.60 m in height) and a platform with adjustable height and movable position. The pool was divided into four equal quadrants (compass position: NE, NW, SE, SW) using a computerised video tracking system (SMART, version 3.0; Panlab, Spain). The pool was filled with water, generally about 48 cm below the edge, to prevent animals from jumping out, and the water temperature was maintained at 22 ± 1 °C. The escape platform was made of a transparent plexiglass (11 cm in diameter and 47 cm in height) and was placed in a fixed position (the centre of the northwest quadrant, that is, the target quadrant), which was soaked 1 cm below the water surface. The pool is placed in a larger room, and there is no light shadow on the pool water surface, and there are four reference objects with different geometric patterns on the pool wall. During the training, at the beginning of each day, the rats were arbitrarily placed in the water facing the pool wall from one of the four quadrants (NE, NW, SE, SW), and the platform was placed in the southeast quadrant, and each experimental rat swam for a total of 60 s to find the hidden platform. If the mouse still failed to find the platform in the pool or climbed the platform within 60 s of swimming in the water, the mouse was guided to stand on the platform for 15 s. The time to reach the hidden platform (i.e. escape latency), the distance to reach the hidden platform, the distance in the target (NW) area, and the average speed were recorded. On day 7 (24 h after the last training session), the platform was removed from the pool and a probe trial (Drogoff test) was performed. Each mouse was allowed to swim once, and if the previous platform position was not found within 60 s, a latency score of 60 s was given to measure the latency to first cross the previous platform position (i.e. the target area), the number of times it crossed the target area, the time spent in the target NW quadrant, the total distance, the distance spent in the NW quadrant, the entry into the NW quadrant, and the mean speed, and compared across experimental groups.

**Figure 2.**
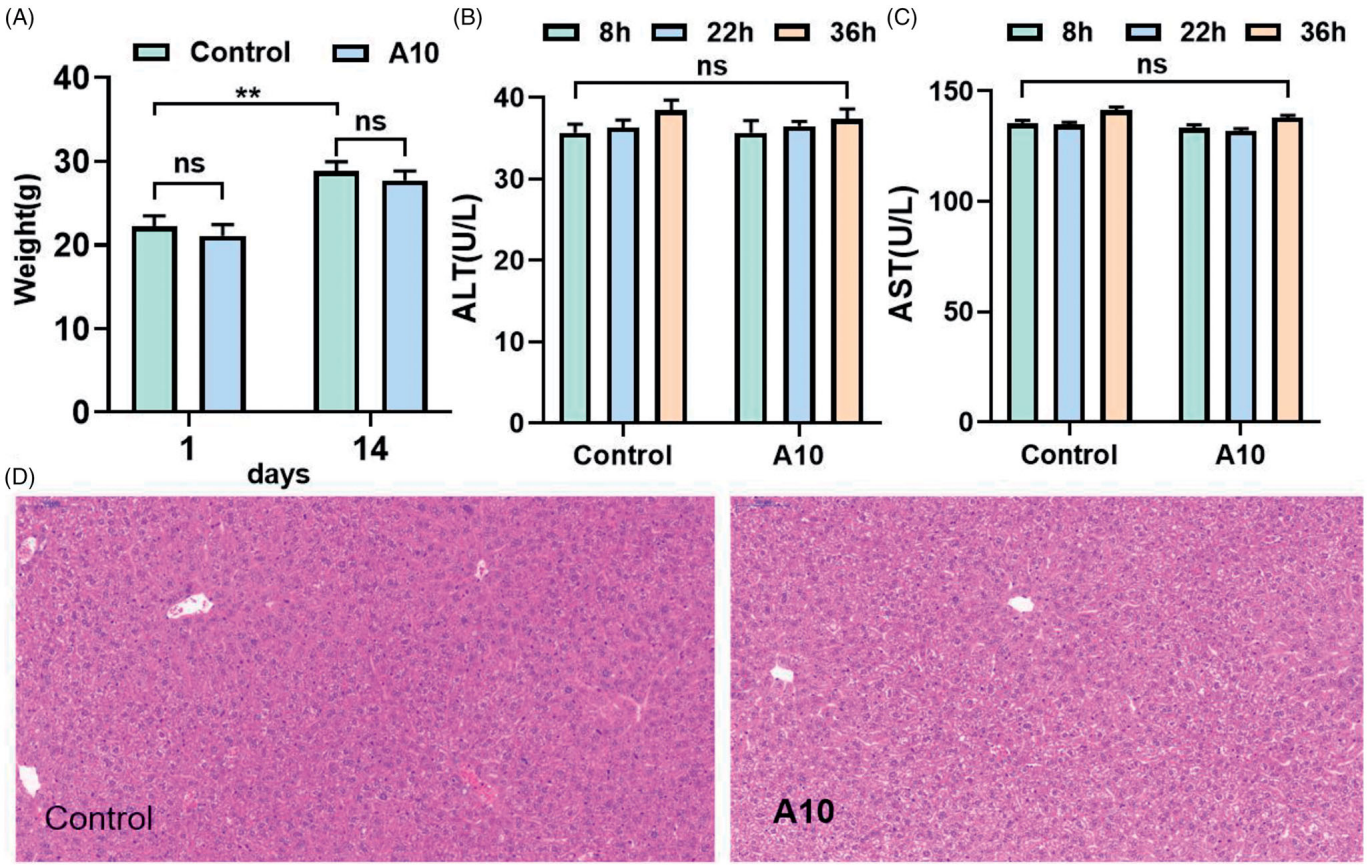
*In vivo* acute toxicity evaluation. (**A**) Body weight of ICR mice (g) ‒ measurement time point (day, **p < 0.01); (B) ALT activity at 8, 22 and 36 h after single dosing (control) or **A10**; (C) AST activity at 8, 22 and 36 h after single dosing (control) or **A10**; (D) Histomorphological observation of mouse liver treated with **A10** vector (control); The HE-stained field was 100 µM.

In the experiment of A*β*_1-42_ oligomeric injury, all mice were sacrificed after the end of behavioural study, and the brains were taken to determine the total content of A*β*_1-42_ with a mouse ELISA kit (Wuhan Huamei Biotechnology Co., Ltd.). Each brain tissue specimen was completely homogenised with a grinder with 10 times PBS (pH = 7.4 ± 0.1) and then centrifuged at 5000×*g* for 5 min. The supernatant was separated for use. The detection procedure was consistent with the instructions, and the standard curve is shown in Figure 2(A). Brain tissue A*β*_1-42_ content was calculated according to the linear regression equation. All values were expressed as mean ± SEM using GraphPadPrism8.0 software.

### Statistical analysis

Data are reported as mean ± SEM of at least three independent experiments and data analysis was performed with GraphPad Prism 8 software.

## Results and discussion

### Chemistry

Recently, we reported a protocol for stereoselective construction of highly functionalised dienyl sulphonyl fluorides with wide scope and excellent functional group compatibility^45^. The *α*-bromo-1,3-dienylsulphonyl fluorides (series **A1**–**A20** as shown in Scheme 1 and Supporting Information) were synthesised by a pyrrolidine-mediated Knoevenagel-type condensation employing various aldehydes to react with bromomethanesulphonyl fluoride.

Subsequently, as shown in Scheme 2 and Supporting Information, the α-chloro-1,3-dienylsulphonyl fluorides (series **B1**–**B6**) and the α-iodo-1,3-dienylsulphonyl fluorides (series **C1**–**C6**) were synthesised by the above condensation from chloromethanesulphonyl fluoride and iodomethanesulphonyl fluoride, respectively.

Finally, the *α*-bromo-1,3-dienylsulphonyl fluorides were used as versatile building blocks in SuFEx click chemistry, for example, Suzuki reaction with arylboronic acids and Sonogashira reaction with alkynes for assembly of highly functionalised dienyl sulphonyl fluoride derivatives **D1**–**D7** and **D8**–**D14**, respectively (Scheme 3 and Supporting Information).

For above sulphonyl fluoride, S–F bonds were incredibly stable and can tolerate unusually harsh reaction conditions^40^. Unlike more common S–Cl counterparts, sulphonyl fluorides series **A**–**D** were hardly hydrolysed in ethanol, aqueous solution and buffer solution (PBS, pH 7.4).

### Inhibitory activity against AChE and BuChE

The inhibitory potency of dienyl sulphonyl fluorides with α-substituent was assessed by Ellman’s assay on *Electrophorus electricus* AChE (*Ee*AChE) and equine BuChE (eqBuChE). The IC_50_ values were obtained and compared to the reference donepezil, which is a FDA-approved selective AChE inhibitor that simultaneously binds to catalytic active and peripheral anionic sites, providing moderate inhibition of Aβ aggregation^49^^,^^50^. The IC_50_ values of all tested compounds against *Ee*AChE and eqBuChE were summarised in Table 1.

**Table 1.** Inhibitory activities against *Ee*AChE and eqBuChE of compounds **A1–A30**, **B1–B6**, **C1–C6** and **D1–D14**.^a^

	(IC_50_, µM or % inhibition at 20 µM)	
Compound	AChE^b^	BuChE^c^
**A1**	na^d^	0.082 ± 0.007
**A2**	na	0.43 ± 0.01
**A3**	33.9 ± 0.9%	42.2 ± 2.1%
**A4**	na	19.2 ± 1.2%
**A5**	na	1.72 ± 0.34
**A6**	na	0.31 ± 0.04
**A7**	11.9 ± 0.5%	45.2 ± 2.0%
**A8**	0.107 ± 0.002	0.44 ± 0.18
**A9**	23.2 ± 4.0%	0.30 ± 0.11
**A10**	40.8 ± 3.3%	0.021 ± 0.006
**A11**	0.15 ± 0.01	0.27 ± 0.15
**A12**	na	2.91 ± 0.26
**A13**	na	0.32 ± 0.06
**A14**	17.9 ± 2.0%	0.11 ± 0.02
**A15**	na	0.60 ± 0.12
**A16**	35.9 ± 1.4%	0.30 ± 0.04
**A17**	12.1 ± 0.7%	0.43 ± 0.03
**A18**	0.079 ± 0.002	42.8 ± 3.6%
**A19**	46.2 ± 0.4%	46.0 ± 1.6%
**A20**	18.7 ± 2.4%	12.5 ± 4.2
**B1**	35.9 ± 1.4%	0.44 ± 0.04
**B2**	18.7 ± 1.7%	0.51 ± 0.01
**B3**	20.6 ± 0.8%	2.21 ± 1.27
**B4**	30.1 ± 3.8%	0.54 ± 0.19
**B5**	34.2 ± 0.4%	0.13 ± 0.01
**B6**	26.8 ± 2.7%	0.50 ± 0.21
**C1**	23.4 ± 2.8%	1.76 ± 0.62
**C2**	17.0 ± 1.7%	0.45 ± 0.03
**C3**	38.9 ± 2.5%	3.07 ± 1.72
**C4**	10.5 ± 3.6%	0.57 ± 0.03
**C5**	39.5 ± 0.3%	0.19 ± 0.04
**C6**	24.0 ± 6.0%	0.50 ± 0.04
**D1**	25.1 ± 1.6%	1.18 ± 0.33
**D2**	25.5 ± 0.4%	28.2 ± 1.89%
**D3**	47.9 ± 0.4%	33.7 ± 1.8%
**D4**	36.7 ± 2.8%	41.2 ± 1.1%
**D5**	29.0 ± 3.4%	30.5 ± 0.9%
**D6**	18.7 ± 2.1%	29.7 ± 3.8%
**D7**	42.3 ± 0.5%	7.4 ± 0.2%
**D8**	2.73 ± 0.10	4.56 ± 2.65
**D9**	47.7 ± 1.9%	31.7 ± 1.0%
**D10**	36.9 ± 1.2%	22.6 ± 0.7%
**D11**	30.4 ± 0.1%	24.3 ± 0.4%
**D12**	43.5 ± 4.0%	34.7 ± 0.1%
**D13**	14.7 ± 0.2%	31.3 ± 0.4%
**D14**	1.54 ± 0.32	0.43 ± 0.08
Donepezil	0.029 ± 0.006	9.83 ± 1.28
Rivastigmine	15.9 ± 1.2	0.052 ± 0.025

^a^Each IC_50_ value is the mean ± SEM from at least three independent experiments

^b^AChE from electric eel

^c^BuChE from horse serum

^d^na: no inhibitory activity (%) against either *Ee*AChE or eqBuChE at 20 µM.

Enzymatic assays revealed that all dienyl sulphonyl fluorides showed inhibitory activities against cholinesterase, among them, the majority exhibited strong inhibitory activity against BuChE, showing selectivity towards BuChE. It was obvious from the data that compounds **A1** and **A10** exhibited the best inhibitory activity against BuChE with IC_50_ values of 0.082 and 0.021 µM, respectively, close to the positive control rivastigmine (IC_50_=0.058 µM); compound **A18** exhibited the best inhibitory activity against AChE with IC_50_ values of 0.079 µM, close to the positive control donepezil (IC_50_=0.026 µM); while compounds **A8** and **A11** exhibited dual AChE and BuChE inhibitory activity (IC_50_ values for AChE and BuChE, 0.107 and 0.44 µM, 0.15 and 0.27 µM, respectively). From the inspection of the chemical structures, it can be concluded that the BuChE inhibitory activity was affected by the substituent groups at α- and *δ*-positions of the dienyl sulphonyl fluorides (Table 1). From Table 1, it is intuitive that the substituent of *δ*-aryl ring at the dienyl sulphonyl fluorides plays more important influence on the activity.

### SARs of novel 1,3-dienylsulphonyl fluorides

As shown in Scheme 1, series **A1**–**A20** were the α-Br-1,3-dienylsulphonyl fluorides and the substituent of δ-aryl ring played important role in the cholinesterase activity and the selectivity. Most of the α-Br-1,3-dienylsulfonyl fluorides exhibited selective BuChE inhibitory activity except for **A18** as an AChE inhibitor, **A8** and **A11** as dual AChE and BuChE inhibitors. The structure–activity relationship (SAR) analysis showed in Table 1: (i) the effect of substituent position at δ-aryl ring on BuChE inhibition: *ortho*- > *meta*- > *para*-, such as **A10 **>** A16 **>** A6** for –OCH_3_, **A9 **>** A15 **>** A5** for –CH_3_, **A8 **>** A4** for –Br, **A14 **>** A3** for –Cl; (ii) the effect of substituent at *δ*-aryl ring on BuChE inhibition: –OCH_3_ >–CH_3_ >–Cl (–Br), such as **A10 **>** A9 **>** A8** for *ortho*-position, **A6 **>** A5 **>** A3** (**A4**) for *para*-position, except for **A14** (–Cl) >**A16** (–OCH_3_) >**A15** (–CH_3_) for *meta*-position; (iii) compound **A1** (*δ*-phenyl-α-Br-dienyl) showed benign BuChE inhibition (IC_50_=0.082 µM), the increase of alkenyl (**A20**, ζ-phenyl-α-Br-trienyl) led to decrease the activity (IC_50_=12.49 µM); (iv) when the *δ*-phenyl ring was replaced with *δ*-pyridyl ring, compound **A18** showed inverse selectivity (IC_50_=0.079 µM for AChE). The SARs on α-substituent group of dienyl sulphonyl fluorides would be further studied.

Series **B** and series **C** are α-Cl and α-I substituted dienyl sulphonyl fluorides, respectively. As shown in Scheme 2 and in Table 1, the α-Cl or α-I substituent of α-Br led to decrease BuChE inhibitory activity, such as **A1 **>** B1 **>** C1** for *δ*-phenyl, **A2 **>** B2 **>** C2** for *δ*-4-F-phenyl, **A10 **>** B5 **>** C5** for *δ*-2-OCH_3_-phenyl, **A17 **>** B6 **>** C6** for *δ*-3-CF_3_-phenyl, **A5 **>** B3** for *δ*-4-CH_3_-phenyl, **A6 **>** B4** for *δ*-4-OCH_3_-phenyl, except for **A3 **<** C3** for *δ*-4-Cl-phenyl and **A4 **<** C4** for *δ*-4-Br-phenyl.

**Figure 3.**
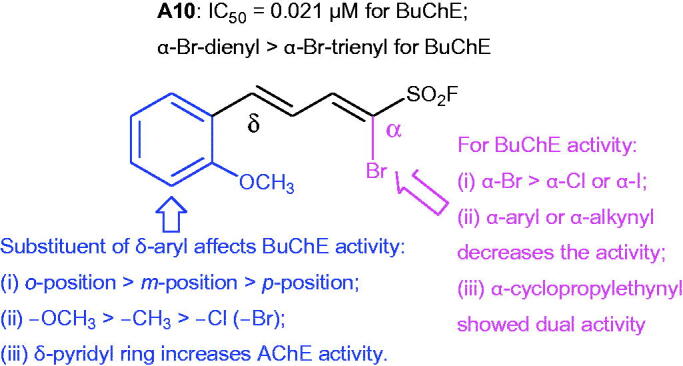
SARs of the BuChE inhibitor based on compound **A10**.

Series **D** are obtained from Suzuki coupling reaction of *δ*-phenyl-α-Br-dienyl sulphonyl fluoride (**A1**) and Sonogashira reaction of *δ*-phenyl-α-I-dienyl sulphonyl fluoride (**C1**). As shown in Scheme 3 and in Table 1, compared to compound **A1**, BuChE inhibition of compounds **D1–D7** with α-aryl and **D8–D14** with α-alkynyl decreased, amongst them, compound **D14** with α-cyclopropylethynyl showed dual cholinesterase inhibitory activity (IC_50_ values for AChE and BuChE, 1.54 and 0.42 µM, respectively). Based on compound **A10**, the structure–activity relationship (SAR) was illustrated in Figure 3.

### Inhibition of hBuChE and hAChE

To determine the potency and selectivity of compounds **A10** and **A18** for the human enzymes, ChE inhibitory activity was assessed by Ellman’s assay on recombinant human AChE (hAChE) and BuChE from human serum (hBuChE)^49^^,^^50^. Compared to the positive control rivastigmine, compound **A10** showed close inhibitory effect on hBuChE and stronger inhibitory effect on hAChE at 20 µM (Table 2). Hence, compound **A10** was identified as a selective hBuChE inhibitor.

**Table 2.** Inhibitory activity on hAChE and hBuChE^a^

Compound	IC_50_, µM (or inhibition% at 20 µM)	
	hAChE^b^	hBuChE^c^
donepezil	0.016 ± 0.004	11.06 ± 2.43
rivastigmine	11.2 ± 1.2%	2.95 ± 0.46
**A10**	49.7 ± 2.7%	3.62 ± 0.32
**A18**	14.3 ± 0.9%	na^d^

^a^ Each IC_50_ value is the mean ± SEM from at least three independent experiments

^b^ hAChE from recombinant human AChE (hAChE)

^c^ hBuChE from human serum

^d^ na: no activity.

### Molecular docking of compounds A10 and A18

**Figure 4.**
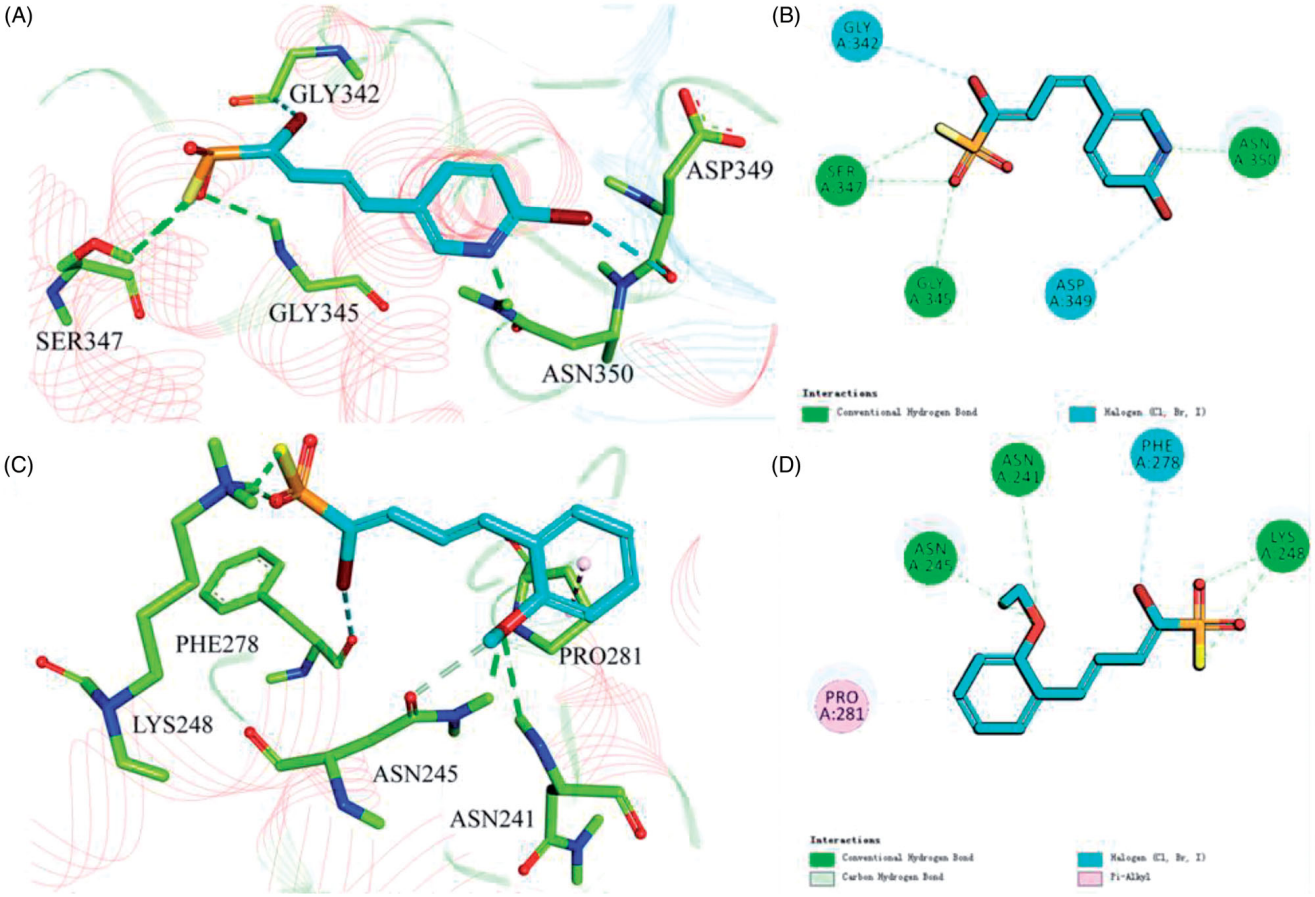
3D diagram of compounds **A18** (A) and **A10** (C) into hAChE (PDB: 4ey4) and hBuChE (PDB: 1p0i) performed respectively. Active site residues of hAChE and hBuChE are presented as sticks with carbon atoms represented in light green (light blue for **A18** and **A10**). The green dashed lines represent hydrogen bonds, the light blue dashed lines represent halogen interaction bonds and the light pink dashed line represents π-alkyl interaction. 2D diagram of compounds **A18** (B) and **A10** (D) into hAChE (PDB: 4ey4) and hBuChE (PDB: 1p0i) performed respectively.

To better understand the capacity of **A18** targeting hAChE and that of **A10** targeting hBuChE, their binding modes were investigated by the CDOCKER molecular docking module in Discovery Studio 2018. As shown in Figure 4(A,B), compound **A18** could insert into hAChE, sulphonyl fluoride (SO_2_F) moiety as a hydrogen bond acceptor can form hydrogen bond interaction with Gly345 and Ser347, and the N atom of the pyridine ring also form hydrogen bond interaction with Asn350. While, compound **A10** could precisely insert into hBuChE (Figure 4(C,D)), sulphonyl fluoride (SO_2_F) moiety as a hydrogen bond acceptor can form hydrogen bond interaction with Lys248, the 2-position methoxy group of the benzene ring can form hydrogen bond interaction with Asn241 and Asn245, and the benzene ring itself can form π-alkyl interaction with Pro281.

### Kinetic study of eqBuChE inhibition

**Figure 5.**
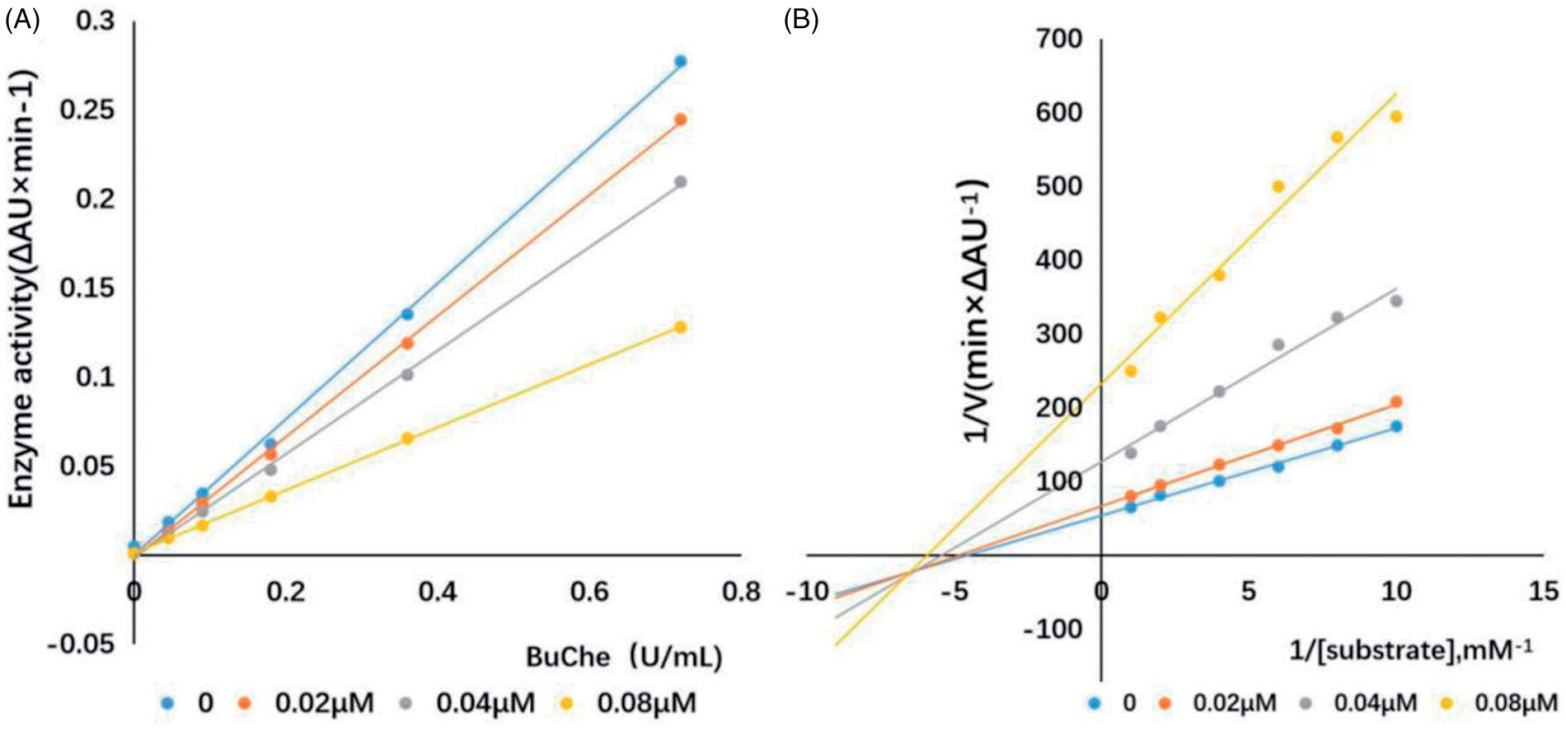
Relationship between eqBuChE inhibition and various concentrations of **A10** (A). Lineweaver‒Burk plots of eqBuChE inhibition kinetics of **A10** (B). Reciprocals of enzyme activity (eqBuChE) vs reciprocals of substrate (butyrylthiocholine iodide) with different concentrations (0‒0.08 μM) of inhibitor.

To determine the kinetics of BuChE inhibition, enzyme kinetic studies were performed on the active compound **A10**. As shown in Figure 5(A), in the presence of different concentrations of **A10**, the change curve of enzyme activity with enzyme concentration (0, 0.045, 0.090, 0.18, 0.36 and 0.72 U/mL) was a series of straight lines and intersected at one point, and the line slope decreased with the increase of inhibitor concentration, indicating that compound **A10** was a reversible BuChE inhibitor. Kinetic types of enzyme inhibition were obtained by the improved Ellman method and Lineweaver-Burk secondary diagrams, and typically, Lineweaver − Burk curves can be represented by reciprocal rates versus reciprocal substrate concentrations^50^^,^^51^. As shown by Figure 5(B), with the increase of compound **A10** concentration, Lineweaver–Burk plot showed higher slope (decreased *V*_max_) and higher intercept (higher *K*_m_), and the trend was usually attributed to mixed inhibition, and the dissociation constant *K*_i_ of compound **A10** was estimated to be 29 nM in Lineweaver–Burk secondary plot.

### Cytotoxicity assays

**Figure 6.**
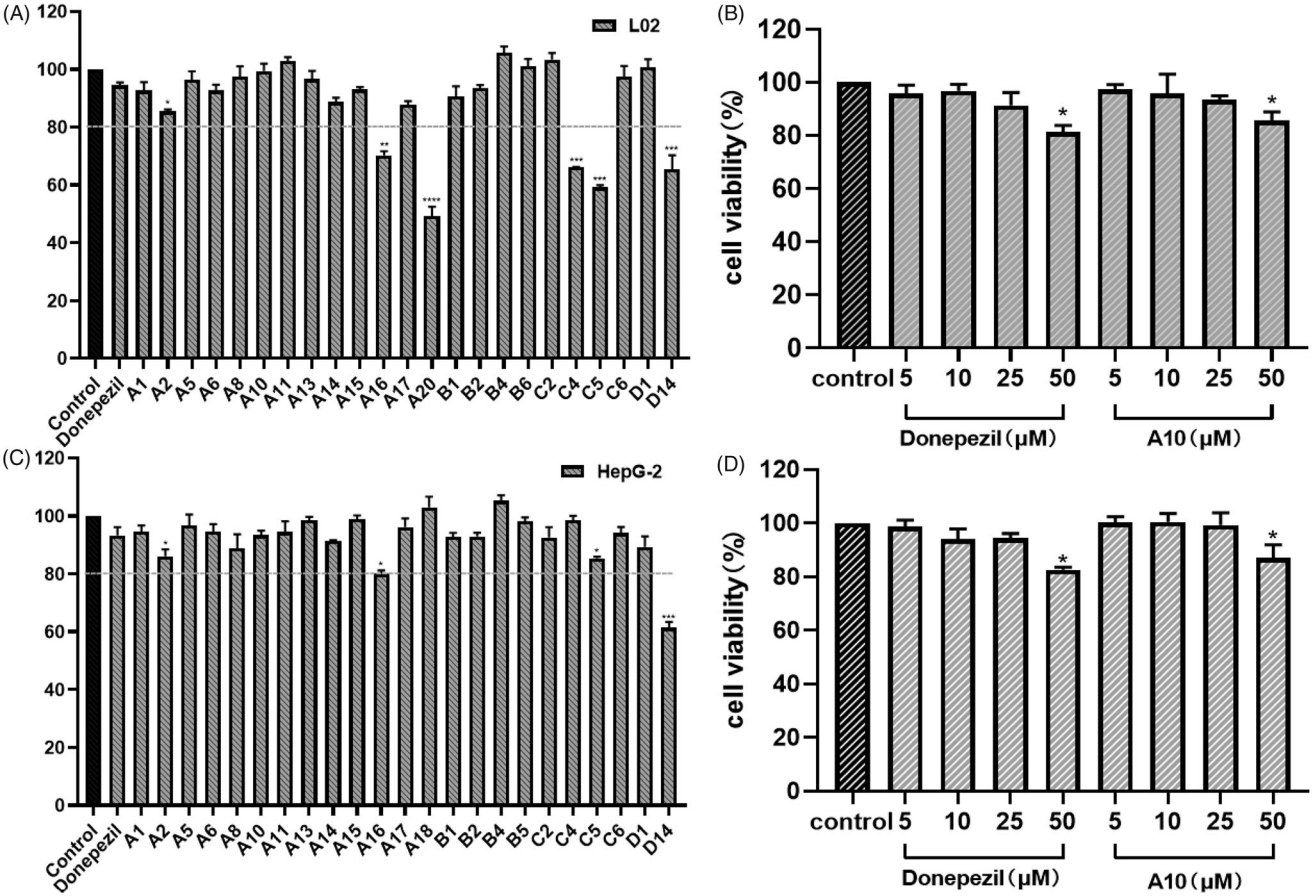
The cytotoxic effect of the better active compounds on L02 (A) cells and HepG2 (B) cells for 24 h was determined at a concentration of 25 µM, and untreated cells were used as controls. L02 (C) cells and HepG2 (D) cells were treated with donepezil and **A10** at concentrations ranging from 1 to 50 µM for 24 h. Untreated cells were used as controls. Results were expressed as a percentage of cell survival versus untreated cells (control) and as mean ± SEM (*n* = 3, *p < 0.05, **p < 0.01, ***p < 0.001, ****p < 0.0001 vs. control group).

In order to study the safety, the cytotoxicity of compounds with better activity against human normal hepatocyte L02 and human hepatoblastoma HepG2 was assayed by 3–(4,5-dimethylthiazol-2-yl)-2,5-diphenyltetrazolium bromide (MTT)^52^, as shown in Figure 6(A,B). On the basis of activity and cytotoxicity, compound **A10** was selected as the representative compound. As shown in Figure 6(C,D), the cell survival rate of compound **A10** did not decrease at 10 and 25 µM concentrations, and decreased to 87.1 and 85.6%, respectively, when the concentration of **A10** increased to 50 µM. The results showed that the target compound **A10** had broad therapeutic safety against L02 cells and HepG2 cells.

### Neuroprotective study

**Figure 7.**
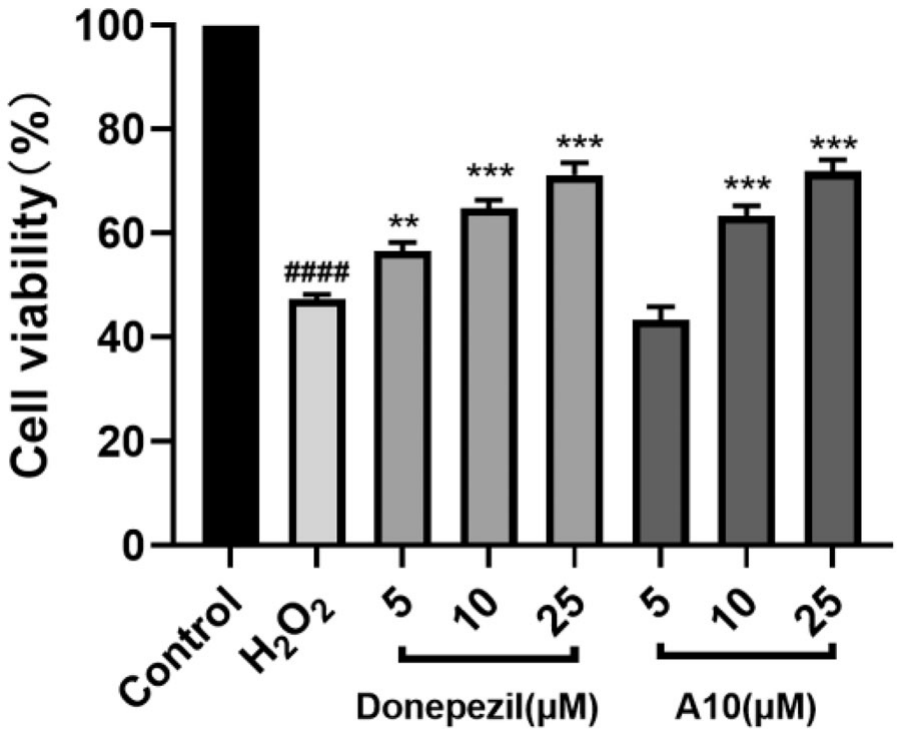
Neuroprotective effect of donepezil and **A10** on PC12 neurons. Results represent mean ± SEM (*n* = 3, ^####^*p* < 0.0001 vs. control group; **p < 0.01, ***p < 0.001 vs. model group).

The protective effect of compound **A10** on free radical damage was evaluated by measuring the protective ability of compound **A10** against H_2_O_2_ damage^49^^,^^55^. As shown in Figure 7, the survival rate of cells treated with 100 µM H_2_O_2_ was significantly decreased to 47.3%, and the survival rate of cells treated with donepezil was increased to 71.2%. After treatment with different concentrations of compound **A10**, the cell survival rate was gradually increased, 63.3 and 71.8%, respectively, slightly better than that of positive drug donepezil. The results showed that compound **A10** had a good protective effect on H_2_O_2_-induced PC12 cell damage.

### PAMPA-BBB penetration assay

**Figure 8.**
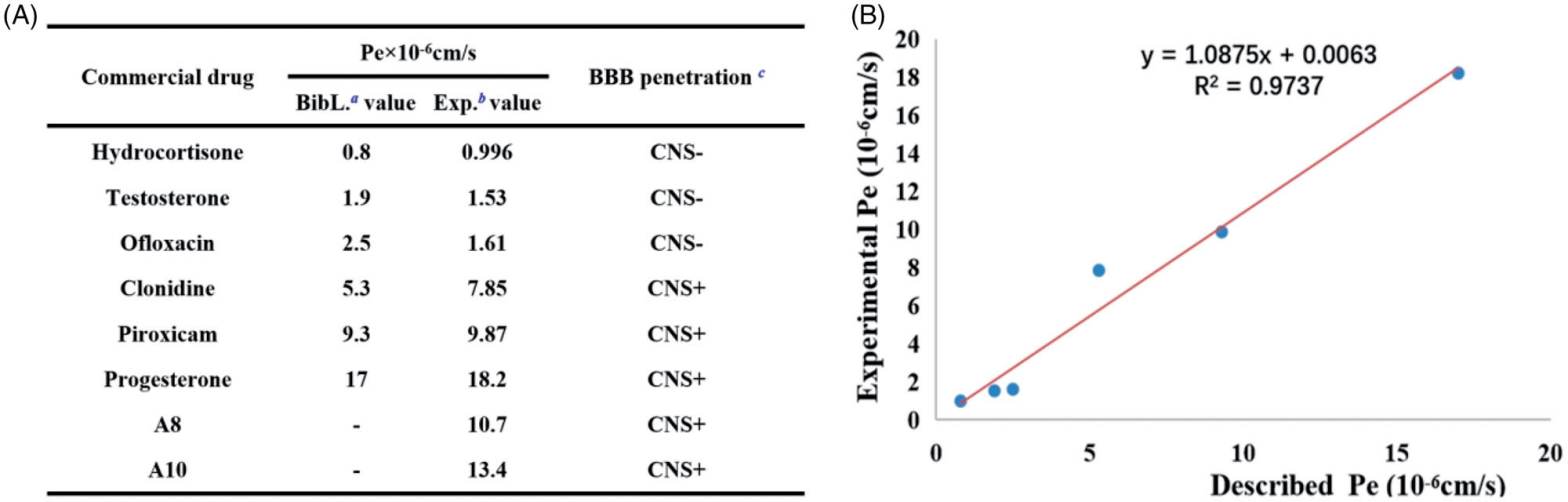
PAMPA-BBB penetration study of **A8** and **A10**. (A) Results of the PAMPA-BBB assay for six commercial drugs used in the experimental procedure validation and **A8** and **A10**. (**B**) Linear correlation presenting experimental versus bibliographic data of commercial drugs. ^a^Bibl. values are reported data from the reference; ^b^All tests were obtained from three independent experiments; *^c^*“CNS +” (high BBB permeation): *Pe* (× 10^‒6 ^cm/s) >4.0; “CNS” +/‒ “(uncertain BBB permeation)”: *Pe* (× 10^‒6 ^cm/s) from 2.0 to 4.0; “CNS ‒” (low BBB permeation): *Pe* (× 10^‒6 ^cm/s) <2.0.

In view of the important role of blood–brain barrier (BBB) in the treatment of AD and the fact that the central nervous system is the final target of anti-AD's drugs, the BBB penetration ability of compounds **A8** and **A10** was evaluated using a parallel artificial membrane penetration assay of blood-brain barrier (PAMPA-BBB)^30^^,^^56^, and six commercial drug validation experimental procedures with reported values were selected, and the curve between the experimental data and the reported values produced a good linear correlation, *Pe* (Exp.)=1.0875 *Pe* (Bib.) + 0.0063 (*R*^2^=0.9737) (Figure 8(B)). When the value of *Pe* is greater than 4 × 10^−6 ^cm/s, corresponding compounds could pass through the blood-brain barrier. According to this procedure, compounds **A8** and **A10** were tested for their permeability. As shown in Figure 8(A), the *Pe* values of compounds **A8** and **A10** was 1.07 × 10^−5 ^cm/s and 1.37 × 10^−5 ^cm/s, respectively, indicating that both had benign blood–brain barrier penetration ability.

### *In vivo* acute toxicity evaluation

A single-dose acute toxicity experiment was performed using ICR mice to evaluate the in vivo toxicity of compound **A10**. After intragastric administration of compound **A10** (1.0 g/kg), the general condition of the rats was good, and no significant change in appearance and activity. As shown in Figure 2(A), the body weight of mice in vehicle group and **A10** group has been increasing during 14 days before treatment, but the difference of body weight changes was not significant, indicating that compound **A10** was well tolerated *in vivo* at high doses (1.0 g/kg). In addition, alanine aminotransferase (ALT) and aspartate aminotransferase (AST) levels were measured, as shown in Figure 2(B,C), serum ALT and AST were directly proportional to the degree of liver injury. Heparinised serum was collected at 8, 22 and 36 h after administration. Not only between vehicle group and **A10** group at each time point, but also in **A10** group at each time point, no significant difference was observed (*p* > 0.05). The hepatotoxicity of compound **A10** was observed morphologically using immunohistochemistry. After 36 h of administration (control group) or paraffin sections of compound **A10** (30 mg/kg), immunohistochemical staining results in Figure 2(D) showed that compound **A10** didn’t show central necrosis or significant steatosis in and around the intermediate zone around the hilum, suggesting that compound **A10** has high *in vivo* safety.

### Behavioural studies

**Figure 9.**
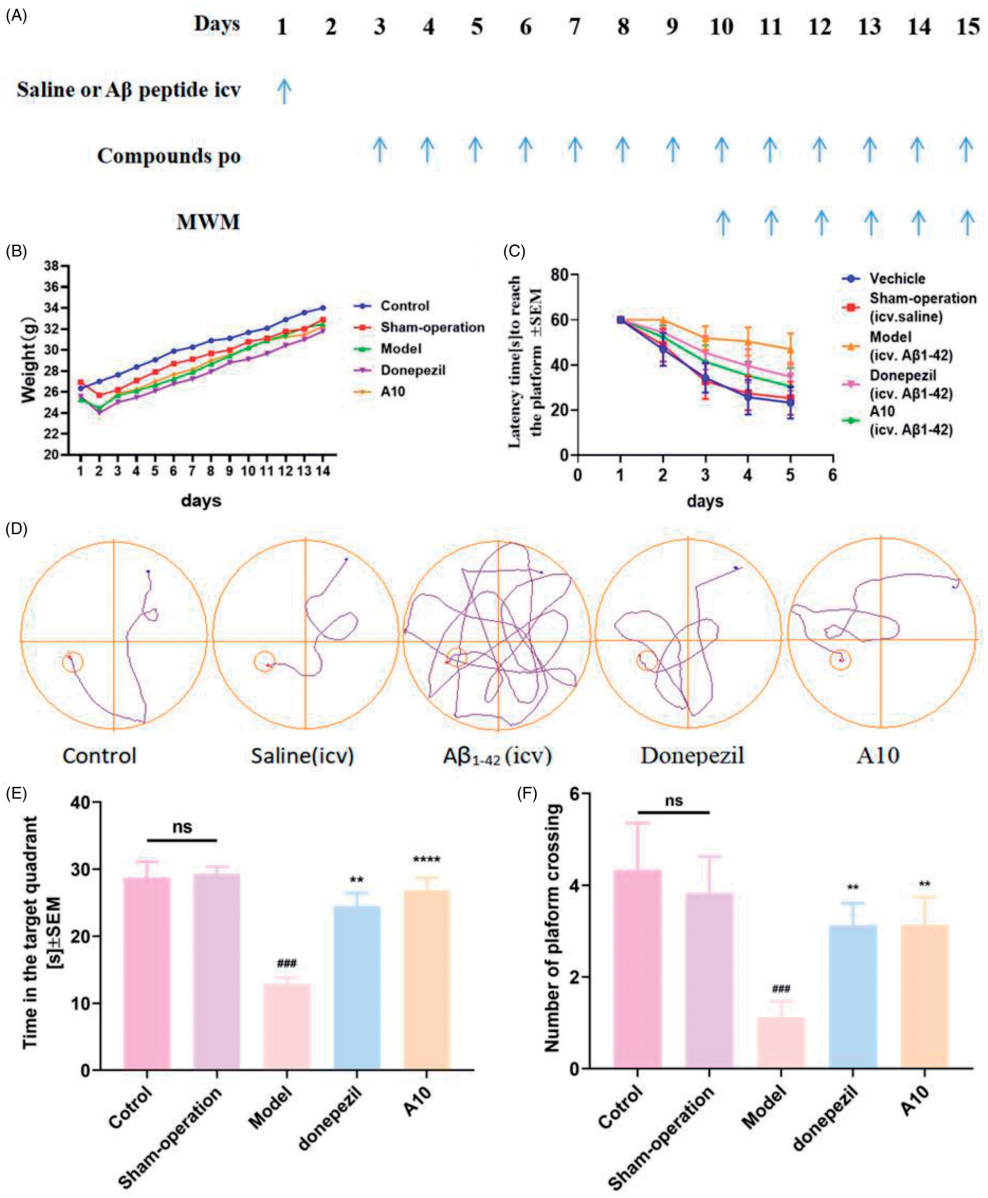
Effects of **A10** and donepezil on oligomeric A*β*_1–42_-induced damage experiments in the MWM task. (A) Protocol followed for *in vivo* experiments. Abbreviations: icv: intraventricular injection; po: orally; MWM: Morris water maze. (B) Daily body weight of mice in different groups during treatment. (C) Learning curves of the escape latencies during the acquisition phase of different groups. (D) Average footprints of mice in MWM on the last day of the study. (E) The time in the target quadrant during the acquisition phase of different groups. (F) The number of times the platform was crossed during the acquisition phase of different groups. Data are presented as mean ± SEM (*n* = 8; ^###^*p* < 0.001 vs. control group, **p < 0.01, ****p < 0.001 vs A*β*_1-42_ peptide model group).

To investigate the anti-AD effect of compound **A10**, we established a model of cognitive impairment induced by A*β*_1-42_ (intracerebroventricular (icv) injection)^58^^‒^^59^. Amyloid peptide (10 µg per mouse) was injected into the ventricles of 30 mice on day 1, while a sham group was set up, that is, only the same amount of saline was injected into the ventricles. Donepezil (15 mg/kg, as positive groups) and **A10** (10 mg/kg) were administered from Day 3 to Day 14 (po). The animal condition and body weight were examined daily during the administration period (Figure 9(B)). Compound **A10** did not affect the body weight gain, with no significant difference from the control group, showing good safety of compound **A10**. Behavioural experiments were performed from Day 10 to Day 14. Morris water maze (MWM) test was used. MWM was a spatial learning test for rodents, which relied on distal cues navigating from the starting point around the open swimming field to locate the underwater escape platform. MWM was mainly used to study the effects of shortening the time to reach the escape platform (i.e. escape time latency) on long-term memory^60^^,^^61^. MWM test included learning behaviour test on Days 10–14 and probe test on Day 15. As shown in Figure 9(C), intraventricular injection of normal saline did not affect the cognitive and learning ability of mice, and no difference from the blank group in terms of undifferentiated alternating behaviour, latency to reach the target and confusion. The learning ability and memory ability of the mice in the model group were significantly worse than those in the control group. As shown in Figure 9(D–F), compared with the model group, the donepezil group could significantly shorten the time to find the platform and increase the time to be on the platform. Compared with the donepezil group, **A10** shortened the latency, simplified the movement trajectory to the platform, improved the interaction ability, and the overall target quadrant preference (the number of crossing the platform and the swimming time in the target quadrant), indicating that both **A10** (10 mg/kg) and donepezil (15 mg/kg) significantly improved the A*β*_1-42_-induced cognitive dysfunction.

**Figure 10.**
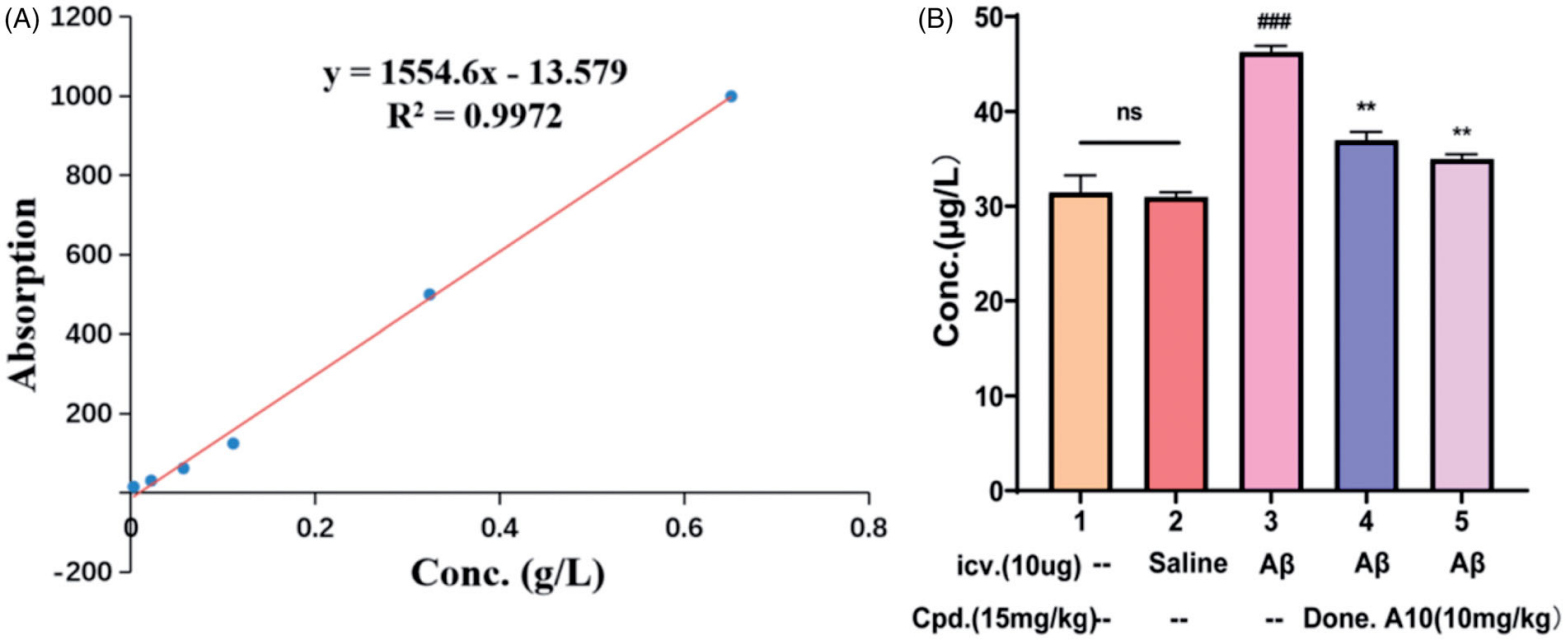
The A*β*_1 − 42_ total amount was quantified by using a mouse A*β*_1 − 42_ ELISA kit. (A) Standard curve; (B) A*β*_1 − 42_ total amount in mice brains of different groups. Brain tissue A*β*_1-42_ content was calculated according to linear regression equation, data are presented as mean ± SEM (*n* = 8; ^###^*p* < 0.001 vs. control group, **p < 0.01 vs. A*β*_1-42_ peptide model group).

At the end of the behavioural study, the mice were sacrificed, and the A*β*_1-42_ levels were measured with a mouse A*β*_1-42_ ELISA kit. As shown in Figure 10(A,B), the total levels of A*β*_1-42_ peptides in the icv A*β*_1-42_ group were significantly increased compared with the control or sham groups, indicating that the modelling was successful, and the A*β*_1-42_ peptides in the mice treated with donepezil or **A10** were significantly decreased (14.7 and 20.6%, respectively), consistent with the results of behavioural experiments, supporting that compound **A10** can further exert a neuroprotective effect on A*β*_1-42_ toxicity by reducing BuChE levels, thereby effectively improving the cognitive function of AD mice.

## Conclusion

A series of novel scaffolds of *α*-substituted δ-aryl-1,3-dienylsulphonyl fluorides were obtained from Knoevenagel-type reaction, Suzuki coupling and Sonogashira reaction. *In vitro* ChEs assay revealed that most of compounds exhibited selective BuChE inhibitory activity, except for compound **A18** (0.079 µM) as AChE inhibitor, compounds **A8** (0.107, 0.44 µM) and **A11** (0.15, 0.27 µM) as dual AChE and BuChE inhibitors. Amongst them, compounds **A1** (0.082 µM) and **A10** (0.021 µM) showed potent selective BuChE inhibition. SAR analysis for *δ*-aryl-*α*-Br-1,3-dienylsulfonyl fluorides showed: Effect of substituent of *δ*-aryl on BuChE activity, (i) *o*-position > *m*-position > *p*-position, (ii) ‒OCH_3_ >‒CH_3_ >‒Cl (‒Br); effect of α-substituent on BuChE activity, (i) α-Br > α-Cl or α-I, (ii) α-Br > α-aryl or α-alkynyl. Compound **A10** was identified as a highly selective BuChE inhibitor (IC_50_ = 21 nM for eqBuChE, 3.62 µM for hBuChE), which was nicely bound into hBuChE via π − alkyl interaction with Pro281 and hydrogen bond interaction with Lys248, Asn241, and Asn245. Kinetic studies showed that BuChE inhibition of compound **A10** was reversible, mixed-competitive (*K*_i_=29 nM). Compound **A10** had remarkable neuroprotective activity and benign BBB penetrating ability. *In vitro* and *in vivo* safety study showed that compound **A10** possessed good neural and hepatic safety and was tolerated up to a dose of 1.0 g/kg. In a subsequent *in vivo* behavioural study, treatment with compound **A10** improved the cognitive impairment caused by A*β*_1 − 42_ induction, significantly prevented the effects of A*β*_1 − 42_ toxicity, and almost restored the cognitive function. Moreover, the evaluation of the A*β*_1 − 42_ total amount confirmed its anti-amyloidogenic profile. However, as a selective BuChE inhibitor, compound **A10** displayed better cognitive improving and anti-amyloidogenic effects than the positive donepezil. Hence, compound **A10** has potential to be further developed as promising therapeutics for AD treatment.

The Supplementary data include: Synthesis of series **A**, **B**, **C** and **D**, and the copies of representative ^1^H and ^13 ^C NMR spectra.

## Supplementary Material

Supplemental Material
