# Supplementary material for: Structure–activity relationship, in vitro and in vivo evaluation of novel dienyl sulphonyl fluorides as selective BuChE inhibitors for the treatment of Alzheimer's disease
Source: J Enzyme Inhib Med Chem. 2021 Aug 23;36(1):1860–73. doi: 10.1080/14756366.2021.1959571 (PMC8386747; doi:10.1080/14756366.2021.1959571)
Supplement: Supplemental Material [file IENZ_A_1959571_SM7520.pdf]

Structure-activity relationship, *in vitro* and *in vivo* evaluation of novel dienyl sulfonyl fluorides as selective BuChE inhibitors for the treatment of Alzheimer's disease

Chengyao Wu[a,*], Guijuan Zhang[b,*], Zai-Wei Zhang[c], Xia Jiang[a], Ziwen Zhang[a], Huanhuan Li[a], Hua-Li Qin[c] and Wen-Jian Tang[a]

[a] School of Pharmacy, Anhui Province Key Laboratory of Major Autoimmune Diseases, Anhui Medical University, Hefei 230032, China

[b] Management Center of Anhui Continuing Education Network Park, Anhui Open University, Hefei 230002, China

[c] School of Chemistry, Chemical Engineering and Life Science, Wuhan University of Technology, Wuhan 430070, China

Table of content

1. Diagrams of molecular docking of **piperine** (**A**) and **piperine sulfonyl fluoride** (**C**) into target hBuChE (PDB: 1p0i) S2
2. Synthesis of series **A**, **B**, **C** and **D** S3
3. NMR spectra and HPLC spectra of series **A** S15
4. NMR spectra and HPLC spectra of series **B** S91
5. NMR spectra and HPLC spectra of series **C** S115
6. NMR spectra and HPLC spectra of series **D** S137

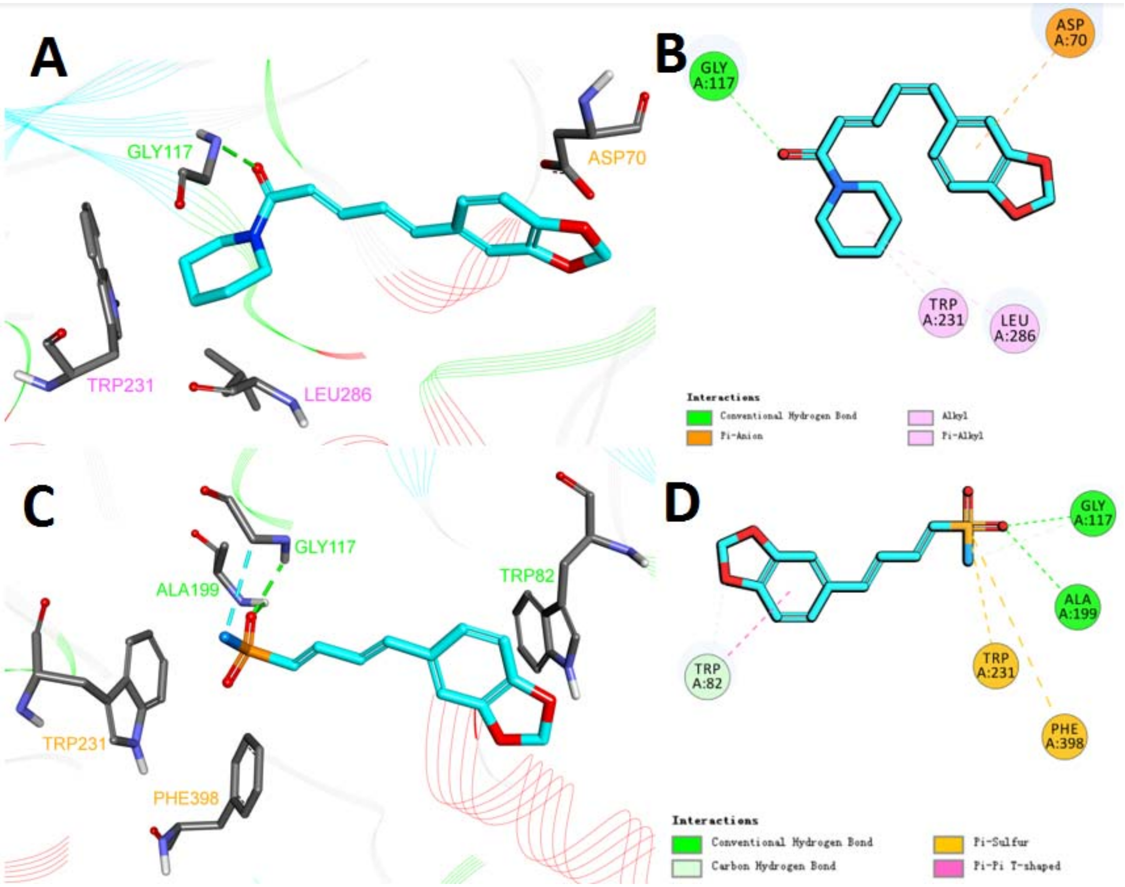

Figure S1. 3D diagram of **piperine** (**A**) and **piperine sulfonyl fluoride** (**C**) into hBuChE (PDB: 1p0i) performed respectively. Active site residues of hBuChE were presented as sticks with carbon atoms represented in light green (light blue for **piperine** and **piperine sulfonyl fluoride**). The green dashed lines represent hydrogen bonds, the light blue dashed lines represent halogen interaction bonds, the light pink dashed line represents π-alkyl interaction and dark pink dashed line represents π-π stacking interaction. 2D diagram of compounds **piperine** (**B**) and **piperine sulfonyl fluoride** (**D**) into hBuChE (PDB: 1p0i) performed respectively.

1. General procedure for the synthesis of series A1–A20, B1–B6 and C1–C6

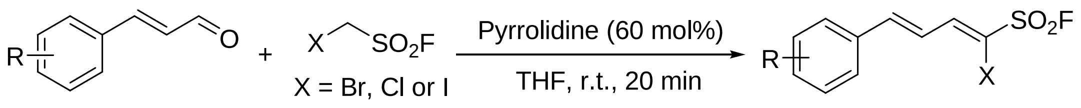

To an oven-dried reaction tube was added aldehyde (**1**, 1.0 mmol, 1 eq.), pyrrolidine (60 mol%, 50 μL) and THF (0.2 M, 5 mL). And the stirring was lasted for 5 min before the subsequent addition of halomethylsulfonyl fluoride (**2**, 3.0 mmol, 3 eq.). Then the resulting mixture was allowed to stir at room temperature for about 20 min, until the aldehyde was completely consumed monitored by TLC. The mixture was diluted with DCM (10 mL) and concentrated to dryness under reduced pressure. The residue was purified by column chromatography on silica gel (petroleum ether/ethyl acetate, 40:1, v/v) to give the desired products **A1–A20**, **B1–B6** and **C1–C6**, and the detailed synthesis process was reported in the literature[44].

*Compound **A1***. White solid, 278 mg, 96% yield; purity, 98.7%; mp: 76–78°C; ^{1}H NMR (500 MHz, $CDCl_3$) δ 7.92 (d, J = 10.6 Hz, 1H), 7.59-7.57 (m, 2H), 7.44-7.43 (m, 3H), 7.26 (d, J = 15.5 Hz, 1H), 7.01 (dd, J_1 = 15.6 Hz, J_2 = 10.7 Hz, 1H); ^{19}F NMR (471 MHz, $CDCl_3$) δ 53.1; ^{13}C NMR (126 MHz, $CDCl_3$) δ 148.1, 145.3, 134.9, 131.1, 129.3, 128.3, 122.0, 109.7 (d, J = 33.6 Hz); HRMS (EI) m/z: $[M]^+$ calcd for $C_{10}H_8BrFO_2S$, 289.9412; found 289.9417.

*Compound **A2***. Yellow solid, 265 mg, 86% yield; purity, 96.0%; mp: 80–82°C; ^{1}H NMR (500 MHz, $CDCl_3$) δ 7.90 (d, J = 10.7 Hz, 1H), 7.59-7.55 (m, 2H), 7.22 (d, J = 15.5 Hz 1H), 7.14-7.10 (m, 2H), 6.92 (dd, J_1 = 15.6 Hz, J_2 = 10.5 Hz, 1H); ^{19}F NMR (471 MHz, $CDCl_3$) δ 53.1, -107.9 (m, 1F); ^{13}C NMR (126 MHz, $CDCl_3$) δ 164.4 (d, J = 253.4 Hz), 146.6, 145.2, 131.2 (d, J = 2.7 Hz) ,130.3 (d, J = 8.2 Hz), 121.7, 116.5 (d, J = 22.7 Hz), 109.7 (d, J = 33.6 Hz); HRMS (EI) m/z: $[M]^+$ calcd. for $C_{10}H_7BrF_2O_2S$, 307.9318; found 307.9299.

*Compound **A3***. Yellow solid, 266 mg, 82% yield; purity, 95.1%; mp: 125–127°C; ^{1}H NMR (500 MHz, $CDCl_3$) δ 7.90 (d, J = 10.6 Hz, 1H), 7.51 (d, J = 8.5 Hz, 2H), 7.40 (d, J = 8.5 Hz, 2H), 7.20 (d, J = 15.6 Hz, 1H), 6.97 (dd, J_1 = 15.6 Hz, J_2 = 10.6 Hz, 1H); ^{19}F NMR (471 MHz, $CDCl_3$) δ 53.1; ^{13}C NMR (126 MHz, $CDCl_3$) δ 146.4,

144.9, 137.0, 133.4, 129.6, 129.4, 122.4, 110.3 (d, J = 33.6 Hz); HRMS (EI) m/z: $[M-H]^+$ calcd. for $C_{10}H_6BrClFO_2S$, 322.8950; found 322.8970.

Compound ***A4***. Yellow solid, 280 mg, 76% yield; mp: 146–148°C. ^{1}H NMR (500 MHz, $CDCl_3$) δ 7.90 (d, J = 10.5 Hz, 1H), 7.56 (d, J = 8.4 Hz, 2H), 7.43 (d, J = 8.4 Hz, 2H), 7.19 (d, J = 15.6 Hz, 1H), 6.99 (dd, J_1 = 15.5 Hz, J_2 = 10.5 Hz, 1H); ^{19}F NMR (471 MHz, $CDCl_3$) δ 53.1; ^{13}C NMR (126 MHz, $CDCl_3$) δ 146.4, 144.9 (d, J = 1.8 Hz), 133.8, 132.6, 129.6, 125.4, 122.5, 110.5 (d, J = 33.6 Hz); HRMS (EI) m/z: $[M]^+$ calcd for $C_{10}H_7Br_2FO_2S$, 367.8518; found 367.8512.

Compound ***A5***. Yellow solid, 283 mg, 93% yield; purity, 96.5%; mp: 109–110°C; ^{1}H NMR (500 MHz, $CDCl_3$) δ 7.90 (d, J = 10.7 Hz, 1H), 7.47 (d, J = 8.0 Hz, 2H), 7.24-7.21 (m, 3H), 6.96 (dd, J_1 = 15.6 Hz, J_2 = 10.7 Hz, 1H), 2.40 (s, 3H); ^{19}FNMR (471 MHz, $CDCl_3$) δ 53.2; ^{13}C NMR (126 MHz, $CDCl_3$) δ 148.2, 145.7(d, J = 2.7 Hz), 141.8, 132.3, 130.0, 128.4, 121.0, 108.8 (d, J = 33.6 Hz), 21.7; HRMS (EI) m/z: $[M]^+$ calcd for $C_{11}H_{10}BrFO_2S$, 303.9569; found 303.9561.

Compound ***A6***. Yellow solid, 301 mg, 94% yield; purity, 96.2%; mp: 93–95°C; ^{1}H NMR (500 MHz, $CDCl_3$) δ 7.89 (d, J = 10.7 Hz, 1H), 7.53 (d, J = 8.7 Hz, 2H), 7.20 (d, J = 15.6 Hz, 1H), 6.94 (d, J = 8.7 Hz, 2H), 6.87 (dd, J_1 = 15.5 Hz, J_2 = 10.7 Hz, 1H), 3.87 (s, 3H); ^{19}F NMR (471 MHz, $CDCl_3$) δ 53.4; ^{13}C NMR (126 MHz, $CDCl_3$) δ 162.2, 148.0, 145.9, 130.2, 127.8, 119.7, 114.8, 107.7 (d, J = 33.6 Hz), 55.6; HRMS (ESI) m/z: $[M + H]^+$ calcd. for $C_{11}H_{11}BrFO_3S$, 320.9591; found 320.9588.

Compound ***A7***. Yellow solid, 296 mg, 81% yield; purity, 97.8%; mp: 150–152°C; ^{1}H NMR (500 MHz, $CDCl_3$) δ 7.94 (d, J = 10.5 Hz, 1H), 7.69-7.62 (m, 6H), 7.50-7.47 (m, 2H), 7.42-7.39 (m, 1H), 7.29 (d, J_1 = 15.4 Hz, 1H), 7.04 (dd, J_1 = 15.5 Hz, J_2 = 10.7 Hz, 1H); ^{19}F NMR (471 MHz, $CDCl_3$) δ 53.2; ^{13}C NMR (126 MHz, $CDCl_3$) δ 147.6, 145.4 (d, J = 1.9 Hz), 143.8, 140.0, 133.8, 129.1, 128.9, 128.3, 127.9, 127.2, 121.8, 109.4 (d, J = 32.7 Hz); HRMS (EI) m/z: $[M]^+$ calcd. for $C_{16}H_{12}BrFO_2S$, 365.9725; found 365.9707.

Compound ***A8***. Yellow solid, 309 mg, 84% yield; purity, 97.9%; mp: 112–114°C; ^{1}H NMR (500 MHz, $CDCl_3$) δ 8.09 (d, J = 8.2 Hz, 1H), 7.98 (d, J = 10.5 Hz, 1H), 7.81-7.70 (m, 3H), 7.60 (t, J = 7.6 Hz 1H), 6.94 (dd, J_1 = 15.4 Hz, J_2 = 10.5 Hz 1H);

^{19}F NMR (471 MHz, $CDCl_3$) δ 52.9; ^{13}C NMR (126 MHz, $CDCl_3$) δ 148.2, 144.2 (d, J = 2.7 Hz), 142.4, 133.9, 130.9, 130.8, 129.1, 126.4, 125.4, 112.9 (d, J = 33.6 Hz); HRMS (EI) m/z: $[M]^+$ calcd for $C_{10}H_7Br_2FO_2S$, 367.8518; found 367.8511.

Compound ***A9***. Yellow solid, 243 mg, 80% yield; purity, 98.2%; mp: 103–105°C; ^1H NMR (500 MHz, $CDCl_3$) δ 7.96 (d, J = 10.6 Hz, 1H), 7.66 (d, J = 7.6 Hz, 1H), 7.54 (d, J = 15.4 Hz, 1H) ,7.33 (td, J_1 = 7.4 Hz, J_2 = 1.1 Hz, 1H), 7.29-7.23 (m, 2H), 6.95 (dd, J_1 = 15.4 Hz, J_2 = 10.7 Hz, 1H), 2.44 (s, 3H); ^{19}F NMR (471 MHz, $CDCl_3$) δ 53.1; ^{13}C NMR (126 MHz, $CDCl_3$) δ 145.6. 145.6, 137.9, 133.8, 131.2, 130.9, 126.8, 126.5, 122.9, 109.5 (d, J = 32.7 Hz), 19.9; HRMS (EI) m/z: $[M]^+$ calcd for $C_{11}H_{10}BrFO_2S$, 303.9569; found 303.9561.

Compound ***A10***. Yellow solid, 269 mg, 84% yield; purity, 98.6%; mp: 78–80°C; ^1H NMR (500 MHz, $CDCl_3$) δ 7.93 (d, J = 10.7 Hz, 1H), 7.63-7.58 (m, 2H), 7.40 (t, J = 8.1 Hz, 1H), 7.07 (dd, J_1 = 15.6 Hz, J_2 = 10.7 Hz, 1H), 7.01 (t, J = 7.6 Hz, 1H), 6.94 (d, J = 8.4 Hz, 1H), 3.92 (s, 3H); ^{19}F NMR (471 MHz, $CDCl_3$) δ 53.3; ^{13}C NMR (126 MHz, $CDCl_3$) δ 158.5, 146.5, 143.6, 132.5, 128.6, 123.9, 122.3, 121.1, 111.5, 108.3 (d, J = 32.7 Hz), 55.8; HRMS (EI) m/z: $[M]^+$ calcd. for $C_{11}H_{10}BrFO_3S$, 319.9518; found 319.9498.

Compound ***A11***. Yellow solid, 234 mg, 70% yield; purity, 95.9%; mp: 109–111°C; ^1H NMR (500 MHz, $CDCl_3$) δ 8.08 (d, J = 8.3 Hz, 1H), 7.98 (d, J = 10.5 Hz, 1H), 7.81-7.70 (m, 3H), 7.59 (t, J = 7.9 Hz, 1H), 6.94 (dd, J_1 = 15.5 Hz, J_2 = 10.6 Hz, 1H); ^{19}F NMR (471 MHz, $CDCl_3$) δ 52.9; ^{13}C NMR (126 MHz, $CDCl_3$) δ 148.2, 144.2 (d, J = 2.7 Hz), 142.4, 133.9, 130.9, 130.8, 129.1, 126.4, 125.4, 112.9 (d, J = 33.6 Hz); HRMS (EI) m/z: $[M]^+$ calcd. for $C_{10}H_7BrFNO_4S$, 334.9263; found 334.9245.

Compound ***A12***. Yellow solid, 0.5mmol scale, 150 mg, 88% yield; purity, 96.0%; mp: 129–131°C; ^1H NMR (500 MHz, $CDCl_3$) δ 7.97-7.93 (m, 2H), 7.90-7.85 (m, 3H), 7.72 (d, J = 8.5 Hz, 1H), 7.58-7.53 (m, 2H), 7.38 (d, J = 15.4 Hz, 1H), 7.10 (dd, J_1 = 15.5 Hz, J_2 = 10.6 Hz, 1H); ^{19}F NMR (471 MHz, $CDCl_3$) δ 53.3; ^{13}C NMR (126 MHz, $CDCl_3$) δ 148.1, 145.4, 134.6, 133.4, 132.4, 130.5, 129.1, 128.8, 128.0, 127.9, 127.2, 123.4, 122.0, 109.5 (d, J = 32.7 Hz); HRMS (EI) m/z: $[M]^+$ calcd for $C_{14}H_{10}BrFO_2S$, 339.9569; found 339.9565.

Compound ***A13***. Brown solid, 237 mg, 80% yield; mp: 89–91°C; ^{1}H NMR (500 MHz, $CDCl_3$) δ 7.85 (d, J = 10.6 Hz, 1H), 7.48 (d, J = 5.0 Hz, 1H), 7.36 (d, J = 15.2 Hz, 1H), 7.31 (d, J = 3.5 Hz, 1H), 7.10 (dd, J_1 = 4.9 Hz, J_2 = 3.7 Hz, 1H), 6.76 (dd, J_1 = 15.3 Hz, J_2 = 10.7 Hz, 1H); ^{19}F NMR (471 MHz, $CDCl_3$) δ 53.4; ^{13}C NMR (126 MHz, $CDCl_3$) δ 145.0 (d, J = 2.7 Hz), 140.4, 140.0, 131.7, 130.0, 128.7, 121.0, 108.7 (d, J = 33.6 Hz); HRMS (EI) m/z: $[M]^+$ calcd for $C_8H_6BrFO_2S_2$, 295.8977; found 295.8981.

Compound ***A14***. Yellow solid, 233 mg, 72% yield; purity, 97.1%; mp: 65–67°C; ^{1}H NMR (500 MHz, $CDCl_3$) δ 7.90 (d, J = 10.5 Hz, 1H), 7.55-7.54 (m, 1H), 7.44 (d, J = 7.3 Hz, 1H), 7.41-7.35 (m, 2H), 7.18 (d, J = 15.7 Hz, 1H), 6.99 (dd, J_1 = 15.6 Hz, J_2 = 10.5 Hz, 1H); ^{19}F NMR (471 MHz, $CDCl_3$) δ 53.0; ^{13}C NMR (126 MHz, $CDCl_3$) δ 146.0, 144.7 (d, J = 1.8 Hz), 136.7, 135.4, 130.8, 130.5, 127.9, 126.5, 123.2, 111.1 (d, J = 32.7 Hz); HRMS (EI) m/z: $[M]^+$ calcd for $C_{10}H_7BrClFO_2S$, 323.9023; found 323.9029.

Compound ***A15***. Yellow solid, 280 mg, 92% yield; purity, 95.8%; mp: 69–71°C; ^{1}H NMR (500 MHz, $CDCl_3$) δ 7.91 (d, J = 10.7 Hz, 1H), 7.37 (m, 2H), 7.34-7.30 (m, 1H), 7.26-7.21 (m, 2H), 6.99 (dd, J_1 = 15.6 Hz, J_2 = 10.7 Hz, 1H), 2.41 (s, 3H); ^{19}F NMR (471 MHz, $CDCl_3$) δ 53.1; ^{13}C NMR (126 MHz, $CDCl_3$) δ 148.4, 145.5 (d, J = 1.8 Hz), 139.0, 134.9, 132.0, 129.2, 128.9, 125.6, 121.7, 109.3 (d, J = 32.7 Hz), 21.4; HRMS (EI) m/z: $[M]^+$ calcd. for $C_{11}H_{10}BrFO_2S$, 303.9569; found 303.9551.

Compound ***A16***. Yellow solid, 298 mg, 93% yield; mp: 54–56°C; ^{1}H NMR (500 MHz, $CDCl_3$) δ 7.91 (d, J = 10.6 Hz, 1H), 7.34 (t, J = 7.9 Hz, 1H), 7.22 (d, J = 15.5 Hz, 1H) , 7.17 (d, J = 7.6 Hz, 1H), 7.07 (m, 1H), 7.00-6.95 (m, 2H), 3.86 (s, 3H); ^{19}F NMR (471 MHz, $CDCl_3$) δ 53.1; ^{13}C NMR (126 MHz, $CDCl_3$) δ 160.2, 148.0, 145.3 (d, J = 1.8 Hz), 136.3, 130.3, 122.2, 121.0, 116.7, 113.5, 109.8 (d, J = 33.6 Hz), 55.5; HRMS (EI) m/z: $[M]^+$ calcd for $C_{11}H_{10}BrFO_3S$, 319.9518; found 319.9513.

Compound ***A17***. Yellow solid, 247 mg, 69% yield; purity, 97.3%; mp: 71–73°C; ^{1}H NMR (500 MHz, $CDCl_3$) δ 7.91 (d, J = 10.5 Hz, 1H), 7.76 (s, 1H), 7.72 (d, J = 7.7 Hz, 1H) , 7.65 (d, J = 7.7 Hz, 1H), 7.54 (t, J = 7.8 Hz, 1H), 7.24 (s, 1H), 7.03 (dd, J_1 = 15.5 Hz, J_2 = 10.5 Hz, 1H); ^{19}F NMR (471 MHz, $CDCl_3$) δ 52.9, -62.9; ^{13}C NMR

(126 MHz, $CDCl_3$) δ 145.8, 144.5, 135.6, 131.9 (q, *J* = 32.7 Hz), 131.2, 129.9, 127.3 (q, *J* = 3.6 Hz), 124.8 (q, *J* = 3.6 Hz), 123.8 (q, *J* = 273.4 Hz), 123.6, 111.6 (d, *J* = 33.6 Hz); HRMS (EI) m/z: $[M]^+$ calcd. for $C_{11}H_7BrF_4O_2S$, 357.9286; found 357.9267.

Compound ***A18***. White solid, 208 mg, 64% yield; purity, 98.7%; mp: 149–151°C; ^1H NMR (500 MHz, $CDCl_3$) δ 8.53 (d, *J* = 2.5 Hz, 1H), 7.92 (d, *J* = 10.5 Hz, 1H), 7.88 (dd, J_1 = 8.3 Hz, J_2 = 2.5 Hz, 1H), 7.41 (d, *J* = 8.4 Hz, 1H), 7.22 (d, *J* = 15.7 Hz, 1H), 7.03 (dd, J_1 = 15.7 Hz, J_2 = 10.5 Hz, 1H); ^{19}F NMR (471 MHz, $CDCl_3$) δ 53.0; ^{13}C NMR (126 MHz, $CDCl_3$) δ 153.3, 149.7, 144.0 (d, *J* = 2.7 Hz), 142.1, 136.5, 129.7, 125.0, 124.3, 112.2 (d, *J* = 33.6 Hz); HRMS (EI) m/z: $[M]^+$ calcd. for $C_9H_6BrClFNO_2S$, 324.8975; found 324.8951.

Compound ***A19***. Yellow solid, 0.5 mmol scale, 186 mg, 77% yield; mp: 154–156°C; ^1H NMR (500 MHz, $CDCl_3$) δ 8.02 (d, *J* = 8.1 Hz, 1H), 7.92-7.90 (m, 2H), 7.83 (d, *J* = 8.4 Hz, 2H), 7.79 (d, *J* = 7.8 Hz, 1H), 7.44-7.27 (m, 5H), 7.04 (dd, J_1 = 15.6 Hz, J_2 = 10.7 Hz, 1H), 2.37 (s, 3H); ^{19}F NMR (471 MHz, $CDCl_3$) δ 53.5; ^{13}C NMR (126 MHz, $CDCl_3$) δ 146.0, 145.7 (d, *J* = 1.8 Hz), 138.8, 135.7, 134.7, 130.3, 128.8, 127.8, 127.2, 126.0, 124.6, 122.2, 120.6, 119.0, 114.1, 108.6 (d, *J* = 32.7 Hz), 21.8; HRMS (EI) m/z: $[M]^+$ calcd for $C_{19}H_{15}BrFNO_4S_2$, 482.9610; found 482.9618.

Compound ***A20***. Yellow solid, 0.5 mmol scale, 142 mg, 90% yield; purity, 99.5%; mp: 78–80°C; ^1H NMR (500 MHz, $CDCl_3$) δ 7.83 (d, *J* = 10.9 Hz, 1H), 7.50 (d, *J* = 7.0 Hz, 2H), 7.41-7.34 (m, 3H), 7.05 (dd, J_1 = 14.1 Hz, J_2 = 9.6 Hz, 1H), 7.01-6.92 (m, 2H), 6.56 (dd, J_1 = 14.3 Hz, J_2 = 10.9 Hz, 1H); ^{19}F NMR (471 MHz, $CDCl_3$) δ 53.4; ^{13}C NMR (126 MHz, $CDCl_3$) δ 148.1, 145.1 (d, *J* = 1.8 Hz), 141.6, 135.9, 129.7, 129.1, 127.6, 127.3, 125.5, 108.9 (d, *J* = 33.6 Hz); HRMS (EI) m/z: $[M]^+$ calcd for $C_{12}H_{10}BrFO_2S$, 315.9569; found 315.9574.

Compound ***B1***. Yellow solid, 0.5 mmol scale, 98 mg, 80% yield; purity, 97.3%; mp: 70–72°C; ^1H NMR (500 MHz, $CDCl_3$) δ 7.71 (d, *J* = 10.7 Hz, 1H), 7.58-7.56 (m, 2H), 7.44-7.42 (m, 3H), 7.20 (d, *J* = 15.7 Hz, 1H), 7.05 (dd, J_1 = 15.8 Hz, J_2 = 10.9 Hz, 1H); ^{19}F NMR (471 MHz, $CDCl_3$) δ 52.6; ^{13}C NMR (126 MHz, $CDCl_3$) δ 147.4, 141.6 (d, *J* = 1.8 Hz), 134.9, 131.0, 129.3, 128.3, 121.1 (d, *J* = 33.6 Hz), 119.8; HRMS (EI) m/z: $[M]^+$ calcd for $C_{10}H_8ClFO_2S$, 245.9918; found 245.9914.

Compound ***B2***. Yellow solid, 0.5 mmol scale, 107 mg, 81% yield; purity, 96.0%; mp: 80–82°C; ^{1}H NMR (500 MHz, $CDCl_3$) δ 7.69 (d, J = 10.7 Hz, 1H), 7.57-7.55 (m, 2H), 7.16 (d, J = 15.7 Hz, 1H), 7.14-7.10 (m, 2H), 6.97 (dd, J_1 = 15.6 Hz, J_2 = 10.8 Hz, 1H); ^{19}F NMR (471 MHz, $CDCl_3$) δ 52.5, -108.1 (m, 1F); ^{13}C NMR (126 MHz, $CDCl_3$) δ 164.3 (d, J = 253.5 Hz), 145.9, 141.4 (d, J = 1.8 Hz), 131.2 (d, J = 2.7 Hz), 130.2 (d, J = 8.2 Hz), 121.2 (d, J = 31.8 Hz), 119.6 (d, J = 2.7 Hz), 116.5 (d, J = 21.8 Hz); HRMS (EI) m/z: $[M-H]^+$ calcd. for $C_{10}H_6ClF_2O_2S$, 262.9751; found 262.9760.

Compound ***B3***. Yellow solid, 0.5 mmol scale, 108 mg, 83% yield; purity, 97.8%; mp: 104–106°C; ^{1}H NMR (500 MHz, $CDCl_3$) δ 7.69 (d, J = 10.7 Hz, 1H), 7.46 (d, J = 8.1 Hz, 2H), 7.24 (d, J = 8.1 Hz, 2H), 7.17 (d, J = 15.6 Hz, 1H), 7.00 (dd, J_1 = 15.5 Hz, J_2 = 10.7 Hz, 1H), 2.40 (s, 3H); ^{19}F NMR (471 MHz, $CDCl_3$) δ 52.7; ^{13}C NMR (126 MHz, $CDCl_3$) δ 147.5, 141.9 (d, J = 1.8 Hz), 141.7, 132.2, 130.0, 128.3, 120.3 (d, J = 33.6 Hz), 118.8, 21.7; HRMS (EI) m/z: $[M]^+$ calcd for $C_{11}H_{10}ClFO_2S$, 260.0074; found 260.0079.

Compound ***B4***. Yellow solid, 0.5 mmol scale, 126 mg, 91% yield; purity, 99.8%; mp: 62–64°C; ^{1}H NMR (500 MHz, $CDCl_3$) δ 7.68 (d, J = 10.9 Hz, 1H), 7.52 (d, J = 8.7 Hz, 2H), 7.14 (d, J = 15.6 Hz, 1H), 6.95-6.88 (m, 3H), 3.86 (s, 3H); ^{19}F NMR (471 MHz, $CDCl_3$) δ 52.8; ^{13}C NMR (126 MHz, $CDCl_3$) δ 162.1, 147.3, 142.2 (d, J = 2.7 Hz), 130.1, 127.8, 119.3 (d, J = 32.7 Hz), 117.5, 114.8, 55.6; HRMS (EI) m/z: $[M-H]^+$ calcd. for $C_{11}H_9ClFO_3S$, 274.9950; found 274.9962.

Compound ***B5***. Yellow solid, 0.5 mmol scale, 123 mg, 89% yield; purity, 96.4%; mp: 62–64°C; ^{1}H NMR (500 MHz, $CDCl_3$) δ 7.72 (d, J = 10.9 Hz, 1H), 7.59-7.54 (m, 2H), 7.41-7.38 (m, 1H), 7.11 (dd, J_1 = 15.6 Hz, J_2 = 10.8 Hz, 1H), 7.01 (t, J = 7.5 Hz, 1H), 6.94 (d, J = 8.2 Hz, 1H), 3.91 (s, 3H); ^{19}F NMR (471 MHz, $CDCl_3$) δ 52.7; ^{13}C NMR (126 MHz, $CDCl_3$) δ 158.5, 142.9, 142.7 (d, J = 2.7 Hz), 132.4, 128.6, 123.9, 121.1, 120.2, 119.9 (d, J = 32.7 Hz), 111.5, 55.7; HRMS (EI) m/z: $[M-H]^+$ calcd. for $C_{11}H_9ClFO_3S$, 274.9950; found 274.9961.

Compound ***B6***. Yellow solid, 0.5 mmol scale, 88 mg, 56% yield; purity, 98.1%; mp: 72–75°C; ^{1}H NMR (500 MHz, $CDCl_3$) δ 7.78 (s, 1H), 7.74 (d, J = 8.1 Hz, 1H), 7.71 (d, J = 10.6 Hz, 1H), 7.67 (d, J = 7.7 Hz, 1H), 7.57 (t, J = 7.8 Hz, 1H), 7.22 (d, J =

15.7, 1H), 7.11 (dd, J_1 = 15.6 Hz, J_2 = 10.6 Hz, 1H); ^{19}F NMR (471 MHz, $CDCl_3$) δ 52.4, -62.9; ^{13}C NMR (126 MHz, $CDCl_3$) δ 145.1, 140.7 (d, J = 2.7 Hz), 135.7, 131.9 (q, J = 32.7 Hz), 131.1, 129.9, 127.2 (q, J = 3.6 Hz), 124.7 (q, J = 3.6 Hz), 123.8 (q, J = 273.4 Hz), 123.0, 121.5; HRMS (EI) m/z: $[M]^+$ calcd for $C_{11}H_7ClF_4O_2S$, 313.9791; found 313.9798.

Compound ***C1***. Yellow solid, 0.5 mmol scale, 127 mg, 75% yield;purity, 96.5%; mp: 103–105°C; ^{1}H NMR (500 MHz, $CDCl_3$) δ 7.88 (d, J = 10.3 Hz, 1H), 7.59-7.57 (m, 2H), 7.45-7.43 (m, 3H), 7.31 (d, J = 15.4 Hz, 1H), 6.89 (dd, J_1 = 15.5 Hz, J_2 = 10.5 Hz, 1H); ^{19}F NMR (471 MHz, $CDCl_3$) δ 53.8; ^{13}C NMR (126 MHz, $CDCl_3$) δ 151.8 (d, J = 1.9 Hz), 148.7, 134.9, 131.2, 129.3, 128.4, 125.9, 82.5 (d, J = 30.9 Hz); HRMS (EI) m/z: $[M]^+$ calcd for $C_{10}H_8FIO_2S$, 337.9274; found 337.9279.

Compound ***C2***. Yellow solid, 0.5 mmol scale, 119 mg, 67% yield; purity, 96.6%; mp 84–86°C; ^{1}H NMR (500 MHz, $CDCl_3$) δ 7.86 (d, J = 10.6 Hz, 1H), 7.59-7.56 (m, 2H), 7.27 (d, J = 15.6 Hz, 1H), 7.14-7.11 (m, 2H), 6.81 (dd, J_1 = 15.4 Hz, J_2 = 10.4 Hz, 1H); ^{19}F NMR (471 MHz, $CDCl_3$) δ 53.8, -107.8 (m, 1F); ^{13}C NMR (126 MHz, $CDCl_3$) δ 164.4 (d, J = 253.4 Hz), 151.6 (d, J = 1.8 Hz), 147.2, 131.2 (d, J = 3.6 Hz), 130.3 (d, J = 8.2 Hz), 125.6, 116.5 (d, J = 22.7 Hz), 82.6 (dd, J_1 = 30.9 Hz, J_2 = 1.8 Hz); HRMS (EI) m/z: $[M]^+$ calcd for $C_{10}H_7F_2IO_2S$, 355.9179; found 355.9173.

Compound ***C3***. White solid, 0.5 mmol scale, 132 mg, 71% yield; mp: 130–132°C; ^{1}H NMR (500 MHz, $CDCl_3$) δ 7.86 (d, J = 10.4 Hz, 1H), 7.51 (d, J = 8.6 Hz, 2H), 7.40 (d, J = 8.5 Hz, 2H), 7.25 (d, J = 15.4 Hz, 1H), 6.85 (dd, J_1 = 15.4 Hz, J_2 = 10.4 Hz, 1H); ^{19}F NMR (471 MHz, $CDCl_3$) δ 53.8; ^{13}C NMR (126 MHz, $CDCl_3$) δ 151.4 (d, J = 2.7 Hz), 147.0, 137.1, 133.4, 129.6, 129.5, 126.4, 83.3 (d, J = 30.9 Hz); HRMS (EI) m/z: $[M]^+$ calcd for $C_{10}H_7ClFIO_2S$, 371.8884; found 371.8887.

Compound ***C4***. Yellow solid, 0.5 mmol scale, 116 mg, 56% yield; mp: 132–134°C; ^{1}H NMR (500 MHz, $CDCl_3$) δ 7.86 (d, J = 10.4 Hz, 1H), 7.56 (dt, J_1 = 8.4 Hz, J_2 = 2.5 Hz, 2H), 7.43 (dt, J_1 = 8.4 Hz, J_2 = 2.3 Hz, 2H), 7.23 (d, J = 15.6 Hz, 1H), 6.87 (dd, J_1 = 15.5 Hz, J_2 = 10.5 Hz, 1H); ^{19}F NMR (471 MHz, $CDCl_3$) δ 53.7; ^{13}C NMR (126 MHz, $CDCl_3$) δ 151.4 (d, J = 2.7 Hz), 147.1, 133.8, 132.6, 129.6, 126.5, 125.5, 83.4 (d, J = 30.8 Hz); HRMS (EI) m/z: $[M]^+$ calcd for $C_{10}H_7BrFIO_2S$, 415.8379;

found 415.8376.

Compound ***C5***. Yellow solid, 0.5 mmol scale, 142 mg, 77% yield; purity, 96.3%; mp: 79–81°C; ^{1}H NMR (500 MHz, $CDCl_3$) δ 7.87 (d, *J* = 10.4 Hz, 1H), 7.65 (d, *J* = 15.5 Hz, 1H), 7.59 (dd, J_1 = 7.8 Hz, J_2 = 1.4 Hz, 1H), 7.40 (td, J_1 = 8.7 Hz, J_2 = 1.6 Hz, 1H), 7.03-6.94 (m, 3H), 3.92 (s, 3H); ^{19}F NMR (471 MHz, $CDCl_3$) δ 54.0; ^{13}C NMR (126 MHz, $CDCl_3$) δ 158.5, 152.9 (d, *J* = 1.8 Hz), 144.3, 132.5, 128.8, 126.5, 123.9, 121.1, 111.5, 81.0 (d, *J* = 30.9 Hz), 55.8; HRMS (EI) m/z: $[M]^+$ calcd for $C_{11}H_{10}FIO_3S$; 367.9379, found 367.9373.

Compound ***C6***. White solid, 0.5 mmol scale, 130 mg, 64% yield; purity, 98.4%; mp: 78–80°C; 1H NMR (500 MHz, $CDCl_3$) δ 7.89 (d, *J* = 10.4 Hz, 1H), 7.79 (s, 1H), 7.76 (d, *J* = 7.9 Hz, 1H), 7.68 (d, *J* = 7.8 Hz, 1H), 7.57 (t, *J* = 7.8 Hz, 1H), 7.32 (d, *J* = 15.6 Hz, 1H), 6.94 (dd, J_1 = 15.4 Hz, J_2 = 10.4 Hz, 1H); ^{19}F NMR (471 MHz, $CDCl_3$) δ 53.6, -62.9; ^{13}C NMR (126 MHz, $CDCl_3$) δ 151.0 (d, *J* = 1.8 Hz), 146.4, 135.6, 131.9 (q, *J* = 32.7 Hz), 131.2, 129.9, 127.6, 127.3 (q, *J* = 3.7 Hz), 124.8 (q, *J* = 3.6 Hz), 123.8 (q, *J* = 273.4 Hz), 84.7 (d, *J* = 31.8 Hz); HRMS (EI) m/z: $[M]^+$ calcd for $C_{11}H_7F_4IO_2S$, 405.9148; found 405.9140.

2. General procedure for the synthesis of series D1–D7

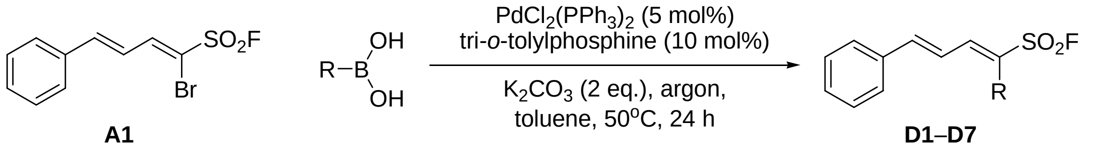

An oven-dried reaction tube was charged with **A1** (0.5 mmol, 145 mg 1 eq.), phenylboronic acid (1.5 eq.), tri-*o*-tolylphosphine (10 mol%), $PdCl_2(PPh_3)_2$ (5 mol%), K_2CO_3 (2 eq.) and dry toluene (2.5 mL) under nitrogen atmosphere before reacting at 50°C for 24 h. Then reaction mixture was concentrated to dryness and the residue was purified through silica gel chromatography using petroleum ether / ethyl acetate = 40:1 (v/v) as eluents to give the desired product **D**, and the detailed synthesis process was reported in the literature[44].

Compound ***D1***. Yellow solid, 114 mg, 72% yield; purity, 98.3%; mp: 96–98°C; ^{1}H NMR (500 MHz, $CDCl_3$) δ 7.71 (d, *J* = 11.3 Hz, 1H), 7.41-7.37 (m, 4H), 7.35-7.33 (m,

5H), 7.14 (d, J = 15.5 Hz, 1H), 6.71 (dd, J_1 = 15.5 Hz, J_2 = 11.1 Hz, 1H), 2.75 (q, J = 7.6 Hz, 2H), 1.32 (t, J = 7.6 Hz, 3H); ^{19}F NMR (471 MHz, $CDCl_3$) δ 56.5; ^{13}C NMR (126 MHz, $CDCl_3$) δ 146.6, 145.5, 143.7 (d, J = 1.8 Hz), 135.3, 133.2 (d, J = 22.7 Hz), 130.7, 130.3, 129.0, 128.7, 128.0, 126.3, 121.8, 28.9, 15.3; HRMS (EI) m/z: $[M-H]^+$ calcd. for $C_{18}H_{16}FO_2S$, 315.0861; found 315.0870.

Compound ***D2***. Yellow solid, 114 mg, 68% yield; purity, 96.8%; mp: 131–133°C; ^{1}H NMR (500 MHz, $CDCl_3$) δ 7.71 (d, J = 11.3 Hz, 1H), 7.41-7.34 (m, 9H), 7.15 (d, J = 15.5 Hz, 1H), 6.70 (dd, J_1 = 15.5 Hz, J_2 = 11.3 Hz, 1H), 2.55 (s, 3H); ^{19}F NMR (471 MHz, $CDCl_3$) δ 56.7; ^{13}C NMR (126 MHz, $CDCl_3$) δ 145.9, 143.9 (d, J = 2.7 Hz), 141.9, 135.2, 132.5 (d, J = 22.7 Hz), 131.1, 130.4, 129.1, 128.0, 126.2, 125.2, 121.6, 15.2; HRMS (EI) m/z: $[M-H]^+$ calcd. for $C_{17}H_{14}FO_2S_2$, 333.0425; found 333.0429.

Compound ***D3***. Yellow solid, 100 mg, 63% yield; purity, 97.1%; mp: 86–88°C; ^{1}H NMR (500 MHz, $CDCl_3$) δ 7.69 (d, J = 11.3 Hz, 1H), 7.41-7.38 (m, 4H), 7.35-7.34 (m, 3H), 7.13 (d, J = 15.6 Hz, 1H), 7.03 (d, J = 8.7 Hz, 2H), 6.70 (dd, J_1 = 15.6 Hz, J_2 = 11.1 Hz, 1H), 3.89 (s, 3H); ^{19}F NMR (471 MHz, $CDCl_3$) δ 56.0; ^{13}C NMR (126 MHz, $CDCl_3$) δ 161.0, 145.4, 143.5 (d, J = 1.8 Hz), 135.3, 132.9 (d, J = 21.8 Hz), 132.2, 130.2, 129.0, 127.9, 121.8, 121.0, 114.6, 55.5; HRMS (EI) m/z: $[M-H]^+$ calcd. for $C_{17}H_{14}FO_3S$, 317.0653; found 317.0658.

Compound ***D4***. Yellow solid, 124 mg, 65% yield; purity, 98.2%; mp: 91–93°C; ^{1}H NMR (500 MHz, $CDCl_3$) δ 7.72 (d, J = 11.1 Hz, 1H), 7.44-7.41 (m, 6H), 7.37-7.36 (m, 3H), 7.21 (t, J = 7.4 Hz, 1H), 7.17-7.13 (m, 3H), 7.10 (d, J = 8.6 Hz, 2H), 6.72 (dd, J_1 = 15.6 Hz, J_2 = 11.3 Hz, 1H); ^{19}F NMR (471 MHz, $CDCl_3$) δ 56.4; ^{13}C NMR (126 MHz, $CDCl_3$) δ 159.5, 155.9, 145.8, 143.9 (d, J = 1.8 Hz), 135.2, 132.5(d, J = 22.7 Hz), 132.5, 130.4, 130.2, 129.1, 128.0, 124.5, 123.1, 121.6, 120.2, 118.3; HRMS (EI) m/z: $[M-H]^+$ calcd. for $C_{22}H_{16}FO_3S$, 379.0810; found 379.0814.

Compound ***D5***. Yellow oil, 113 mg, 62% yield; purity, 96.6%; ^{1}H NMR (500 MHz, $CDCl_3$) δ 7.74 (d, J = 11.3 Hz, 1H),7.67-7.64 (m, 2H), 7.42-7.38 (m, 2H), 7.38-7.34 (m, 5H), 7.18 (d, J = 15.4 Hz, 1H), 6.64 (dd, J_1 = 15.6 Hz, J_2 = 11.3 Hz, 1H); ^{19}F NMR (471 MHz, $CDCl_3$) δ 57.0; ^{13}C NMR (126 MHz, $CDCl_3$) 146.6, 144.5 (d, J = 1.8 Hz) 135.0, 132.5, 132.4, 131.6 (d, J = 23.6 Hz), 130.6, 129.1, 128.1, 124.9, 121.1;

HRMS (EI) m/z: $[M]^+$ calcd for C16H12BrFO2S, 365.9725; found 365.9729.

Compound ***D6***. Yellow solid, 97 mg, 49% yield; purity, 96.0%; mp: 113–115°C; ^{1}H NMR (500 MHz, $CDCl_3$) δ 7.70 (d, J = 11.1 Hz, 1H), 7.49-7.35 (m, 12H), 7.14 (d, J = 15.7 Hz, 1H), 7.12-7.09 (m, 2H), 6.71 (dd, J_1 = 15.5 Hz, J_2 = 11.1 Hz, 1H), 5.14 (s, 2H); ^{19}F NMR (471 MHz, $CDCl_3$) δ 56.2; ^{13}C NMR (126 MHz, $CDCl_3$) δ 160.3, 145.4, 143.6 (d, J = 1.8 Hz), 136.5, 135.3, 132.9 (d, J = 22.7 Hz), 132.3, 130.3, 129.1, 128.8, 128.4, 127.9, 127.7, 121.8, 121.3, 115.5, 70.3; HRMS (EI) m/z: $[M]^+$ calcd for $C_{23}H_{19}FO_3S$, 394.1039; found 394.1032.

Compound ***D7***. Yellow solid, 100 mg, 59% yield; purity, 97.7%; mp: 92–94°C; ^{1}H NMR (500 MHz, $CDCl_3$) δ 7.99 (d, J = 8.9 Hz, 2H), δ 7.94 (t, J = 6.2 Hz, 2H), 7.82 (d, J = 11.2 Hz, 1H), 7.64-7.58 (m, 2H), 7.55 (dd, J_1 = 8.4 Hz, J_2 = 1.7 Hz, 1H), 7.37-7.34 (m, 2H), 7.32-7.29 (m, 3H), 7.19 (d, J = 15.5 Hz, 1H), 6.72 (dd, J_1 = 15.4 Hz, J_2 = 11.1 Hz, 1H); ^{19}F NMR (471 MHz, $CDCl_3$) δ 56.9; ^{13}C NMR (126 MHz, $CDCl_3$) 146.0, 144.2 (d, J = 2.7 Hz) 135.2, 133.8, 133.2, 133.0 (d, J = 22.7 Hz) 131.0, 130.4, 129.0, 129.0, 128.6, 128.0, 127.7, 127.4, 127.1, 126.5, 121.6; HRMS (EI) m/z: $[M-H]^+$ calcd. for $C_{20}H_{14}FO_2S$, 337.0704; found 337.0707.

3. General procedure for the synthesis of series D8–D14

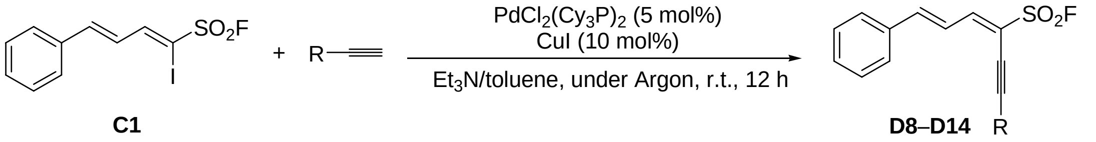

An oven-dried reaction tube was charged with **C1** (0.5 mmol, 169 mg, 1 eq.), $PdCl_2$Bis(tricyclohexylphosphine) (5 mol%), CuI (10 mol%), dry toluene (1.5 mL), Et_3N (0.5 mL) and alkyne (1.5 mmol, 1.5 eq.) under nitrogen atmosphere, and the mixture was stirred at room temperature for 12 h. The mixture was concentrated to dryness and the residue was purified through silica gel chromatography using a mixture of petroleum ether / ethyl acetate = 40:1 (v/v) as eluent to afford the desired product **D**, and the detailed synthesis process was reported in the literature[44].

Compound ***D8***. Yellow solid, 156 mg, 80% yield; mp: 109–111°C; ^{1}H NMR (500 MHz, $CDCl_3$) δ 7.75-7.71 (m, 2H), 7.60-7.57 (m, 3H), 7.53 (d, J = 7.8 Hz, 1H)

7.47-7.41 (m, 3H), 7.31-7.21 (m, 3H); ^{19}F NMR (471 MHz, $CDCl_3$) δ 57.6; ^{13}C NMR (126 MHz, $CDCl_3$) δ 148.5 (d, *J* = 1.9 Hz), 147.8, 135.0, 134.6, 133.2, 131.1, 130.6, 130.2, 129.3, 128.5, 123.3, 122.5, 122.4, 116.5 (d, *J* = 28.1 Hz), 100.8, 79.1; HRMS (EI) m/z: $[M]^+$ calcd. for $C_{18}H_{12}BrFO_2S$, 389.9725; found 389.9711.

Compound ***D9***. Yellow solid, 102 mg, 62% yield; purity, 97.4%; mp: 119–121°C; ^{1}H NMR (500 MHz, $CDCl_3$) δ 7.73 (d, *J* = 11.2 Hz, 1H), 7.61-7.56 (m, 3H), 7.46-7.40 (m, 4H), 7.37 (dd, J_1 = 15.4 Hz, J_2 = 11.2 Hz, 1H), 7.26-7.17 (m, 3H); ^{19}F NMR (471 MHz, $CDCl_3$) δ 57.8, -108.6 (m, 1F); ^{13}C NMR (126 MHz, $CDCl_3$) δ 163.0 (d, *J* = 253.4 Hz), 148.7, 147.6, 135.1, 133.4, 131.8 (d, *J* = 8.1 Hz), 131.0, 129.3, 128.5, 124.4 (d, *J* = 3.6 Hz), 122.7, 116.6 (d, *J* = 27.2 Hz), 115.9 (d, *J* = 20.9 Hz), 110.3 (d, *J* = 15.4 Hz), 96.1, 83.0 (d, *J* = 2.7 Hz); HRMS (EI) m/z: $[M]^+$ calcd for $C_{18}H_{12}F_2O_2S$, 330.0526; found 330.0520.

Compound ***D10***. Yellow solid, 152 mg, 78% yield; purity, 99.3%; mp: 97–99°C; ^{1}H NMR (500 MHz, $CDCl_3$) δ 7.74-7.68 (m, 1H), 7.59-7.54 (m, 4H), 7.47-7.42 (m, 5H), 7.27-7.20 (m, 2H); ^{19}F NMR (471 MHz, $CDCl_3$) δ 57.5; ^{13}C NMR (126 MHz, $CDCl_3$) δ 148.2, 147.5, 135.0, 133.4, 132.1, 131.1, 129.3, 128.4, 124.6, 122.5, 120.3, 116.7 (d, *J* = 27.2 Hz), 101.5, 79.1; HRMS (EI) m/z: $[M]^+$ calcd for $C_{18}H_{12}BrFO_2S$, 389.9725; found 389.9721.

Compound ***D11***. Yellow solid, 115 mg, 74% yield; purity, 97.3%; mp: 104–106°C; ^{1}H NMR (500 MHz, $CDCl_3$) δ 7.70 (d, *J* = 10.2 Hz, 1H), 7.62-7.57 (m, 4H), 7.45-7.40 (m, 6H), 7.31-7.21 (m, 2H); ^{19}F NMR (471 MHz, $CDCl_3$) δ 57.2; ^{13}C NMR (126 MHz, $CDCl_3$) δ 147.7, 147.1, 135.1, 132.1, 130.9, 130.0, 129.2, 128.7, 128.4, 122.6, 121.4, 117.0 (d, *J* = 27.2 Hz), 102.7, 78.0; HRMS (EI) m/z: $[M]^+$ calcd for $C_{18}H_{13}FO_2S$, 312.0620; found 312.0628.

Compound ***D12***. Yellow solid, 123 mg, 75% yield; mp: 126–128°C; ^{1}H NMR (500 MHz, $CDCl_3$) δ 7.67 (d, *J* = 10.5 Hz, 1H), 7.59-7.57 (m, 2H), 7.50 (d, *J* = 8.1 Hz, 2H), 7.45-7.41 (m, 3H), 7.30-7.19 (m, 4H), 2.42 (s, 3H); ^{19}F NMR (471 MHz, $CDCl_3$) δ 57.0; ^{13}C NMR (126 MHz, $CDCl_3$) δ 147.3, 146.8, 140.5, 135.2, 132.0, 130.9, 129.5, 129.2, 128.3, 122.7, 118.3, 117.2 (d, *J* = 27.3 Hz), 103.1, 77.5, 21.8; HRMS (EI) m/z: $[M]^+$ calcd for $C_{19}H_{15}FO_2S$, 326.0777; found 326.0774.

Compound ***D13***. Yellow solid, 122 mg, 77% yield; mp: 98–100°C; ^{1}H NMR (500 MHz, CDCl$_3$) δ 7.71-7.68 (m, 2H), 7.59-7.56 (m, 2H), 7.45-7.42 (m, 3H), 7.37 (dd, J_1 = 5.0 Hz, J_2 = 3.0 Hz, 1H), 7.28-7.20 (m, 3H); ^{19}F NMR (471 MHz, CDCl$_3$) δ 57.2; ^{13}C NMR (126 MHz, CDCl$_3$) δ 147.7 (d, J = 1.8 Hz), 147.1, 135.1, 131.3, 130.9, 129.9, 129.2, 128.4, 126.2, 122.6, 120.5, 117.0 (d, J = 26.3 Hz), 97.9, 77.6; HRMS (EI) m/z: [M]$^+$ calcd for $C_{16}H_{11}FO_2S_2$, 318.0184; found 318.0181.

Compound ***D14***. Yellow solid, 112 mg, 81% yield; purity, 95.5%; mp: 88–90°C; ^{1}H NMR (500 MHz, CDCl$_3$) δ 7.58 (t, J = 5.2 Hz 1H), 7.56-7.53 (m, 2H), 7.44-7.39 (m, 3H), 7.15-7.14 (m, 2H), 1.62-1.56 (m, 1H), 1.04-0.93 (m, 4H); ^{19}F NMR (471 MHz, CDCl$_3$) δ 55.9; ^{13}C NMR (126 MHz, CDCl$_3$) δ 147.1 (d, J = 1.8 Hz), 146.1, 135.3, 130.6, 129.2, 128.2, 122.7, 117.6 (d, J = 26.3 Hz), 108.6, 64.8, 9.7, 0.9; HRMS (EI) m/z: [M]$^+$ calcd for $C_{15}H_{13}FO_2S$, 276.0620; found 276.0626.

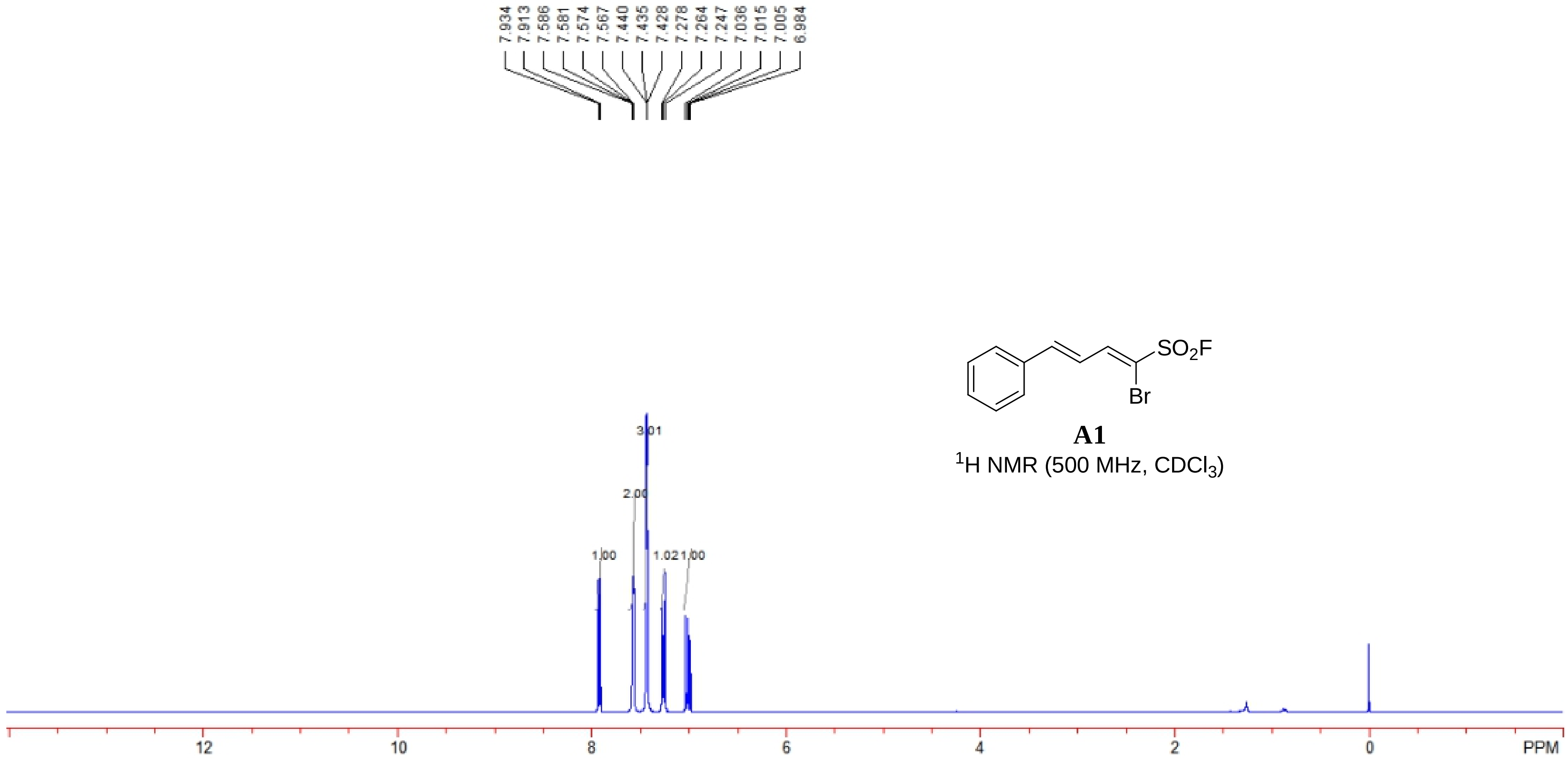

A1

^{1}H NMR (500 MHz, $CDCl_3$)

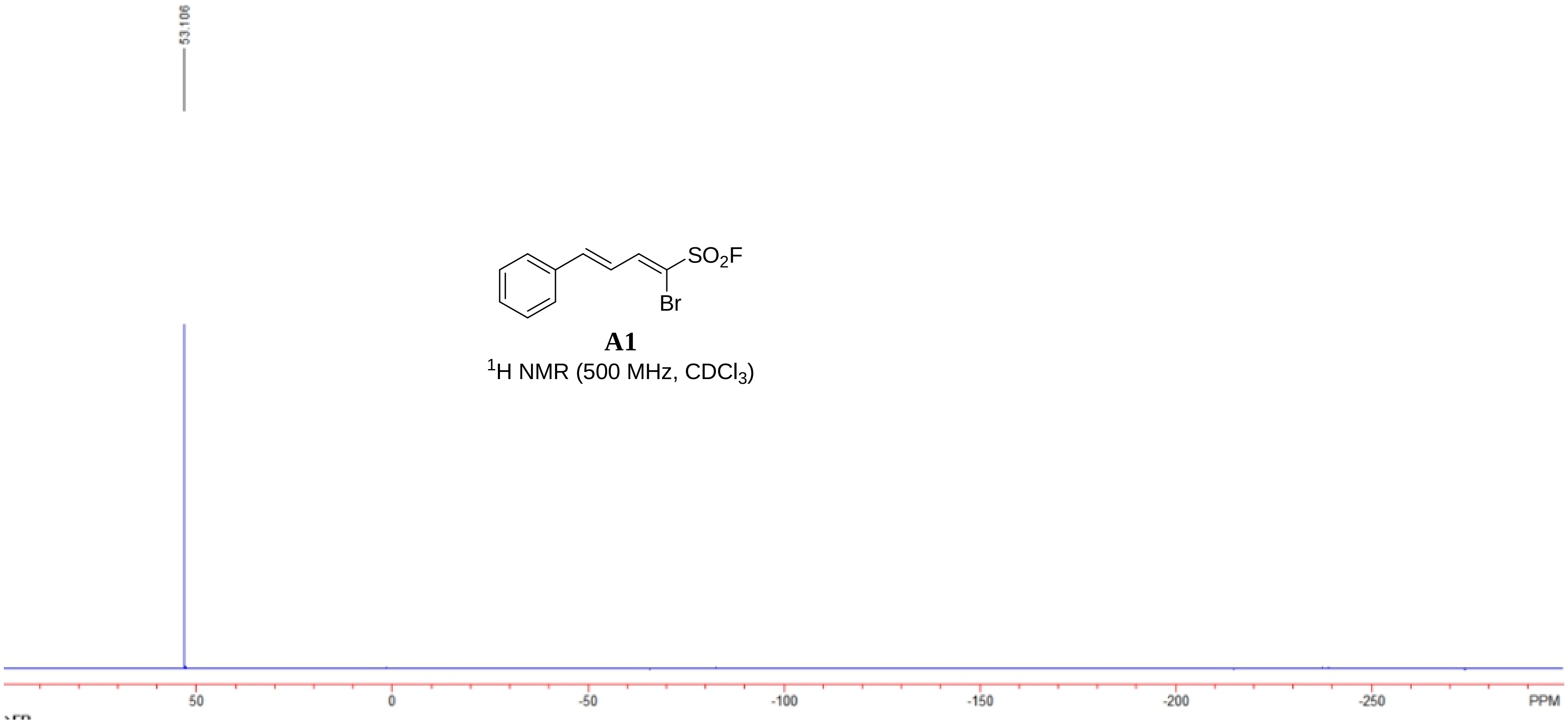

A1

^{1}H NMR (500 MHz, $CDCl_3$)

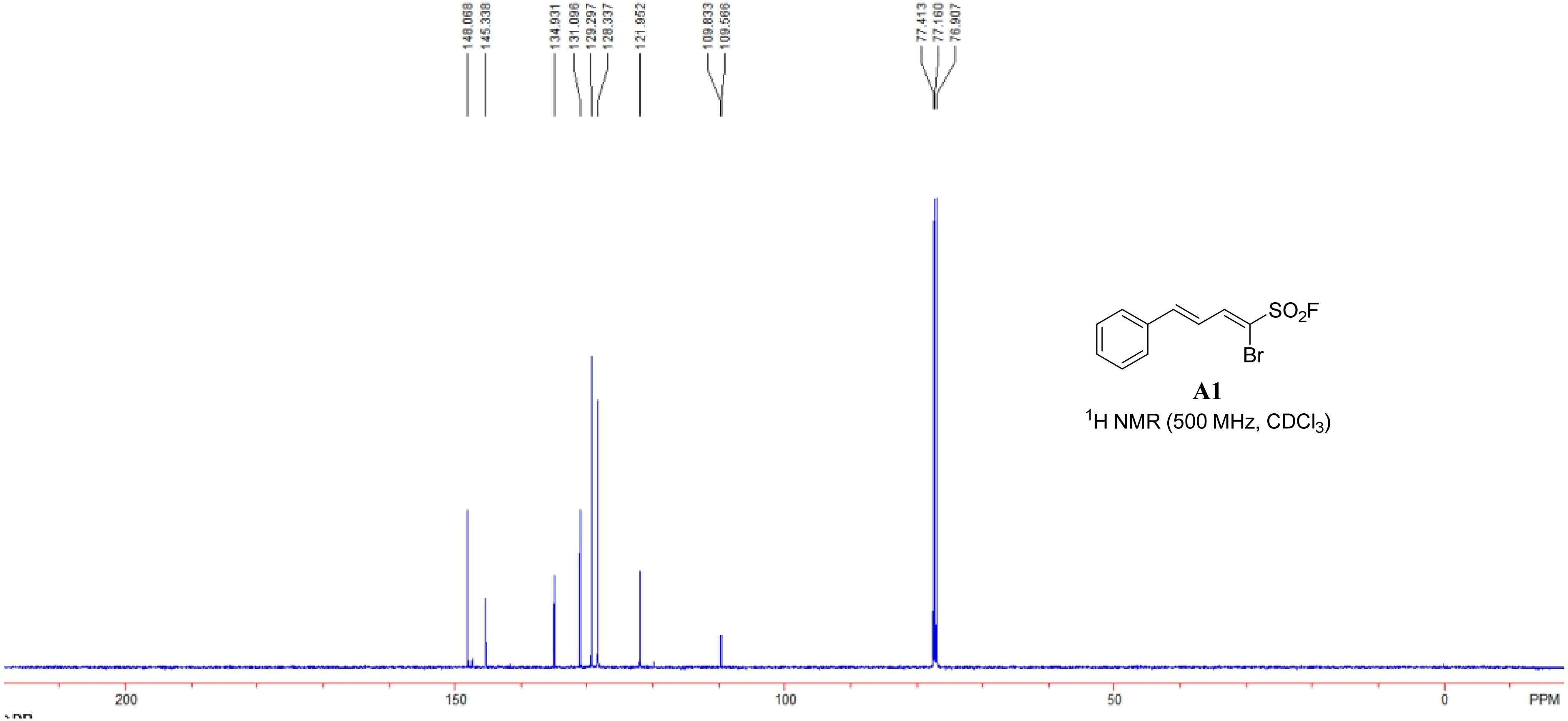

A1

^1H NMR (500 MHz, $CDCl_3$)

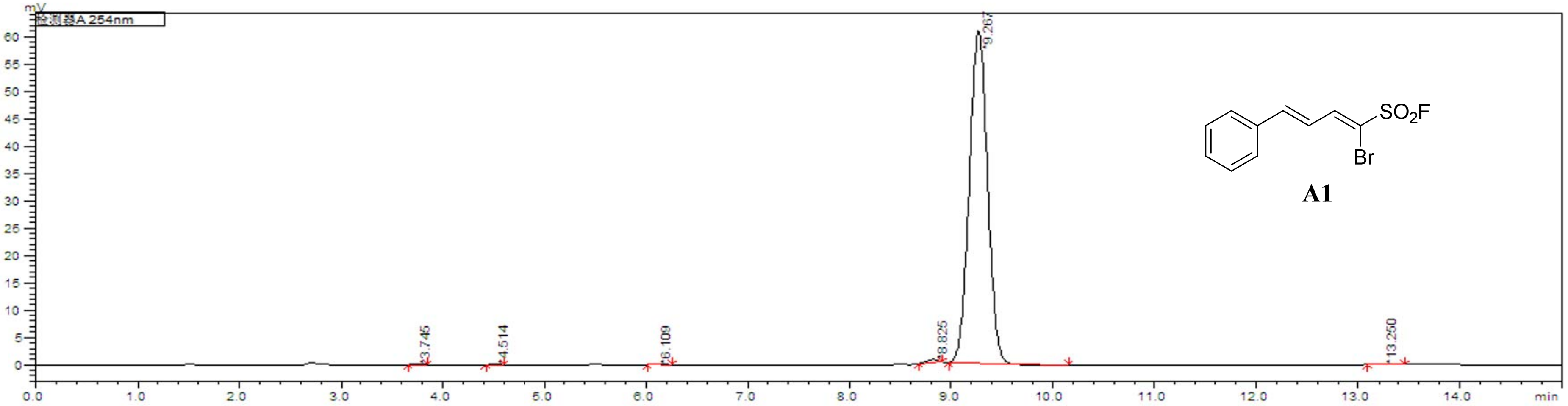

No.	Ret Time (min)	Area (mAU*min)	Rel.Area (%)
1	3.745	1153	0.15%
2	4.514	526	0.07%
3	6.109	1569	0.21%
4	8.825	4798	0.63%
5	9.267	751963	98.74%
6	13.250	1538	0.2%
Total		761546	

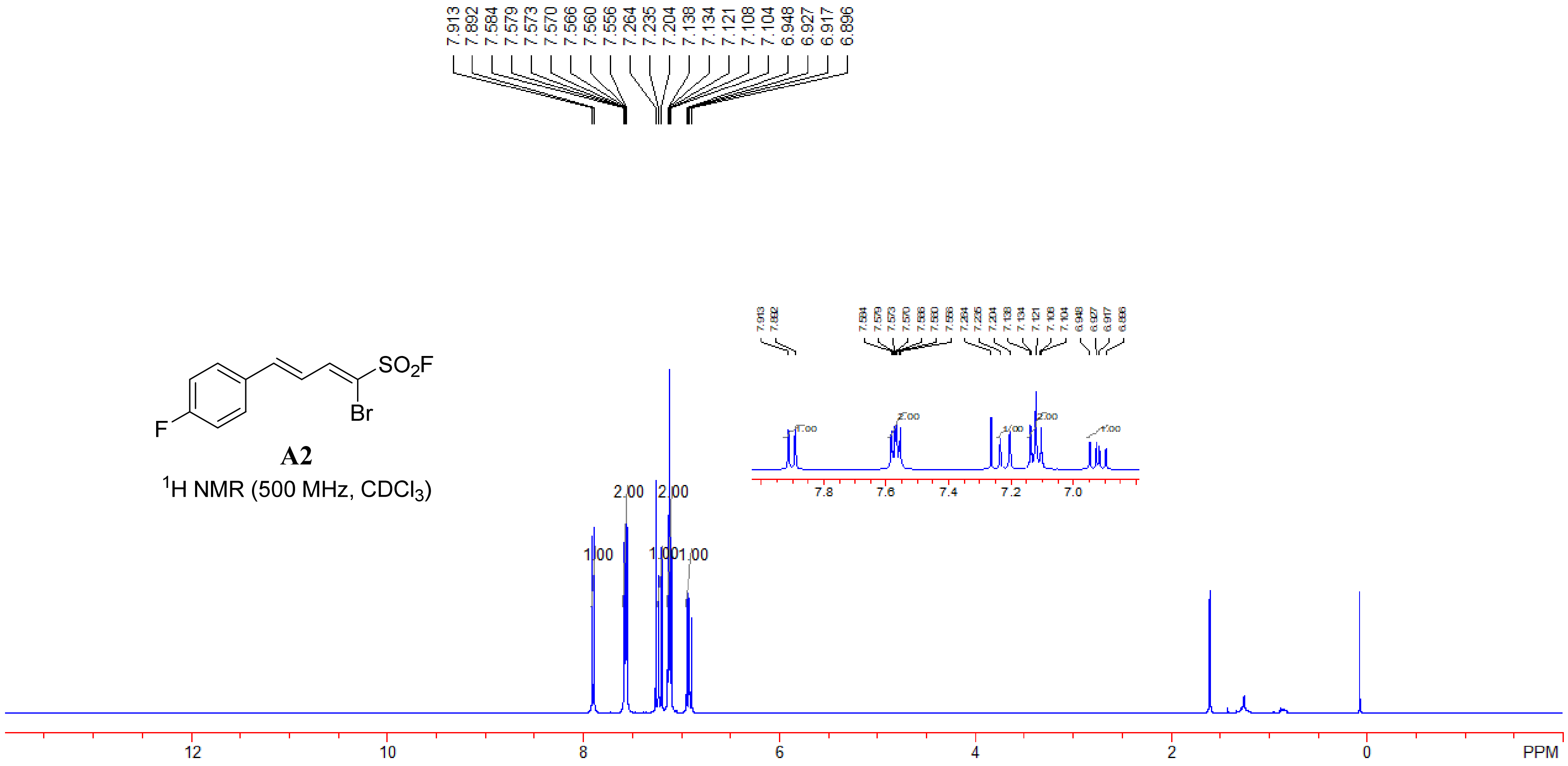

A2

^1H NMR (500 MHz, $CDCl_3$)

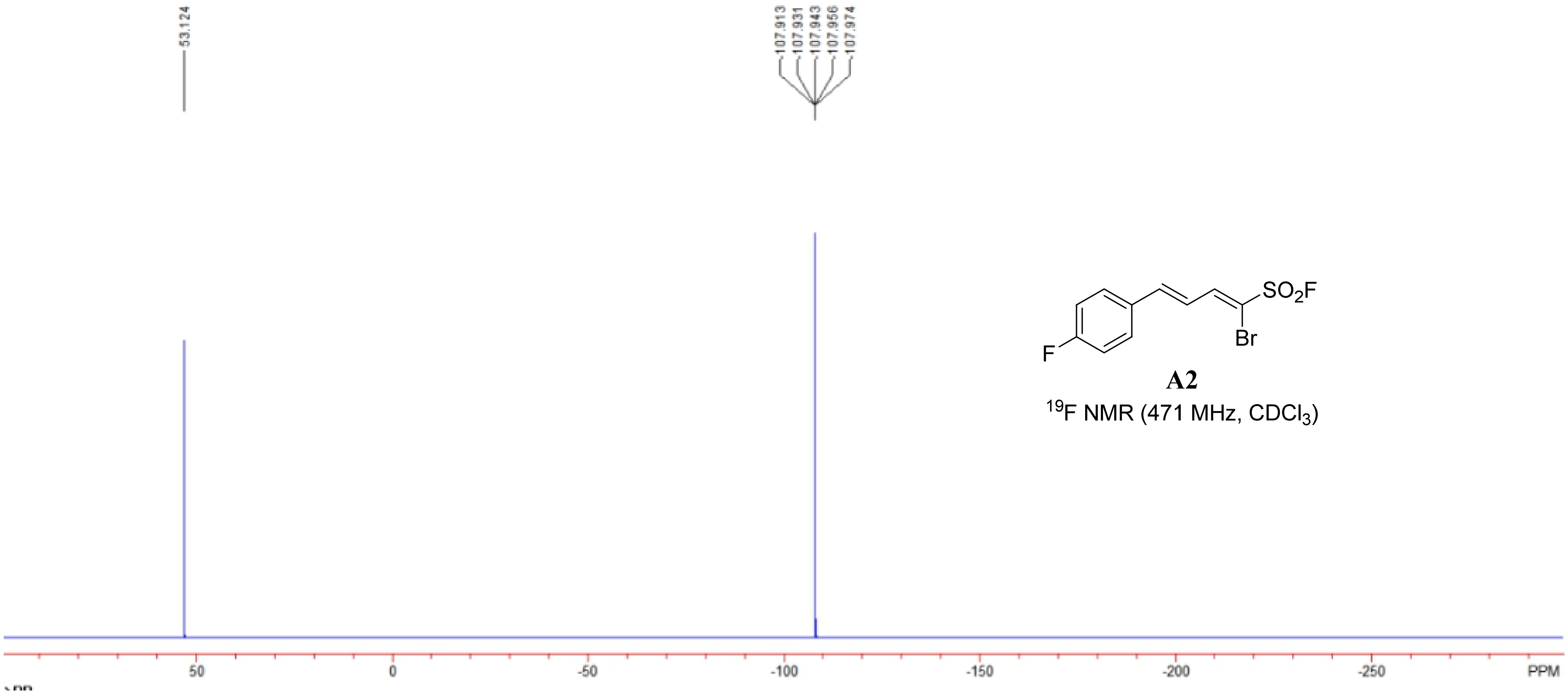

A2

^{19}F NMR (471 MHz, $CDCl_3$)

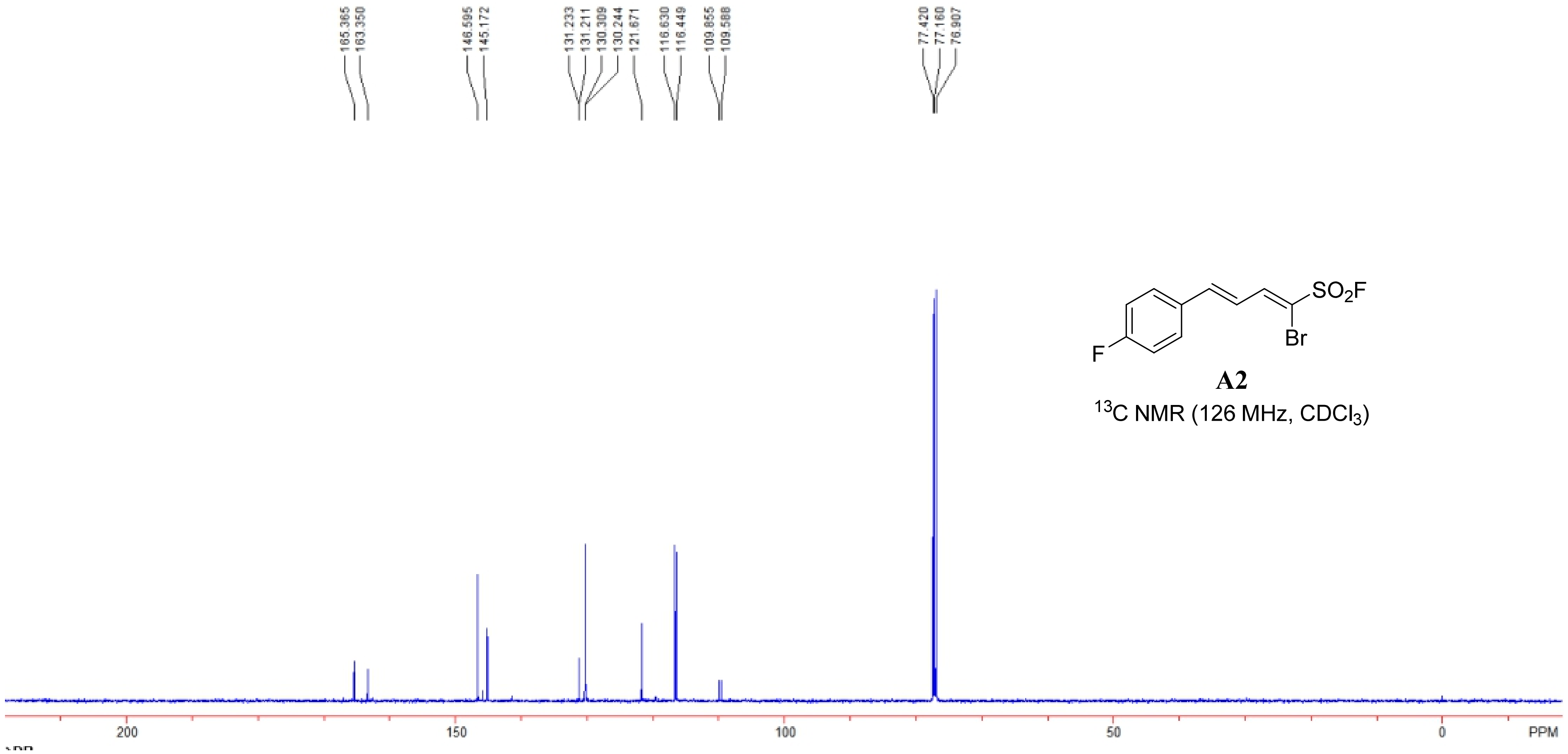

A2

^{13}C NMR (126 MHz, $CDCl_3$)

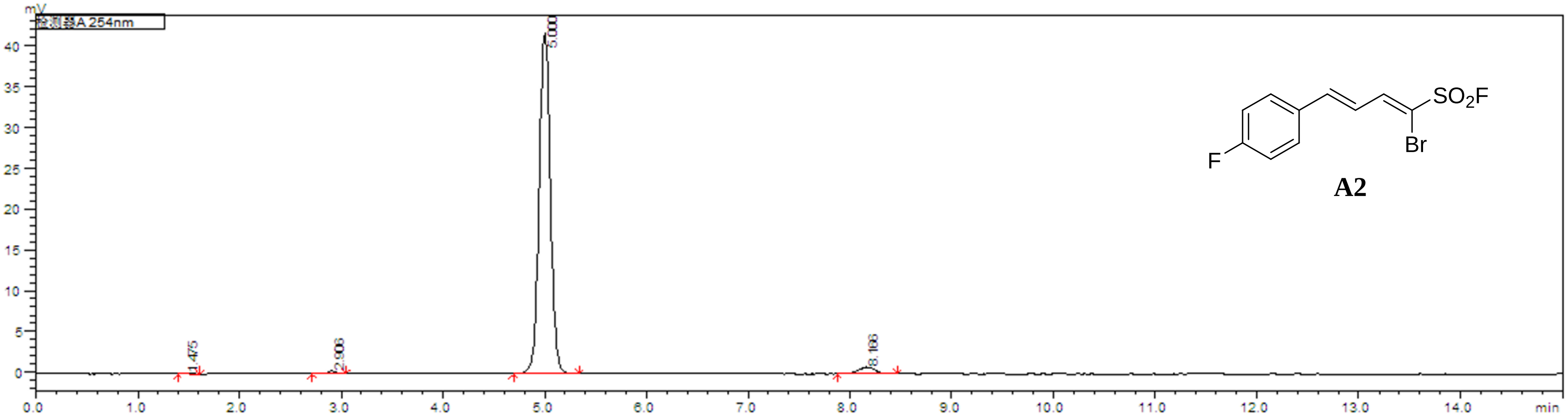

A2

No.	Ret Time (min)	Area (mAU*min)	Rel.Area (%)
1	1.475	1412	0.42%
2	2.905	2105	0.63%
3	5.000	321252	95.99%
4	8.166	9889	2.95%
Total		334658	

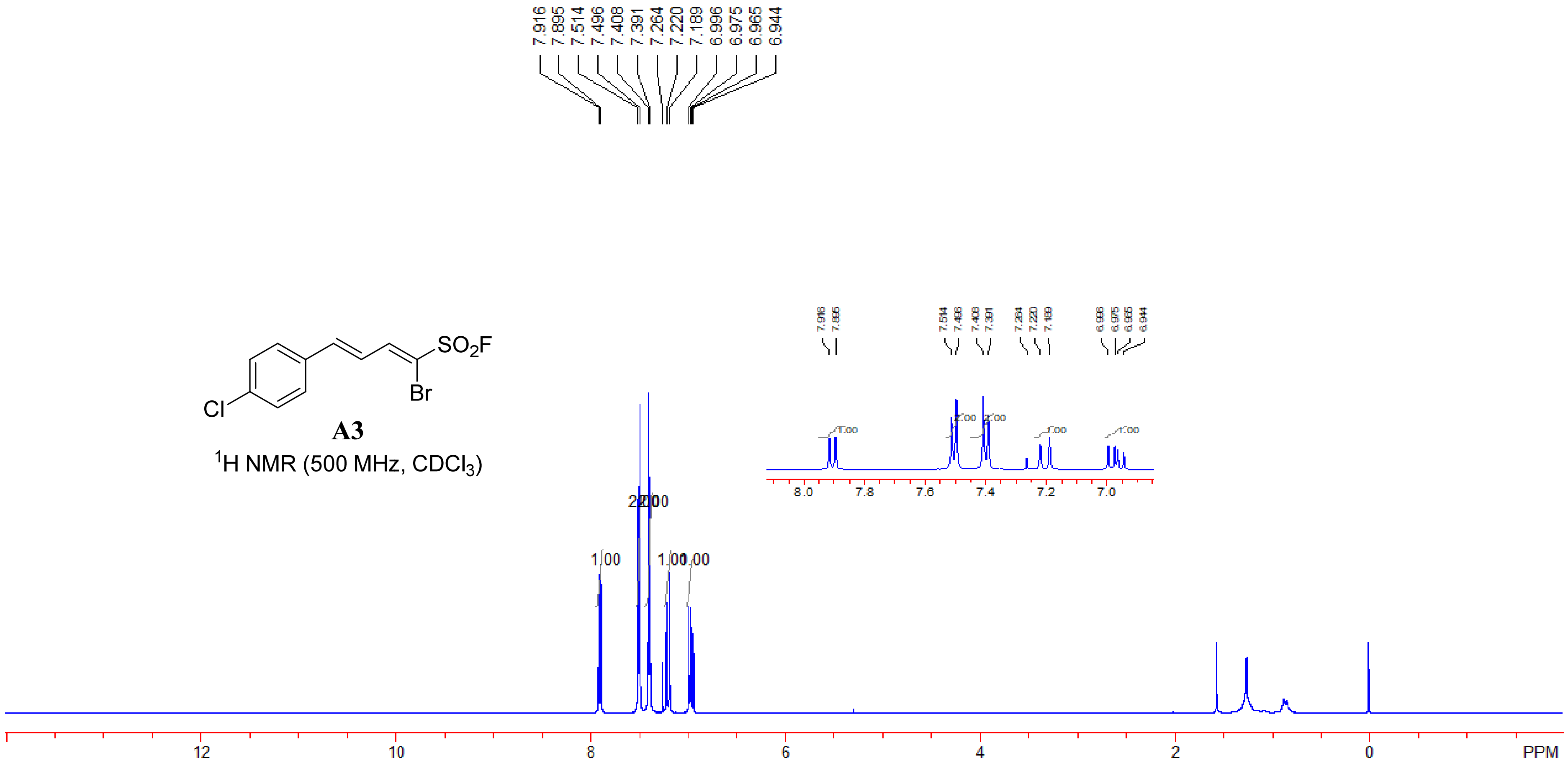

A3

^{1}H NMR (500 MHz, $CDCl_3$)

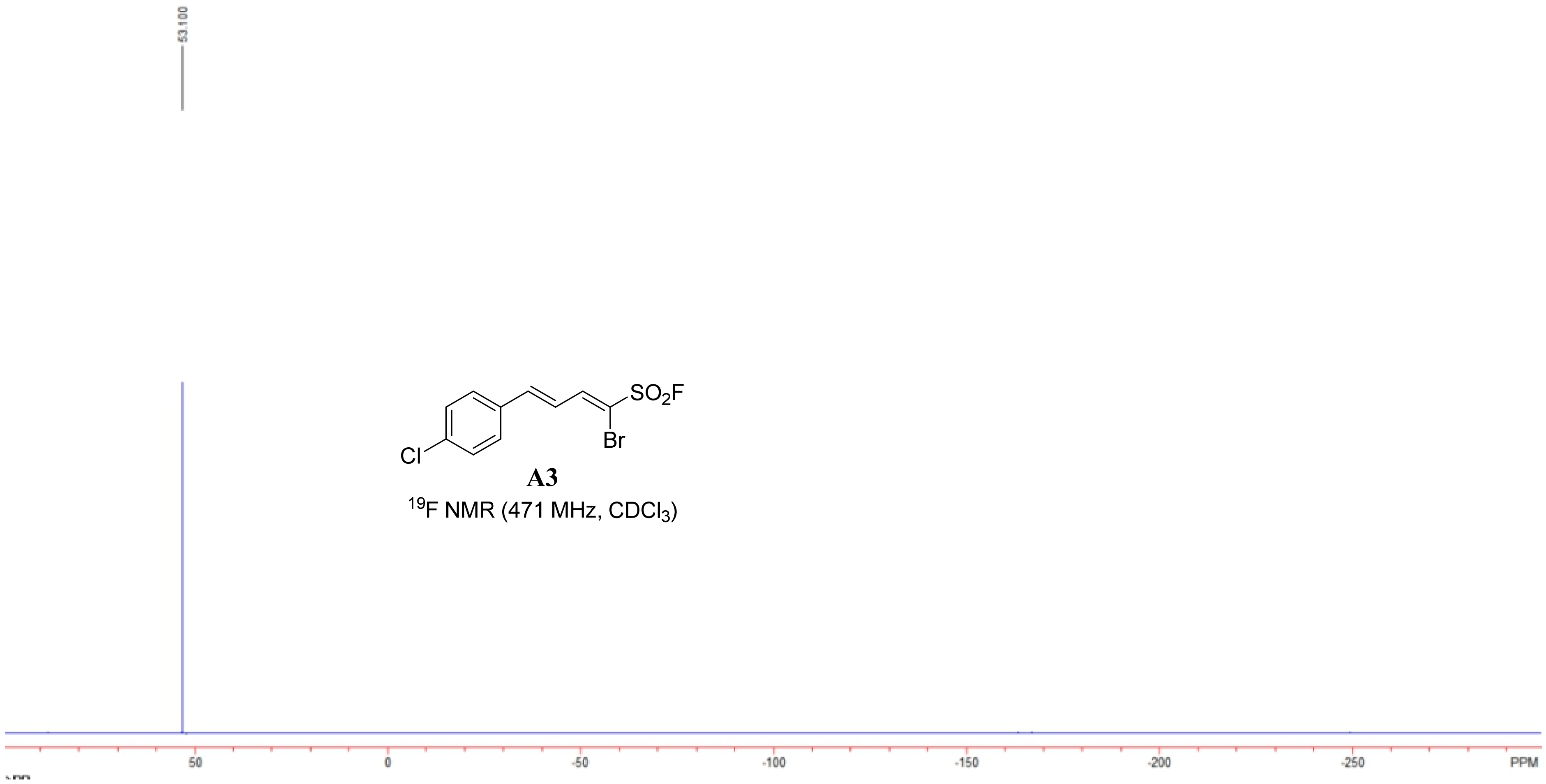

A3

^{19}F NMR (471 MHz, $CDCl_3$)

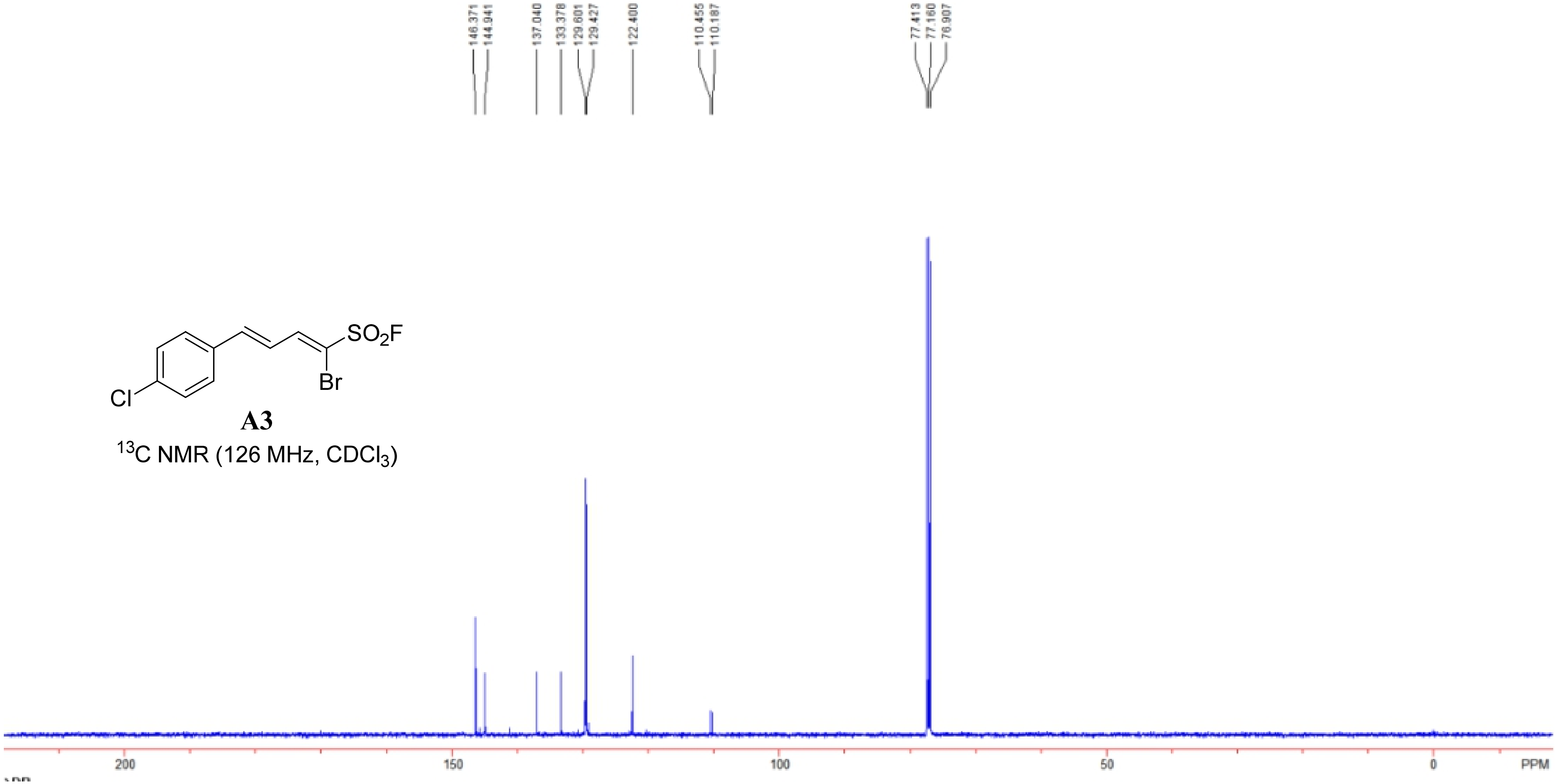

A3

^{13}C NMR (126 MHz, $CDCl_3$)

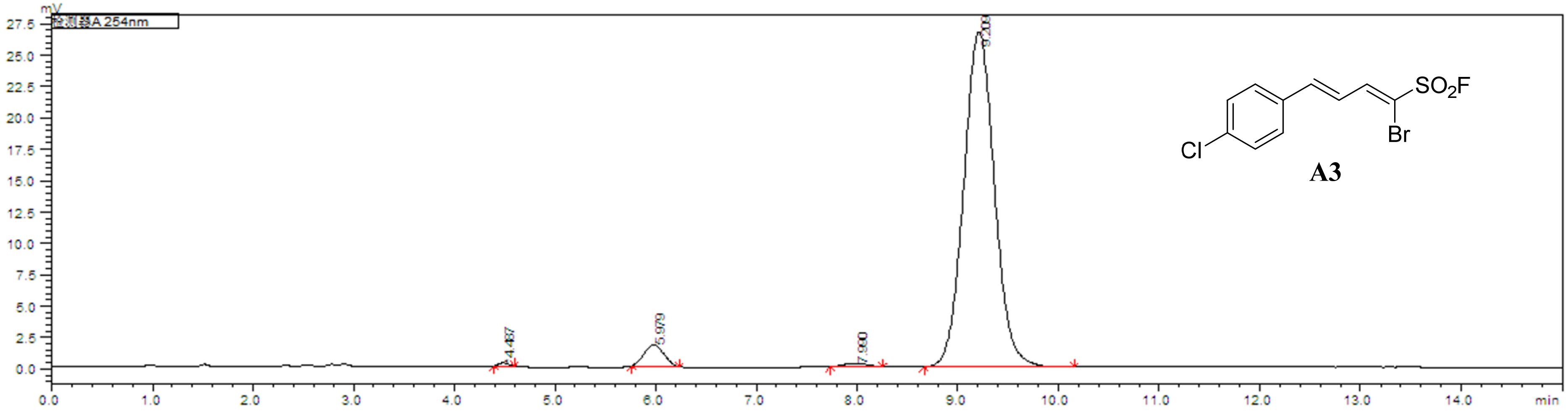

No.	Ret Time (min)	Area (mAU*min)	Rel.Area (%)
1	4.487	1625	0.27%
2	5.979	23076	3.88%
3	7.990	4451	0.75%
4	9.209	565811	95.12%
Total		594864	

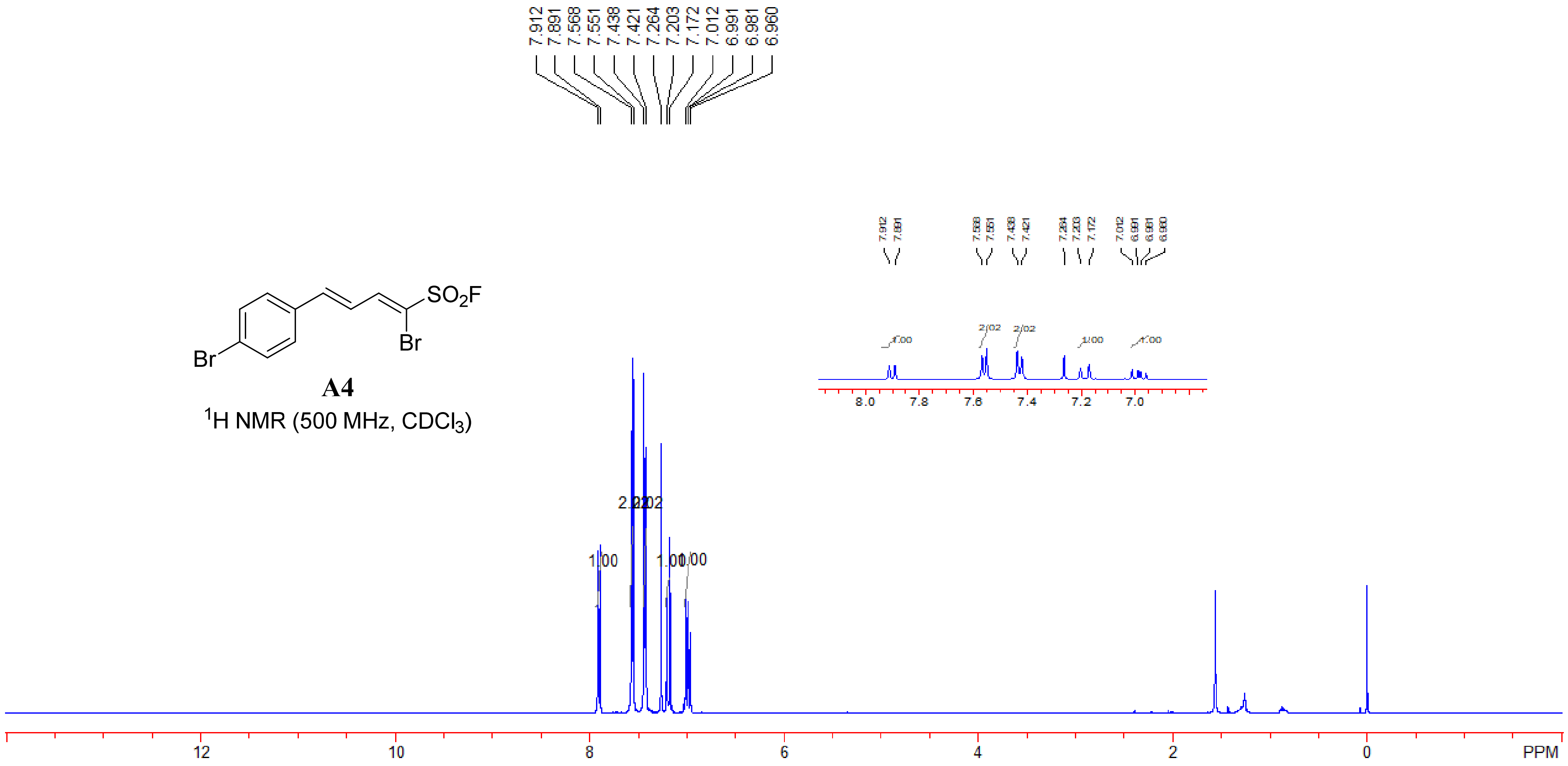

A4

^{1}H NMR (500 MHz, $CDCl_3$)

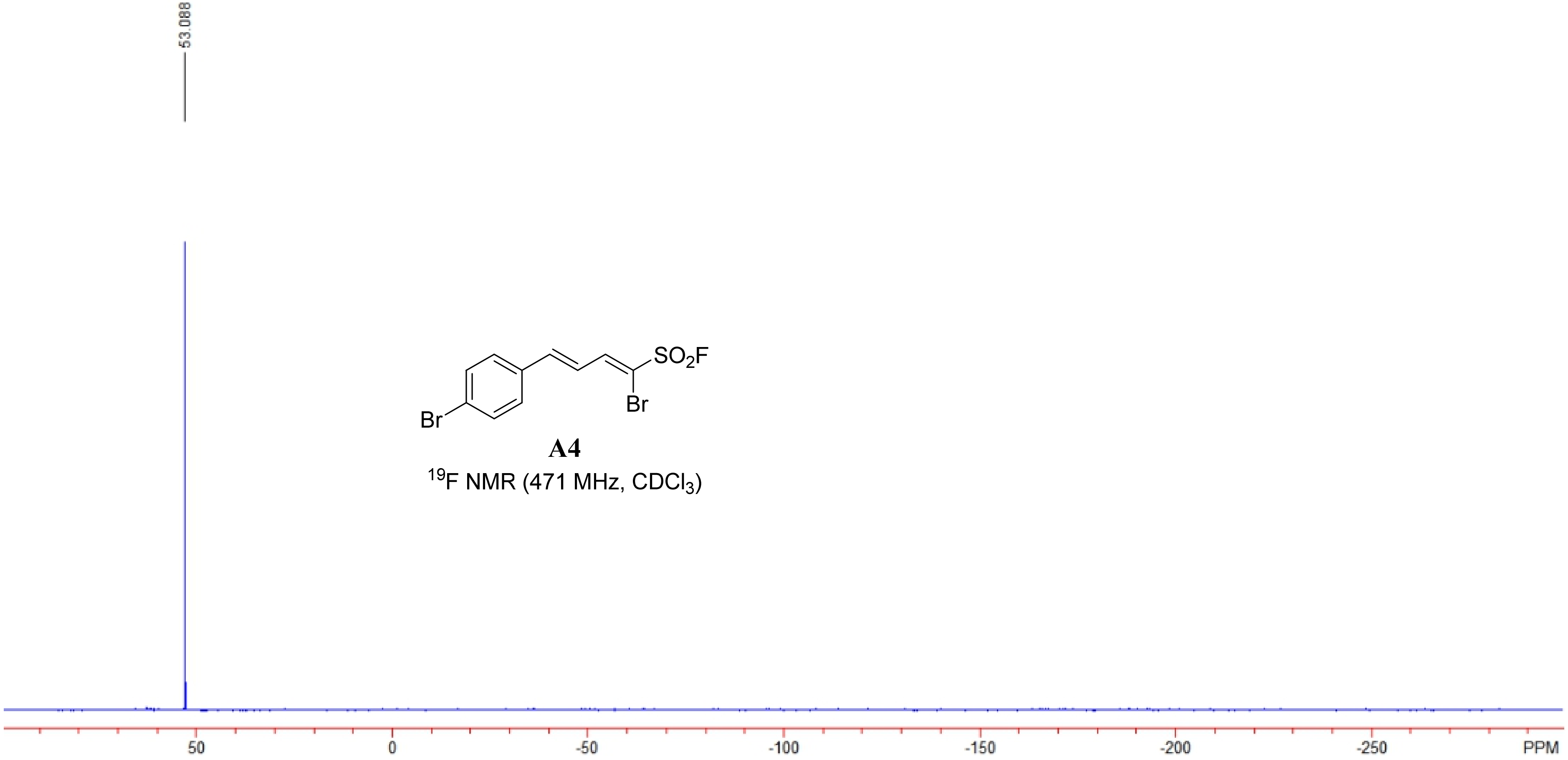

A4

^{19}F NMR (471 MHz, $CDCl_3$)

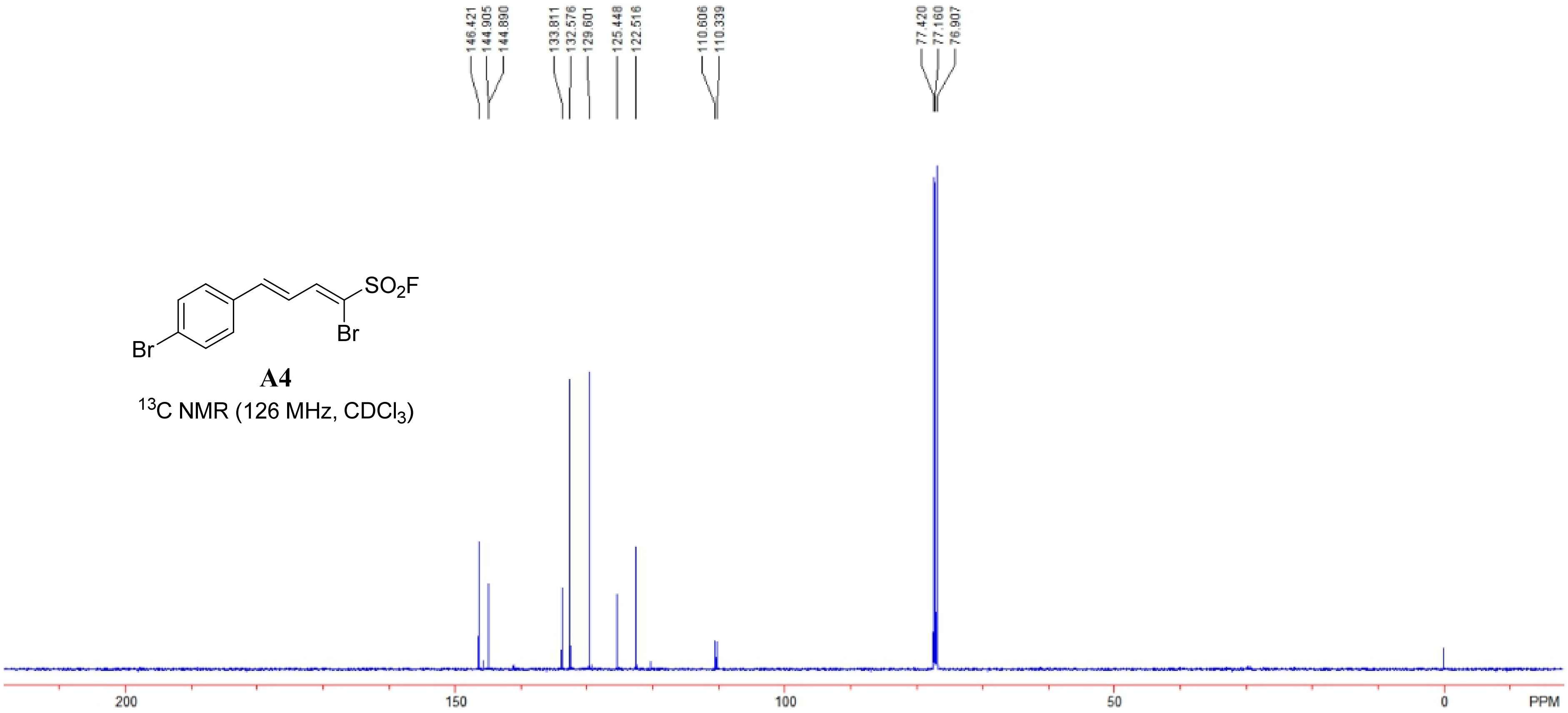

A4

^{13}C NMR (126 MHz, $CDCl_3$)

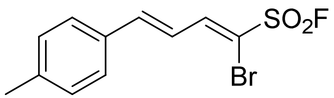

A5

^{1}H NMR (500 MHz, $CDCl_3$)

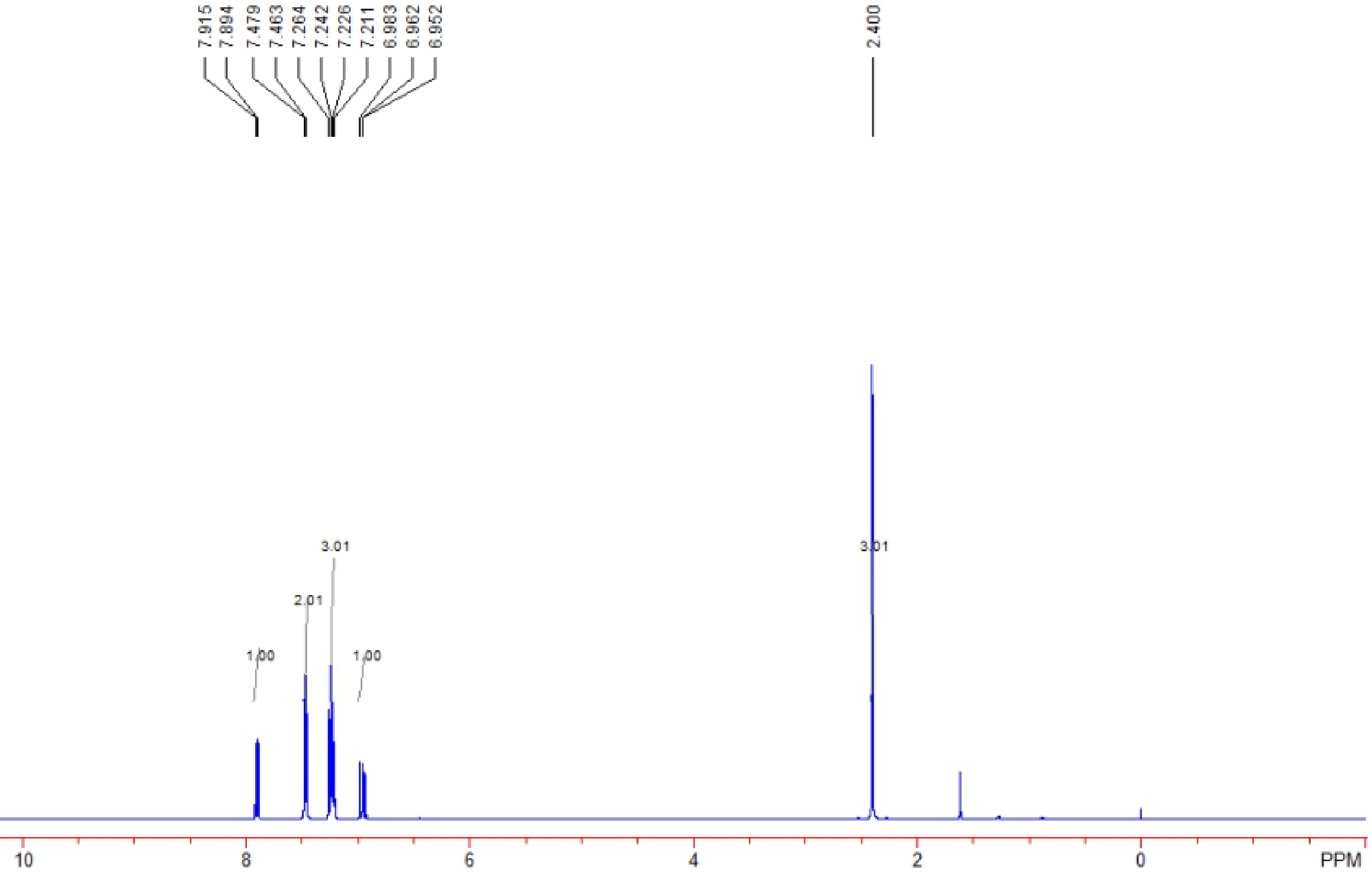

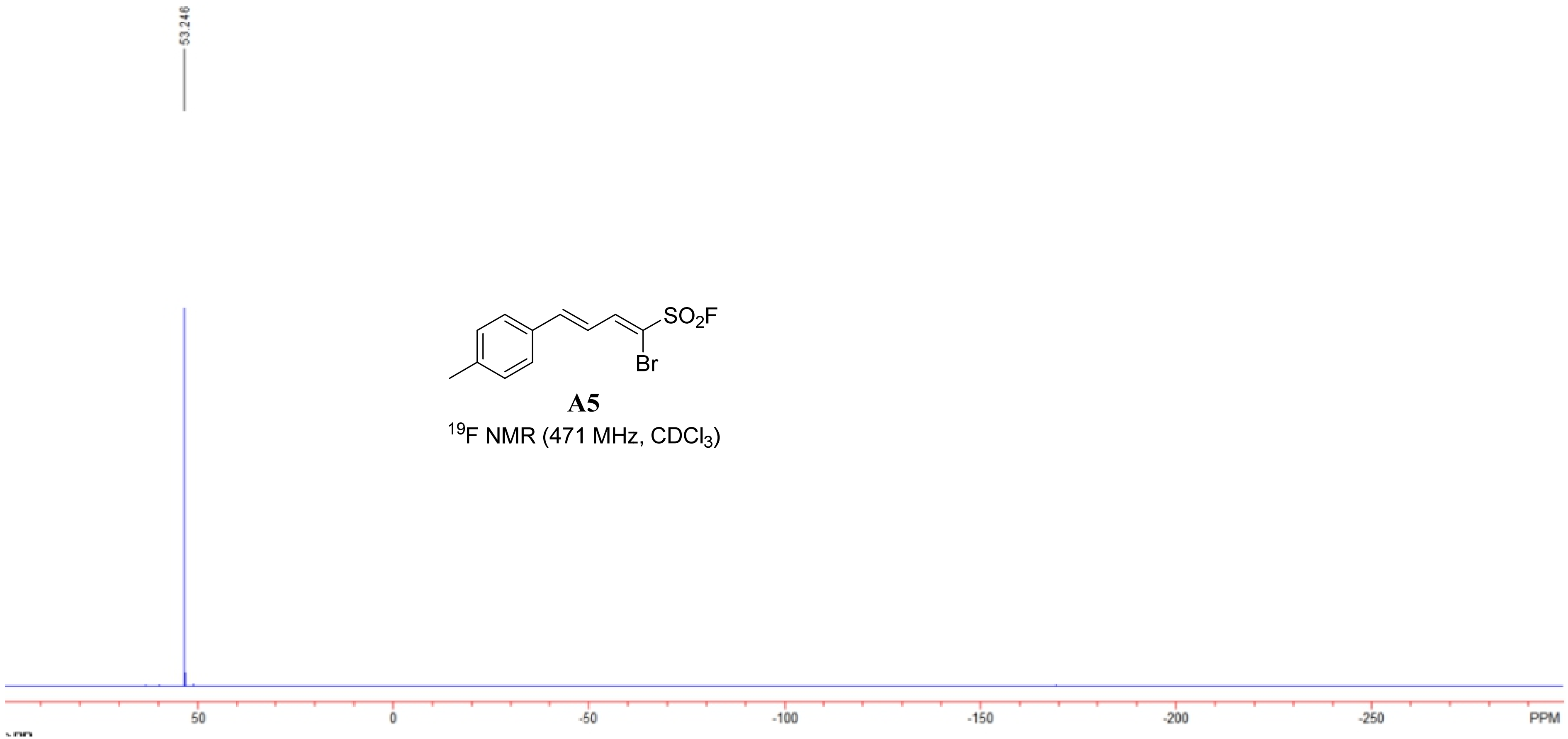

A5

^{19}F NMR (471 MHz, $CDCl_3$)

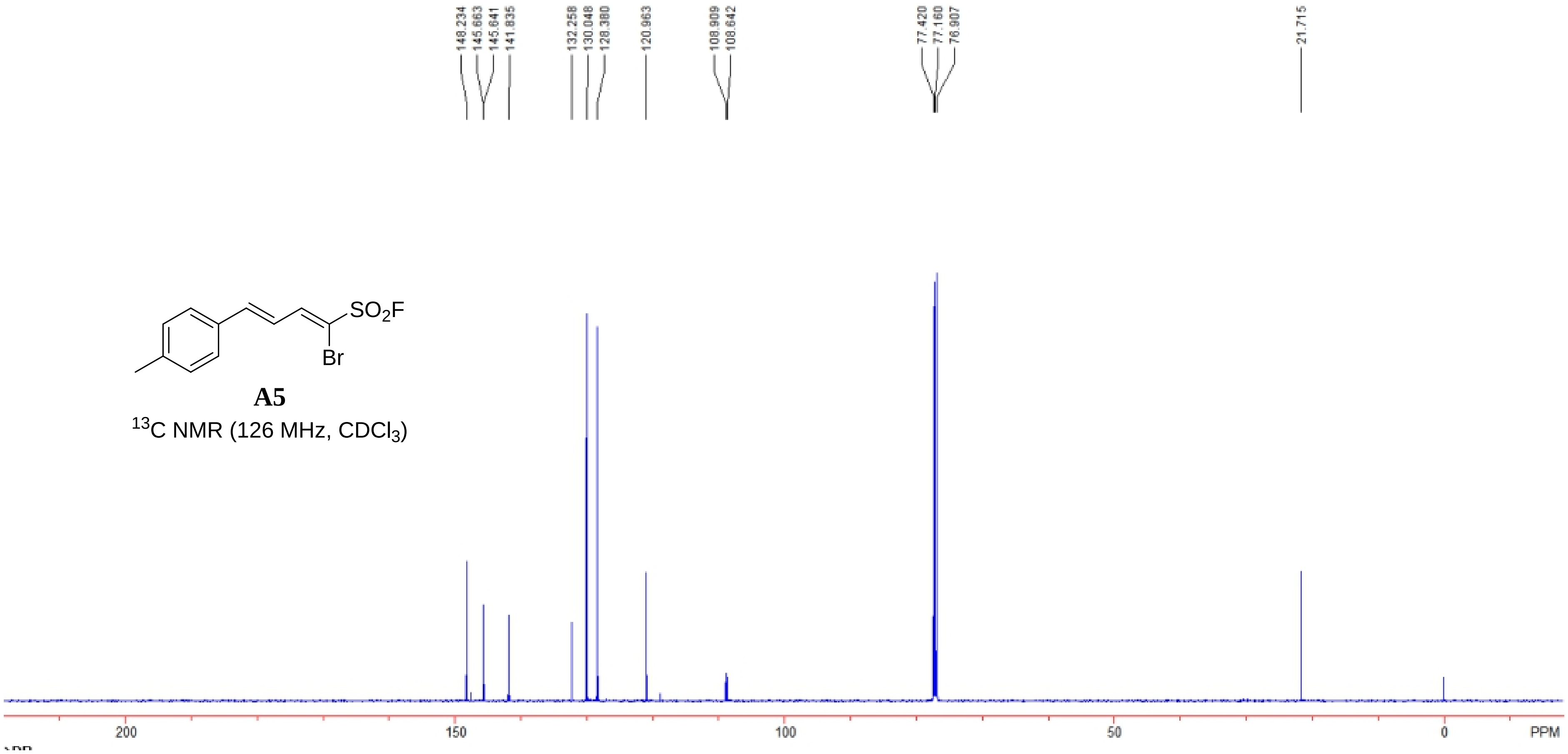

A5

^{13}C NMR (126 MHz, $CDCl_3$)

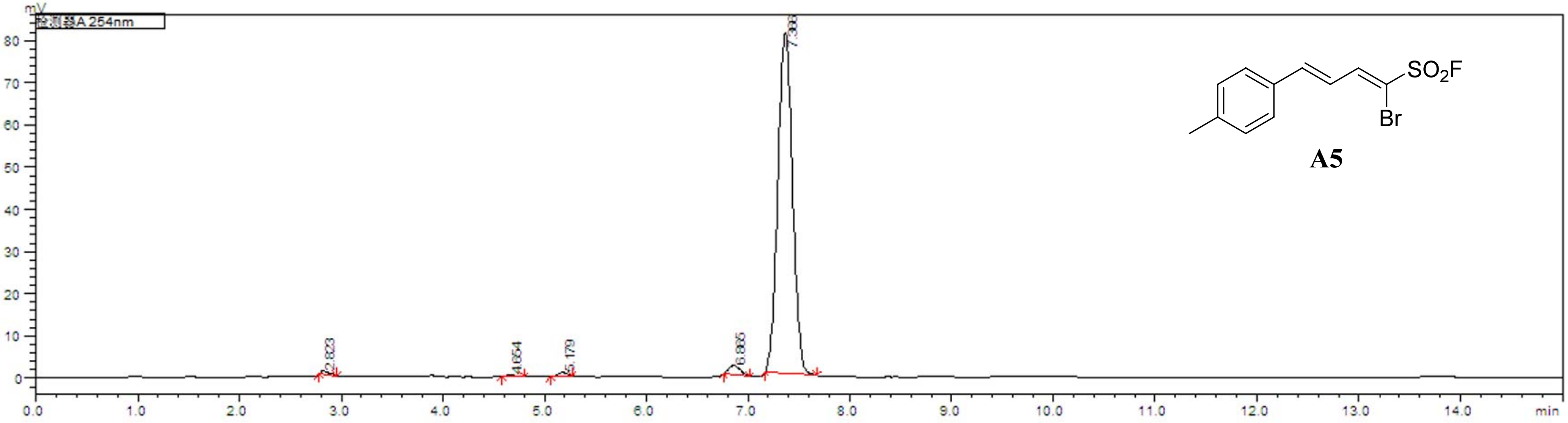

No.	Ret Time (min)	Area (mAU*min)	Rel.Area (%)
1	2.823	4738	0.57%
2	4.654	2025	0.24%
3	5.179	5852	0.70%
4	6.865	16645	2.00%
5	7.366	804116	96.48%
Total		833426	

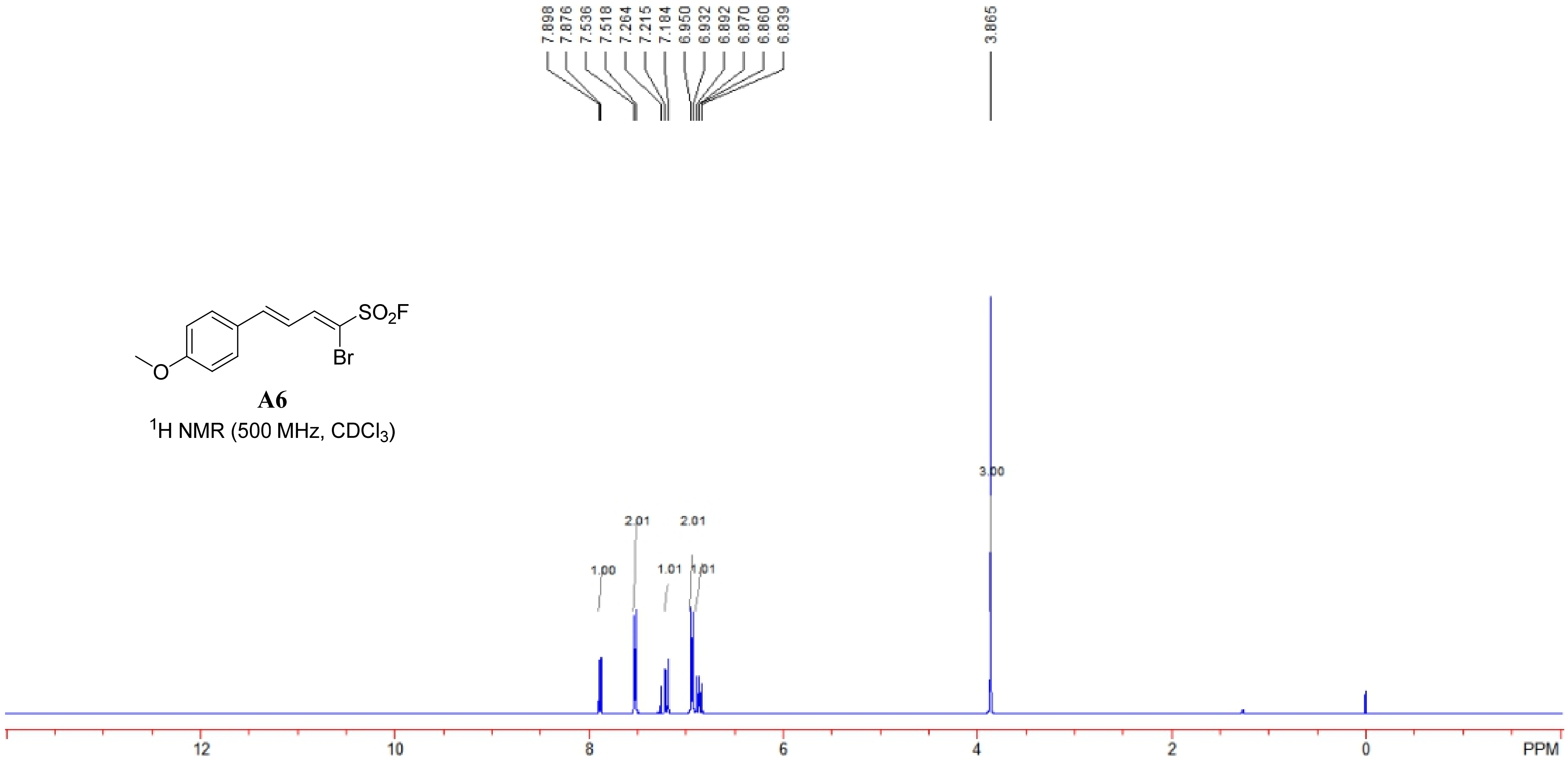

A6

^{1}H NMR (500 MHz, $CDCl_3$)

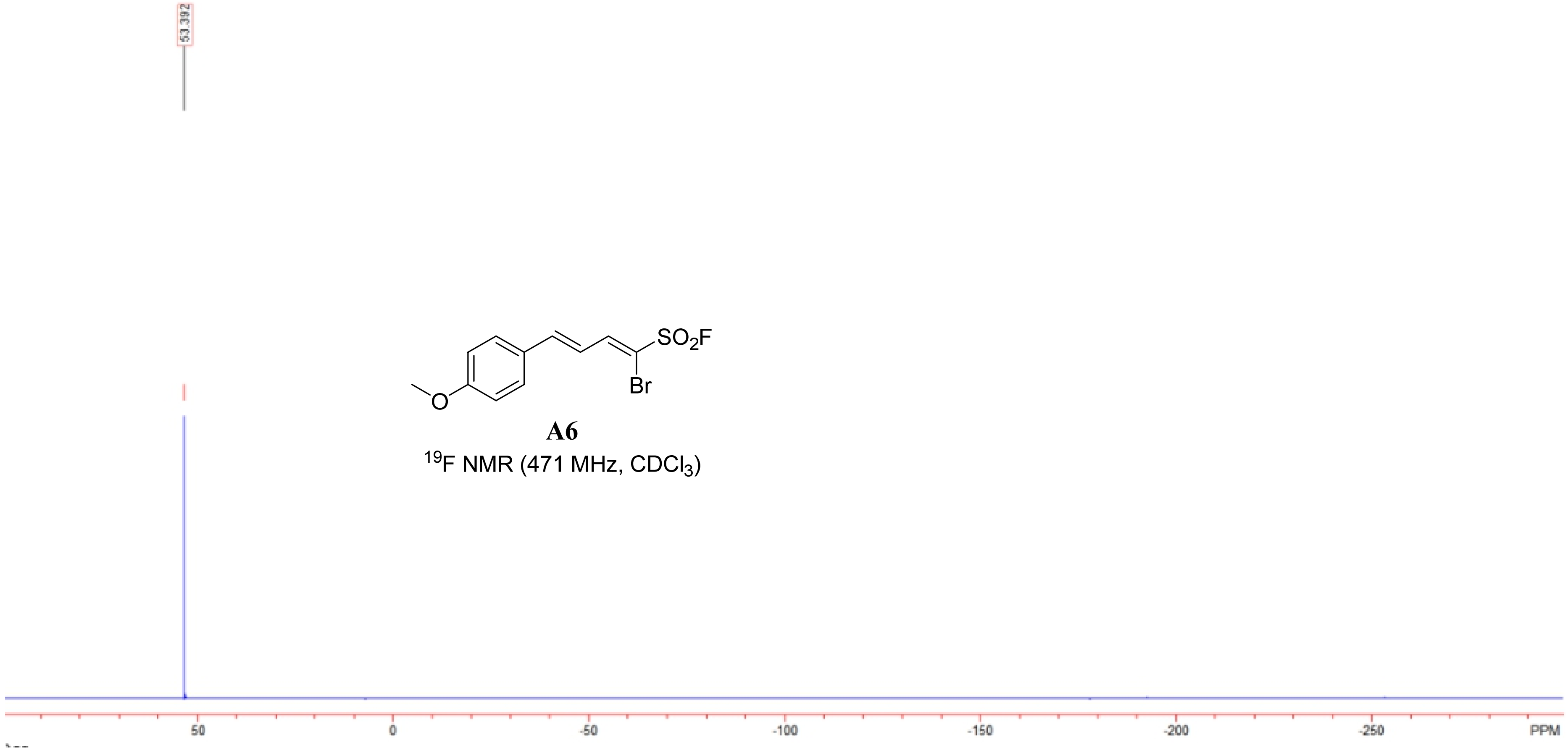

A6

^{19}F NMR (471 MHz, $CDCl_3$)

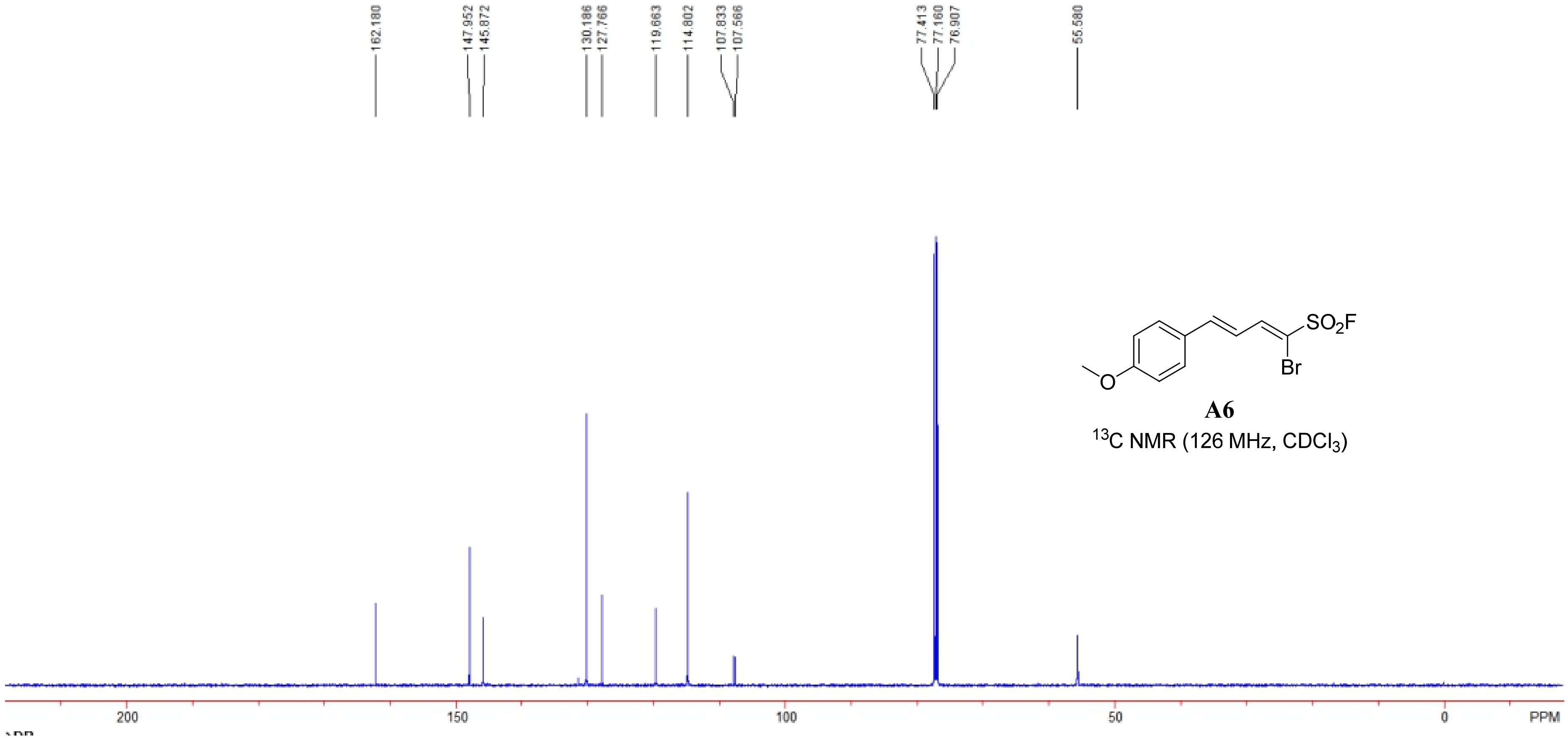

A6

^{13}C NMR (126 MHz, $CDCl_3$)

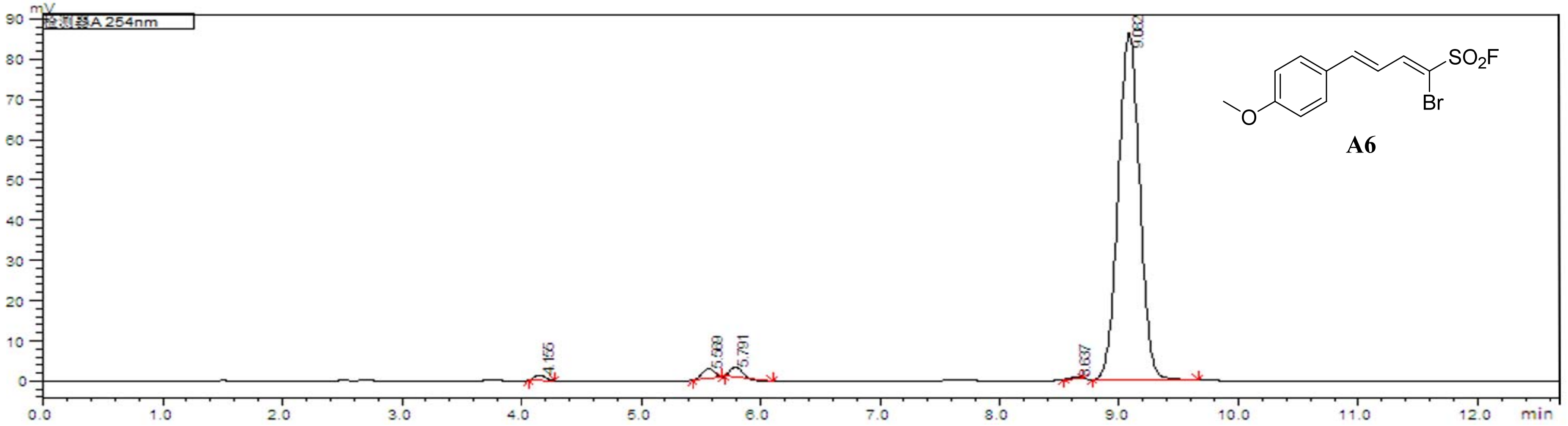

No.	Ret Time (min)	Area (mAU*min)	Rel.Area (%)
1	4.155	7857	0.71%
2	5.569	17081	1.54%
3	5.791	14958	1.35%
4	8.637	1877	0.17%
5	9.082	1068992	96.24%
Total		1110765	

A7

^1H NMR (500 MHz, $CDCl_3$)

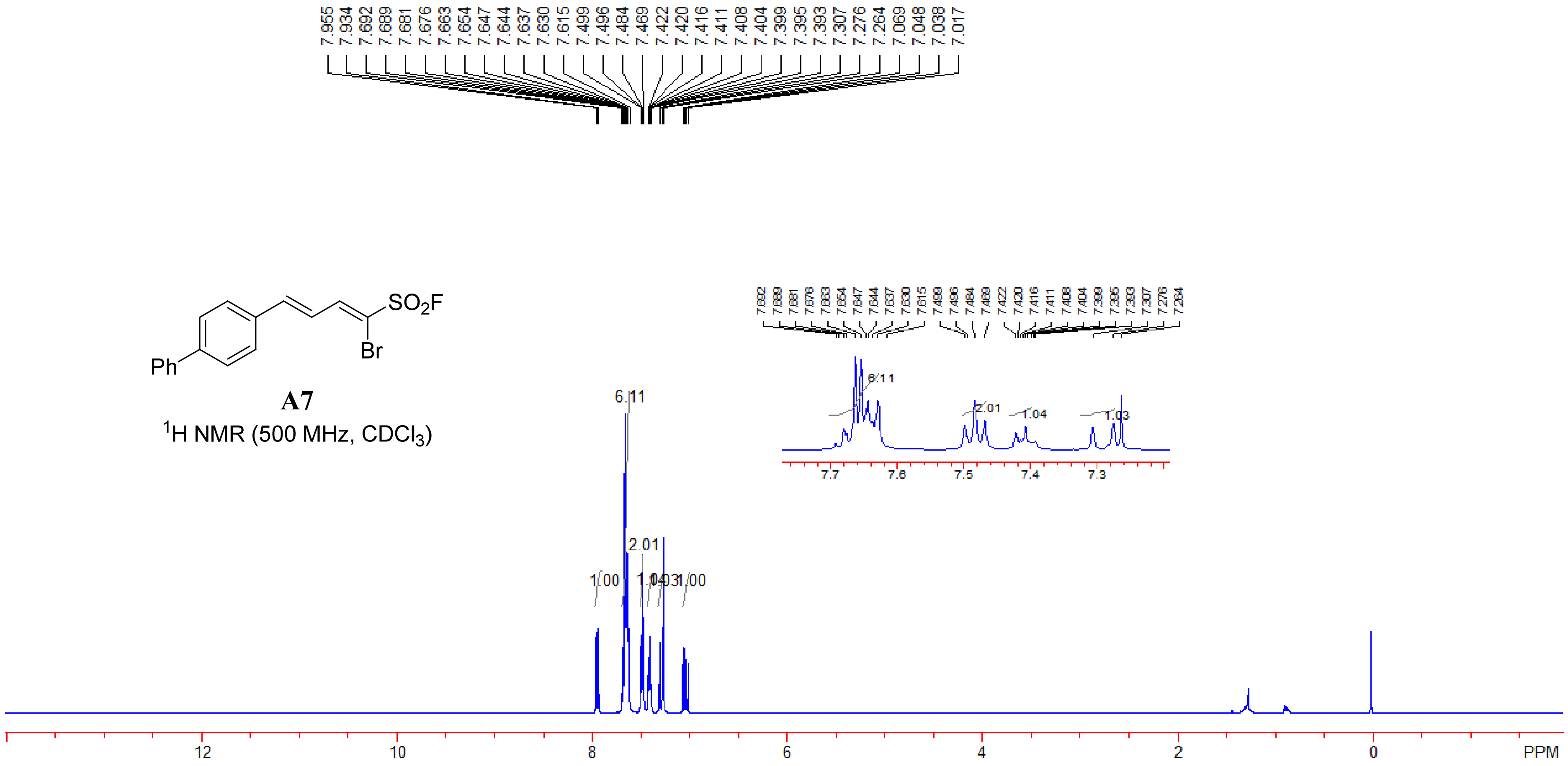

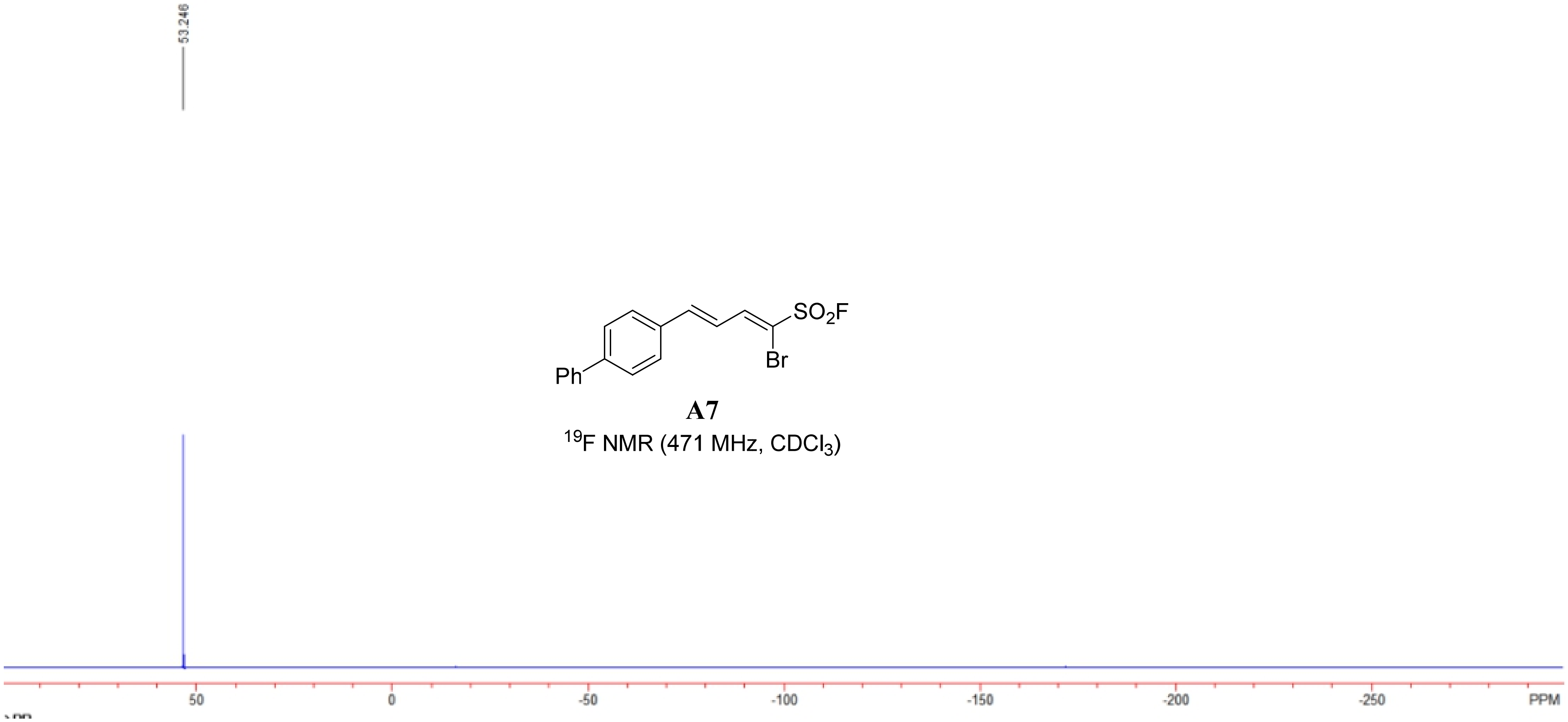

A7

^{19}F NMR (471 MHz, $CDCl_3$)

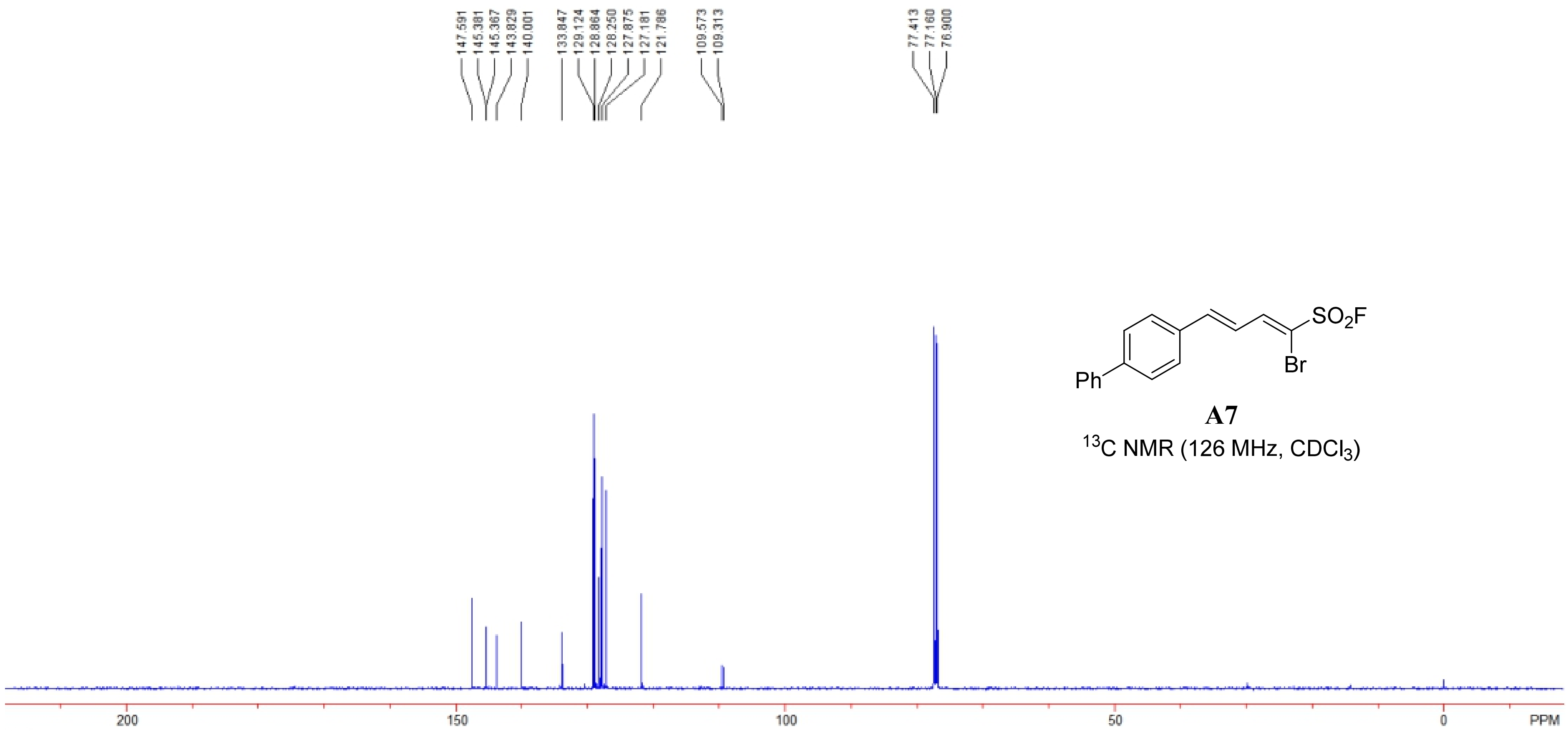

A7

^{13}C NMR (126 MHz, $CDCl_3$)

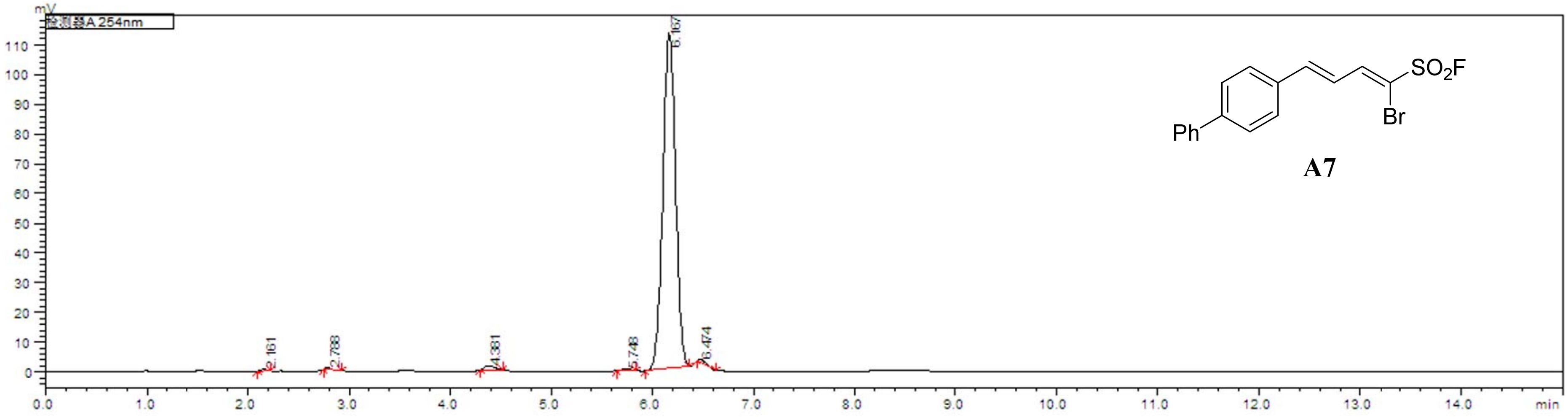

A7

No.	Ret Time (min)	Area (mAU*min)	Rel.Area (%)
1	2.161	2622	0.26%
2	2.788	2027	0.20%
3	4.381	10016	0.99%
4	5.748	3789	0.38%
5	6.167	986201	97.77%
6	6.474	4082	0.40%
Total		1008736	

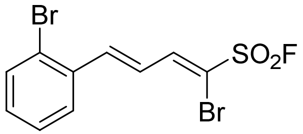

A8

^{1}H NMR (500 MHz, $CDCl_3$)

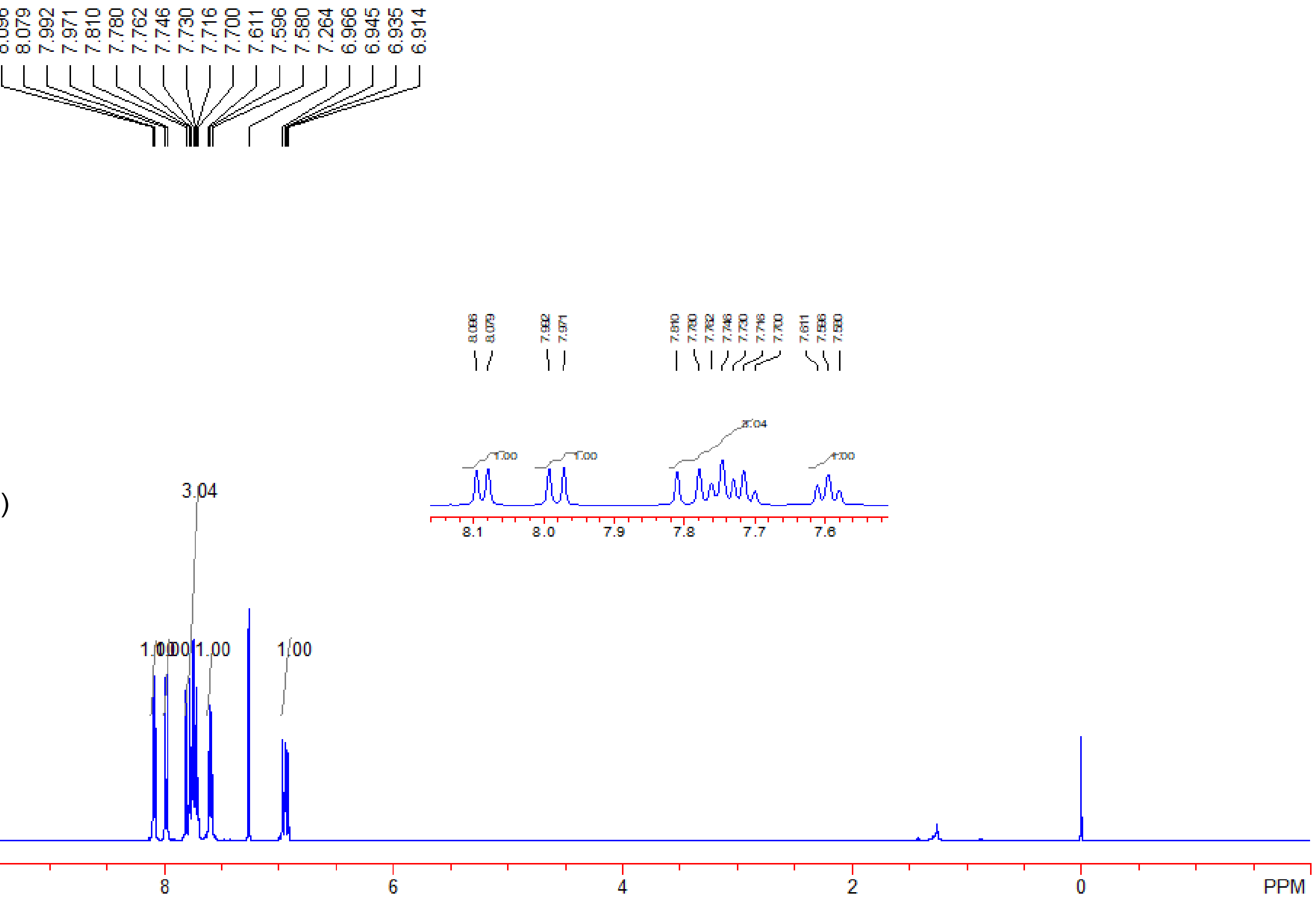

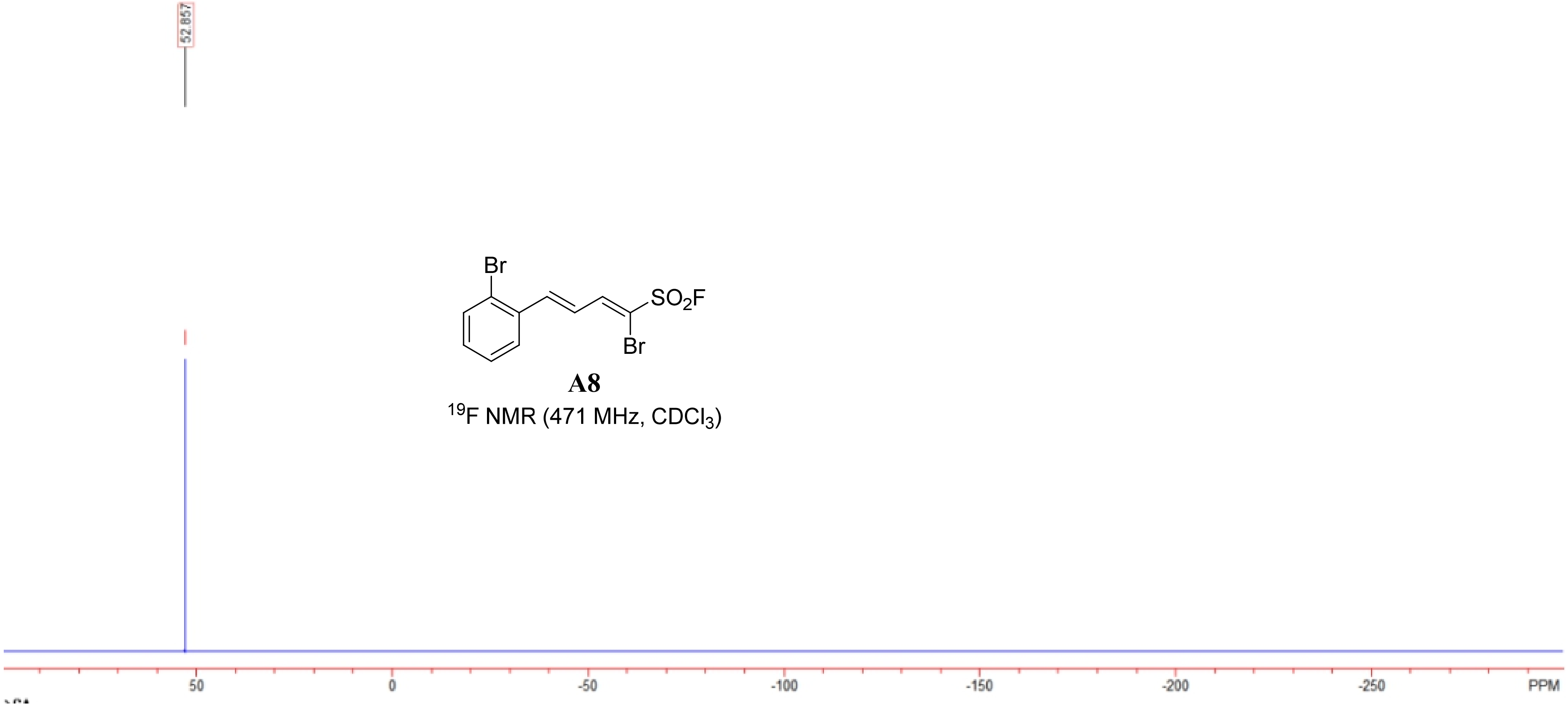

A8

^{19}F NMR (471 MHz, $CDCl_3$)

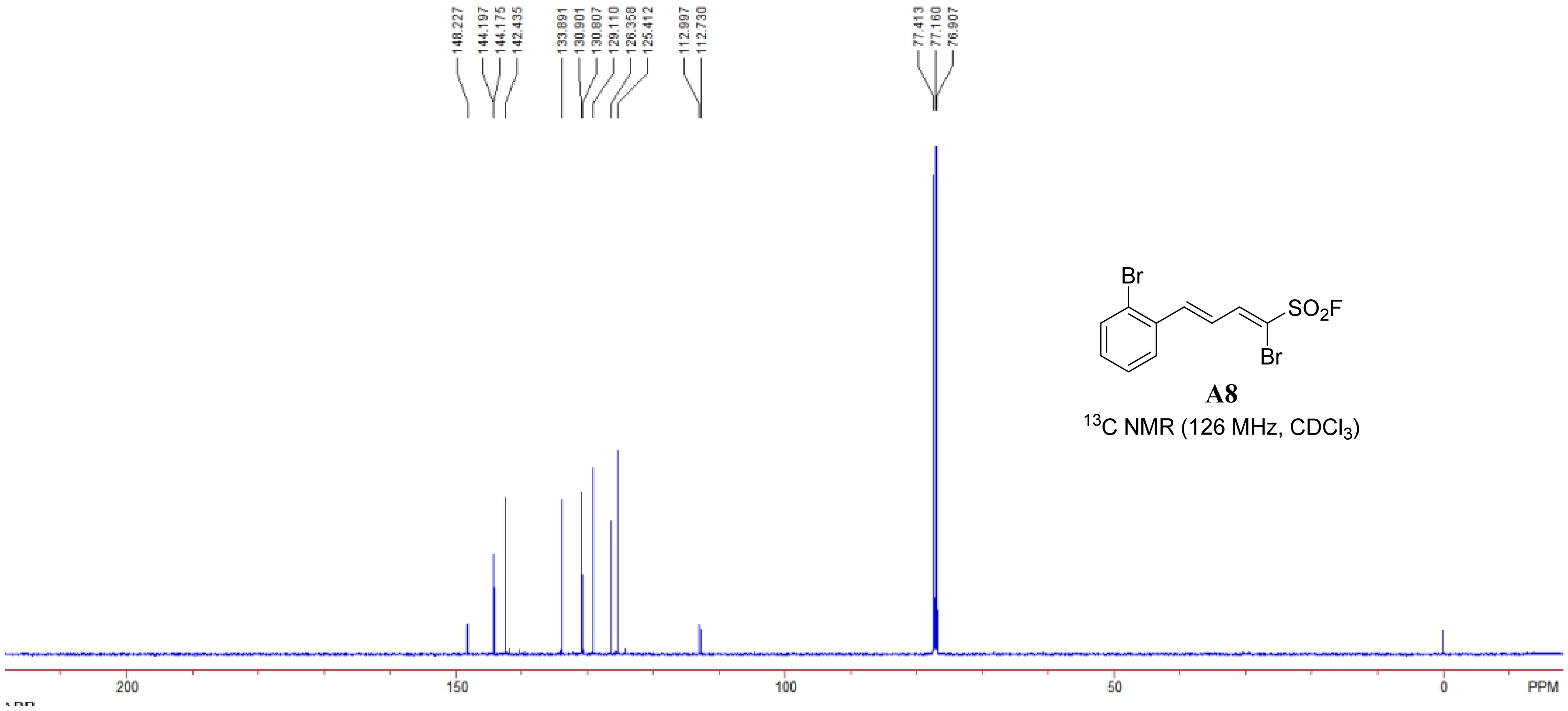

A8

^{13}C NMR (126 MHz, $CDCl_3$)

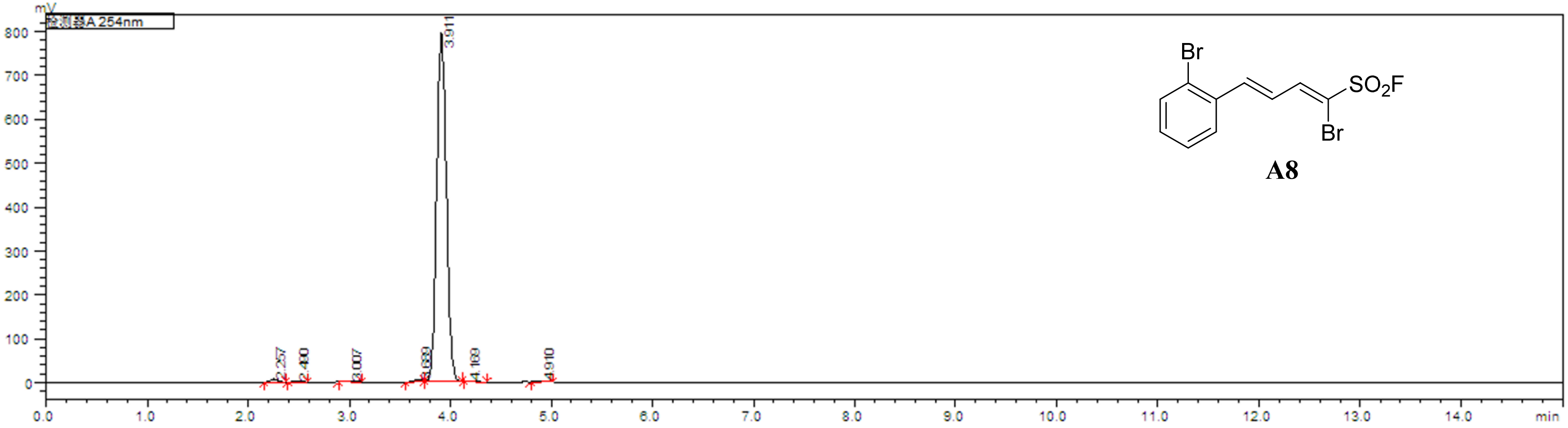

No.	Ret Time (min)	Area (mAU*min)	Rel.Area (%)
1	2.257	36162	0.68%
2	2.490	20924	0.39%
3	3.007	16454	0.31%
4	3.689	16931	0.32%
5	3.911	5213366	97.91%
6	4.169	2247	0.04%
7	4.910	18425	0.35%
Total		5324508	

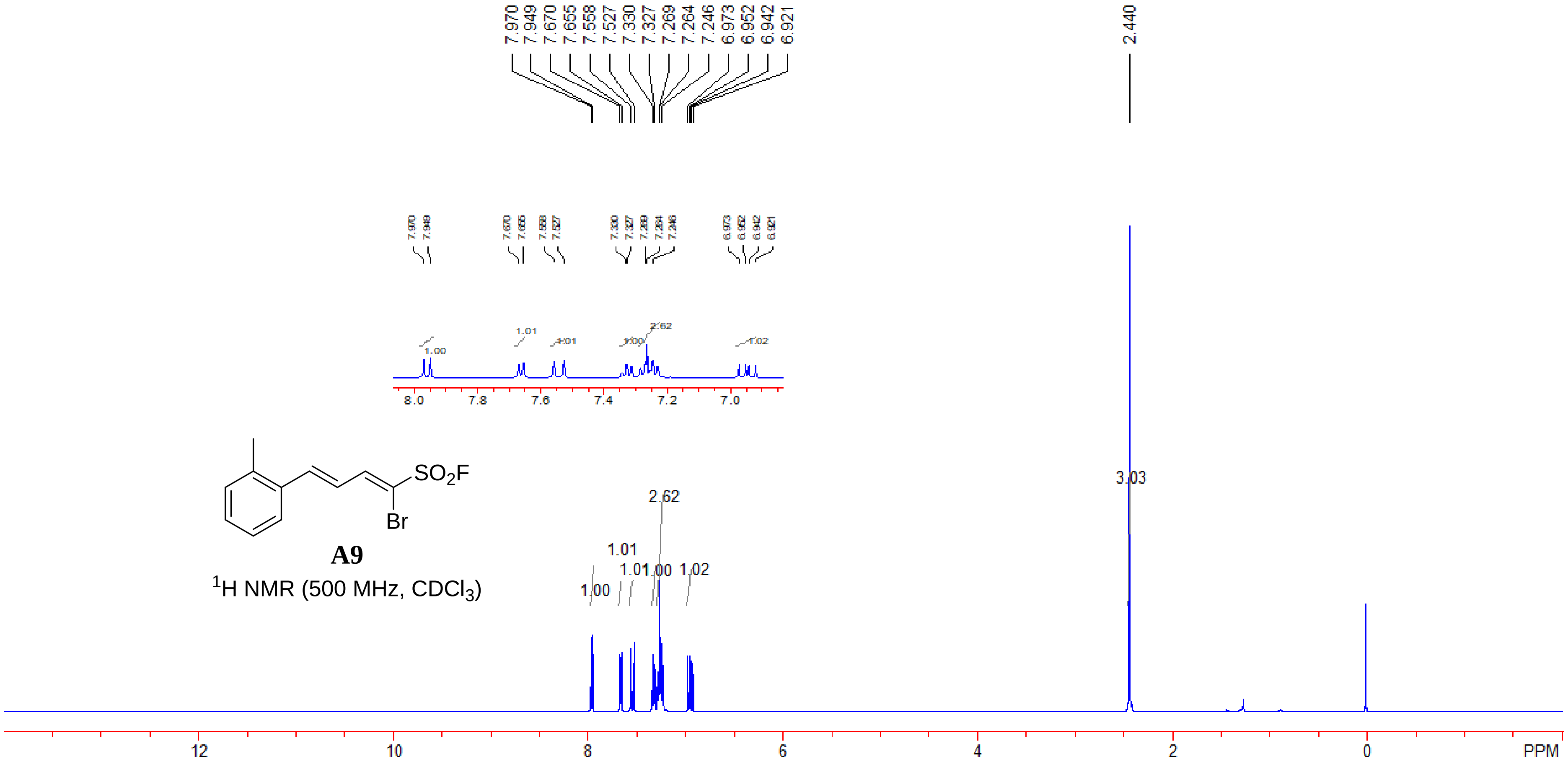

A9

^{1}H NMR (500 MHz, $CDCl_3$)

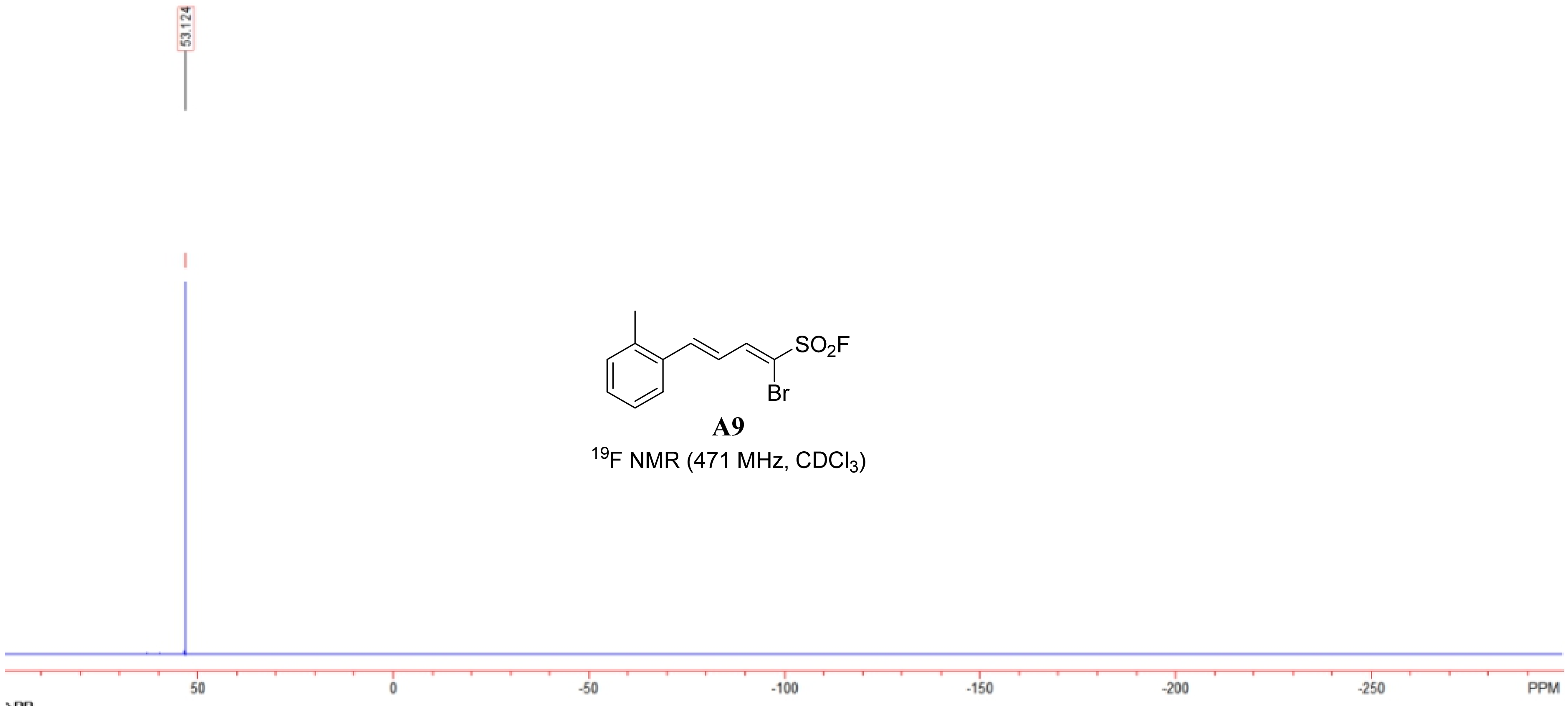

A9

^{19}F NMR (471 MHz, $CDCl_3$)

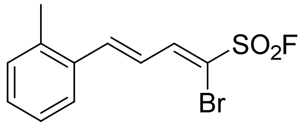

A9

^{13}C NMR (126 MHz, $CDCl_3$)

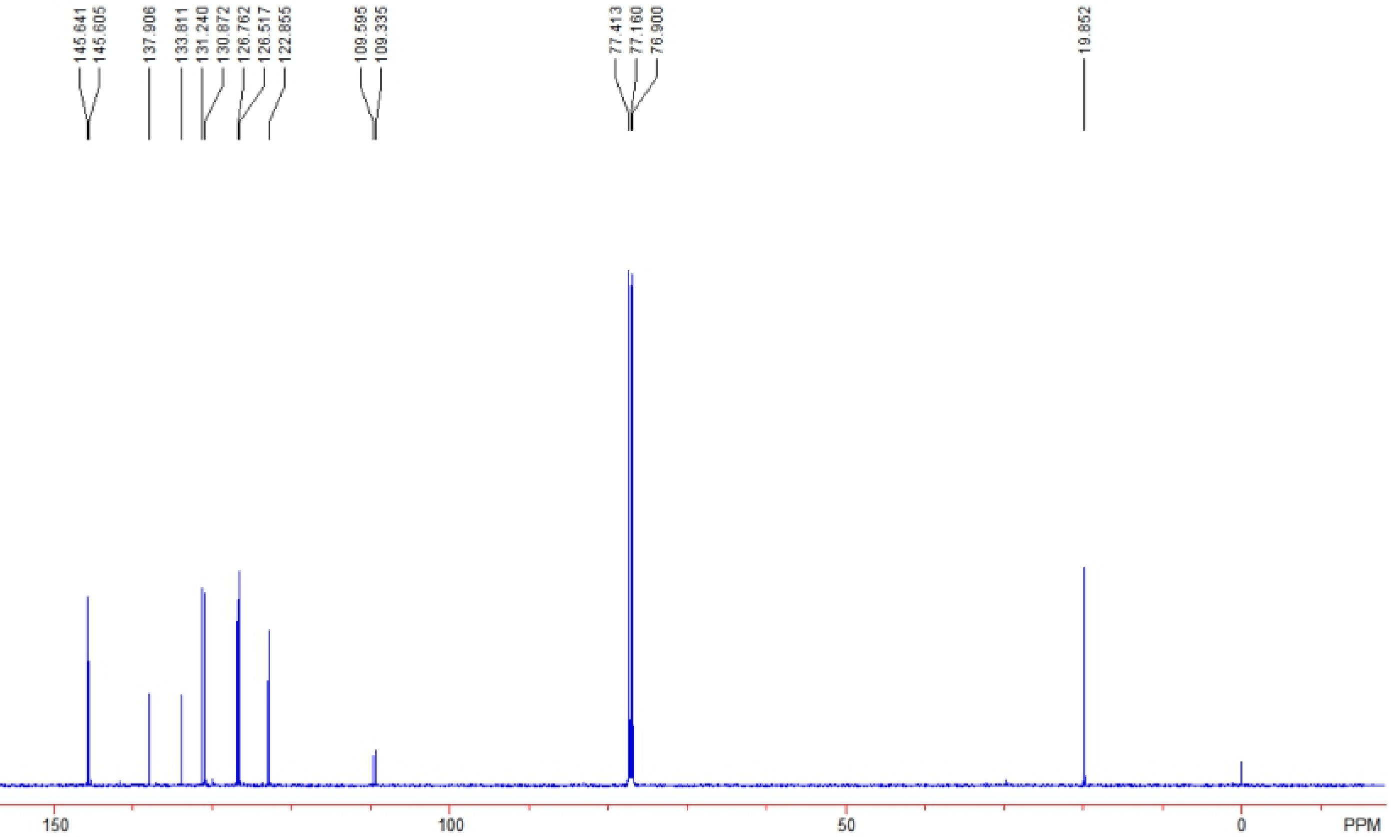

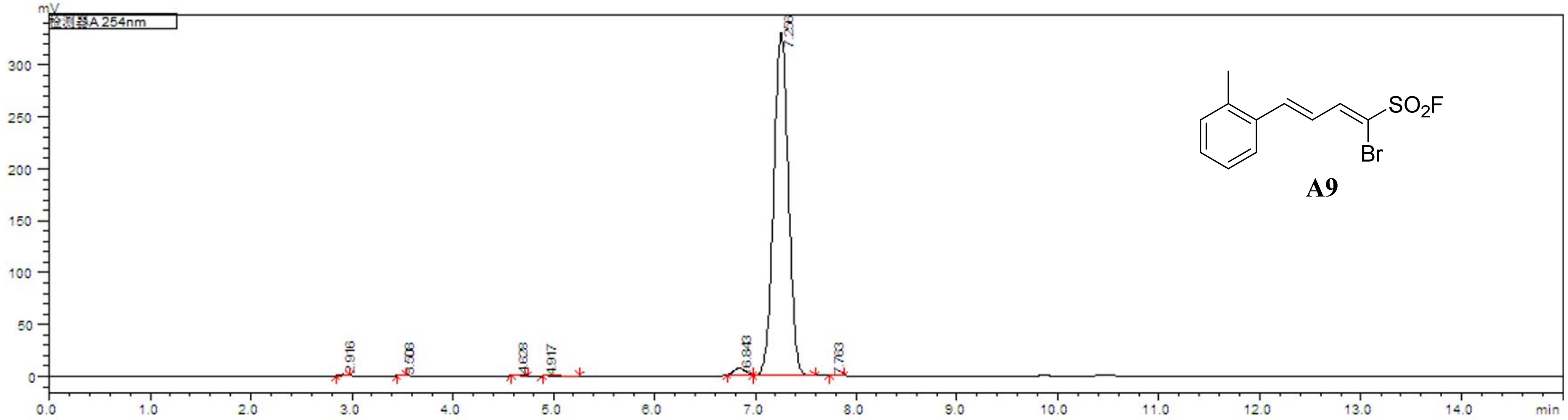

No.	Ret Time (min)	Area (mAU*min)	Rel.Area (%)
1	2.916	6525	0.19%
2	3.508	833	0.02%
3	4.628	1878	0.06%
4	4.917	239	0.01%
5	6.843	49747	1.46%
6	7.256	3349157	98.24%
7	7.763	1174	0.03%
Total		3409075	

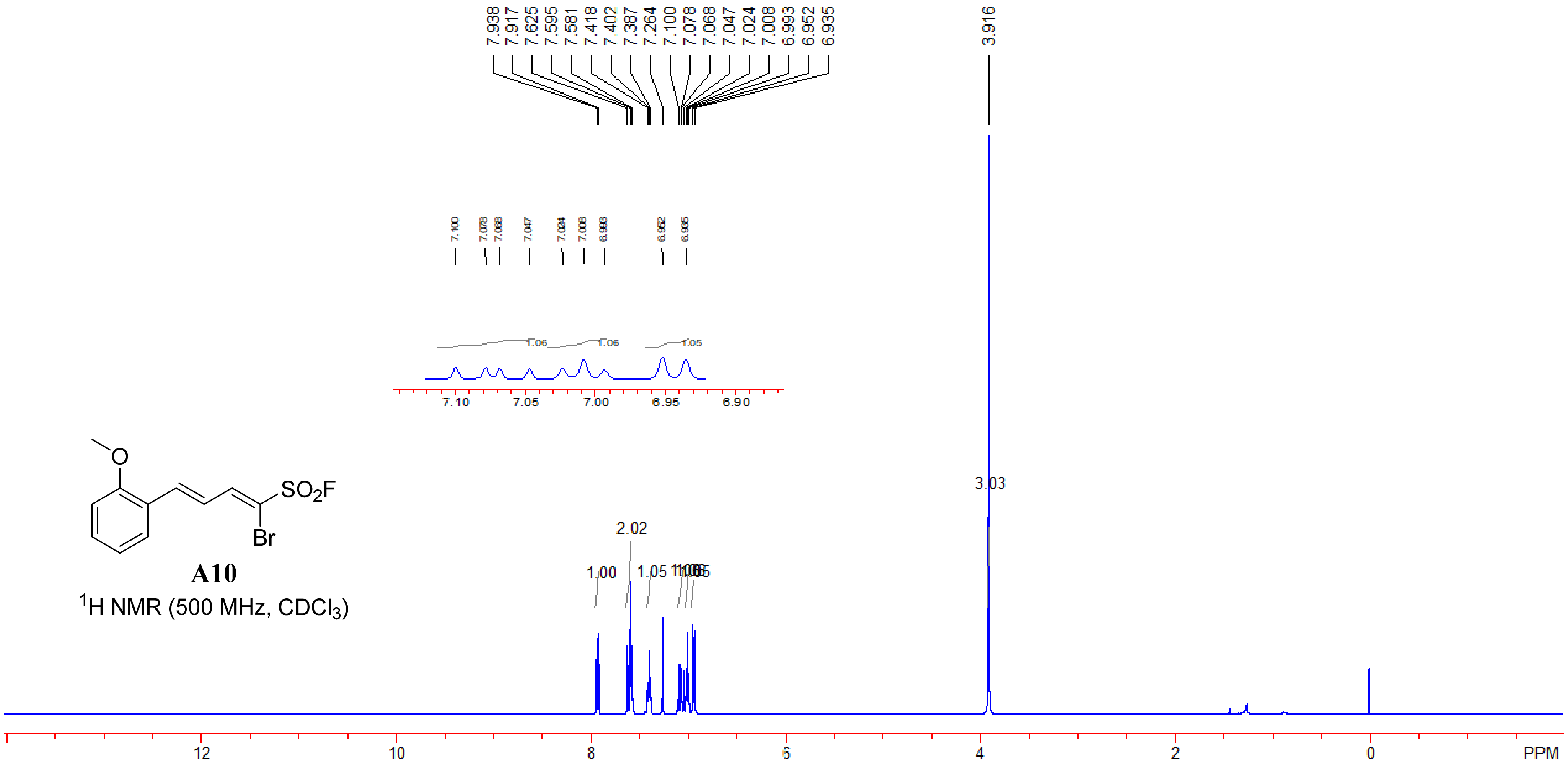

A10

^{1}H NMR (500 MHz, $CDCl_3$)

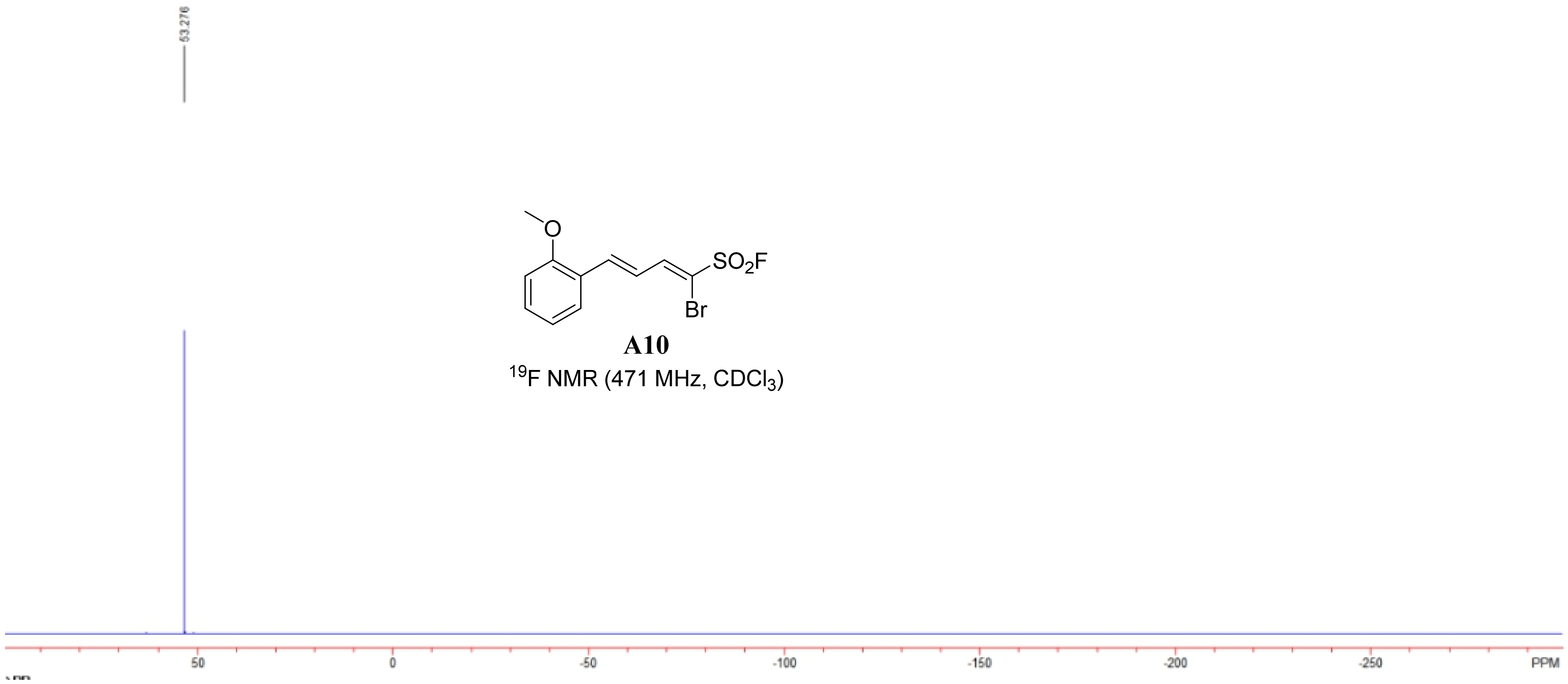

A10

^{19}F NMR (471 MHz, $CDCl_3$)

A10

^{13}C NMR (126 MHz, $CDCl_3$)

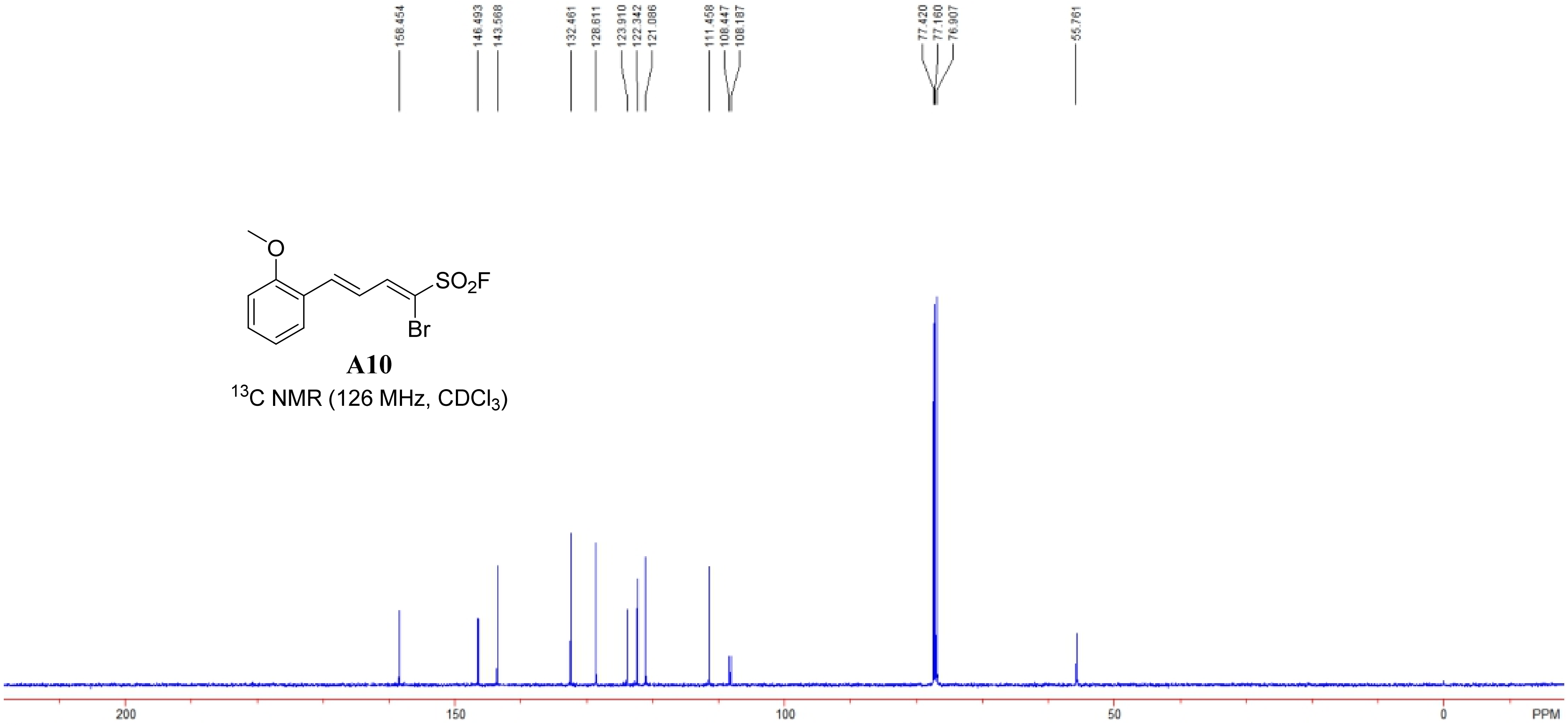

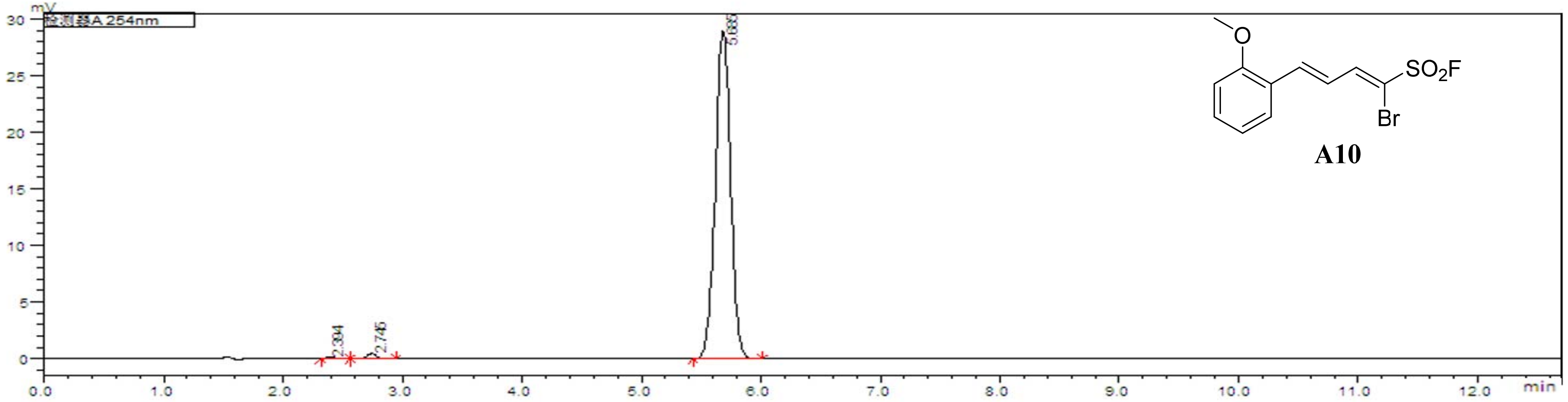

A10

No.	Ret Time (min)	Area (mAU*min)	Rel.Area (%)
1	2.394	1068	0.41%
2	2.745	2708	1.03%
3	5.685	258159	98.56%
Total		261935	

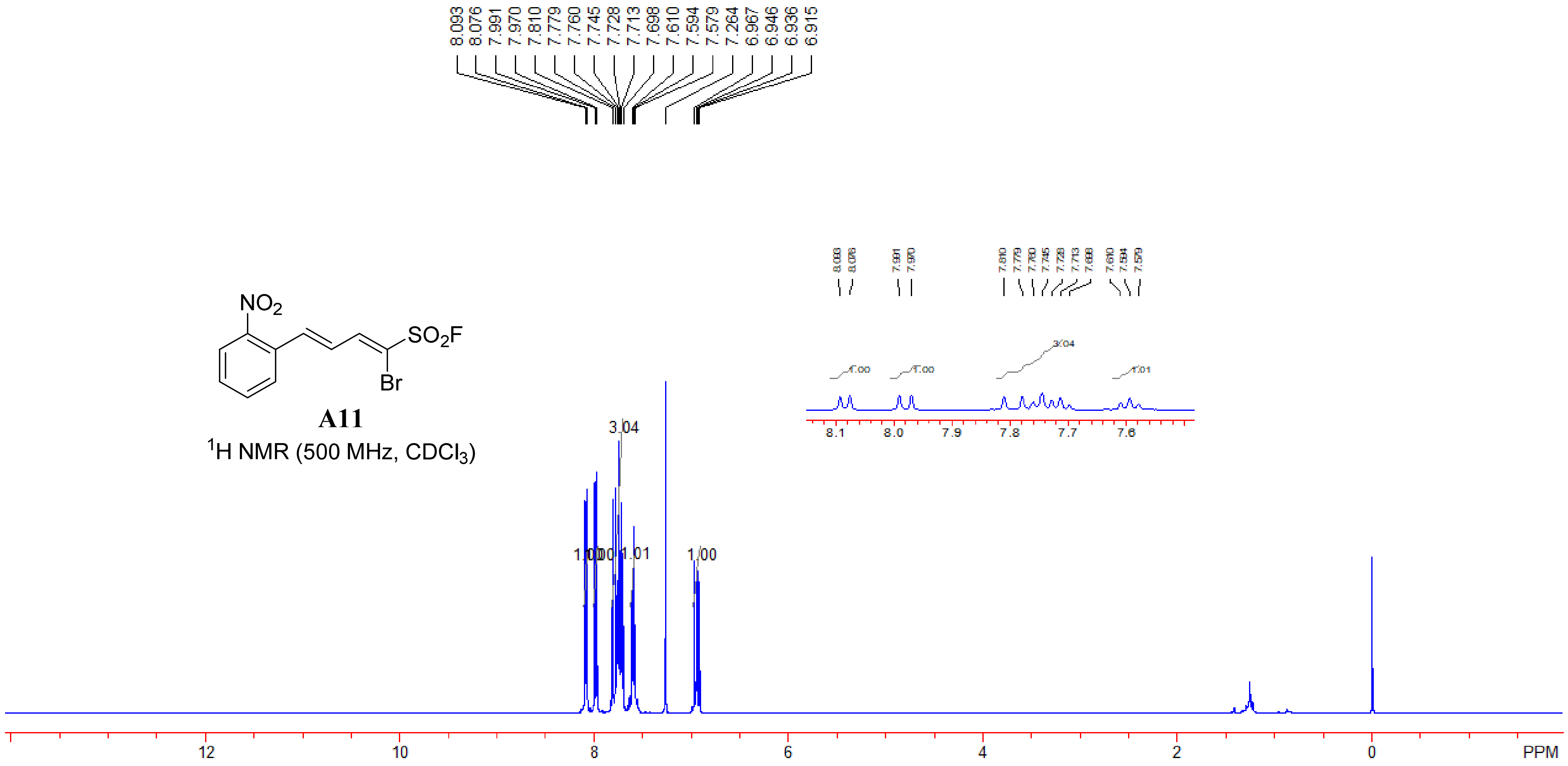

A11

^{1}H NMR (500 MHz, $CDCl_3$)

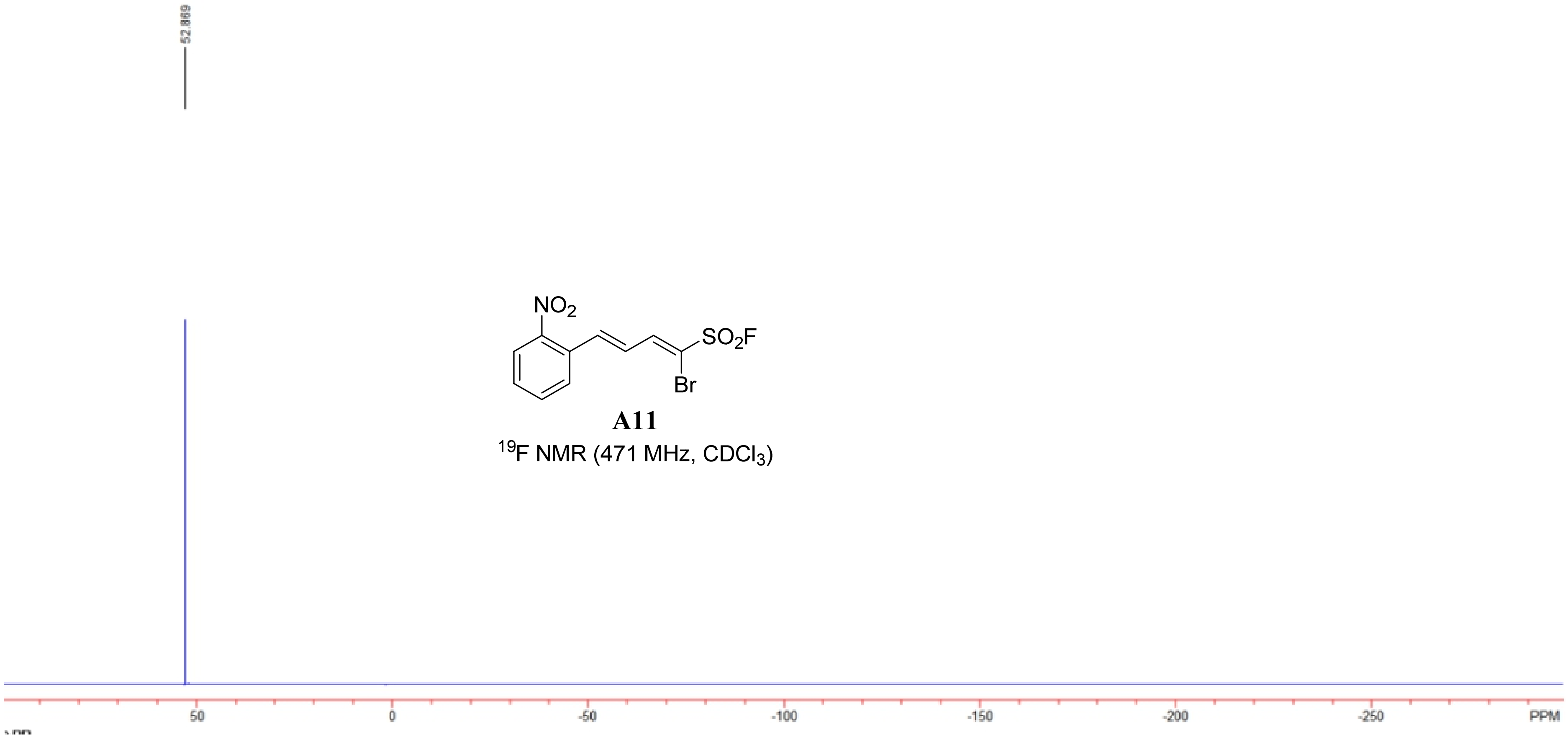

A11

^{19}F NMR (471 MHz, $CDCl_3$)

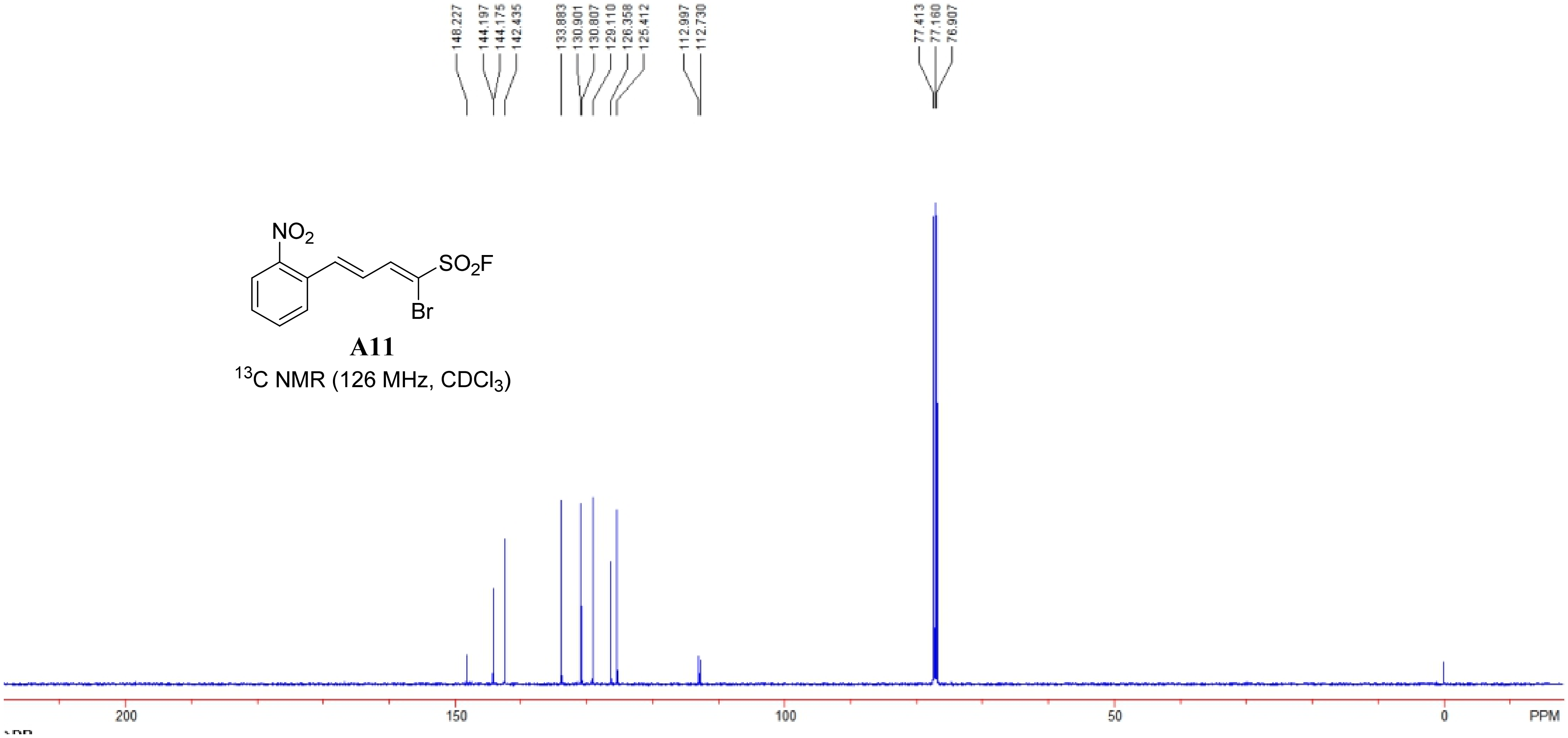

A11

^{13}C NMR (126 MHz, $CDCl_3$)

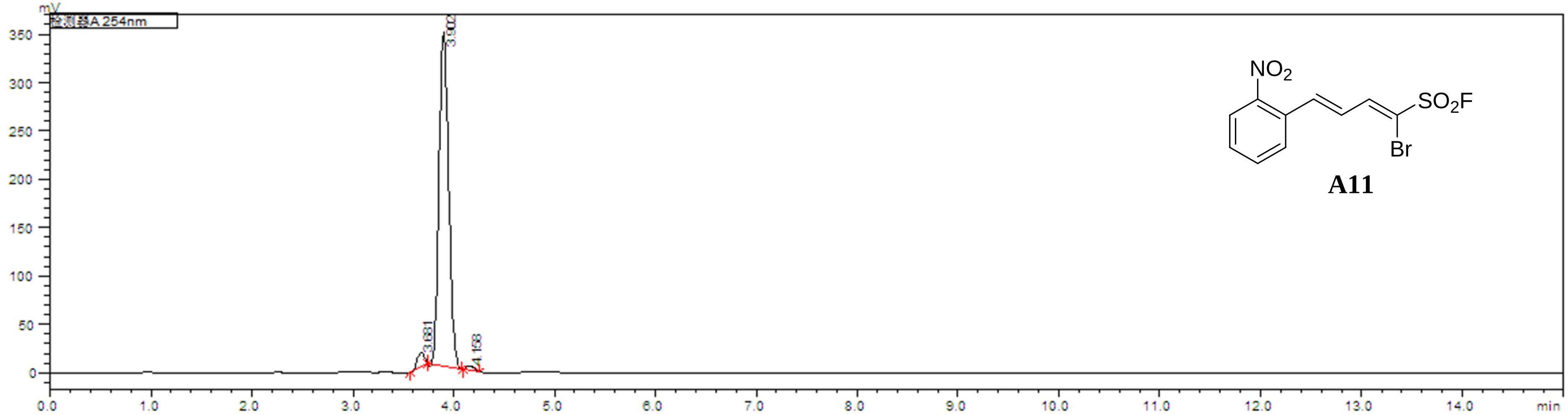

A11

No.	Ret Time (min)	Area (mAU*min)	Rel.Area (%)
1	3.681	75621	3.19%
2	3.902	2274965	95.89%
3	4.158	22000	0.93%
Total		2372586	

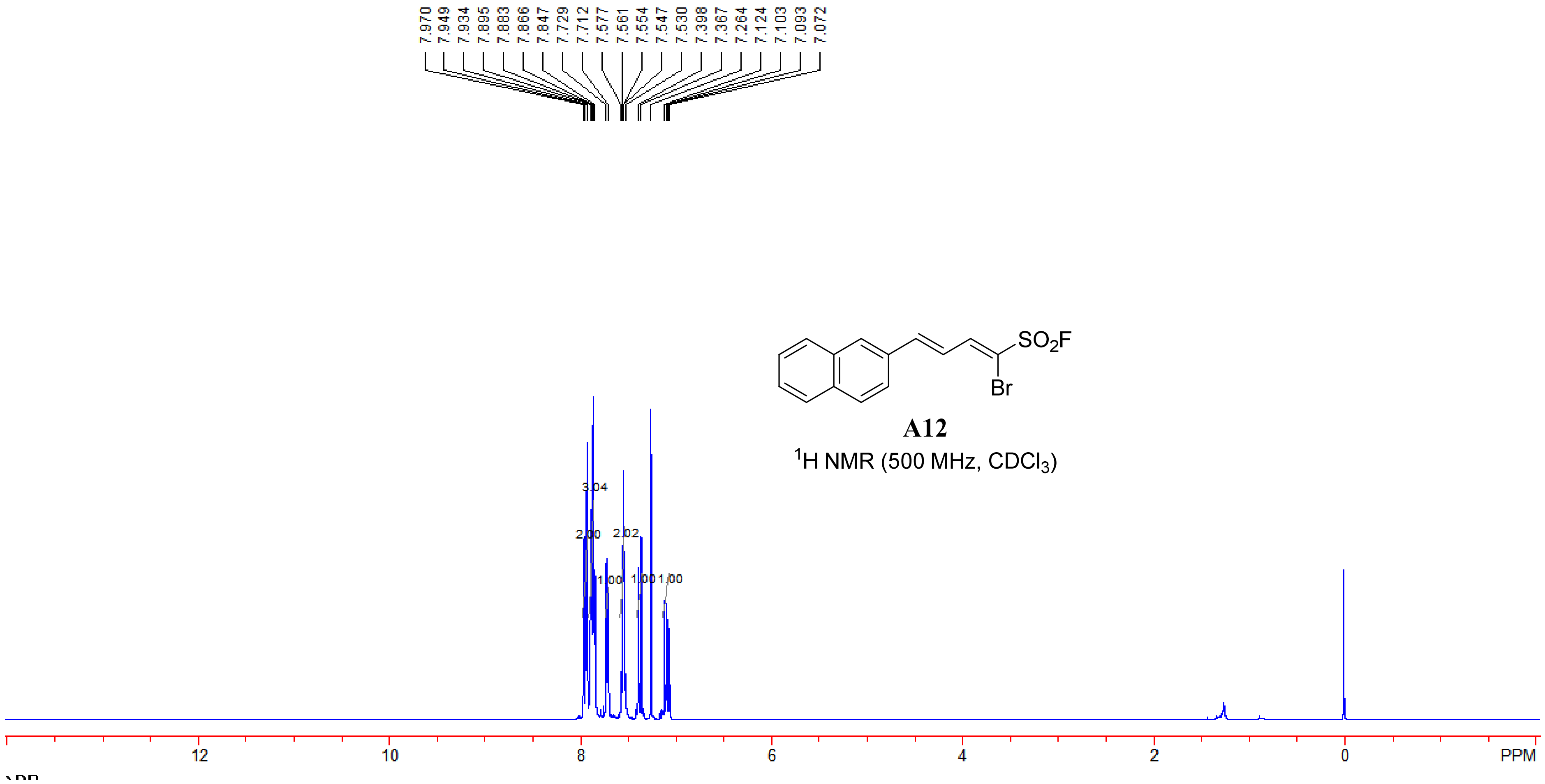
7.970
7.949
7.934
7.895
7.883
7.866
7.847
7.729
7.712
7.577
7.561
7.554
7.547
7.530
7.398
7.367
7.264
7.124
7.103
7.093
7.072
SO2F
Br
A12
1H NMR (500 MHz, CDCl3)
3.04
2.00
2.02
1.00
1.00
1.00
12
10
8
6
4
2
0
PPM

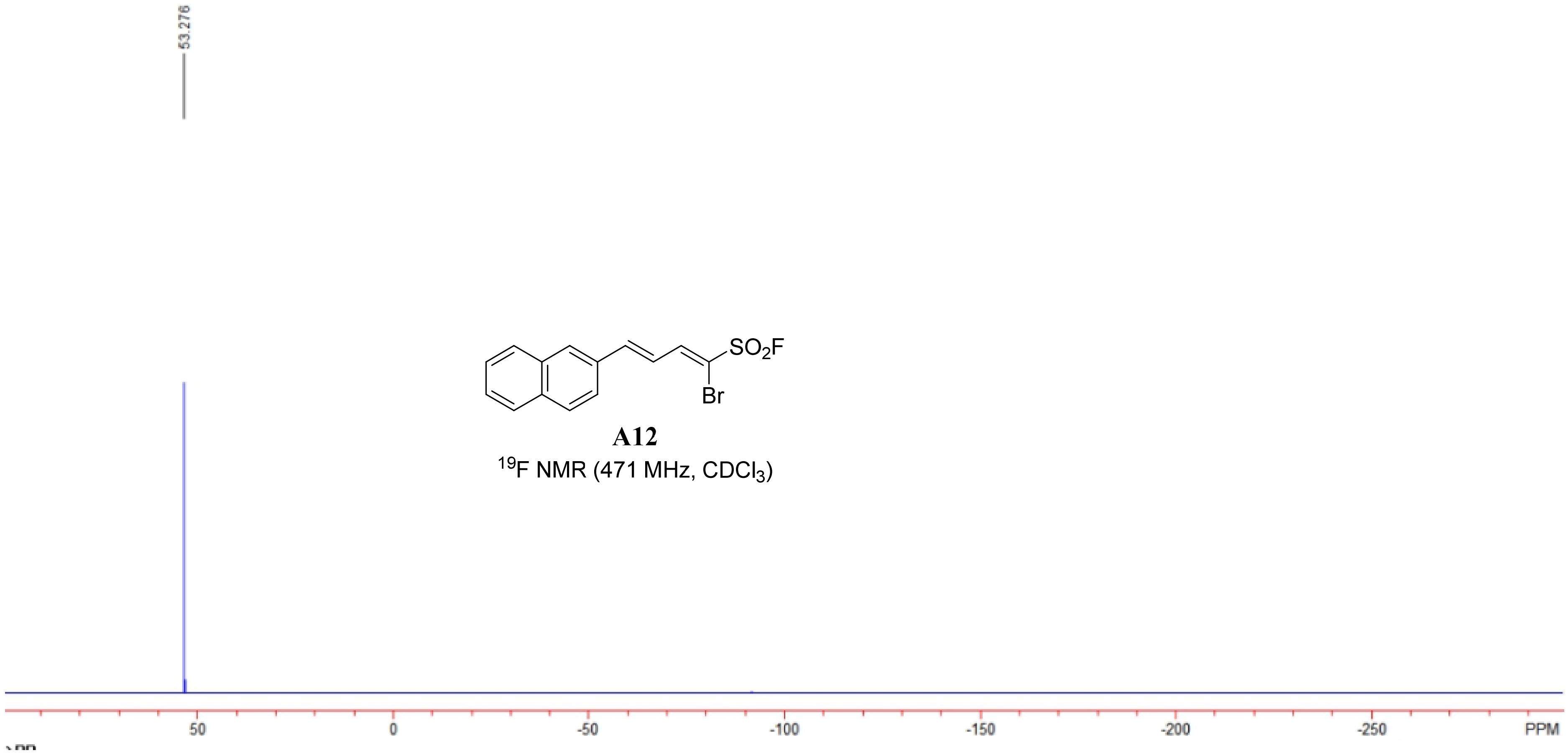

A12

^{19}F NMR (471 MHz, $CDCl_3$)

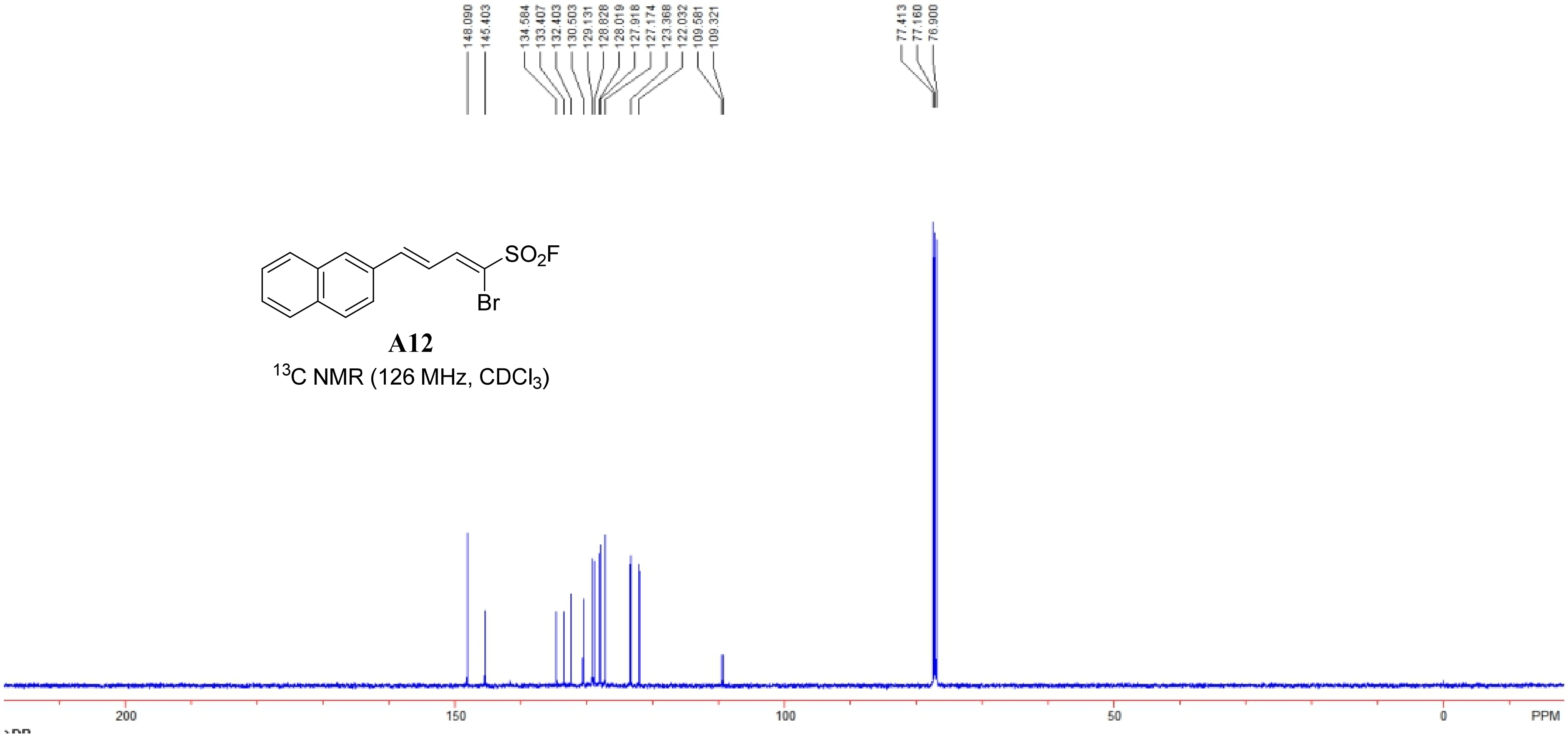

A12

^{13}C NMR (126 MHz, $CDCl_3$)

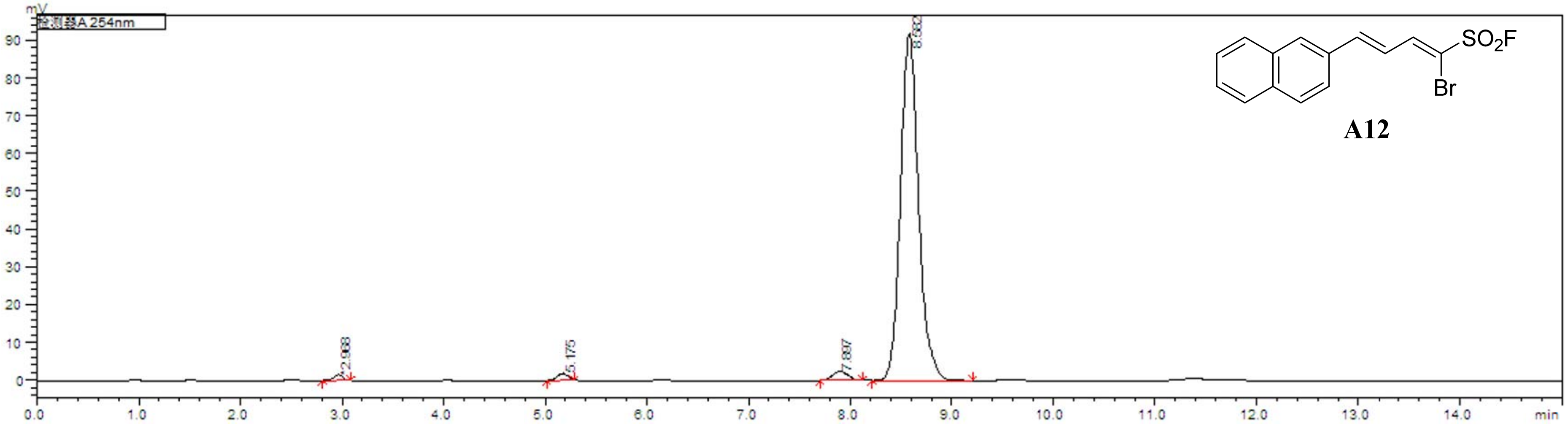

No.	Ret Time (min)	Area (mAU*min)	Rel.Area (%)
1	2.968	9368	0.80%
2	5.175	12581	1.07%
3	7.897	24809	2.11%
4	8.582	1131206	96.03%
Total		1177965	

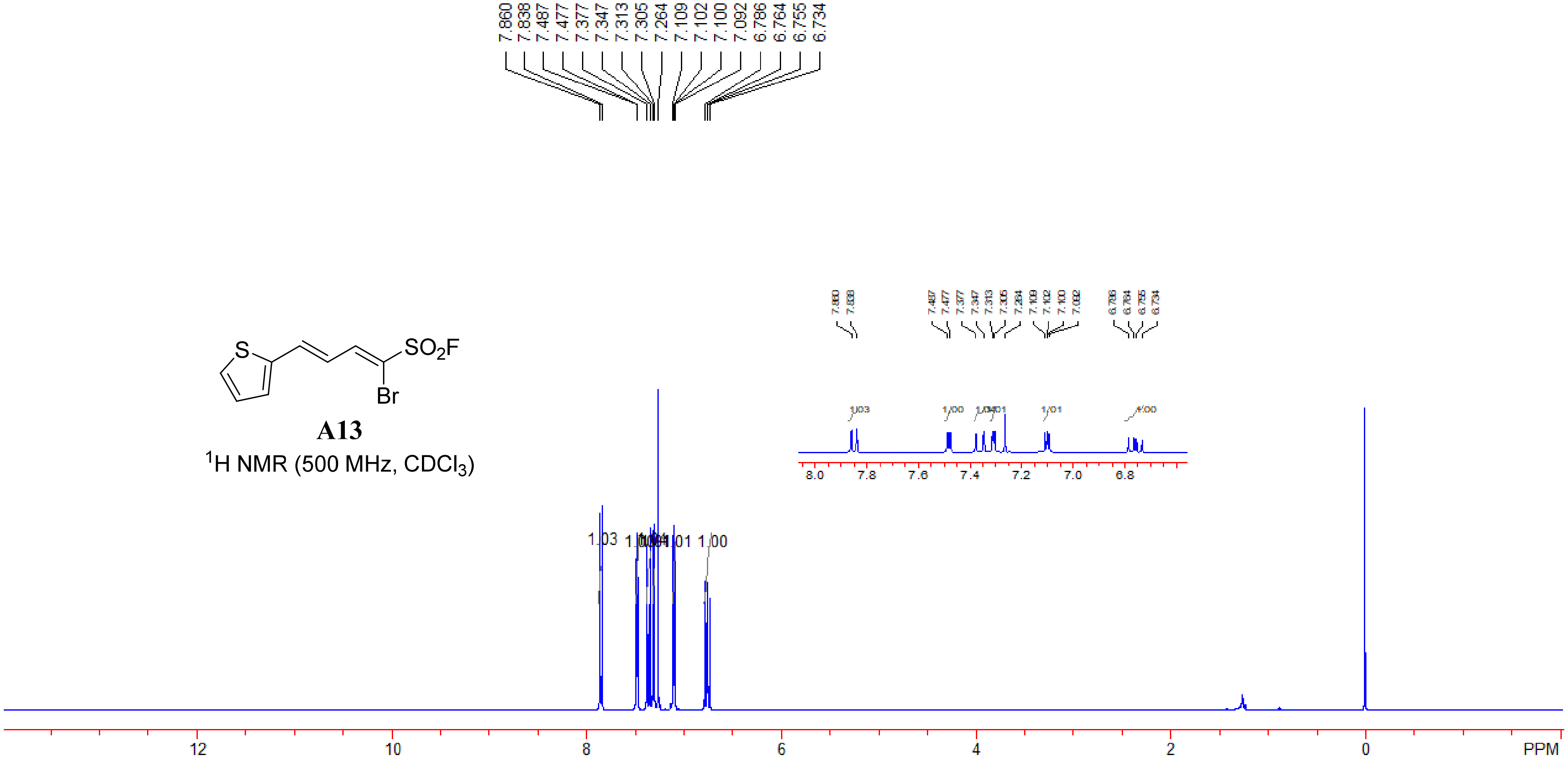

A13

^1H NMR (500 MHz, $CDCl_3$)

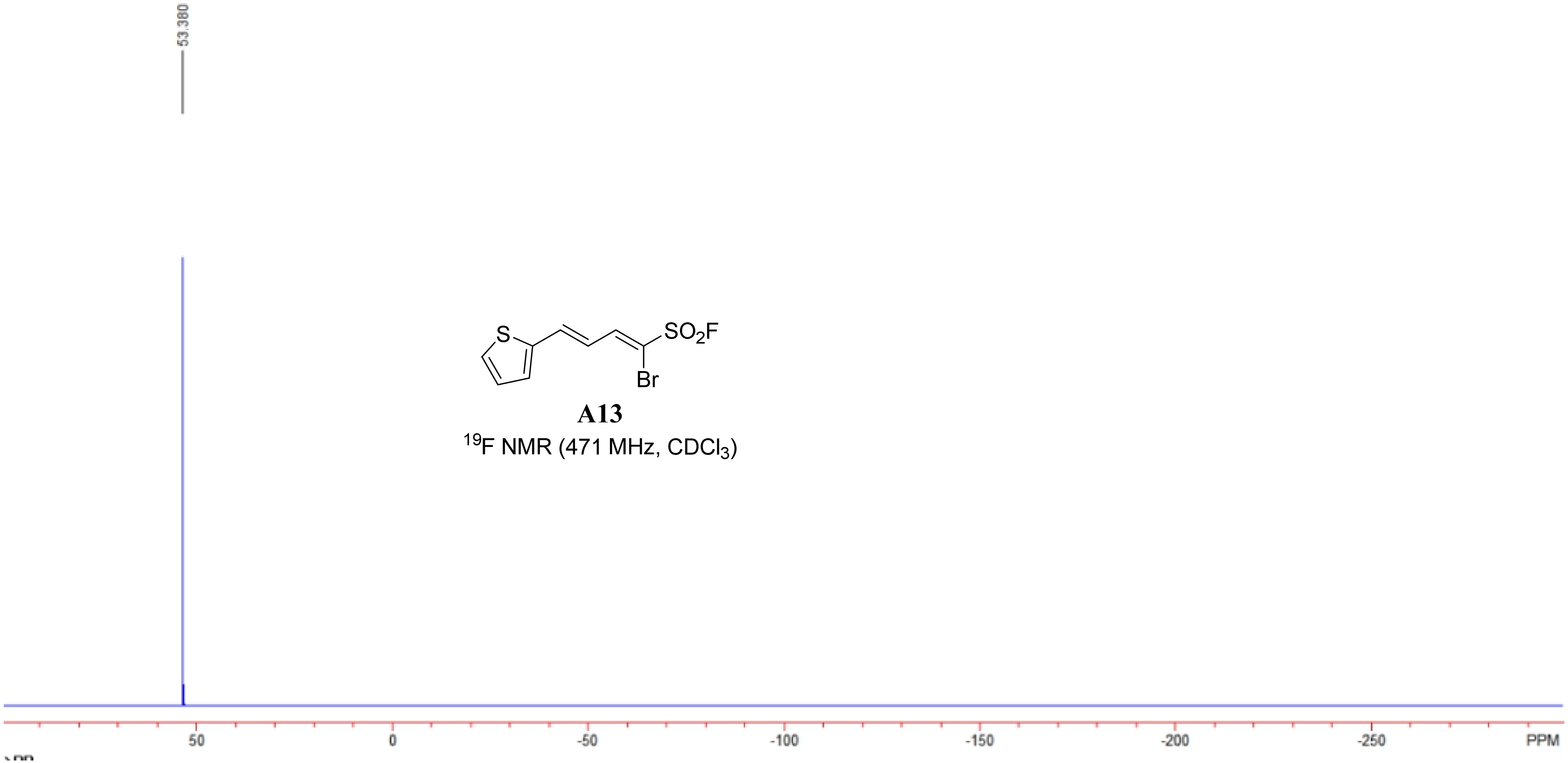

A13

^{19}F NMR (471 MHz, $CDCl_3$)

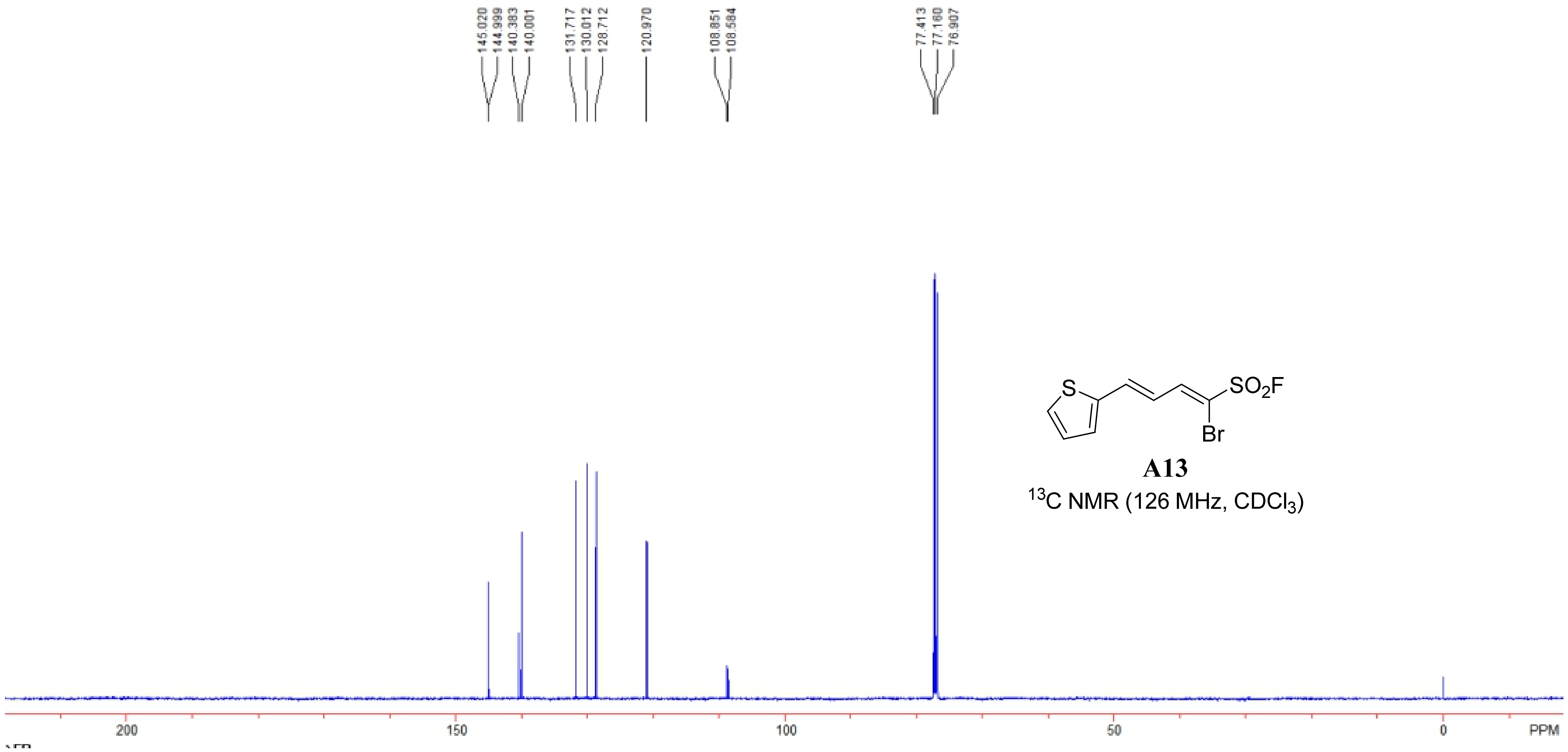

A13

^{13}C NMR (126 MHz, $CDCl_3$)

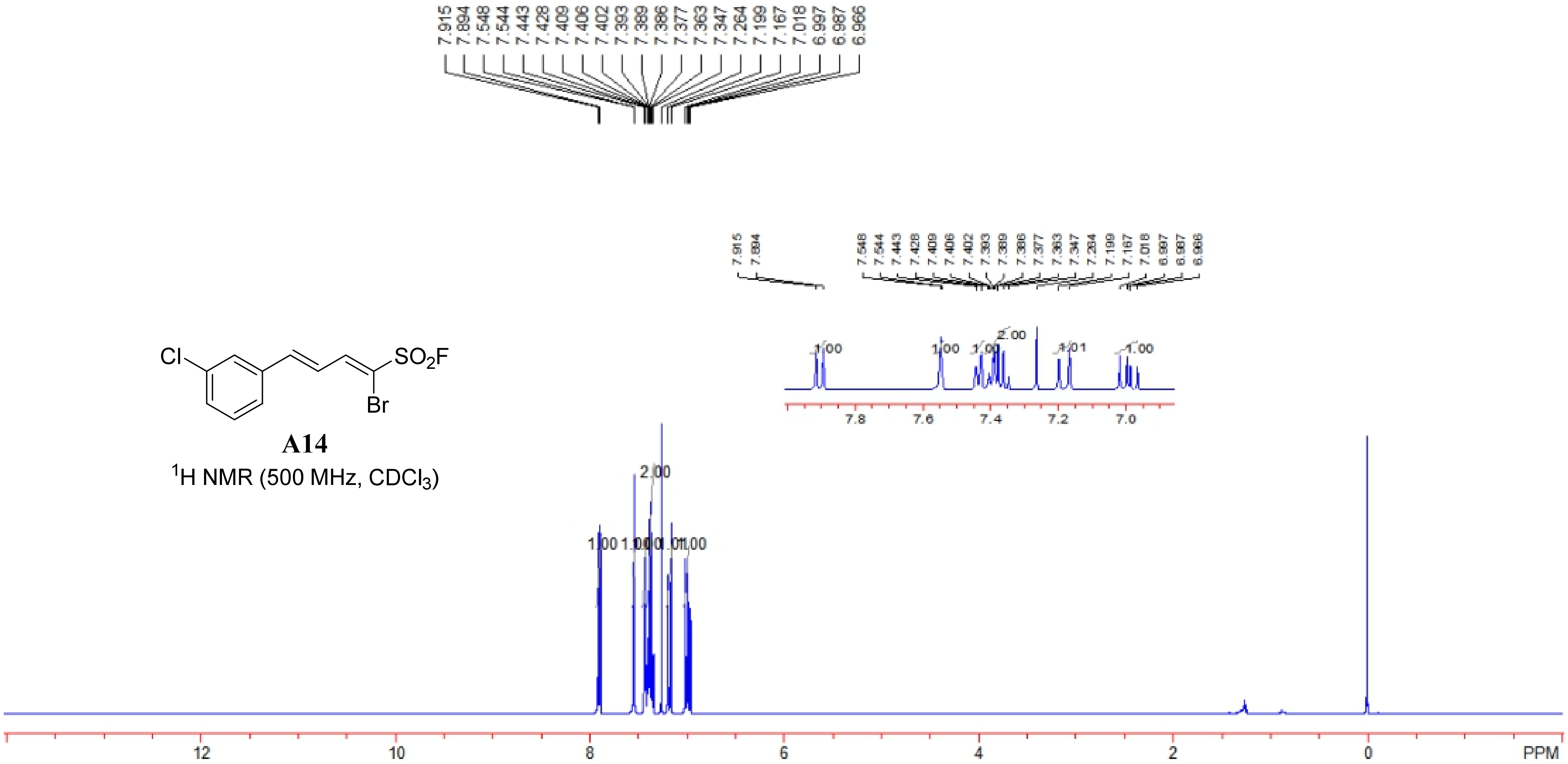

A14

^{1}H NMR (500 MHz, $CDCl_3$)

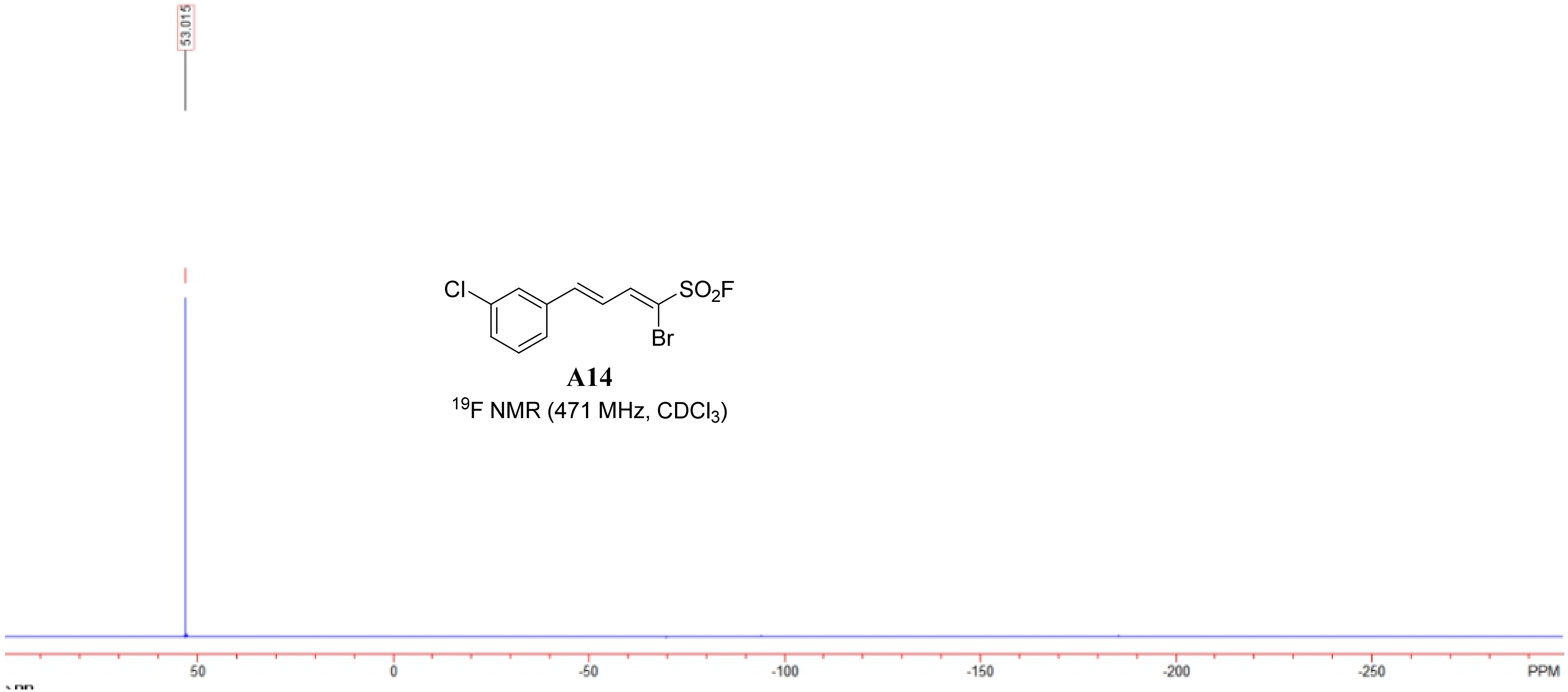

A14

^{19}F NMR (471 MHz, $CDCl_3$)

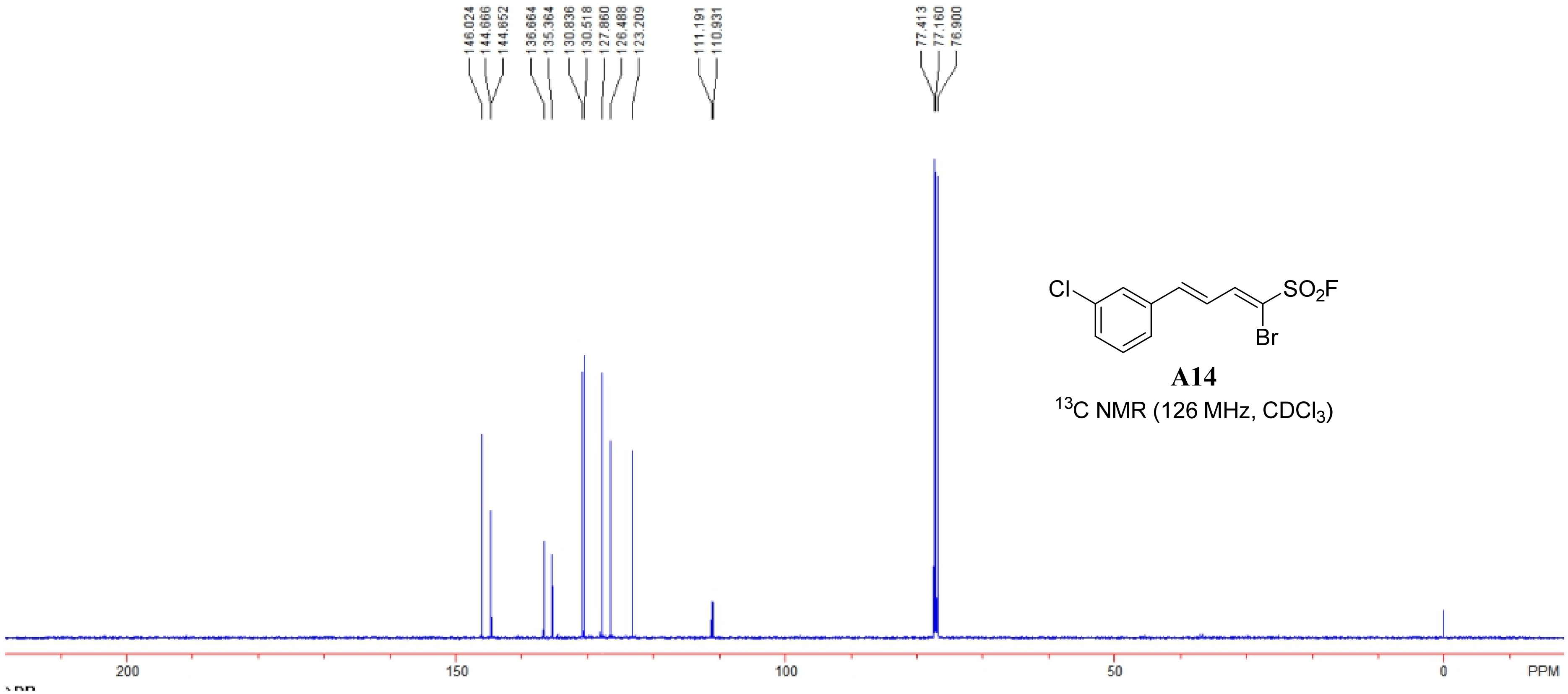

A14

^{13}C NMR (126 MHz, $CDCl_3$)

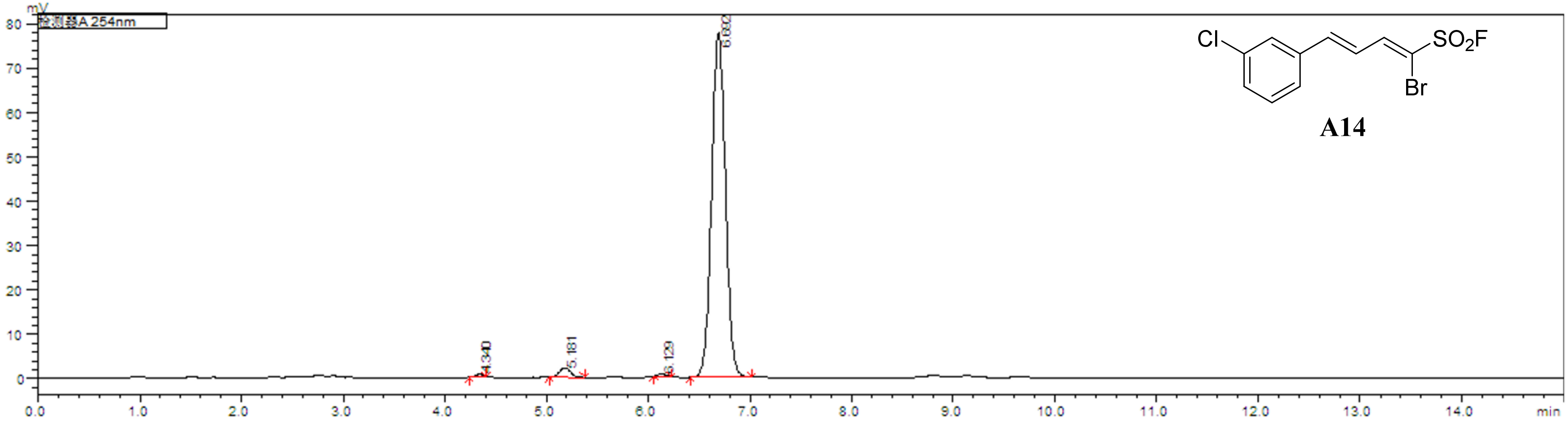

A14

No.	Ret Time (min)	Area (mAU*min)	Rel.Area (%)
1	4.340	3035	0.40%
2	5.181	16230	2.15%
3	6.129	2722	0.36%
4	6.692	732288	97.09%
Total		754274	

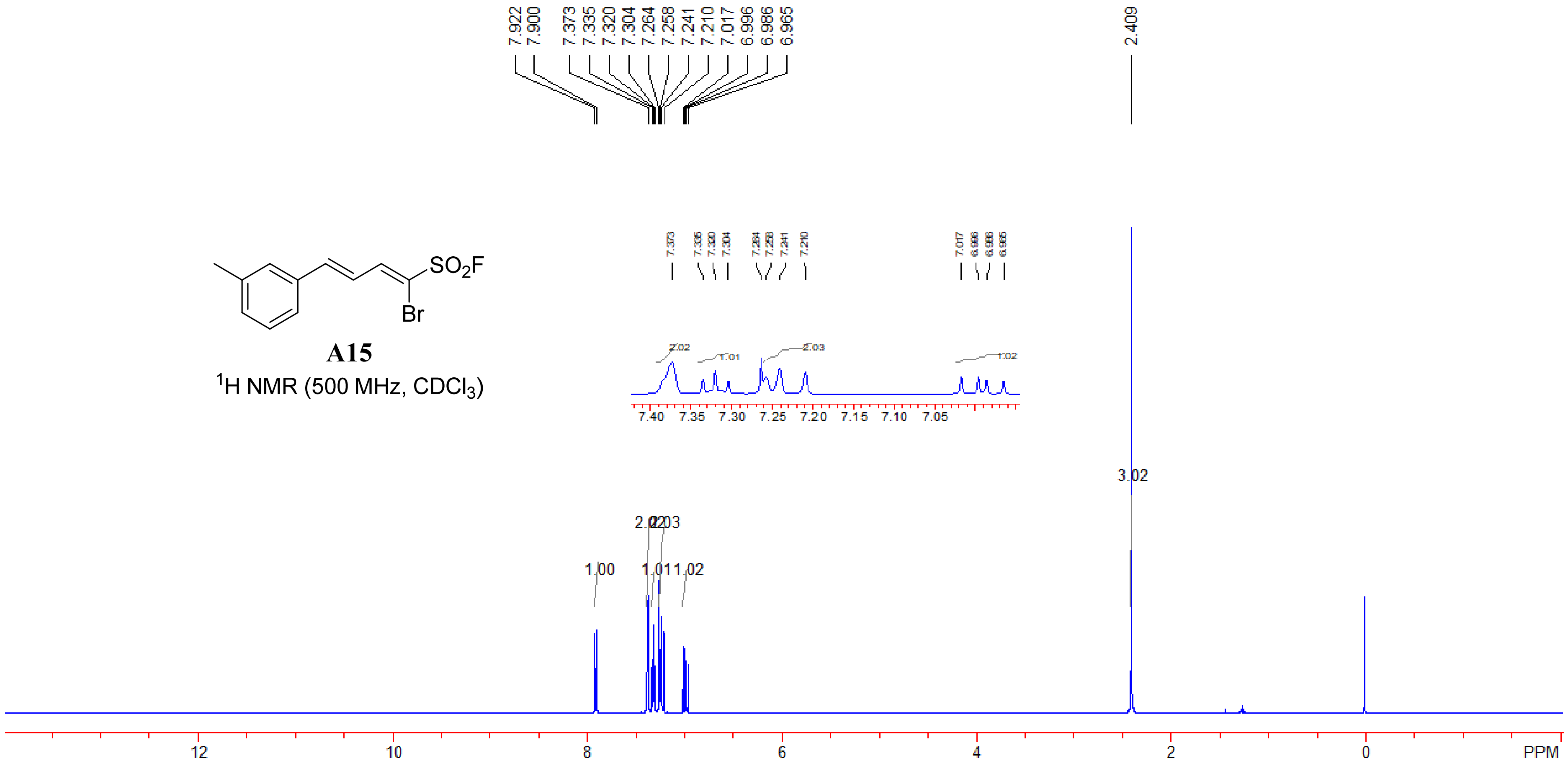

A15

^{1}H NMR (500 MHz, $CDCl_3$)

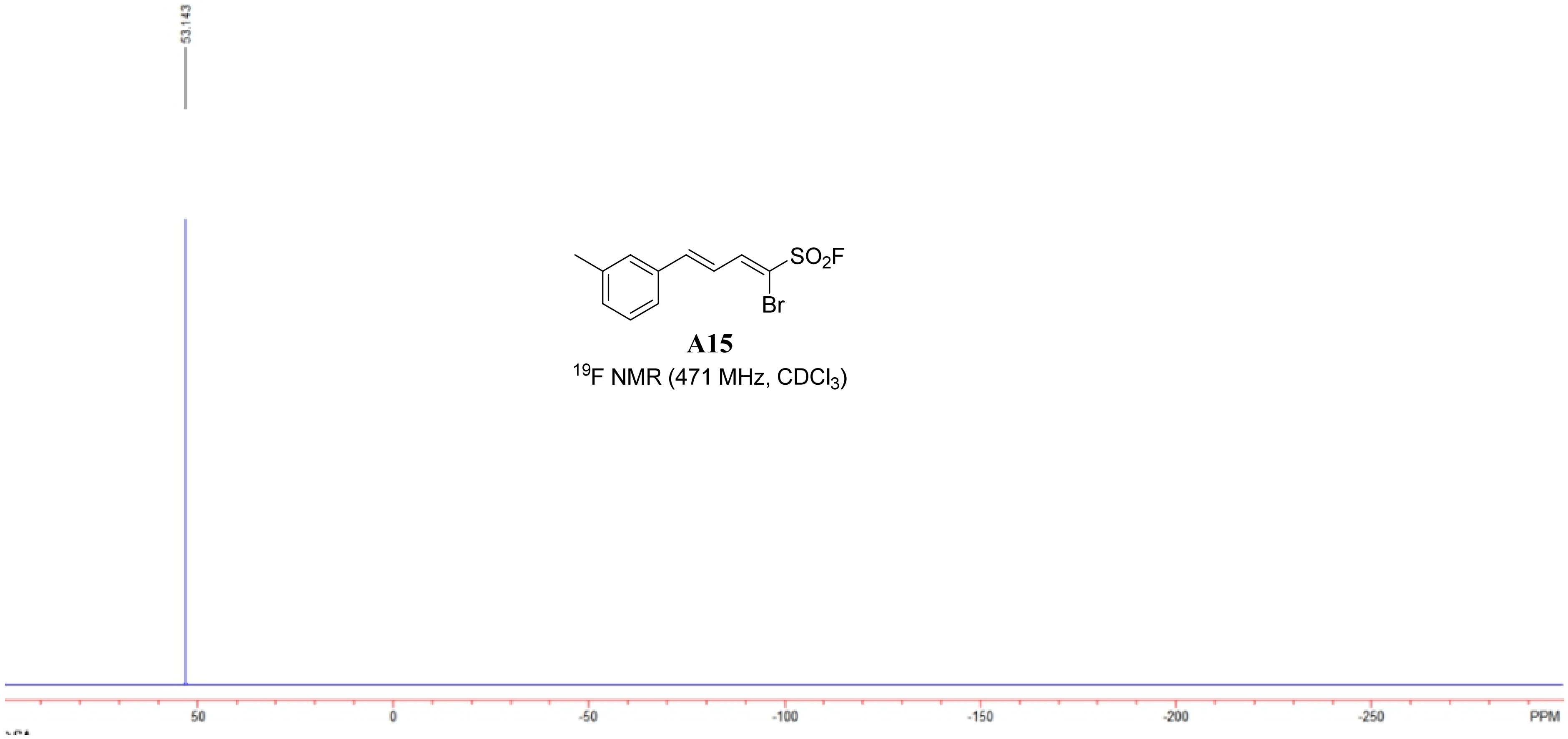

A15

^{19}F NMR (471 MHz, $CDCl_3$)

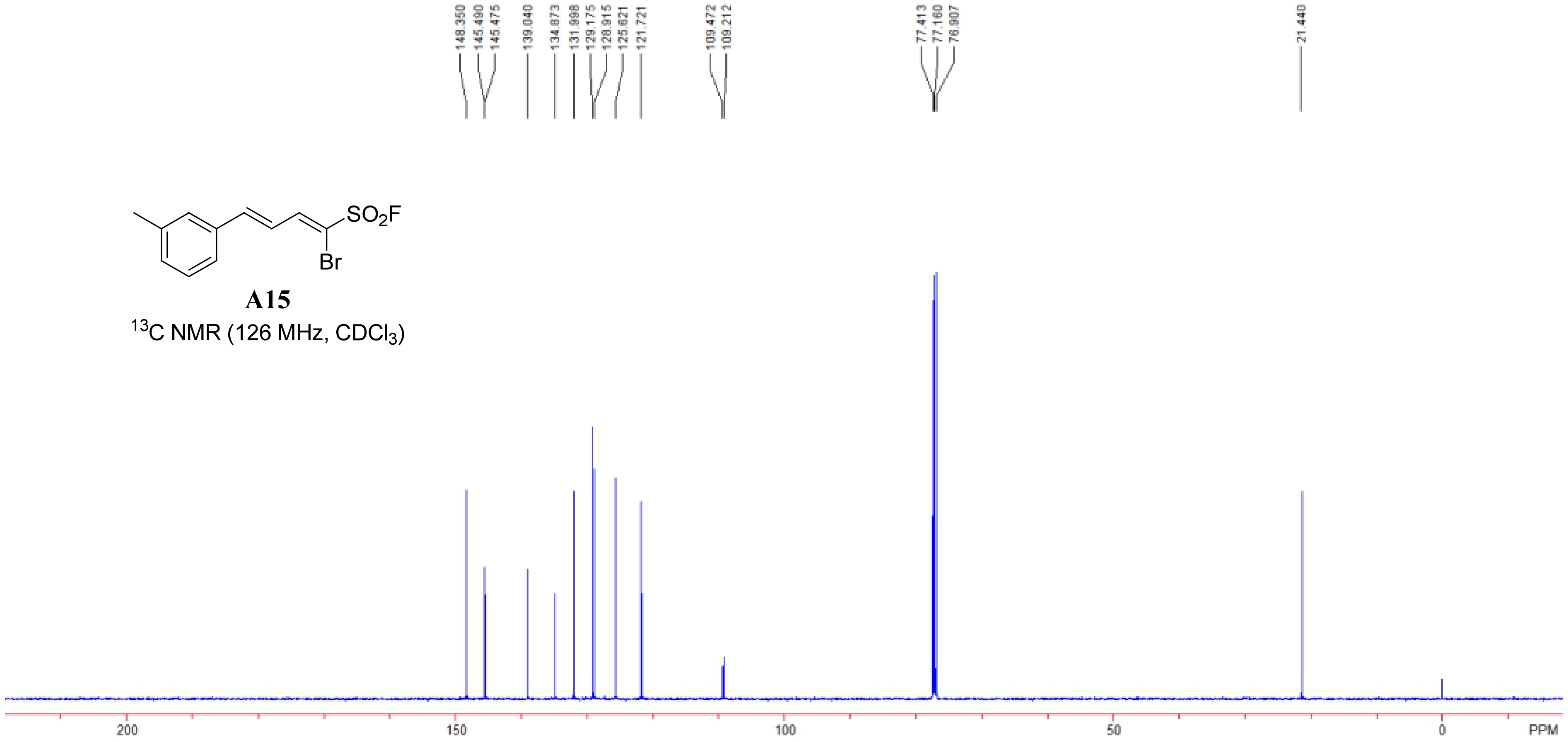

A15

^{13}C NMR (126 MHz, $CDCl_3$)

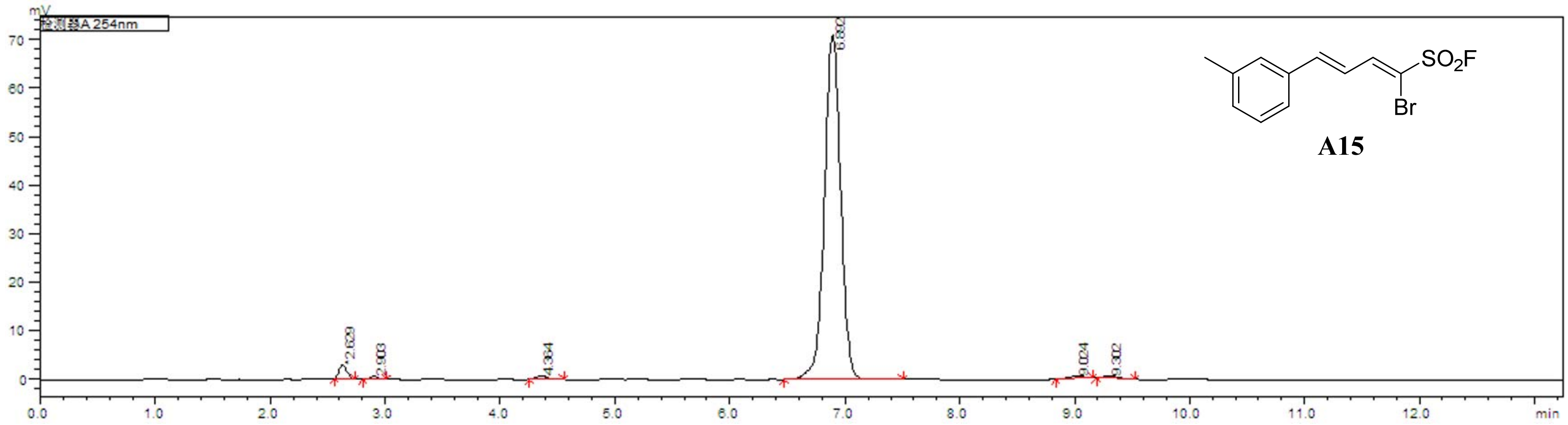

No.	Ret Time (min)	Area (mAU*min)	Rel.Area (%)
1	2.629	13372	1.84%
2	2.903	3189	0.44%
3	4.364	5148	0.71%
4	6.892	698070	95.81%
5	9.024	4588	0.63%
6	9.302	4246	0.58%
Total		728612	

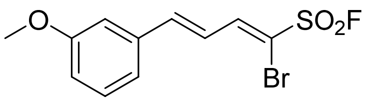

A16

^{1}H NMR (500 MHz, $CDCl_3$)

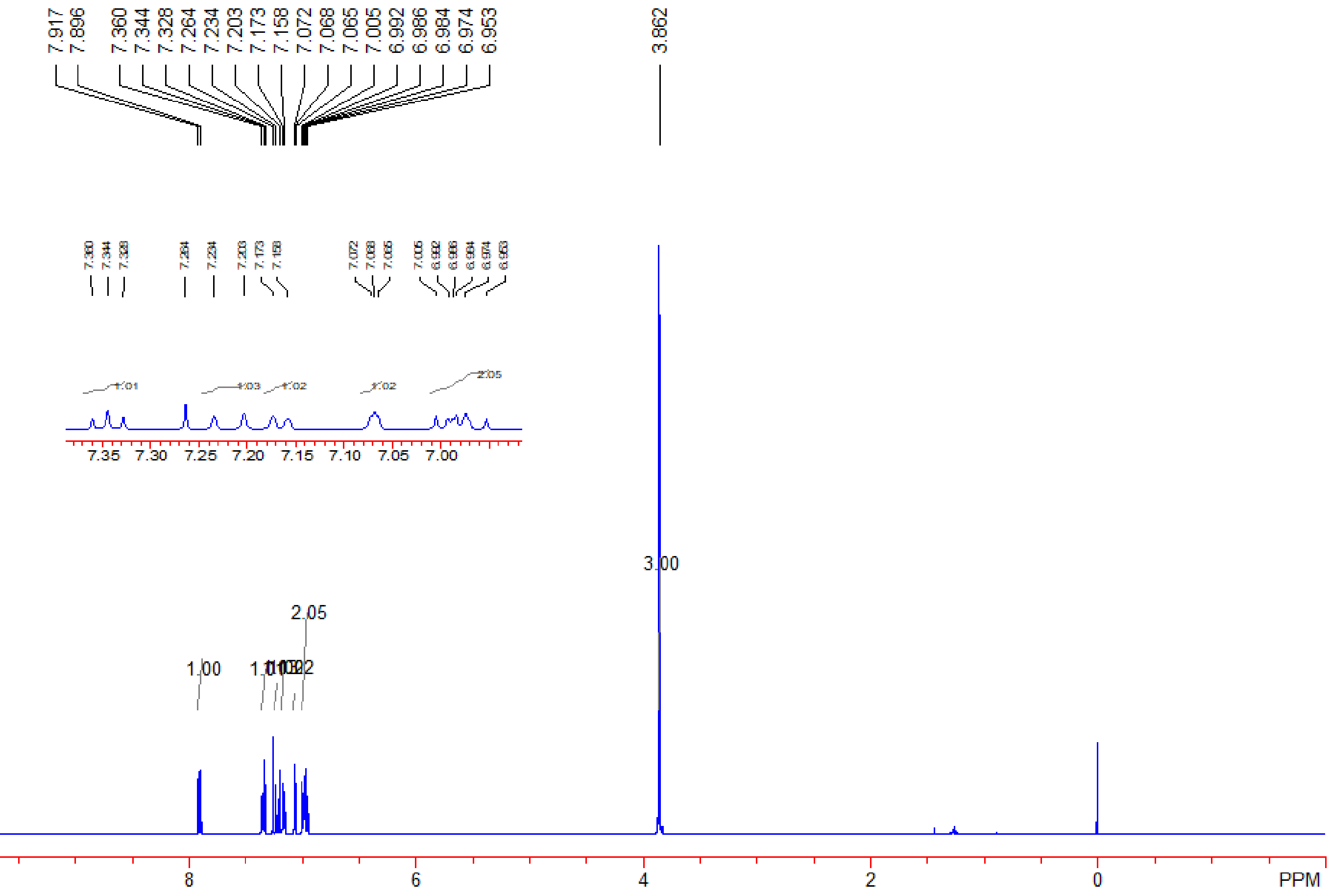

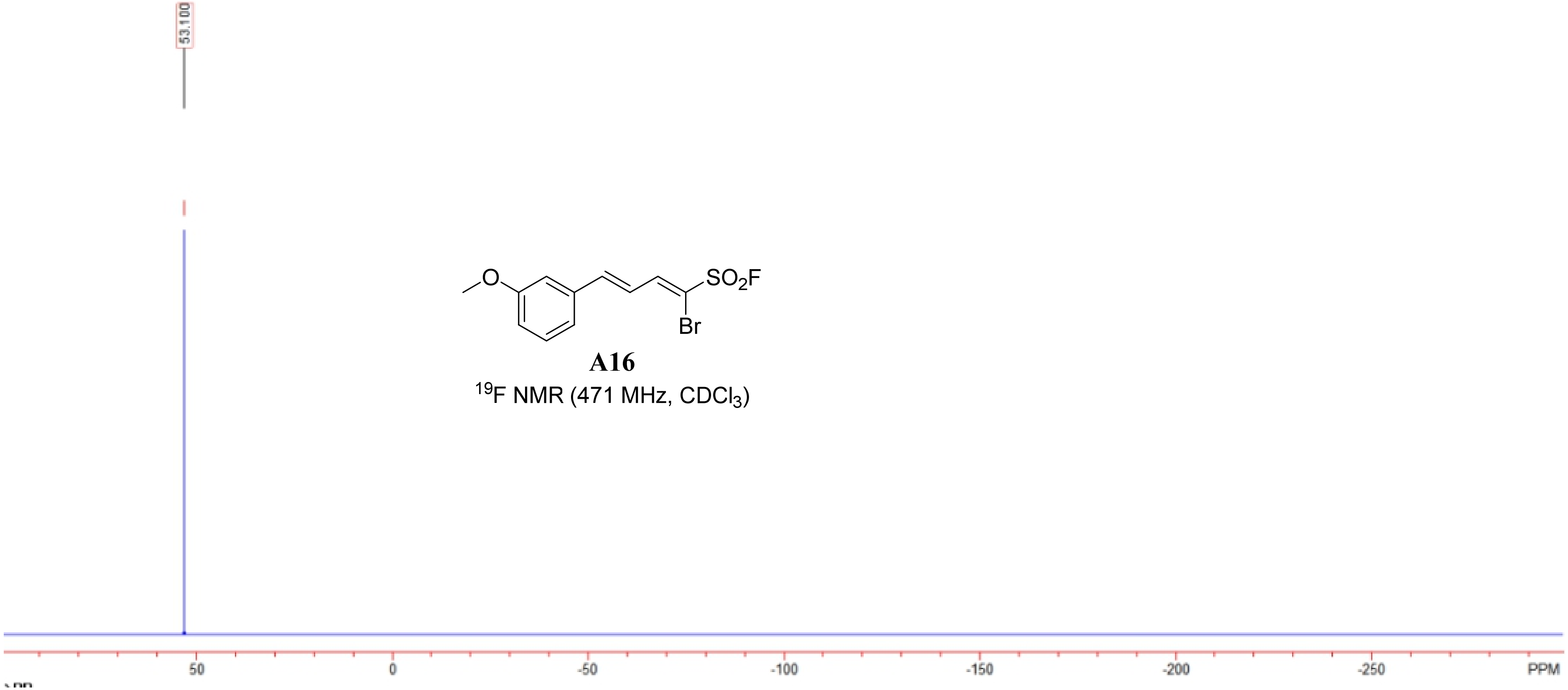

A16

^{19}F NMR (471 MHz, $CDCl_3$)

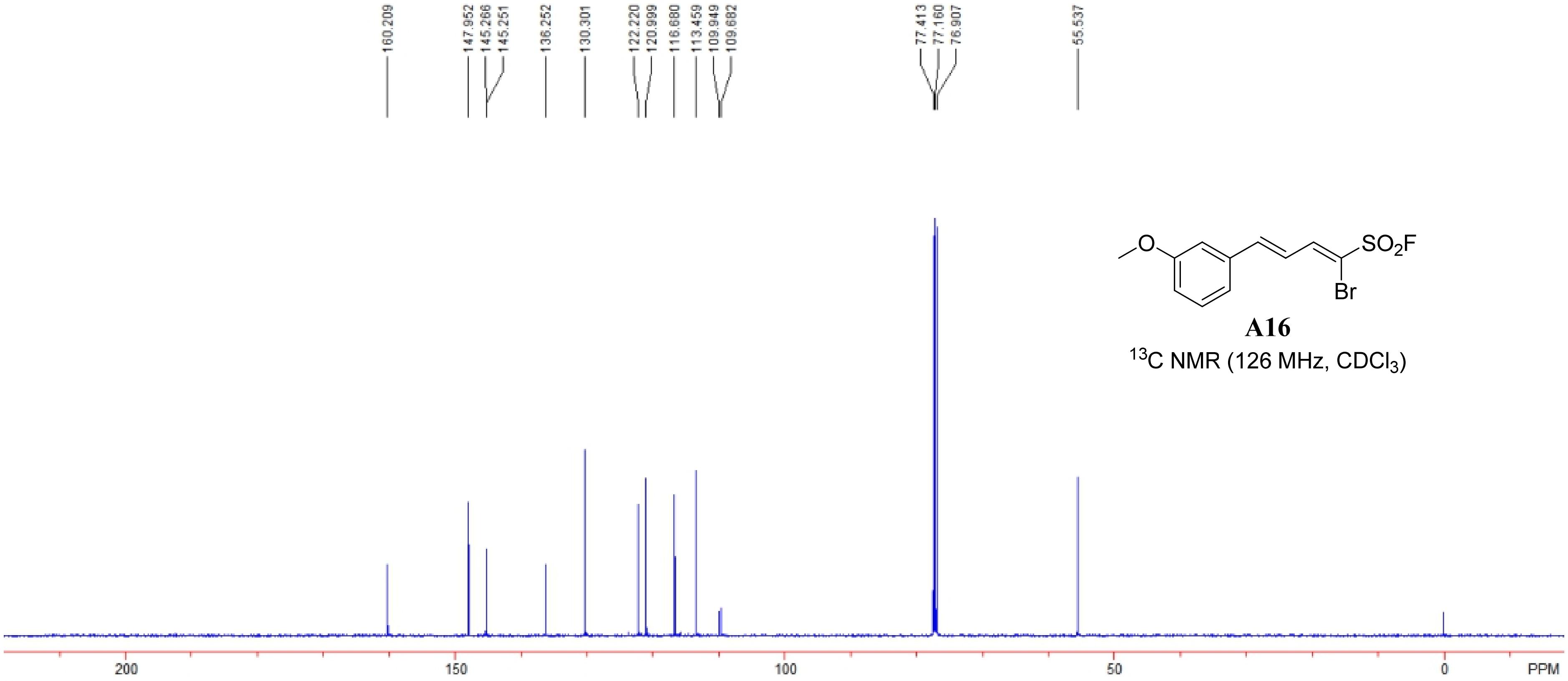

A16

^{13}C NMR (126 MHz, $CDCl_3$)

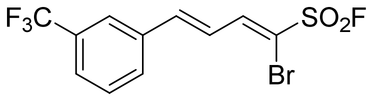

A17

^{1}H NMR (500 MHz, $CDCl_3$)

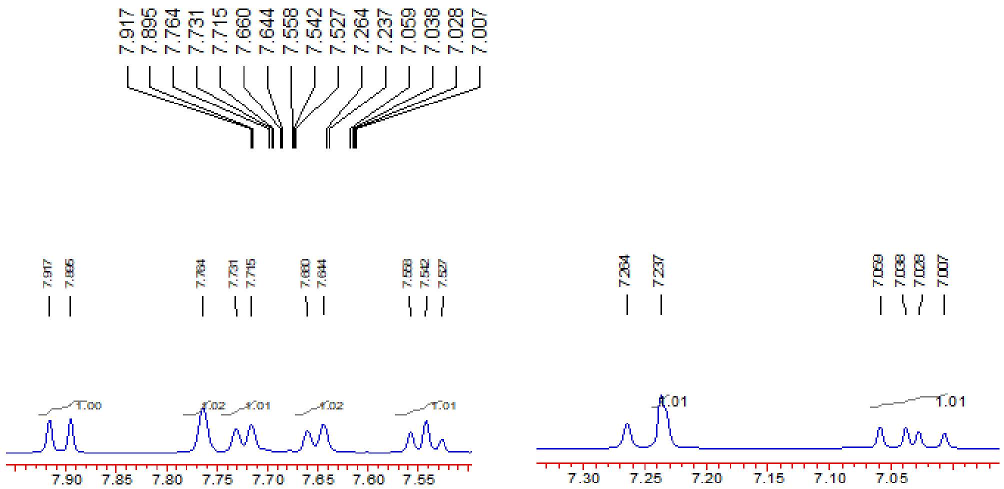

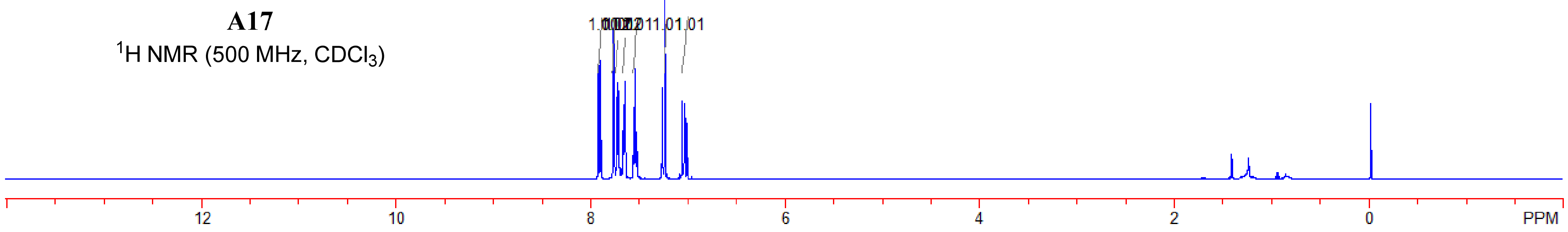

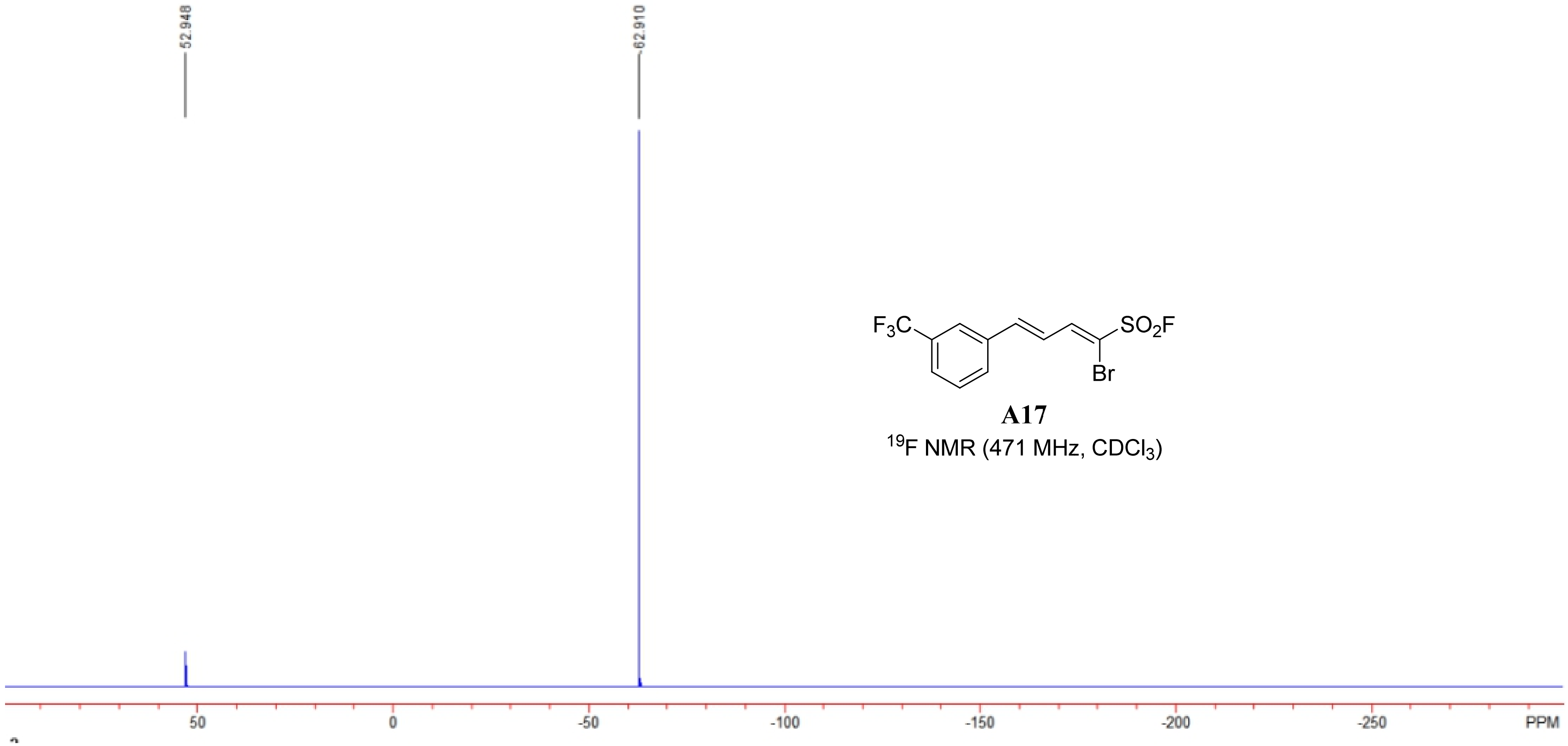

A17

^{19}F NMR (471 MHz, $CDCl_3$)

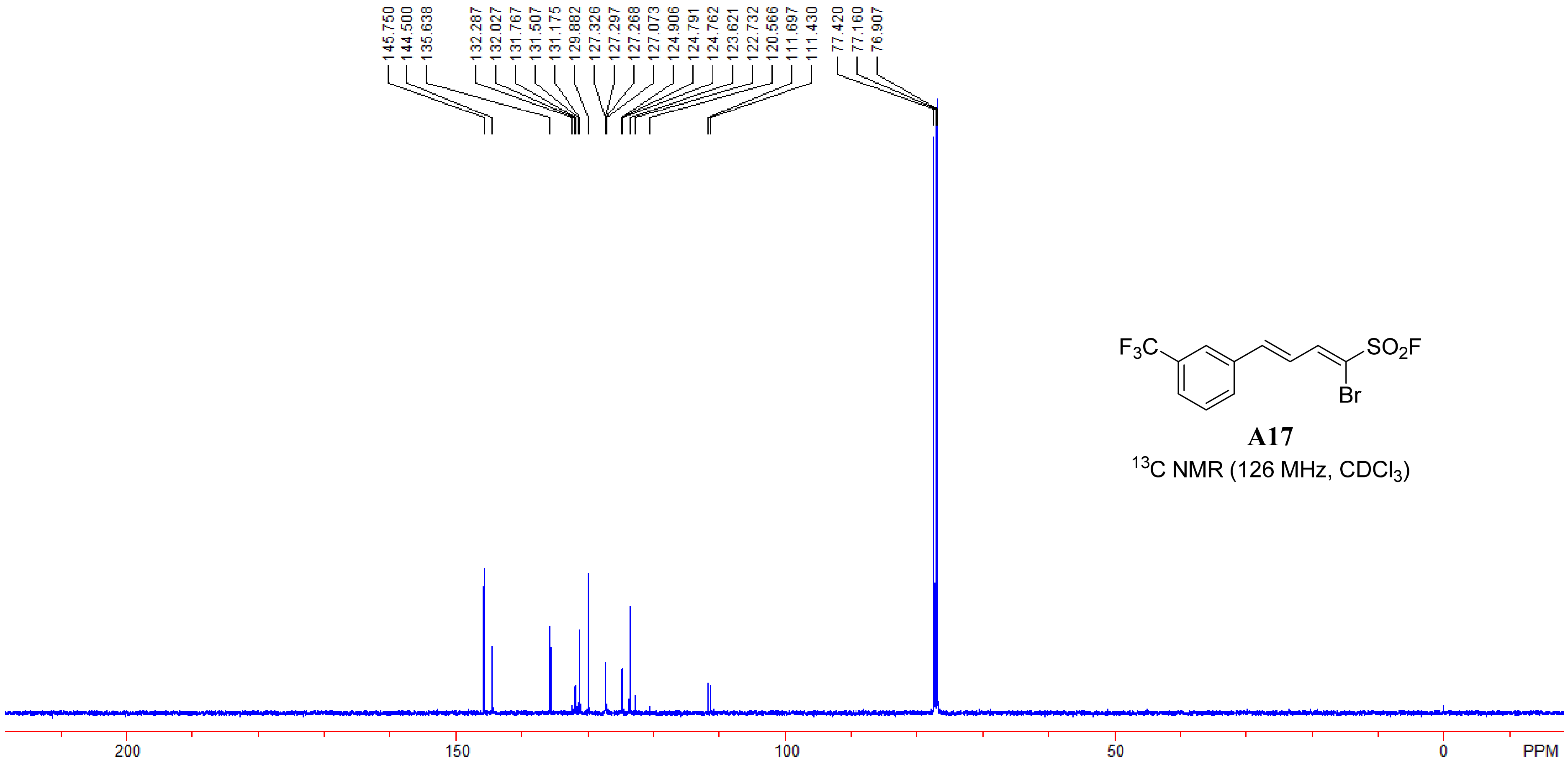

A17

^{13}C NMR (126 MHz, $CDCl_3$)

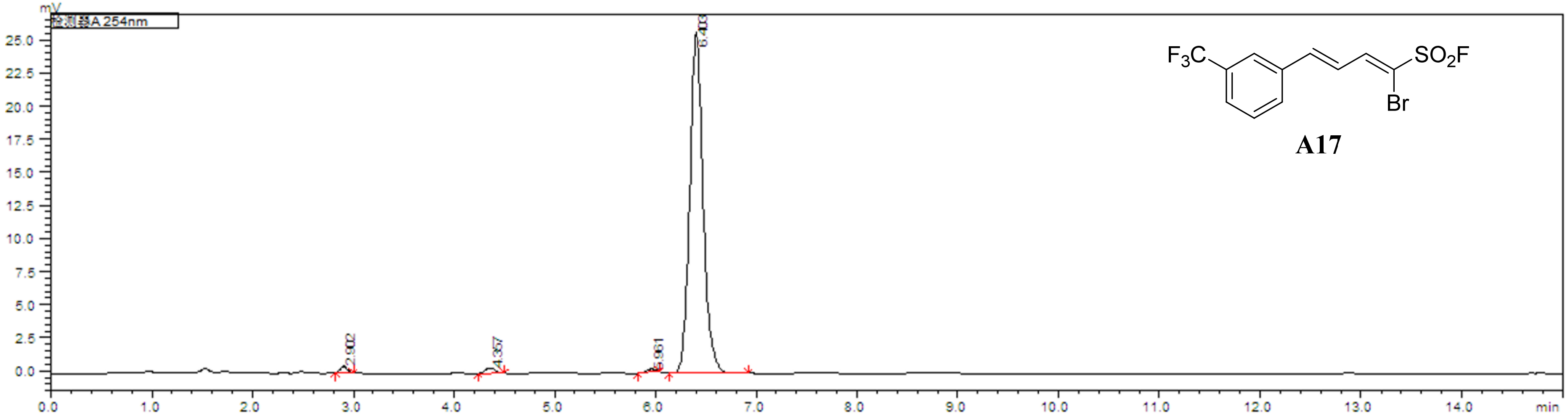

A17

No.	Ret Time (min)	Area (mAU*min)	Rel.Area (%)
1	2.902	2396	0.94%
2	4.357	2805	1.10%
3	5.961	1633	0.64%
4	6.403	247123	97.31%
Total		253957	

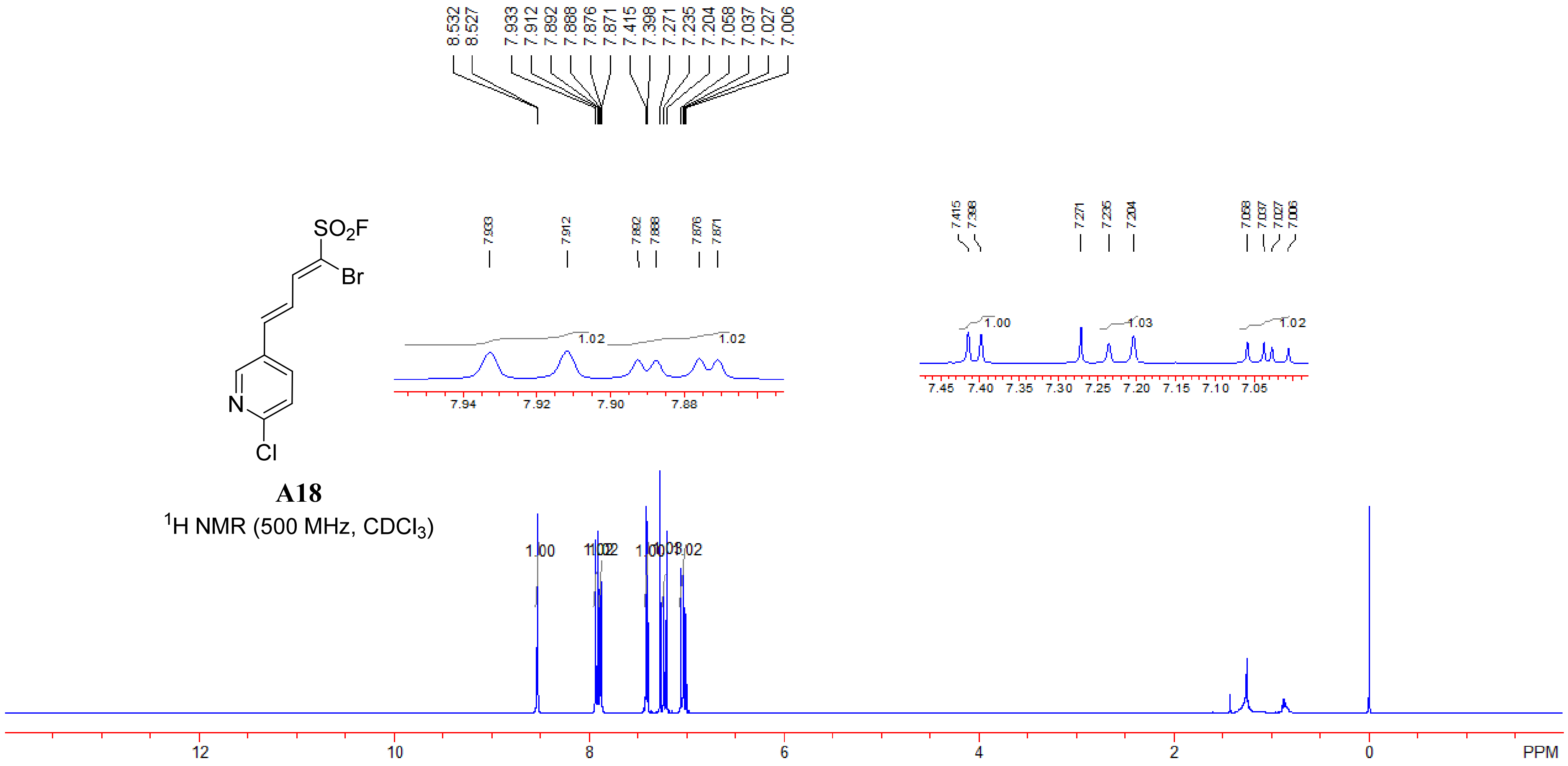

A18

^{1}H NMR (500 MHz, $CDCl_3$)

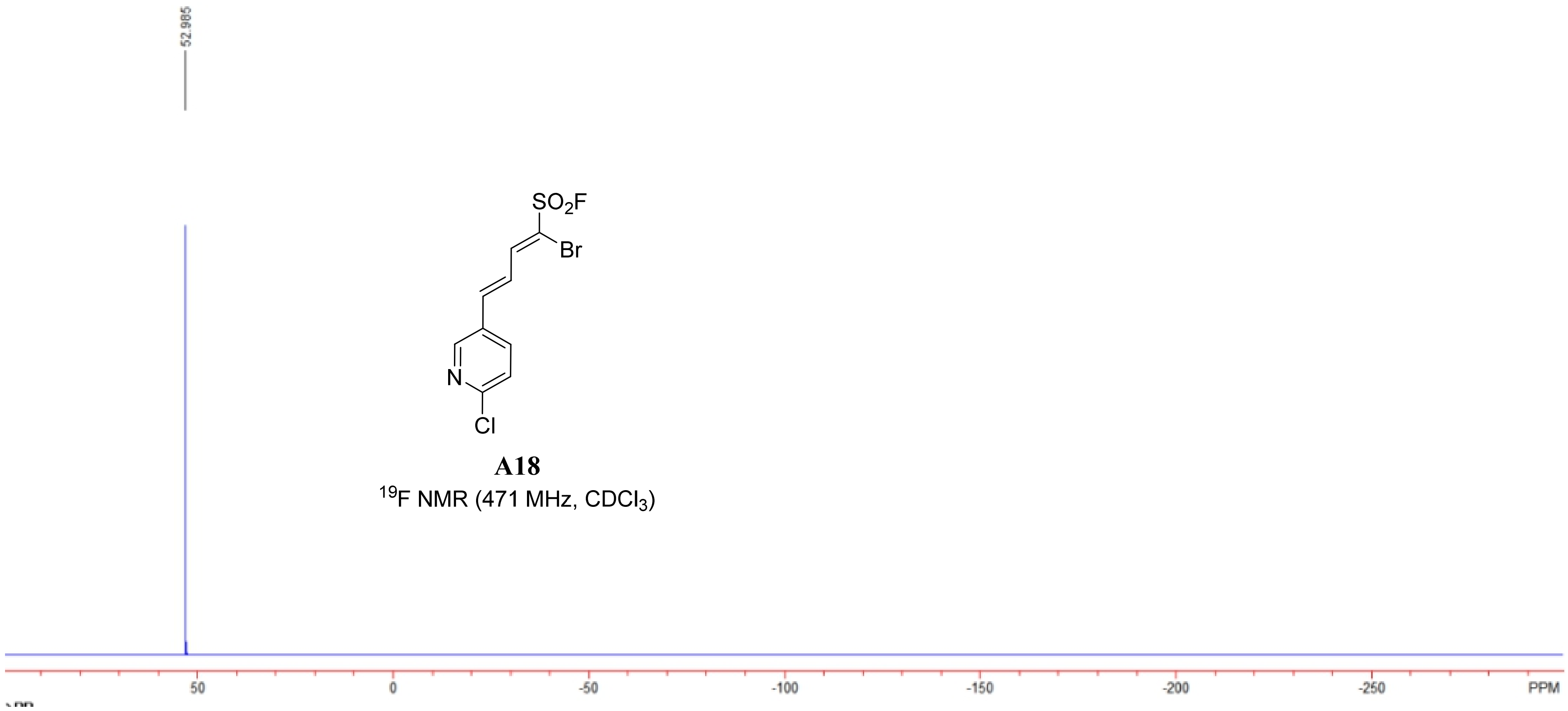

A18

^{19}F NMR (471 MHz, $CDCl_3$)

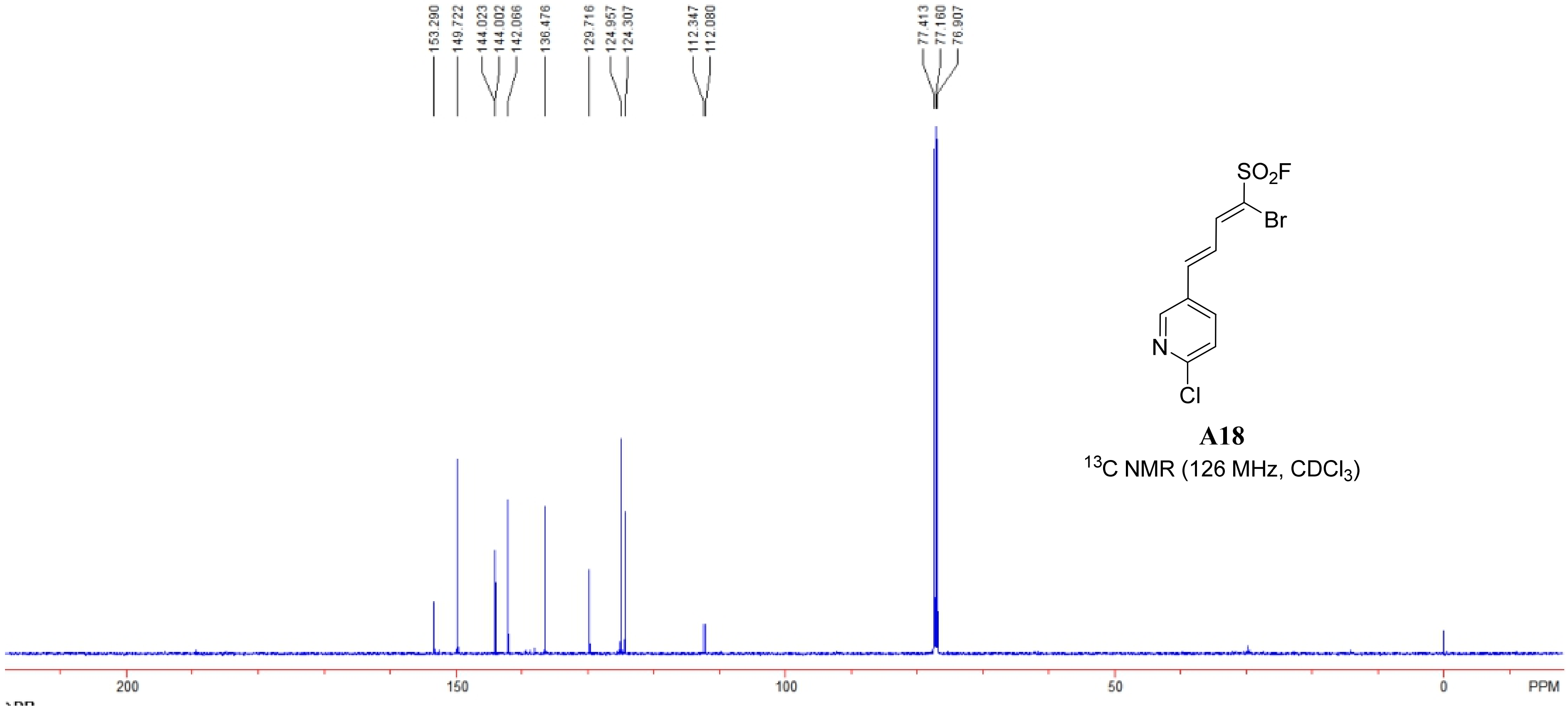

A18

^{13}C NMR (126 MHz, $CDCl_3$)

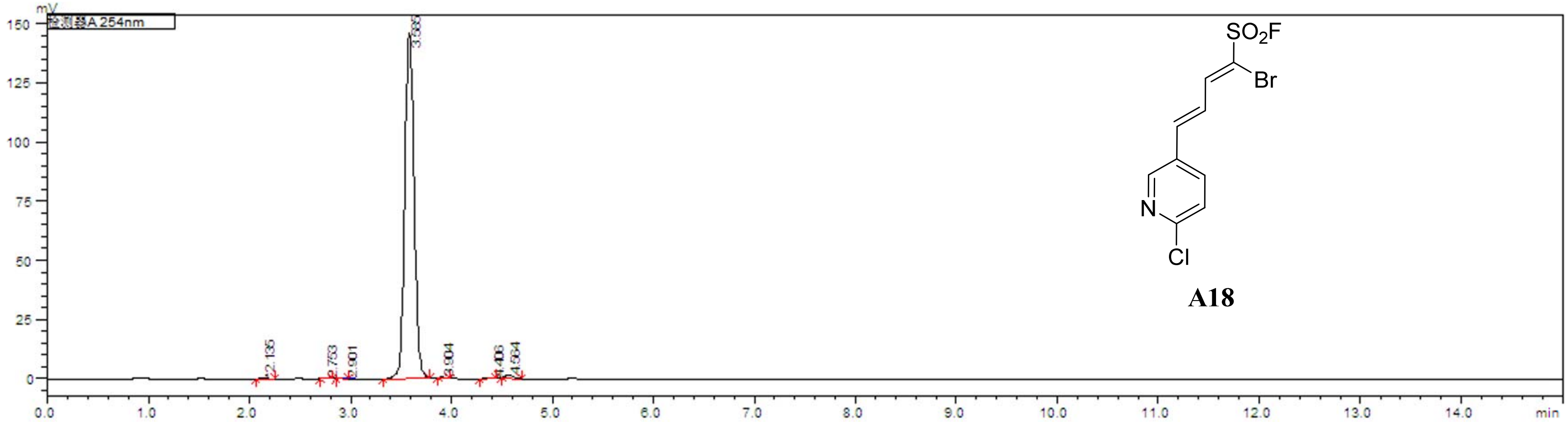

A18

No.	Ret Time (min)	Area (mAU*min)	Rel.Area (%)
1	2.135	6546	0.70%
2	2.753	1235	0.13%
3	2.901	1218	0.13%
4	3.585	918355	98.71%
5	3.904	1317	0.14%
6	4.406	1063	0.11%
7	4.564	5640	0.61%
Total		930373	

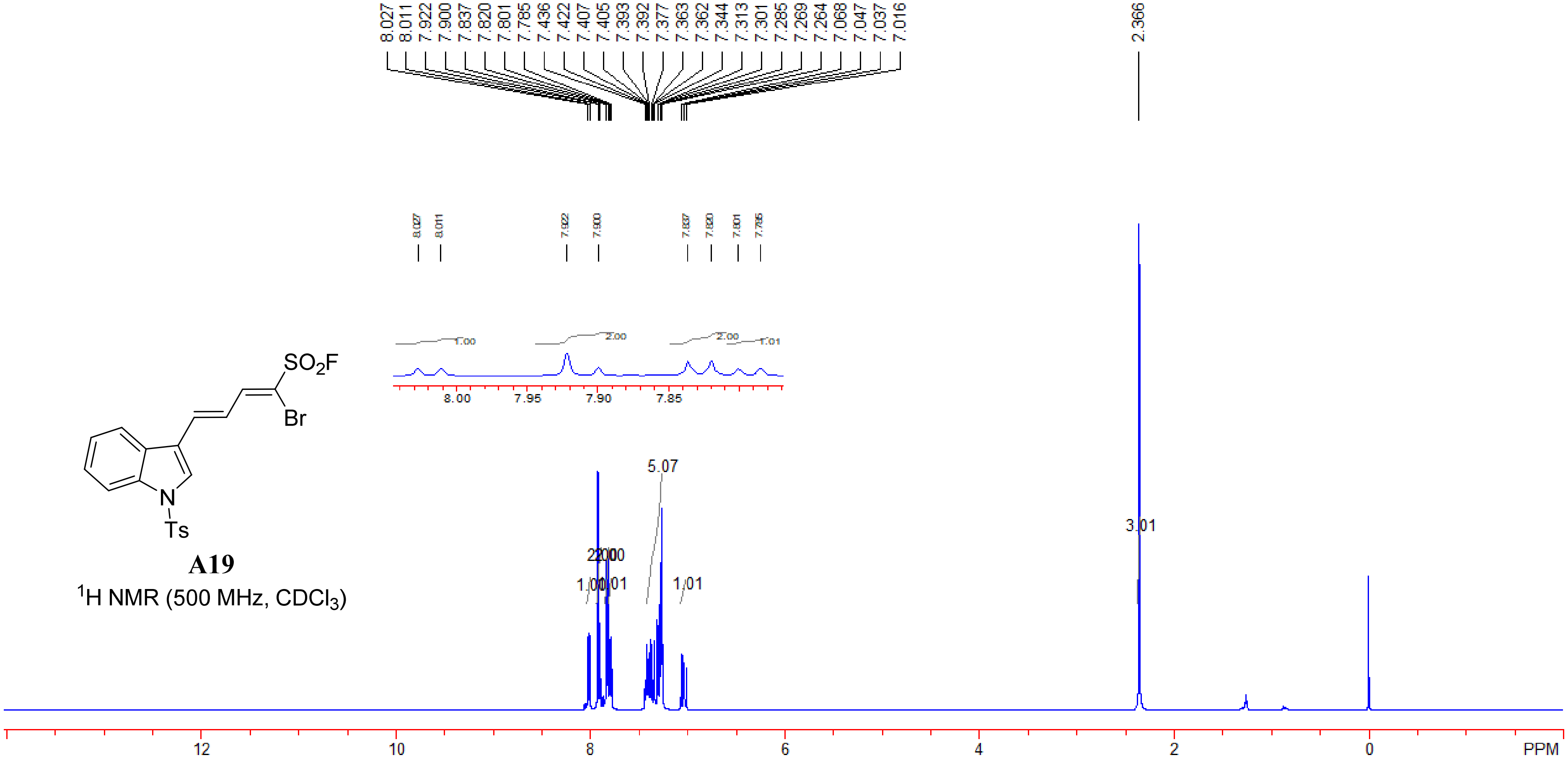

A19

^{1}H NMR (500 MHz, $CDCl_3$)

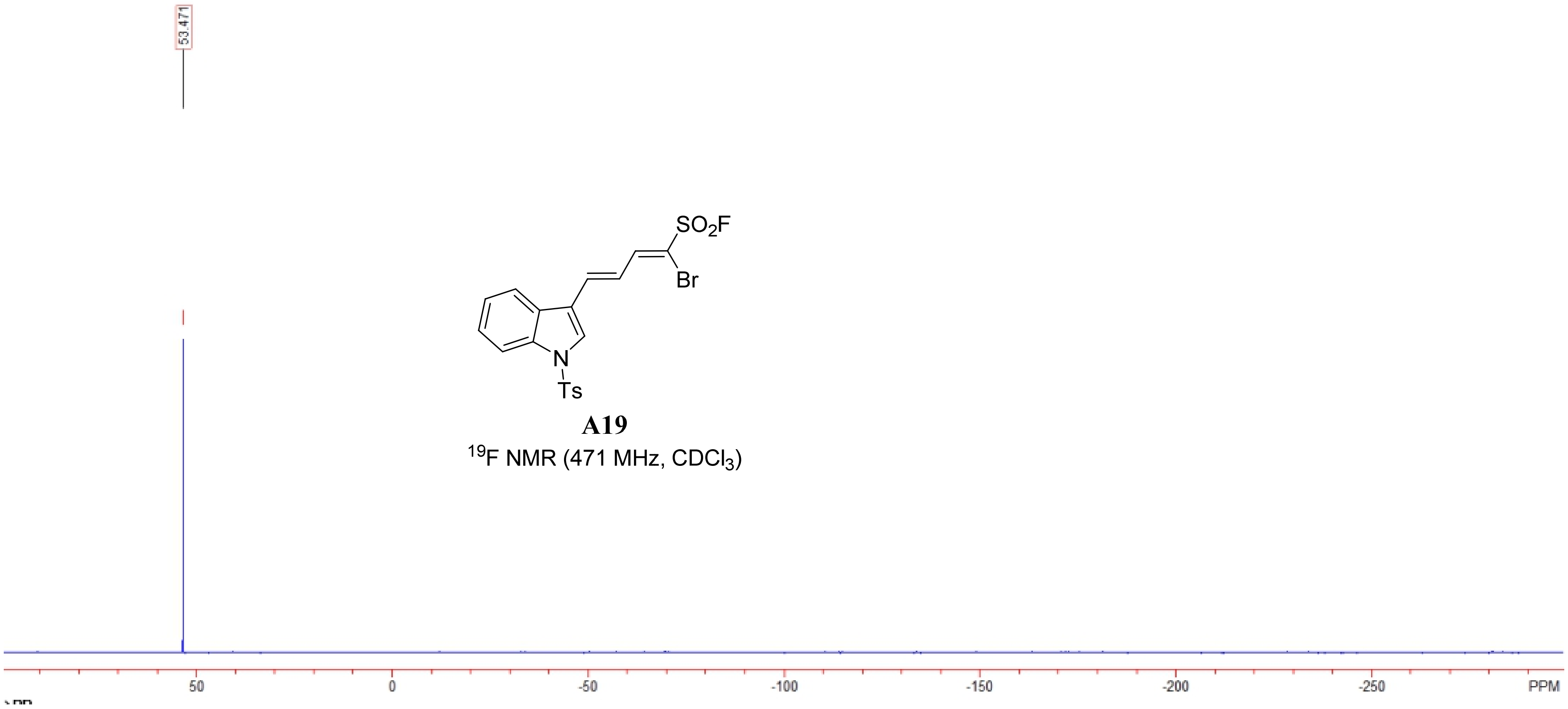

A19

^{19}F NMR (471 MHz, $CDCl_3$)

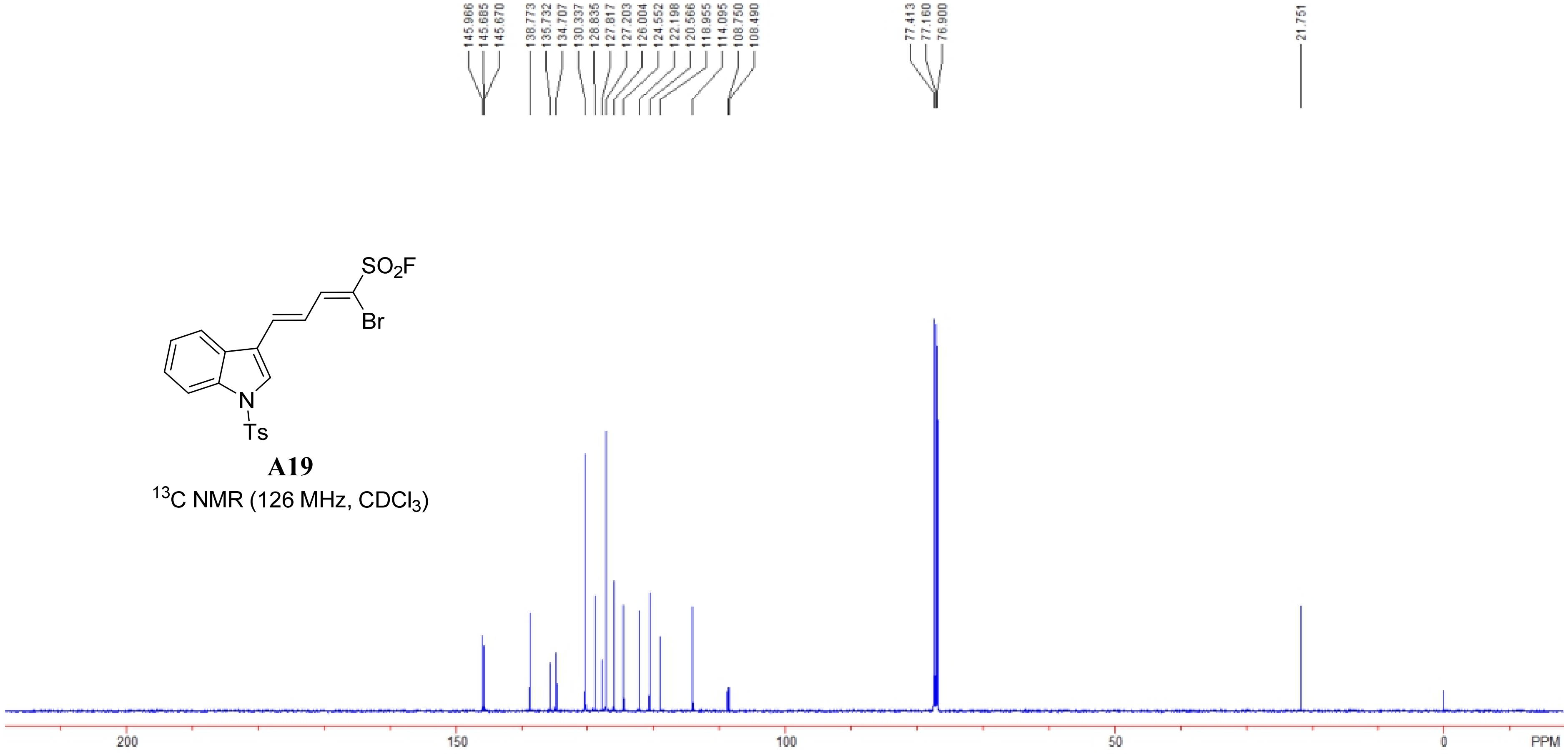

A19

^{13}C NMR (126 MHz, $CDCl_3$)

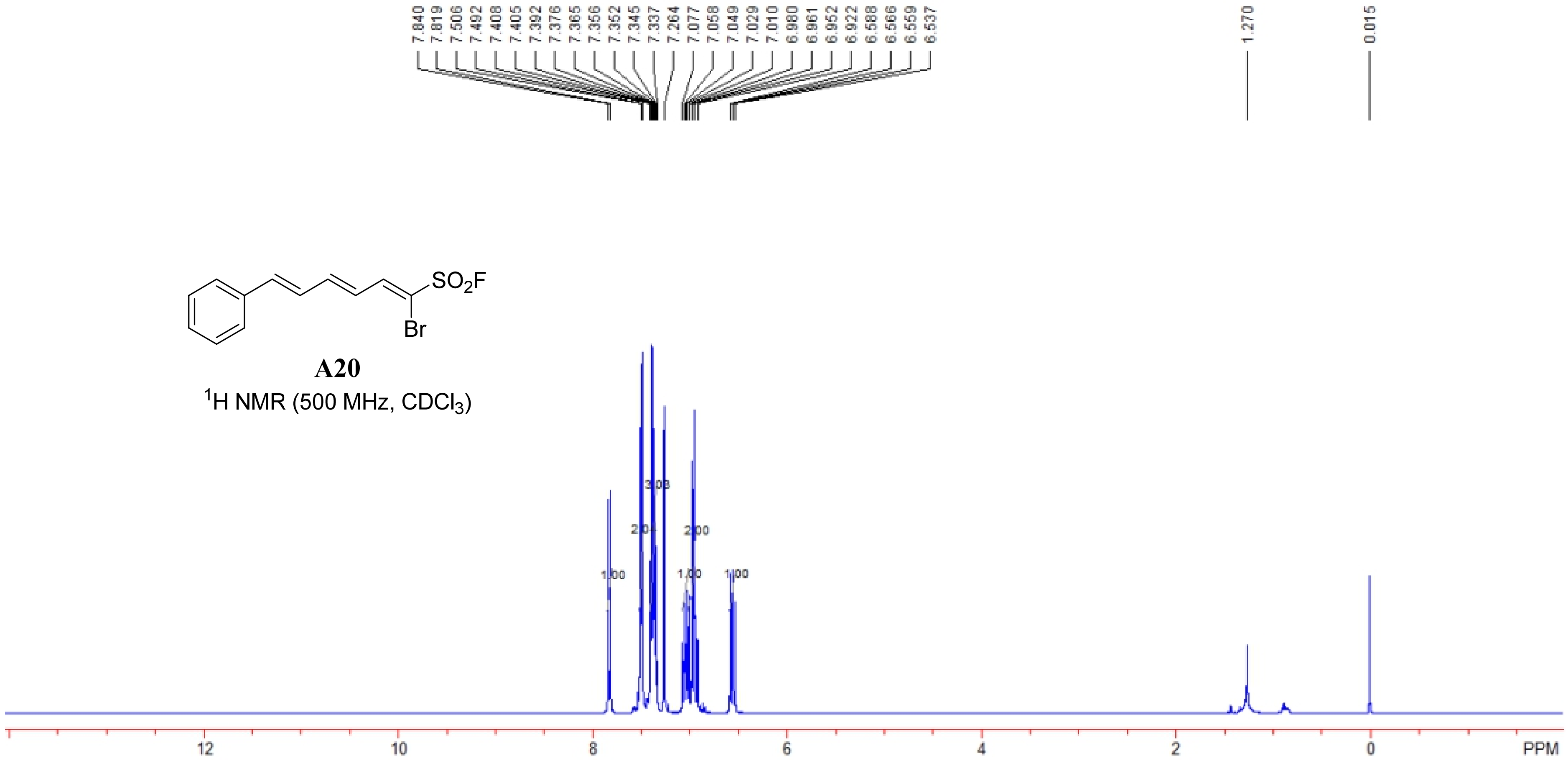

A20

^{1}H NMR (500 MHz, $CDCl_3$)

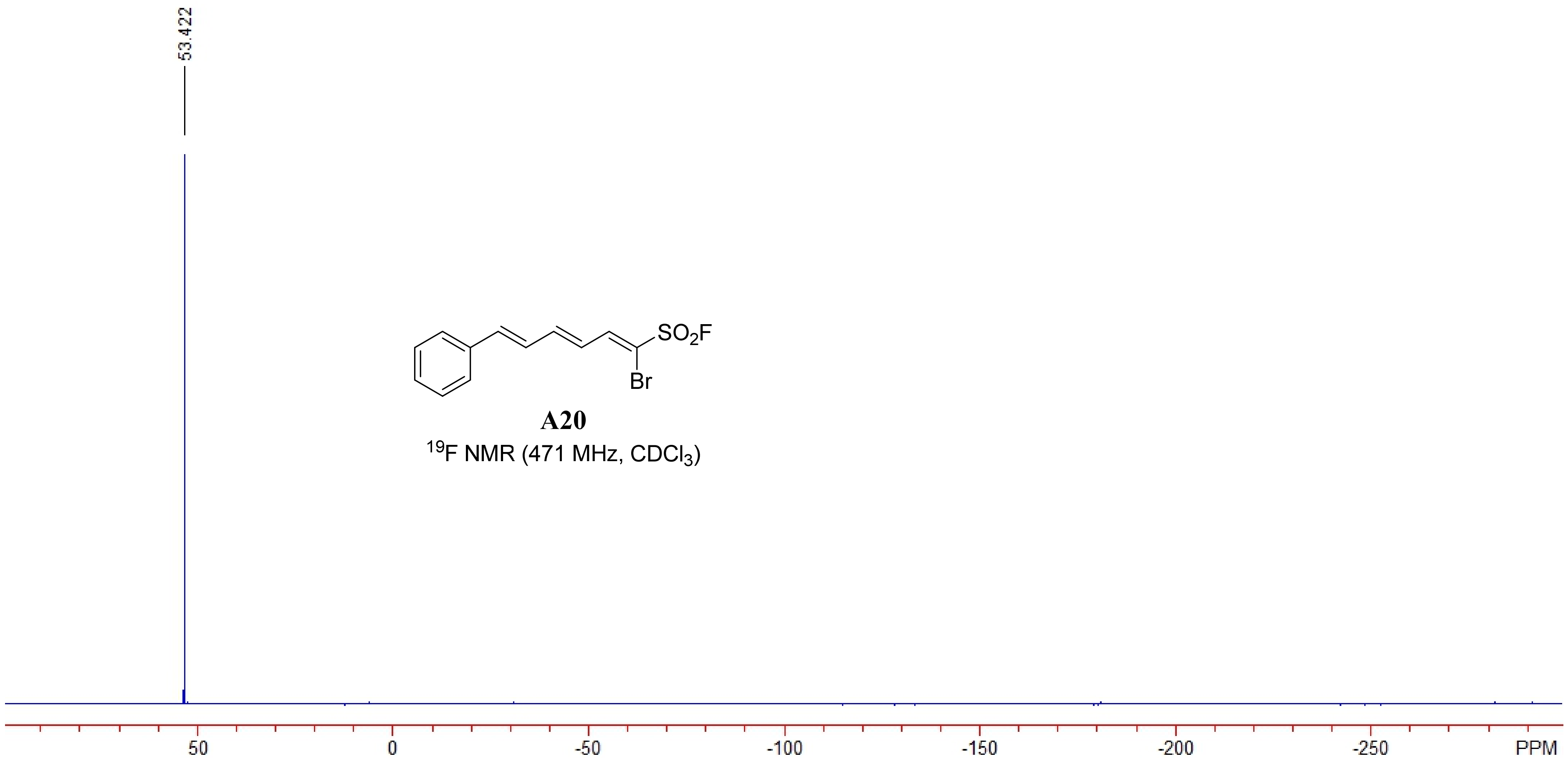
53.422
SO2F
Br
A20
19F NMR (471 MHz, CDCl3)
50
0
-50
-100
-150
-200
-250
PPM

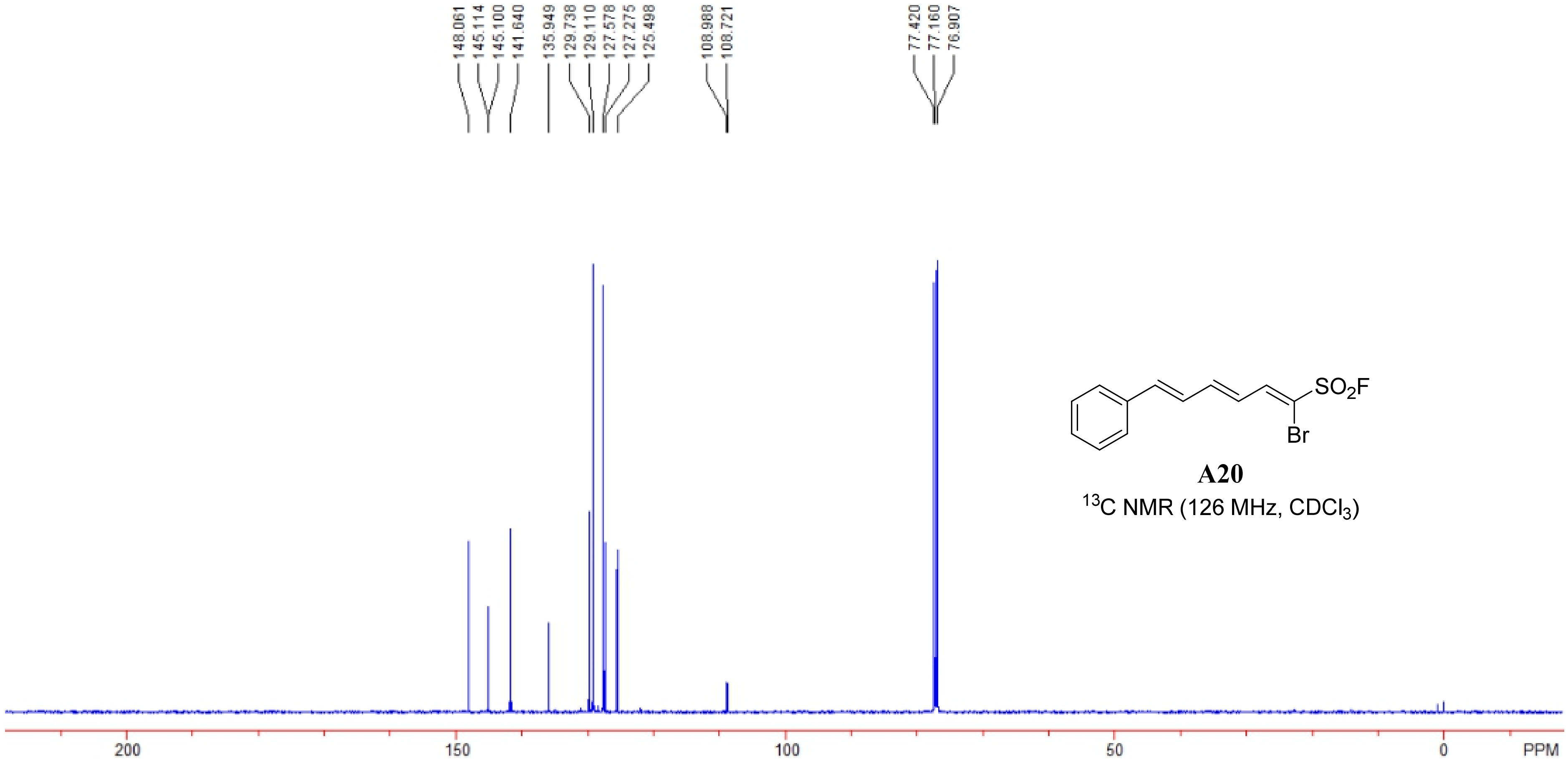

A20

^{13}C NMR (126 MHz, $CDCl_3$)

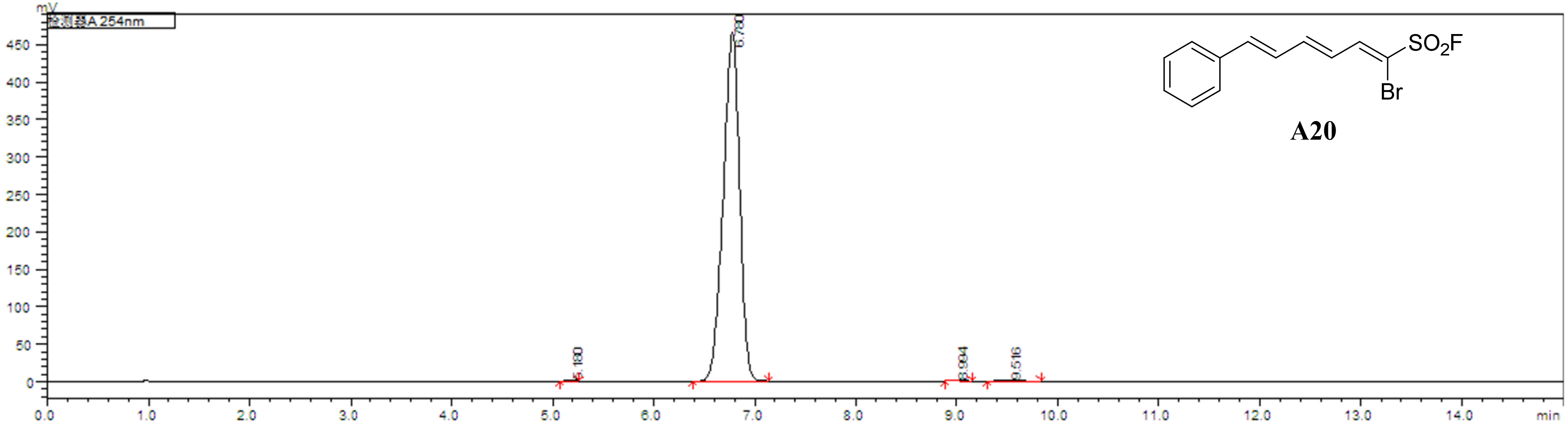

No.	Ret Time (min)	Area (mAU*min)	Rel.Area (%)
1	5.180	6074	0.11%
2	6.780	5279609	99.51%
3	8.994	6989	0.13%
4	9.516	12727	0.24%
Total		5305399	

B1

^{1}H NMR (500 MHz, $CDCl_3$)

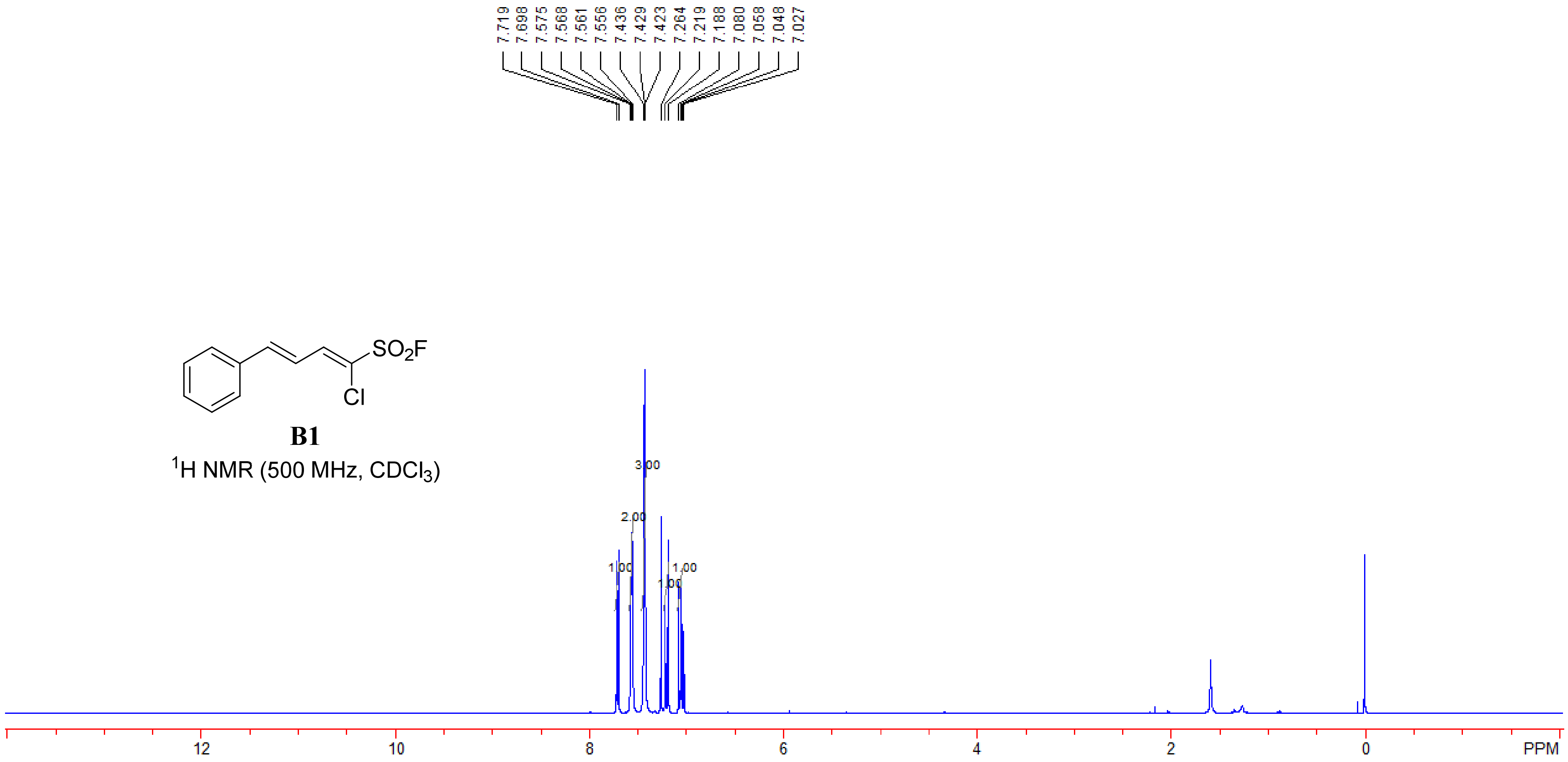

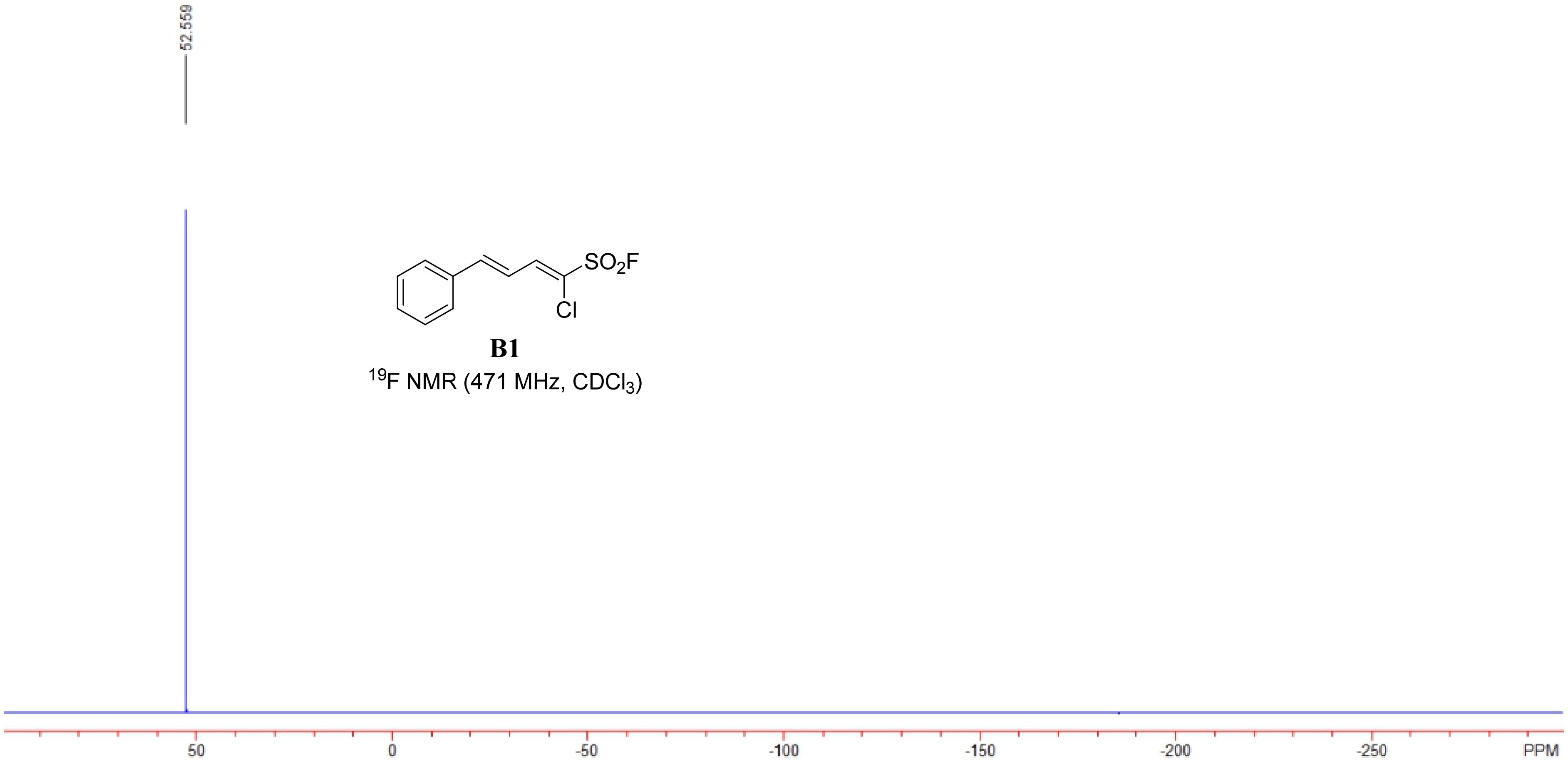

B1

^{19}F NMR (471 MHz, $CDCl_3$)

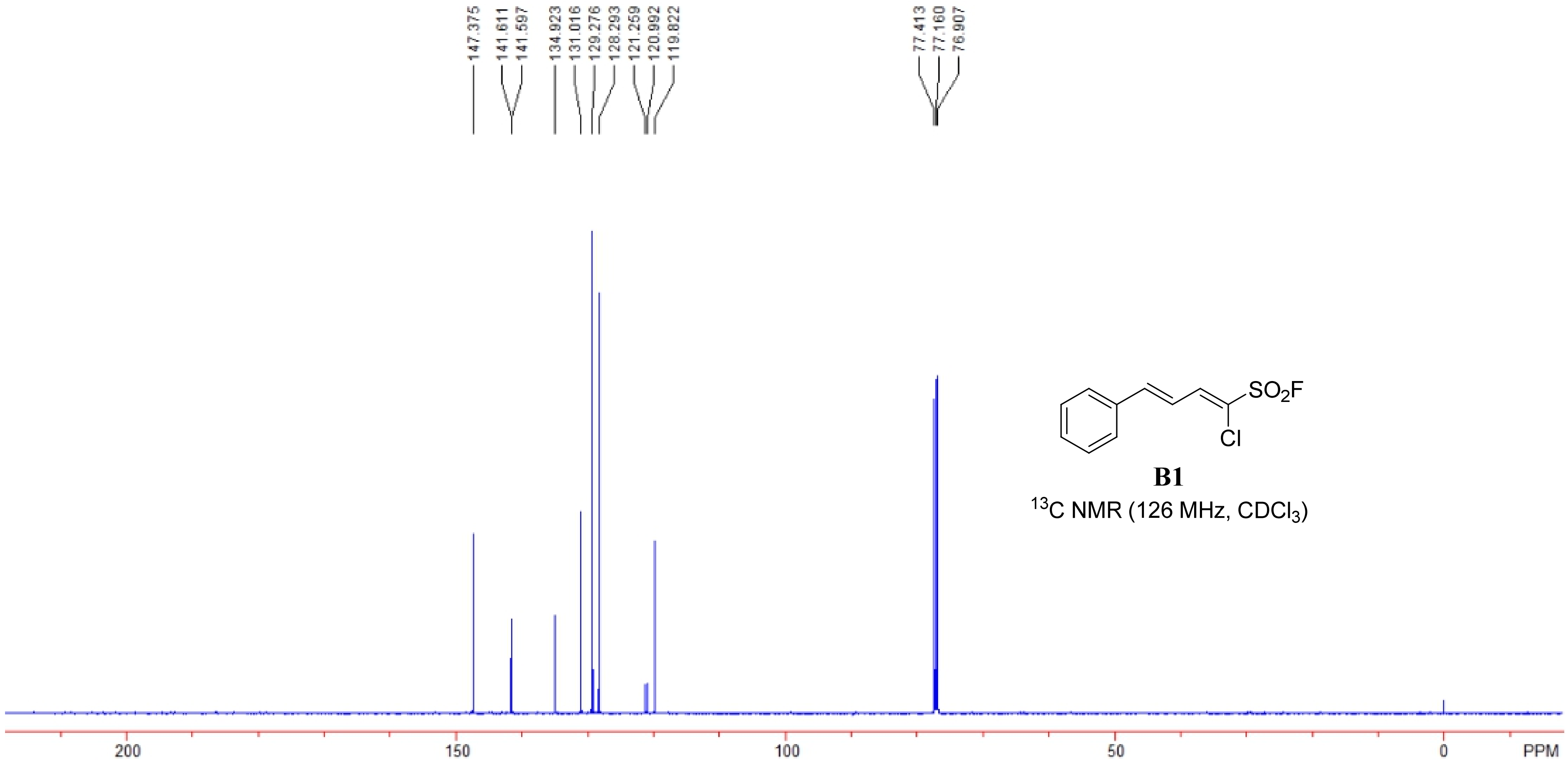

B1

^{13}C NMR (126 MHz, $CDCl_3$)

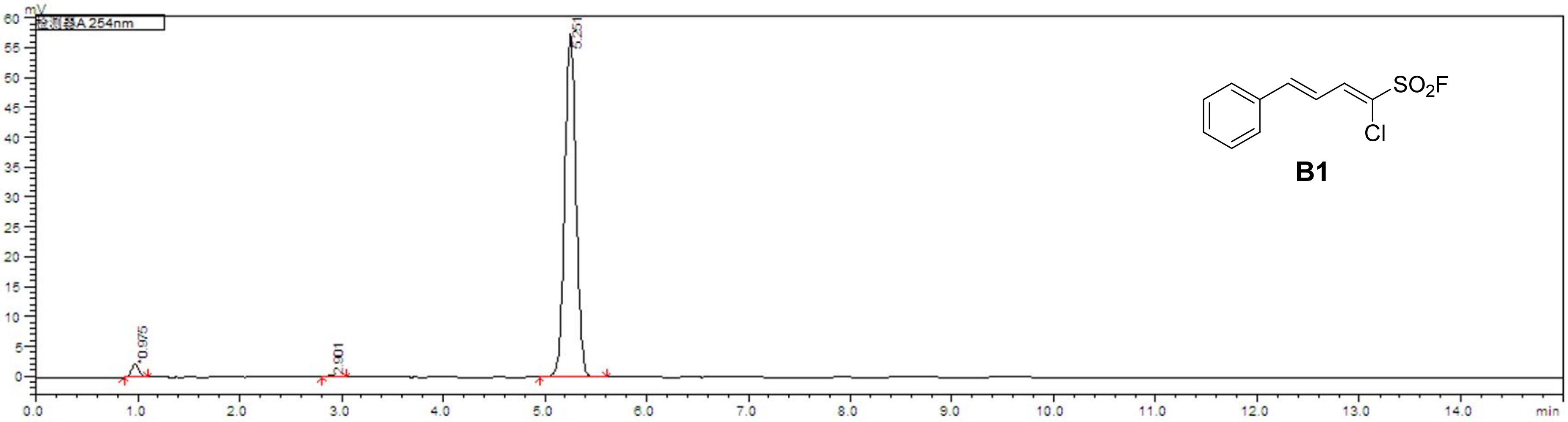

B1

No.	Ret Time (min)	Area (mAU*min)	Rel.Area (%)
1	0.975	10326	2.25%
2	2.901	2298	0.50%
3	5.251	446563	97.25%
Total		459186	

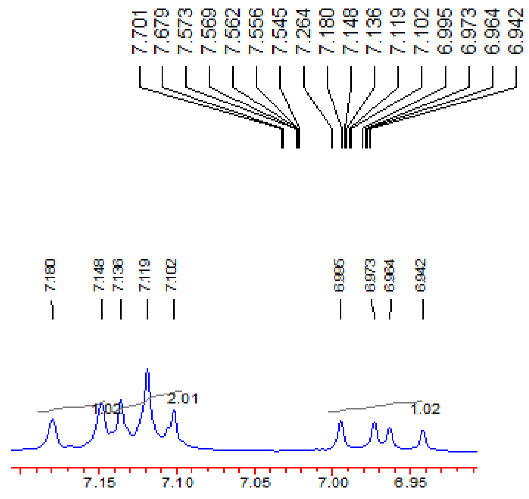

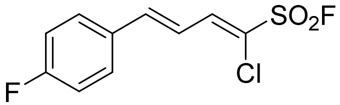

B2

^1H NMR (500 MHz, $CDCl_3$)

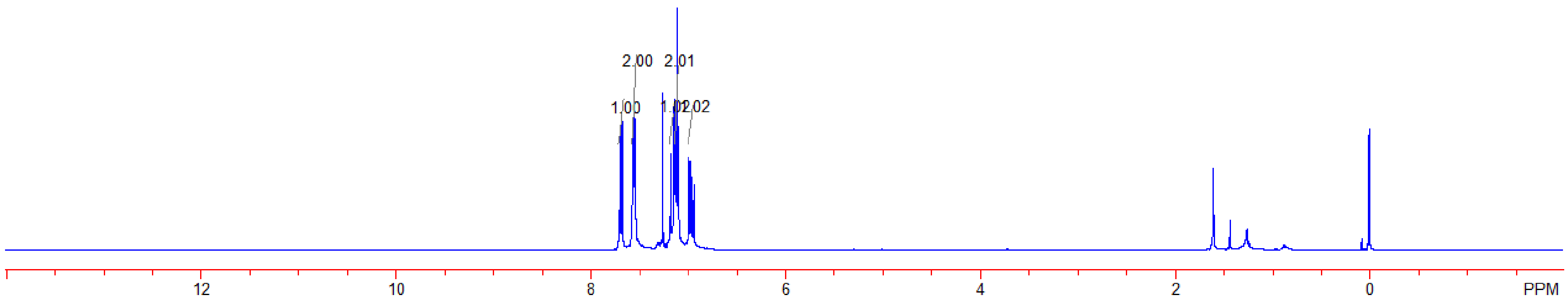

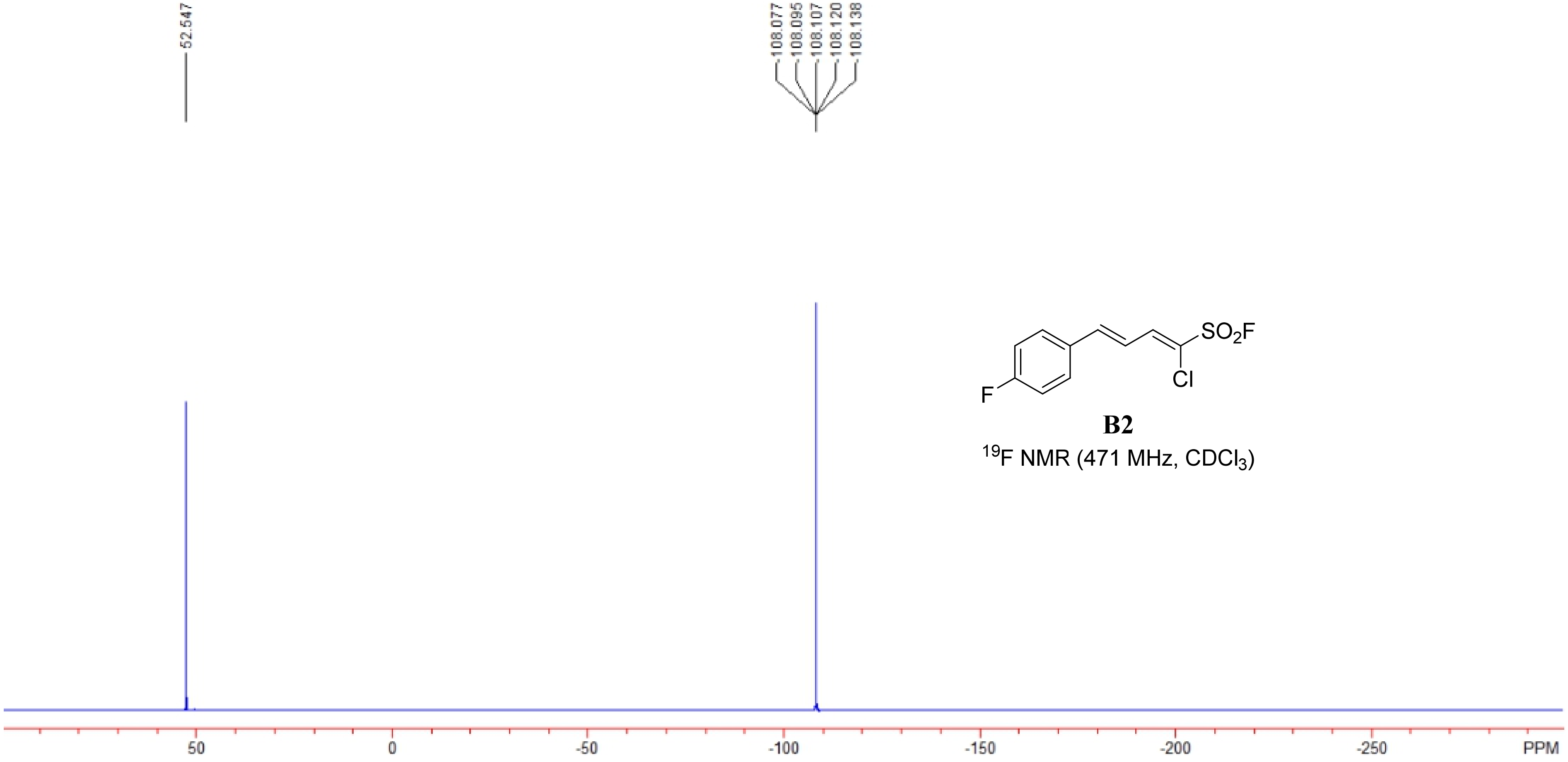

B2

^{19}F NMR (471 MHz, $CDCl_3$)

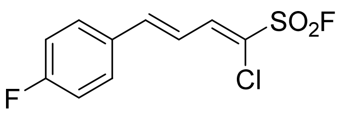

B2

^{13}C NMR (126 MHz, $CDCl_3$)

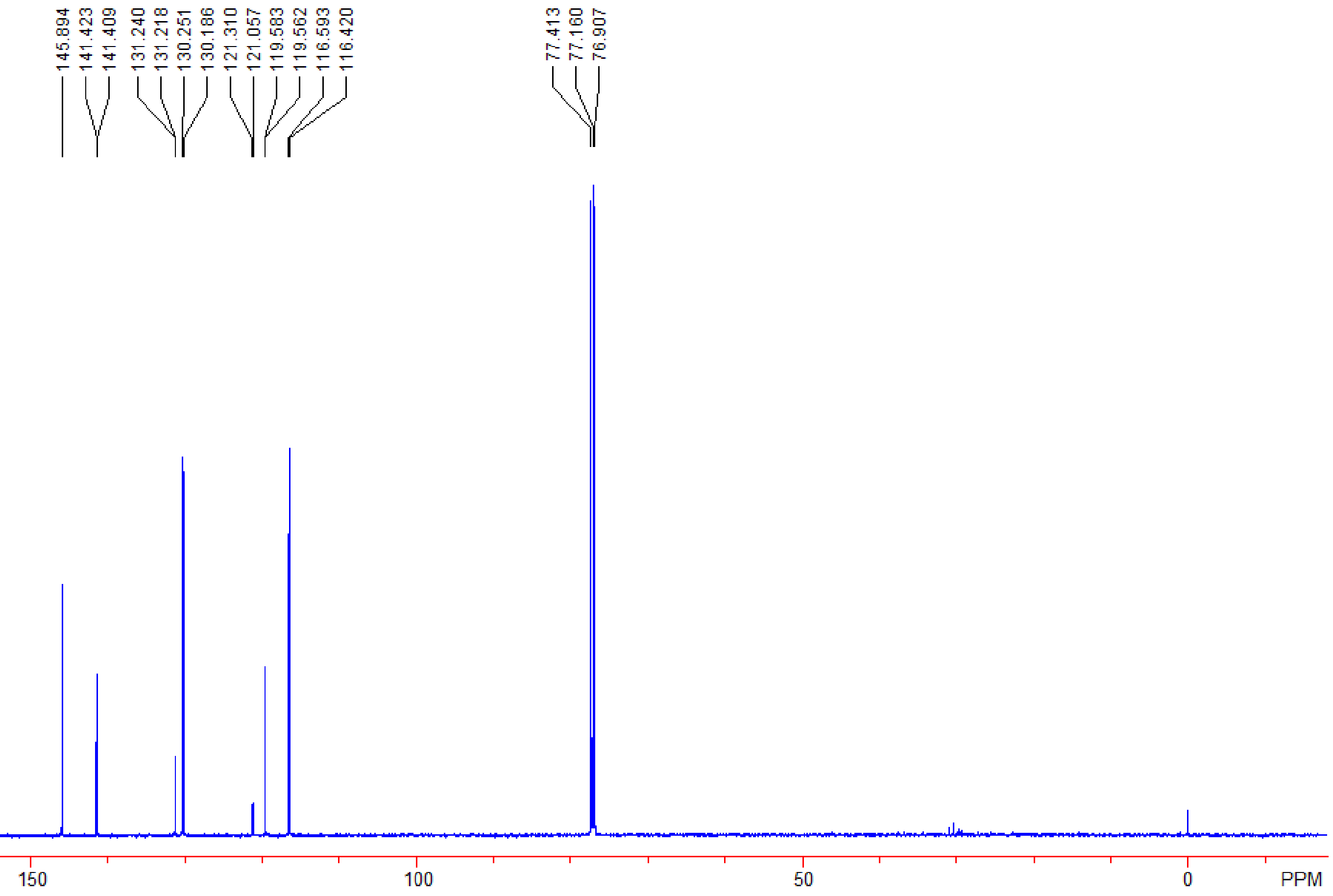

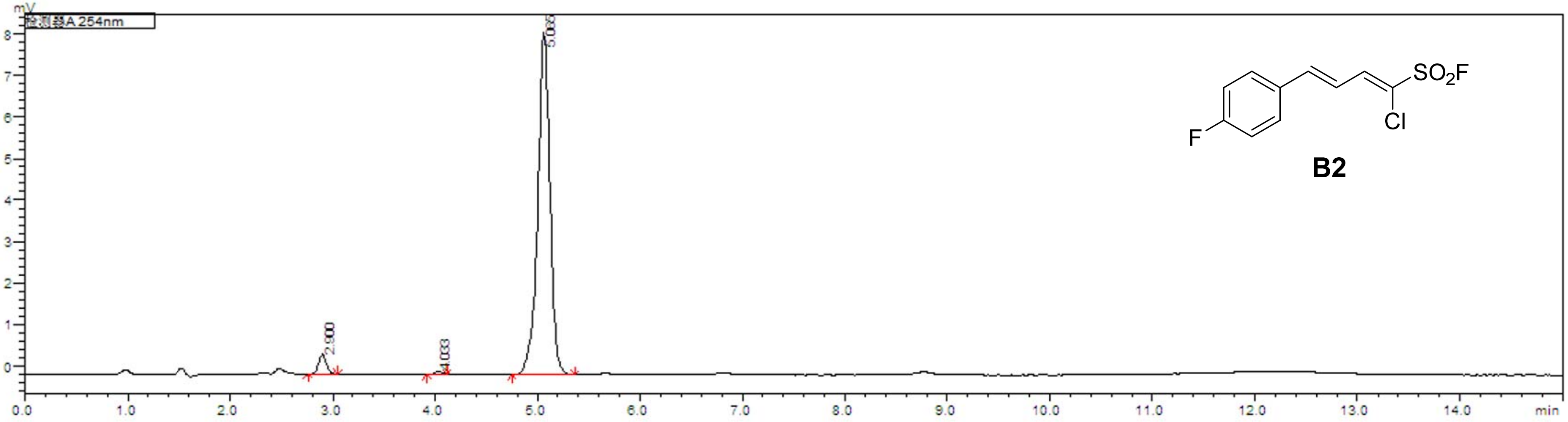

B2

No.	Ret Time (min)	Area (mAU*min)	Rel.Area (%)
1	2.900	2535	3.54%
2	4.033	344	0.48%
3	5.065	68689	95.98%
Total		71568	

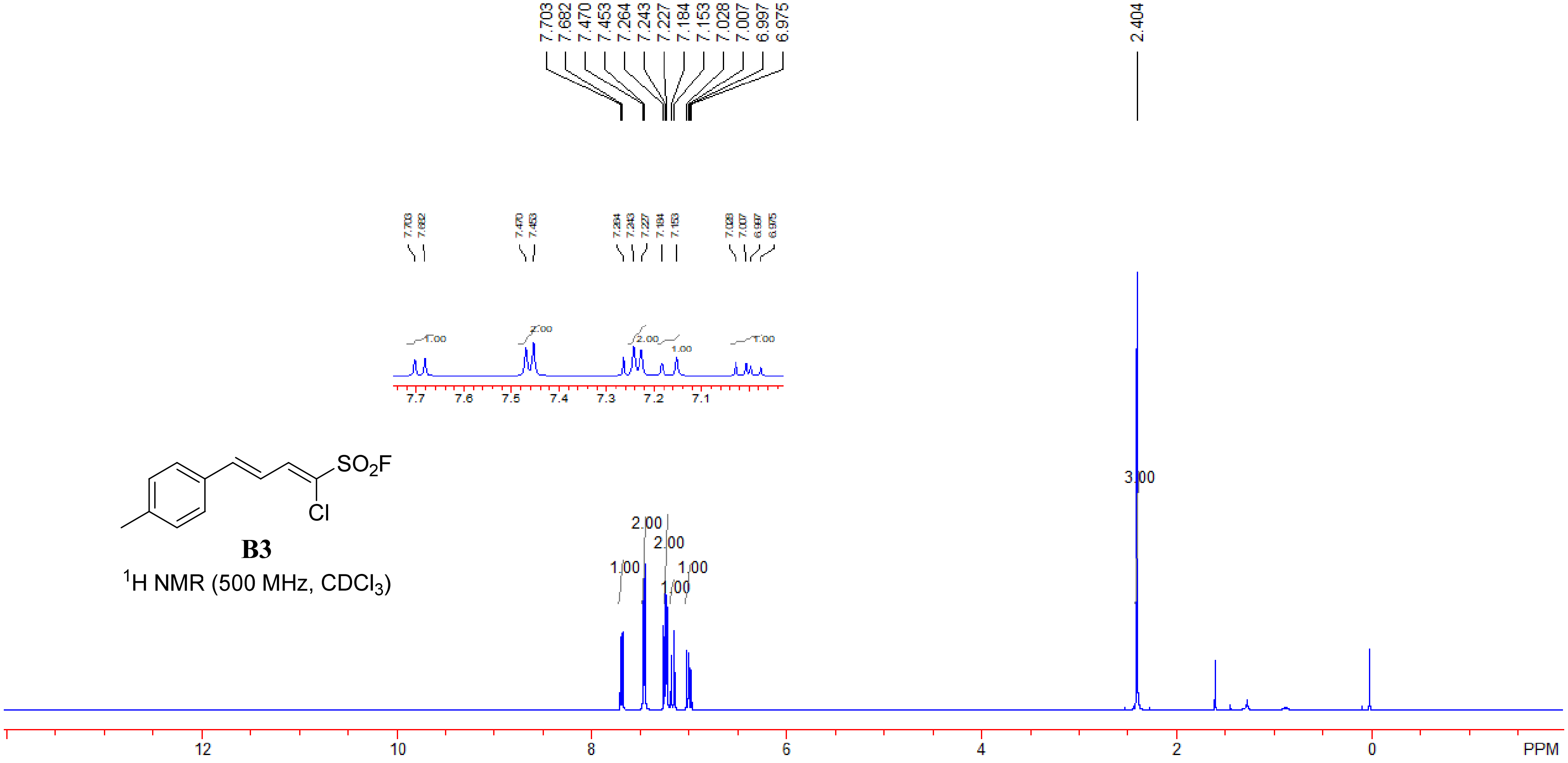

B3

^{1}H NMR (500 MHz, $CDCl_3$)

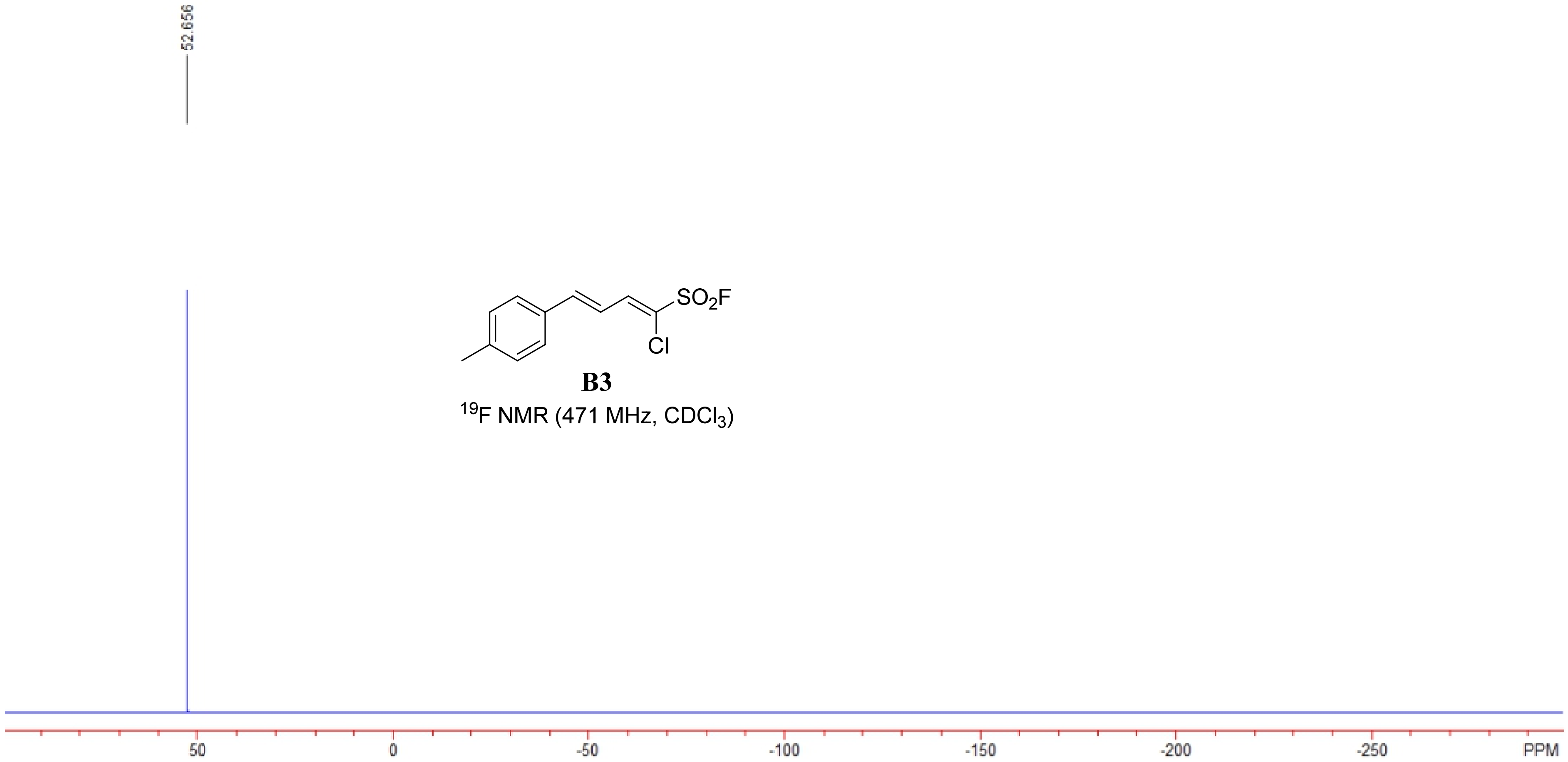
52.656
B3
^{19}F NMR (471 MHz, $CDCl_3$)
50
0
-50
-100
-150
-200
-250
PPM

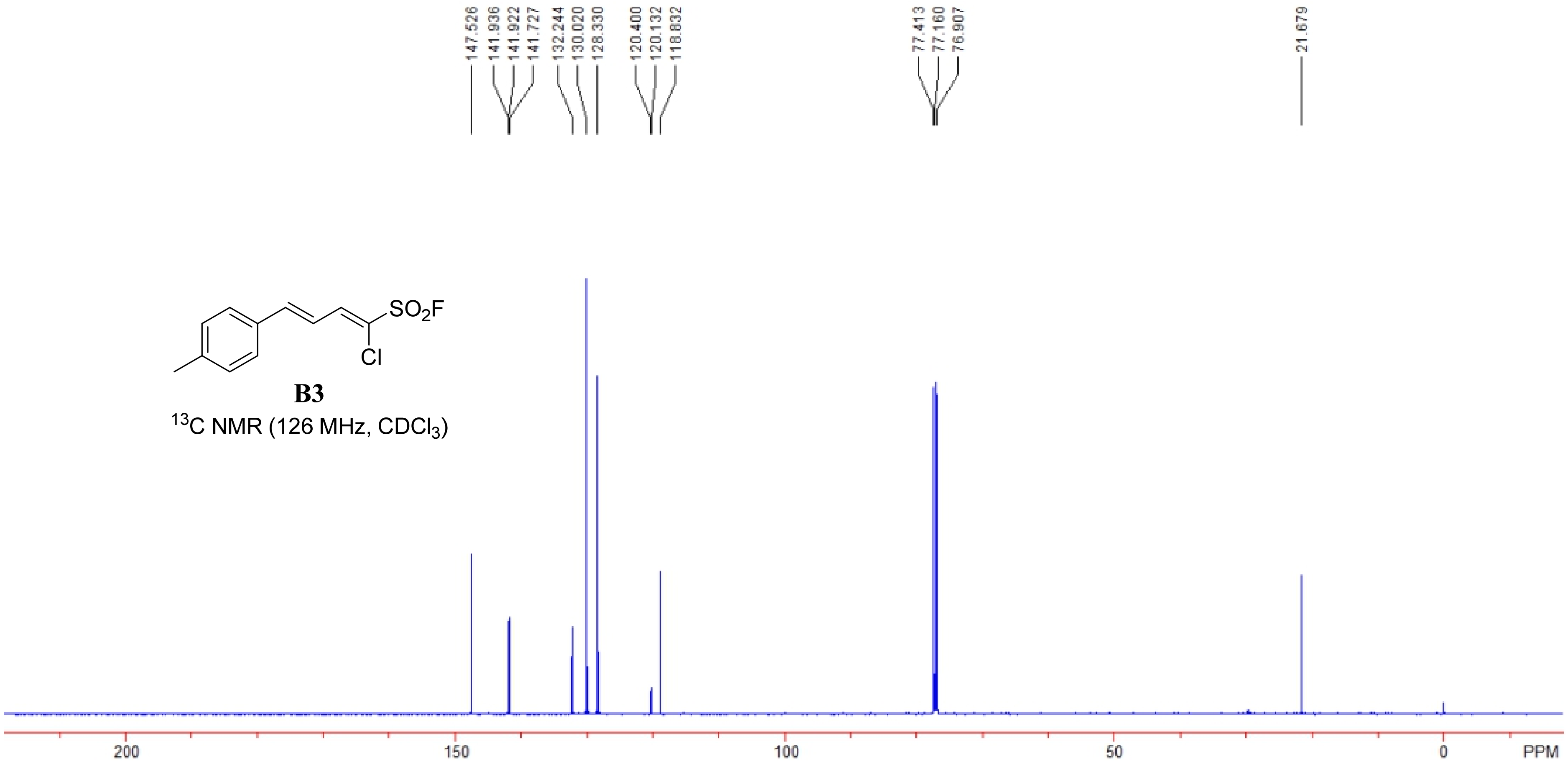

B3

^{13}C NMR (126 MHz, $CDCl_3$)

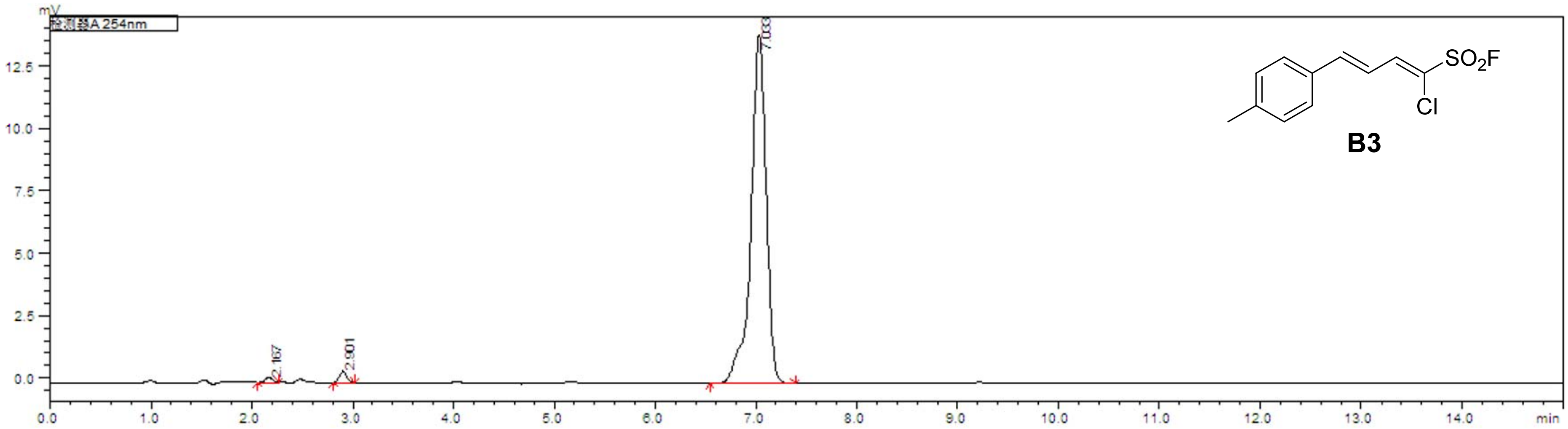

B3

No.	Ret Time (min)	Area (mAU*min)	Rel.Area (%)
1	2.167	1018	0.68%
2	2.901	2297	1.54%
3	7.033	146176	97.78%
Total		149491	

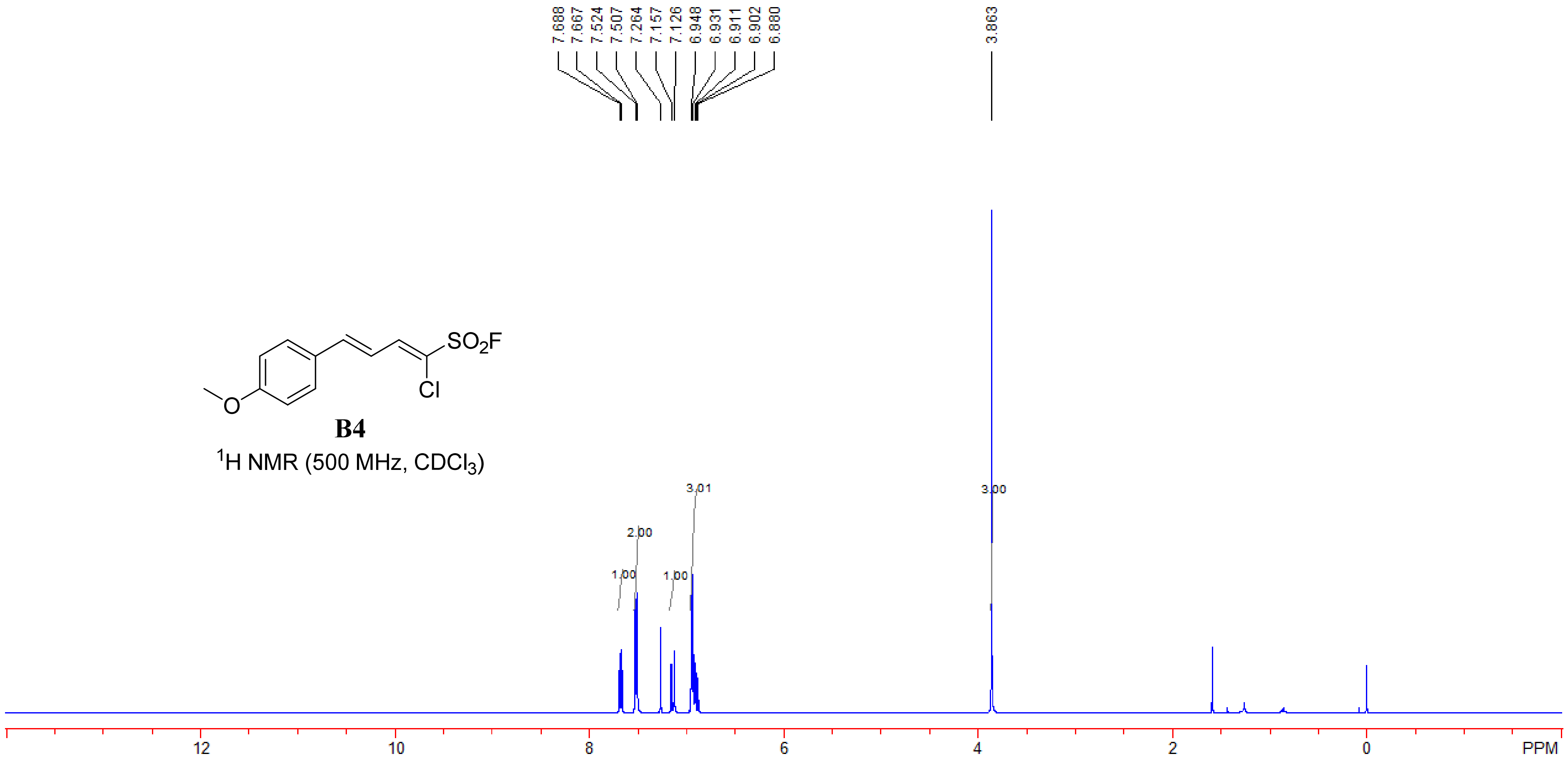

B4

^{1}H NMR (500 MHz, $CDCl_3$)

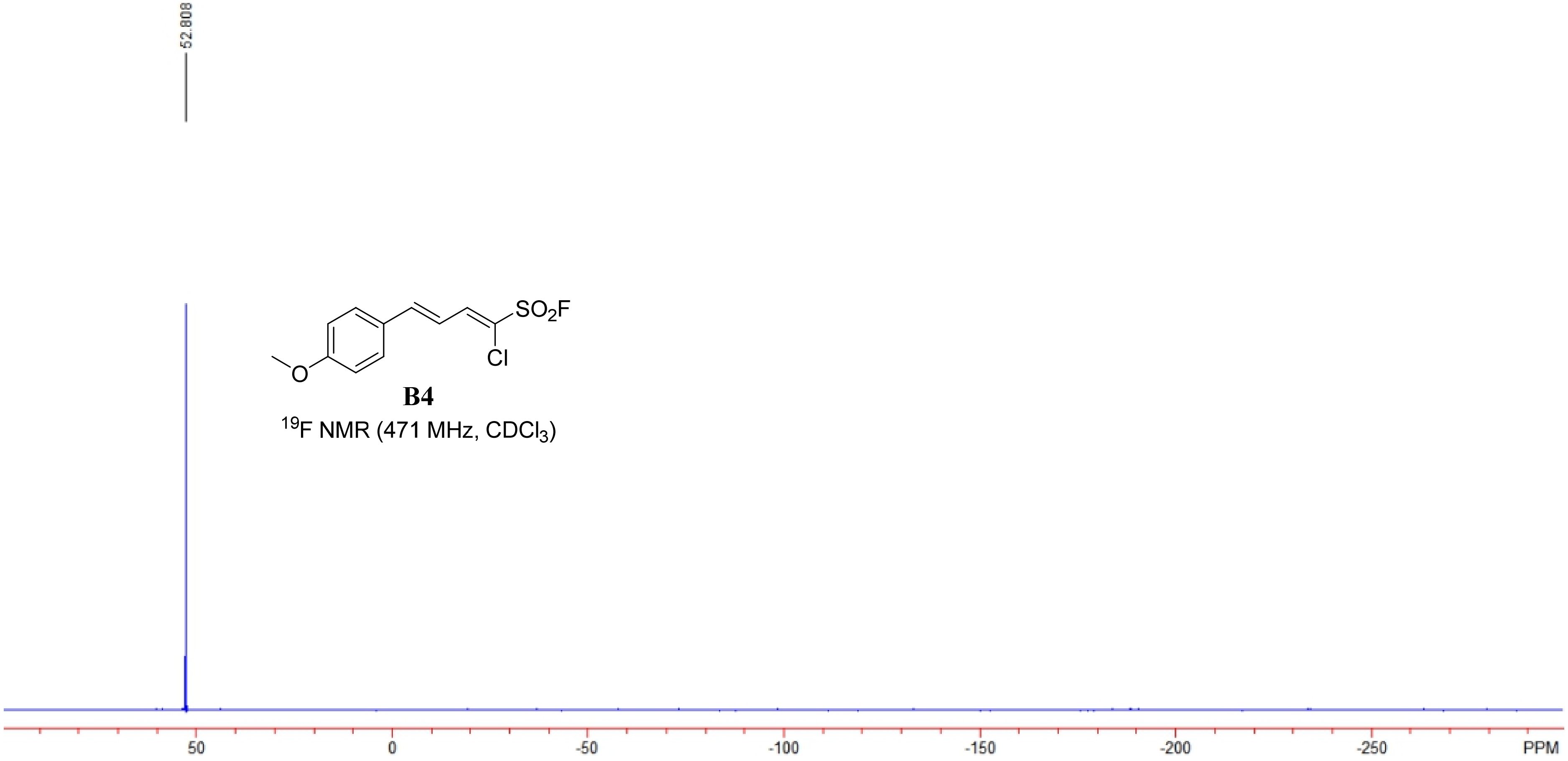

B4

^{19}F NMR (471 MHz, $CDCl_3$)

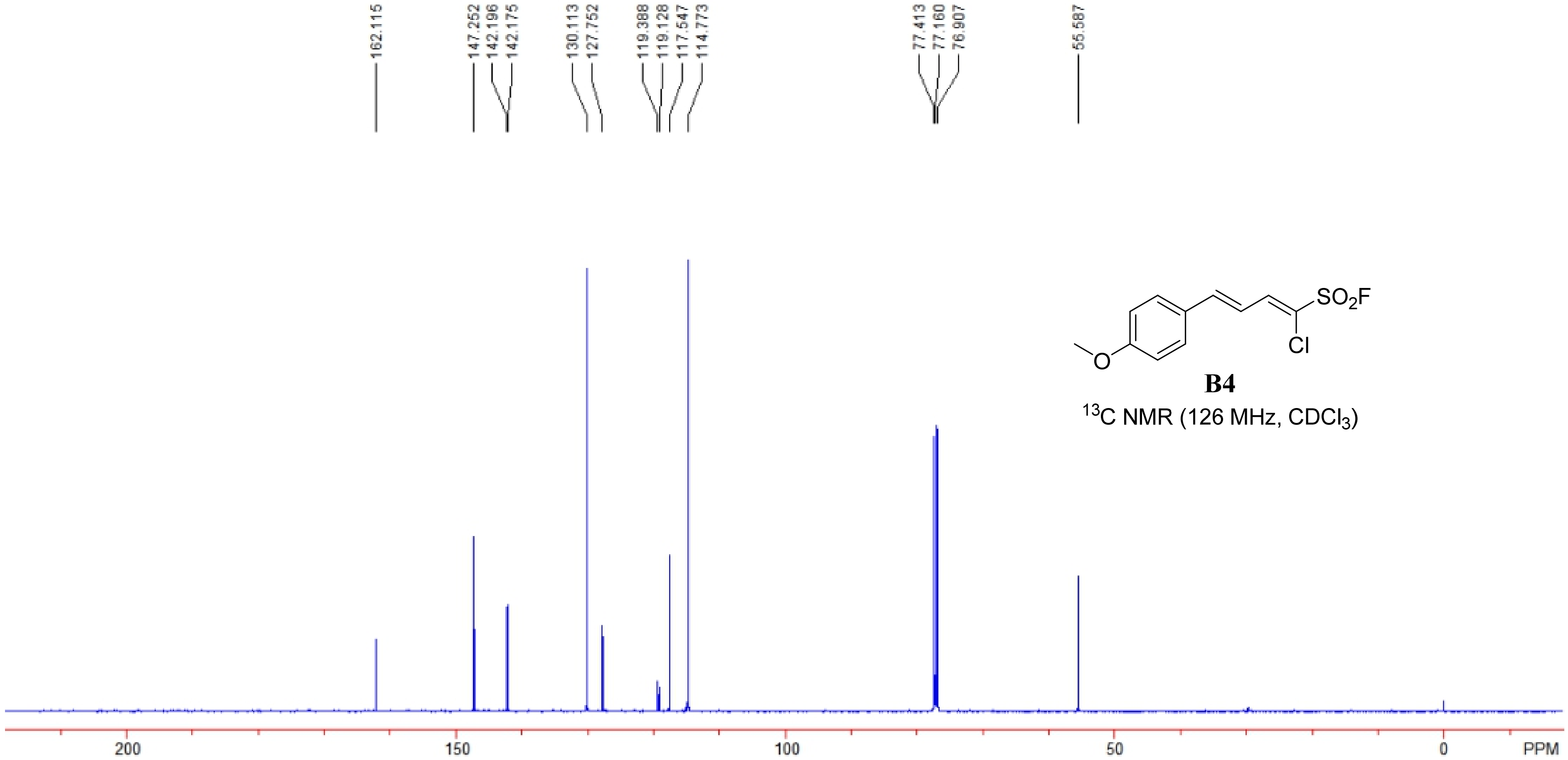
162.115
147.252
142.196
142.175
130.113
127.752
119.388
119.128
117.547
114.773
77.413
77.160
76.907
55.587
SO_2F
Cl
O
B4
^{13}C NMR (126 MHz, $CDCl_3$)
200
150
100
50
0
PPM

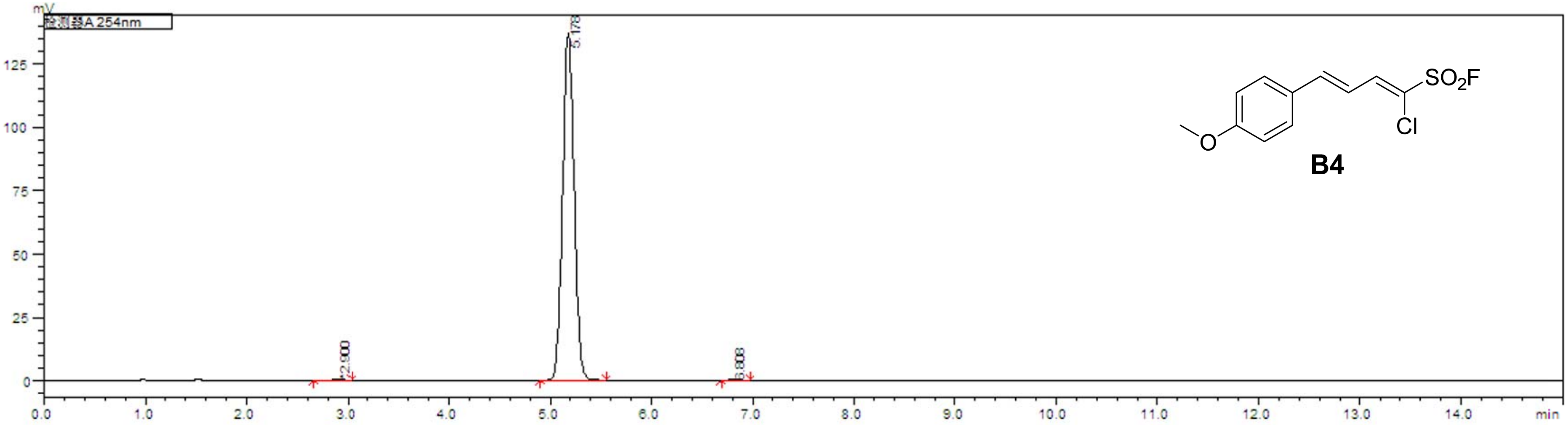

No.	Ret Time (min)	Area (mAU*min)	Rel.Area (%)
1	2.900	2063	0.19%
2	5.178	1091117	99.76%
3	6.808	585	0.05%
Total		1093765	

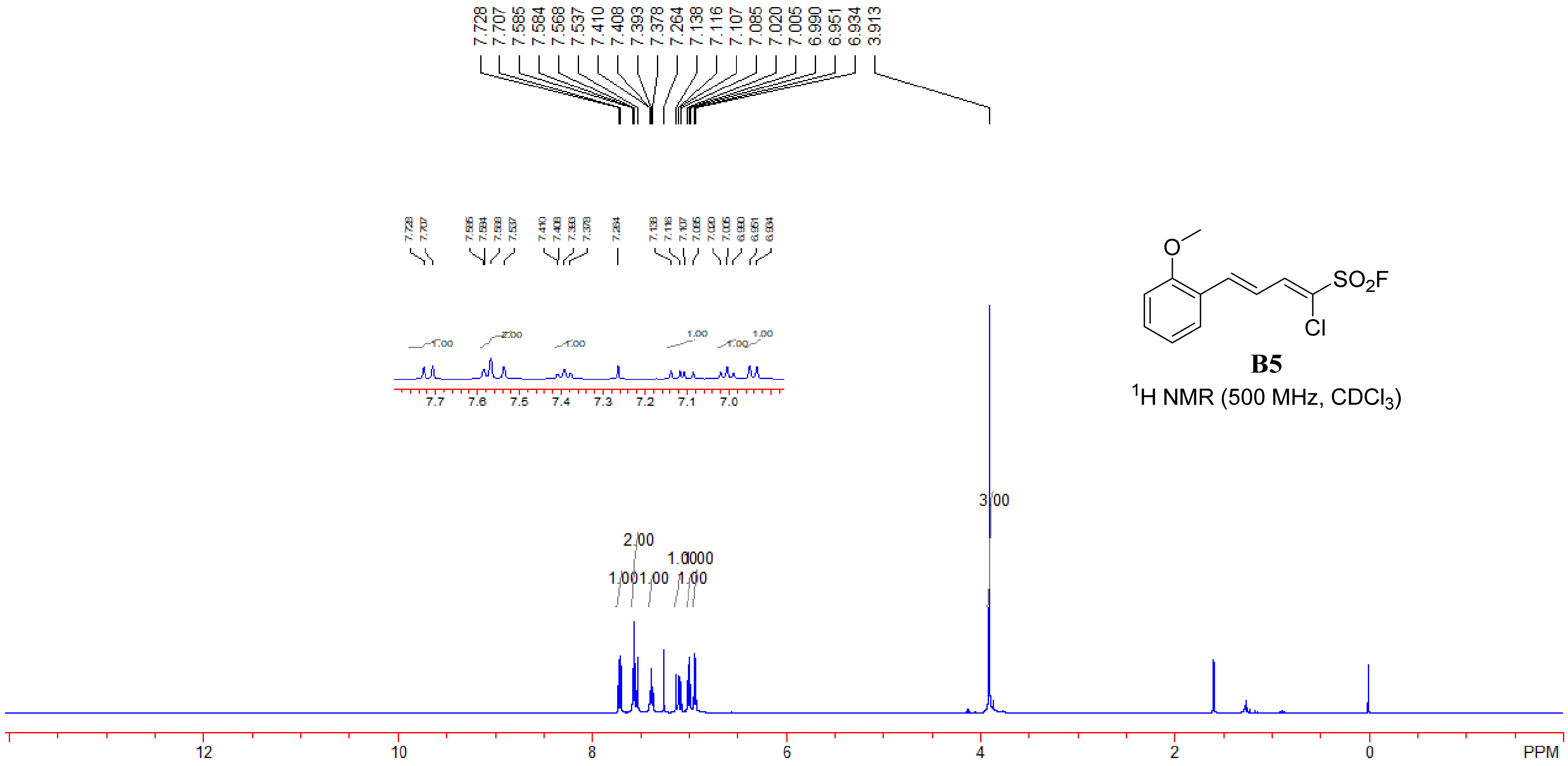

B5

^{1}H NMR (500 MHz, $CDCl_3$)

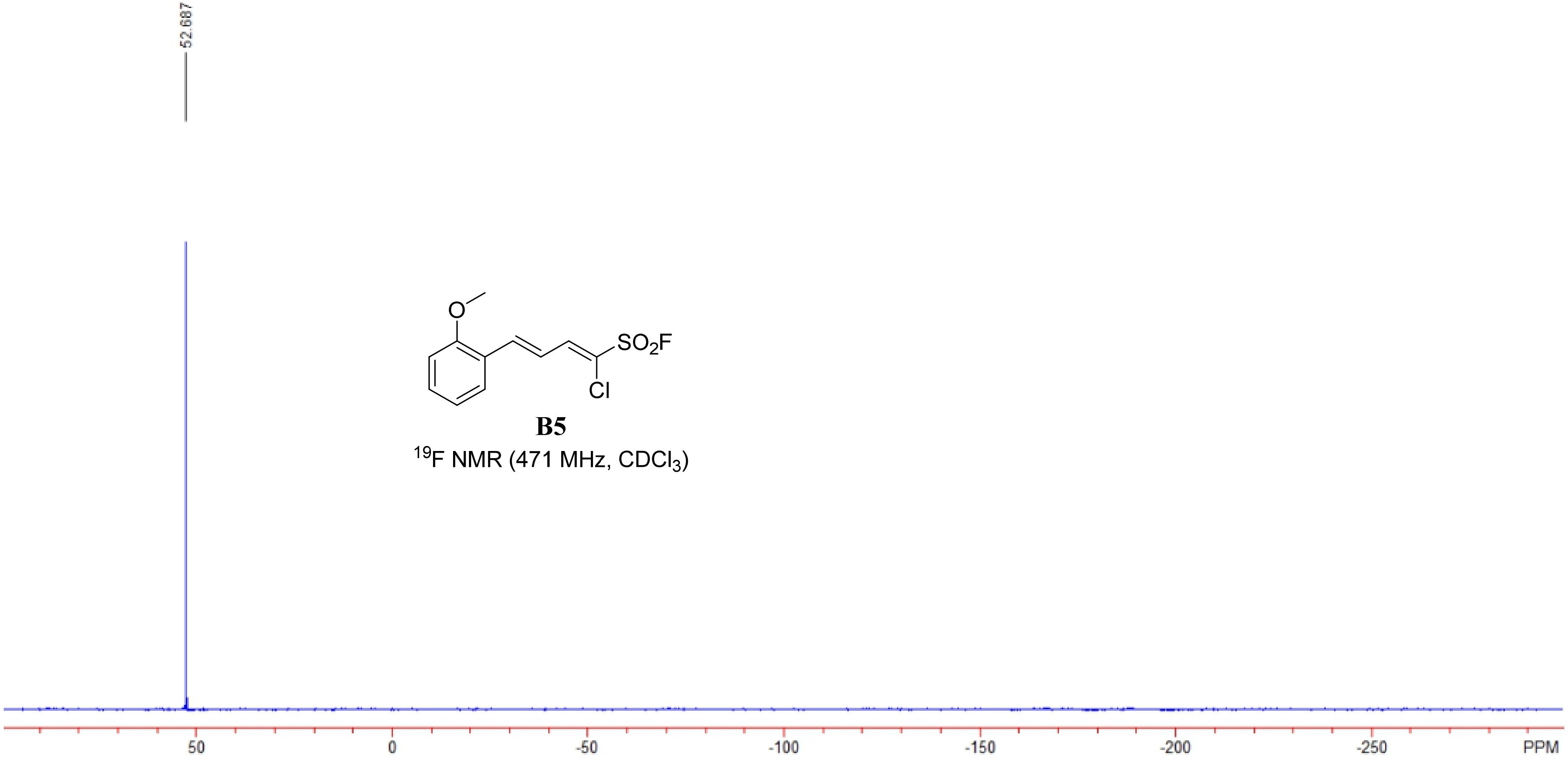

B5

^{19}F NMR (471 MHz, $CDCl_3$)

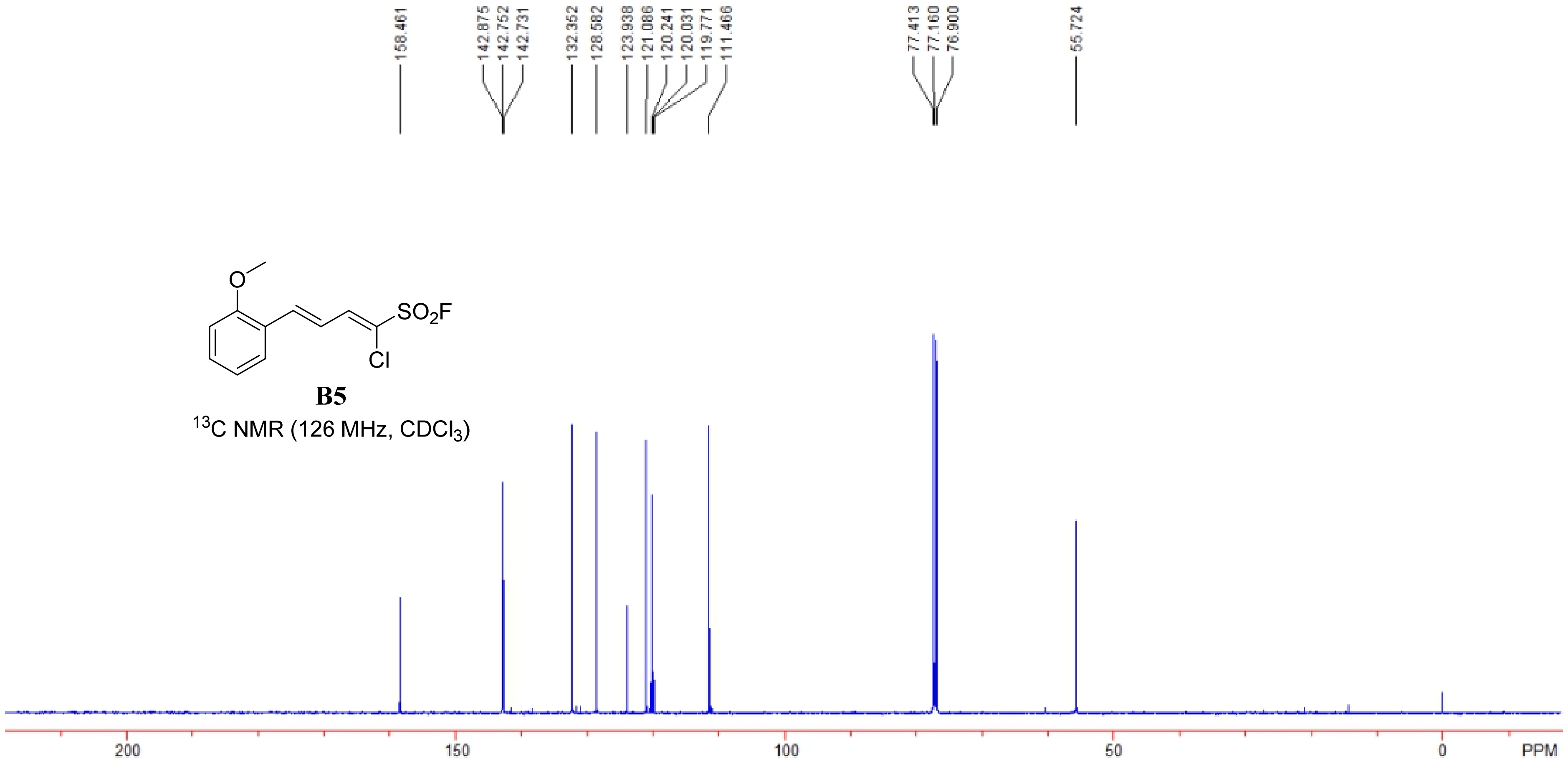

B5

^{13}C NMR (126 MHz, $CDCl_3$)

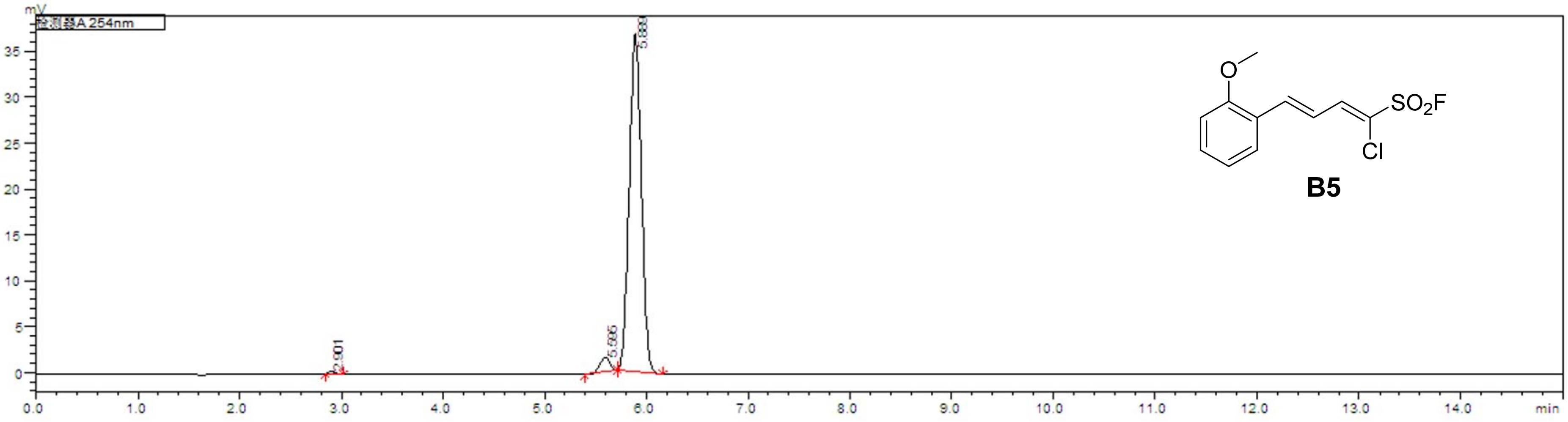

No.	Ret Time (min)	Area (mAU*min)	Rel.Area (%)
1	2.901	1420	0.45%
2	5.595	10111	3.19%
3	5.889	305103	96.36%
Total		316634	

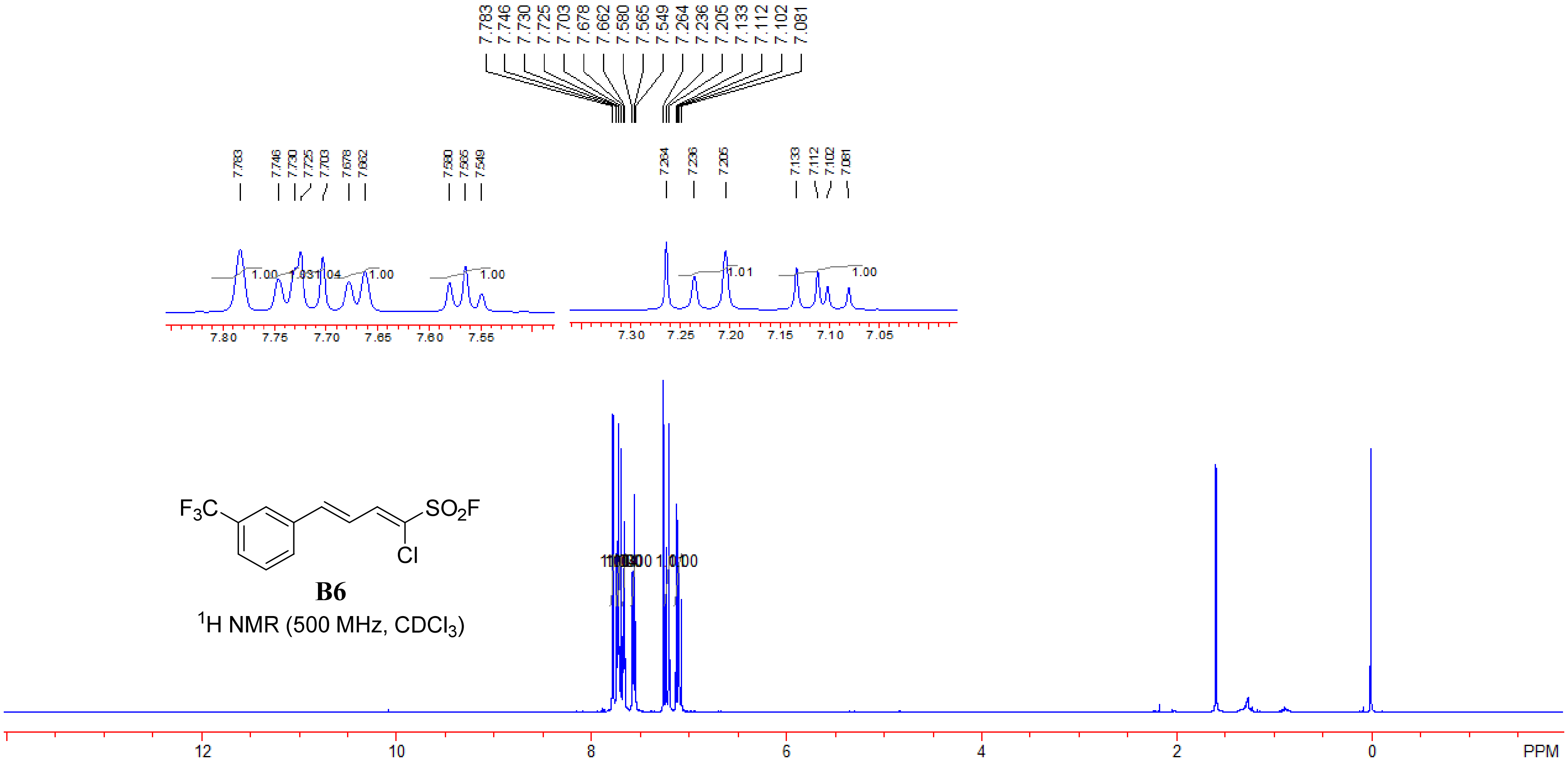
7.783
7.746
7.730
7.725
7.703
7.678
7.662
7.580
7.565
7.549
7.264
7.236
7.205
7.133
7.112
7.102
7.081
1.00
1.03
1.04
1.00
1.00
1.01
1.00
F3C
SO2F
Cl
B6
1H NMR (500 MHz, CDCl3)
12
10
8
6
4
2
0
PPM

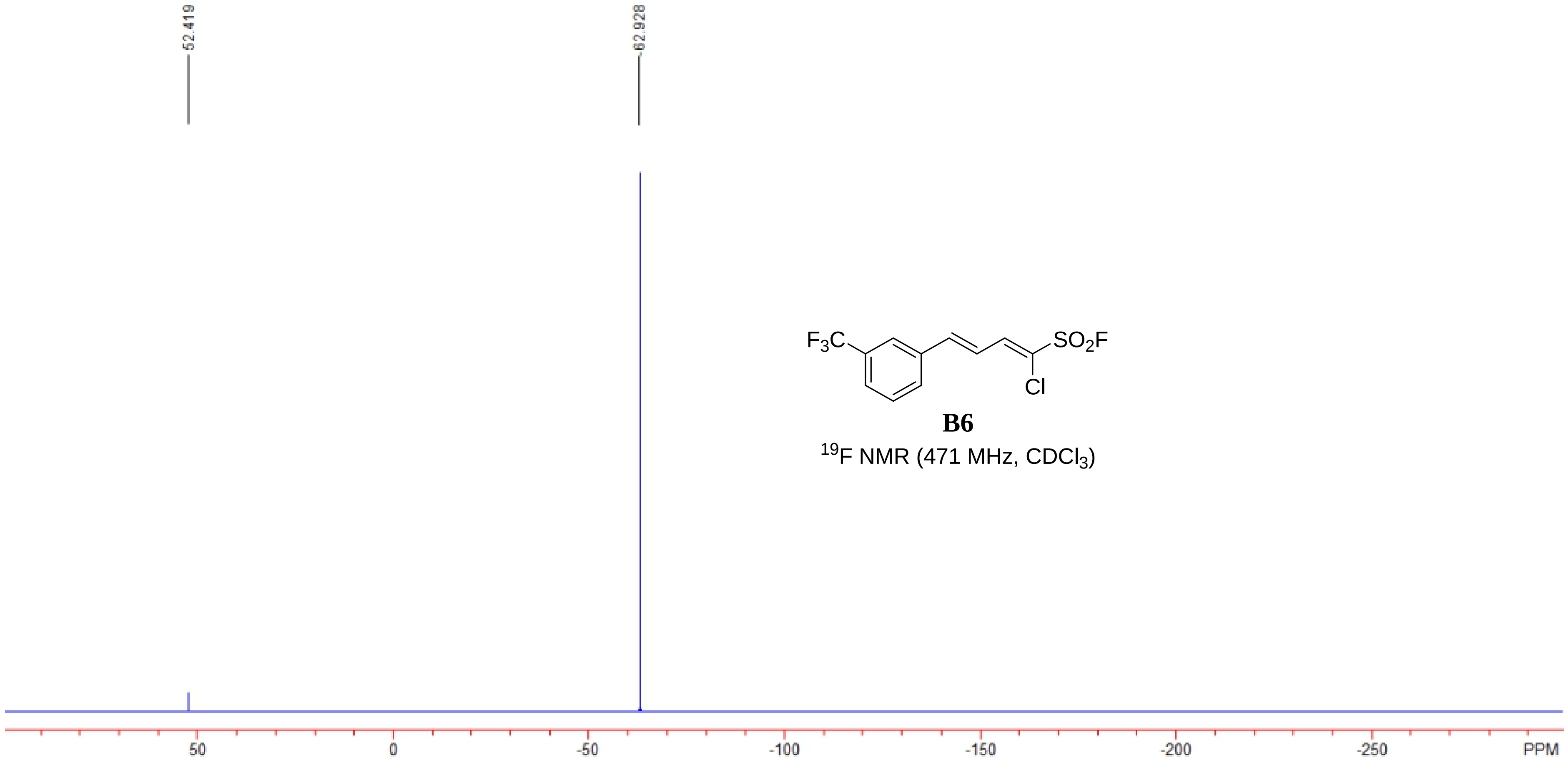

B6

^{19}F NMR (471 MHz, $CDCl_3$)

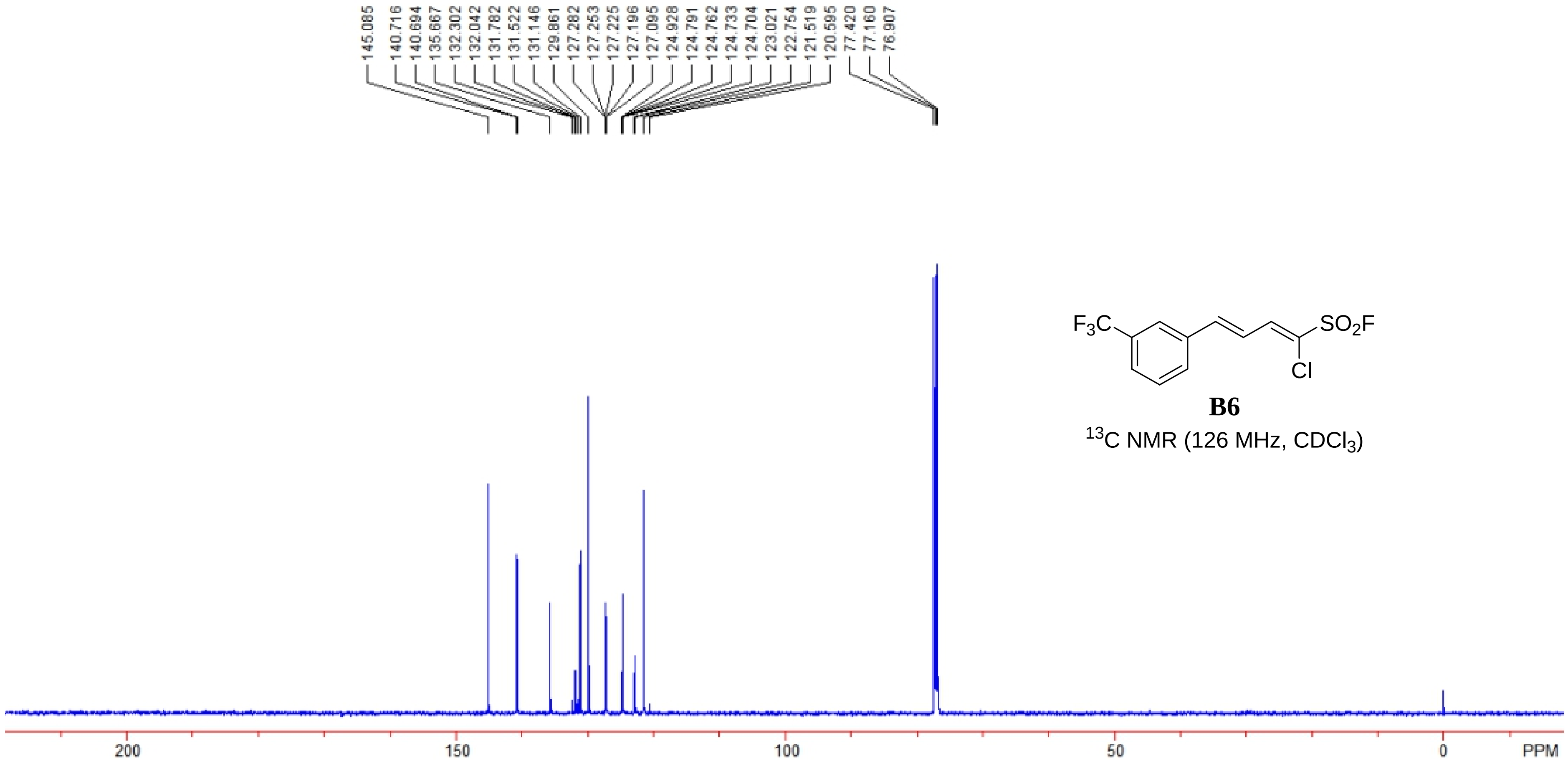

B6

^{13}C NMR (126 MHz, $CDCl_3$)

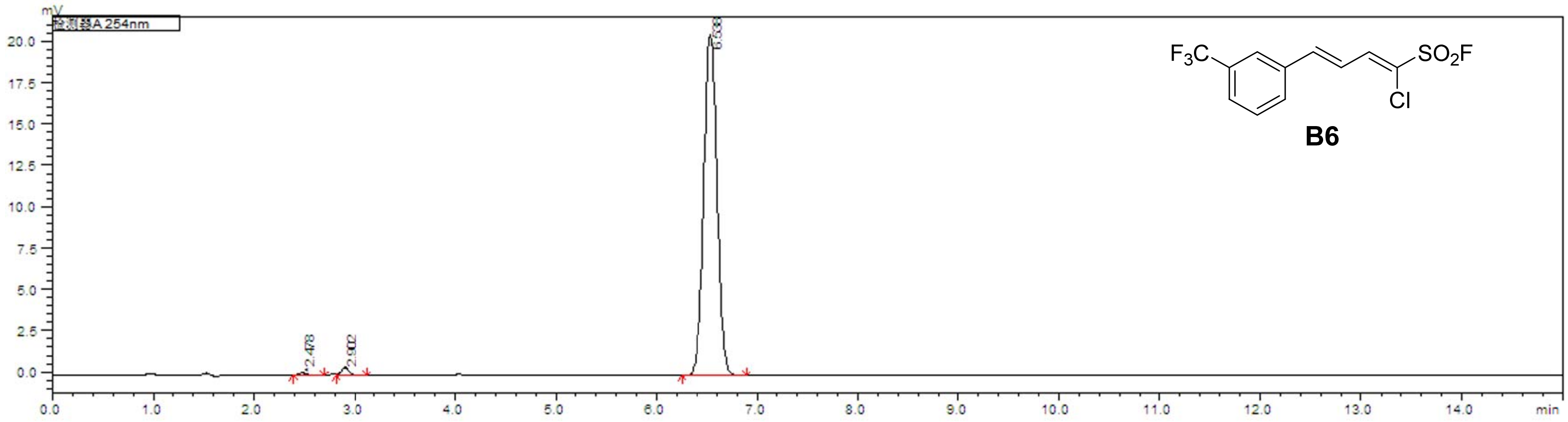

No.	Ret Time (min)	Area (mAU*min)	Rel.Area (%)
1	2.478	1084	0.56%
2	2.902	2644	1.37%
3	6.538	189854	98.07%
Total		193582	

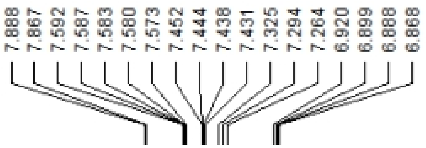

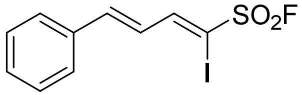

C1

^{1}H NMR (500 MHz, $CDCl_3$)

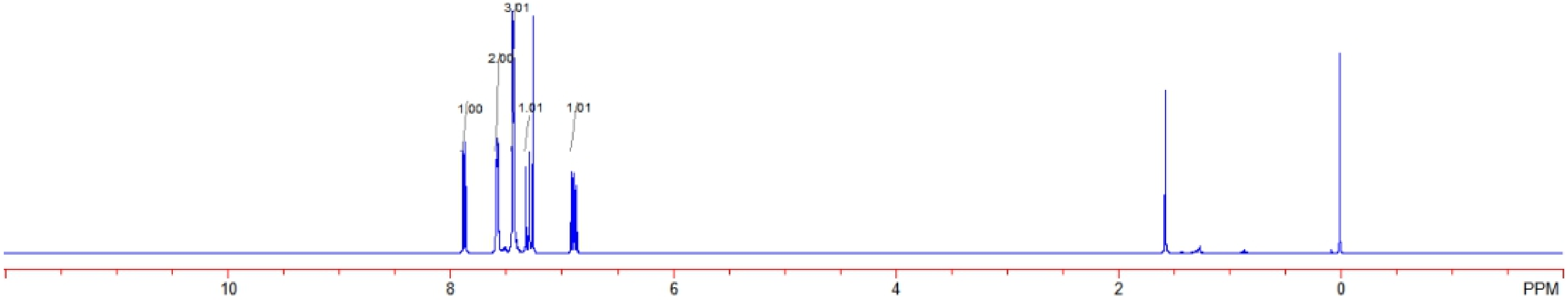

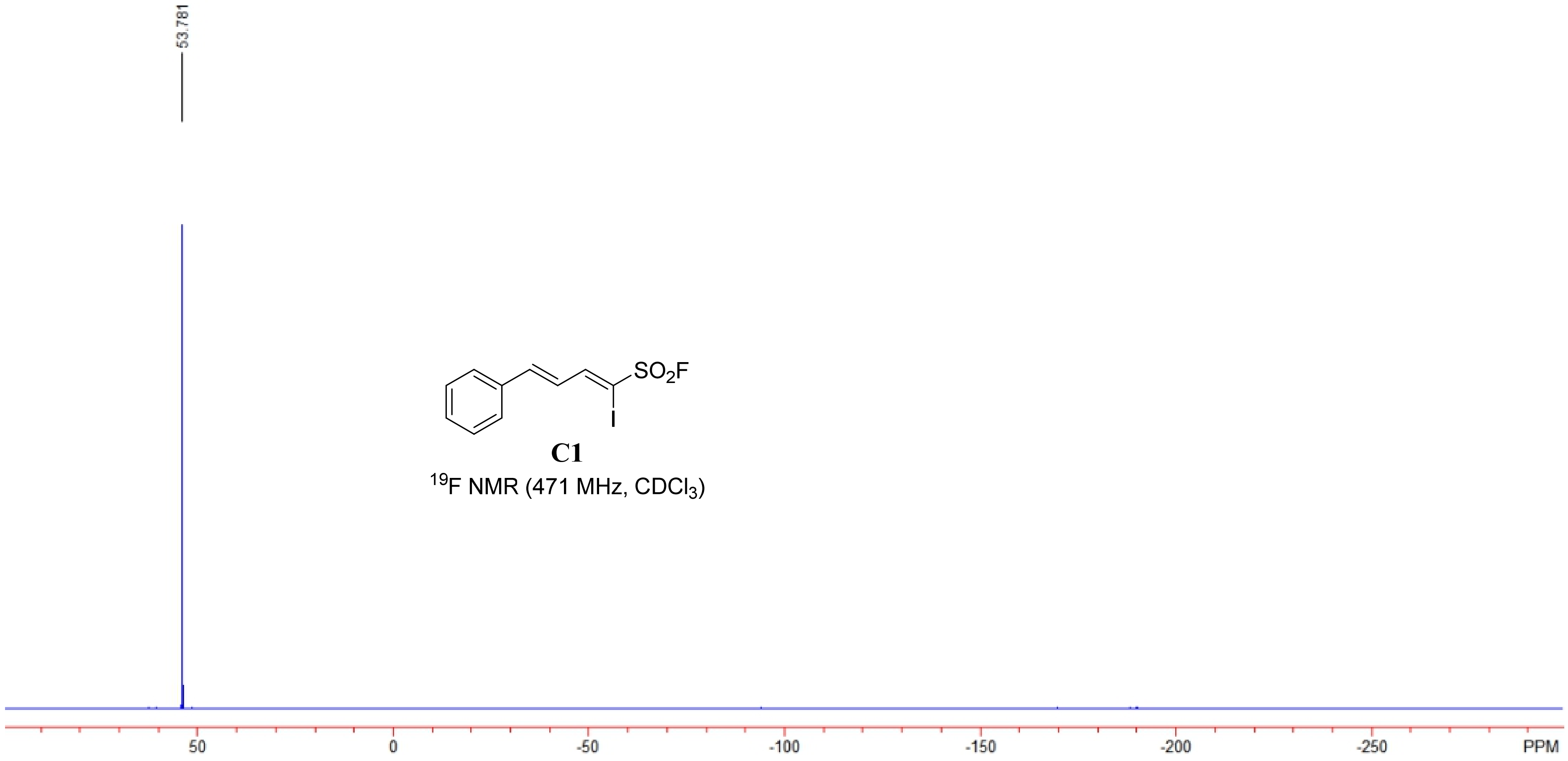

C1

^{19}F NMR (471 MHz, $CDCl_3$)

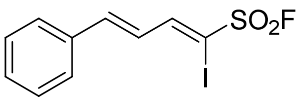

C1

^{13}C NMR (126 MHz, $CDCl_3$)

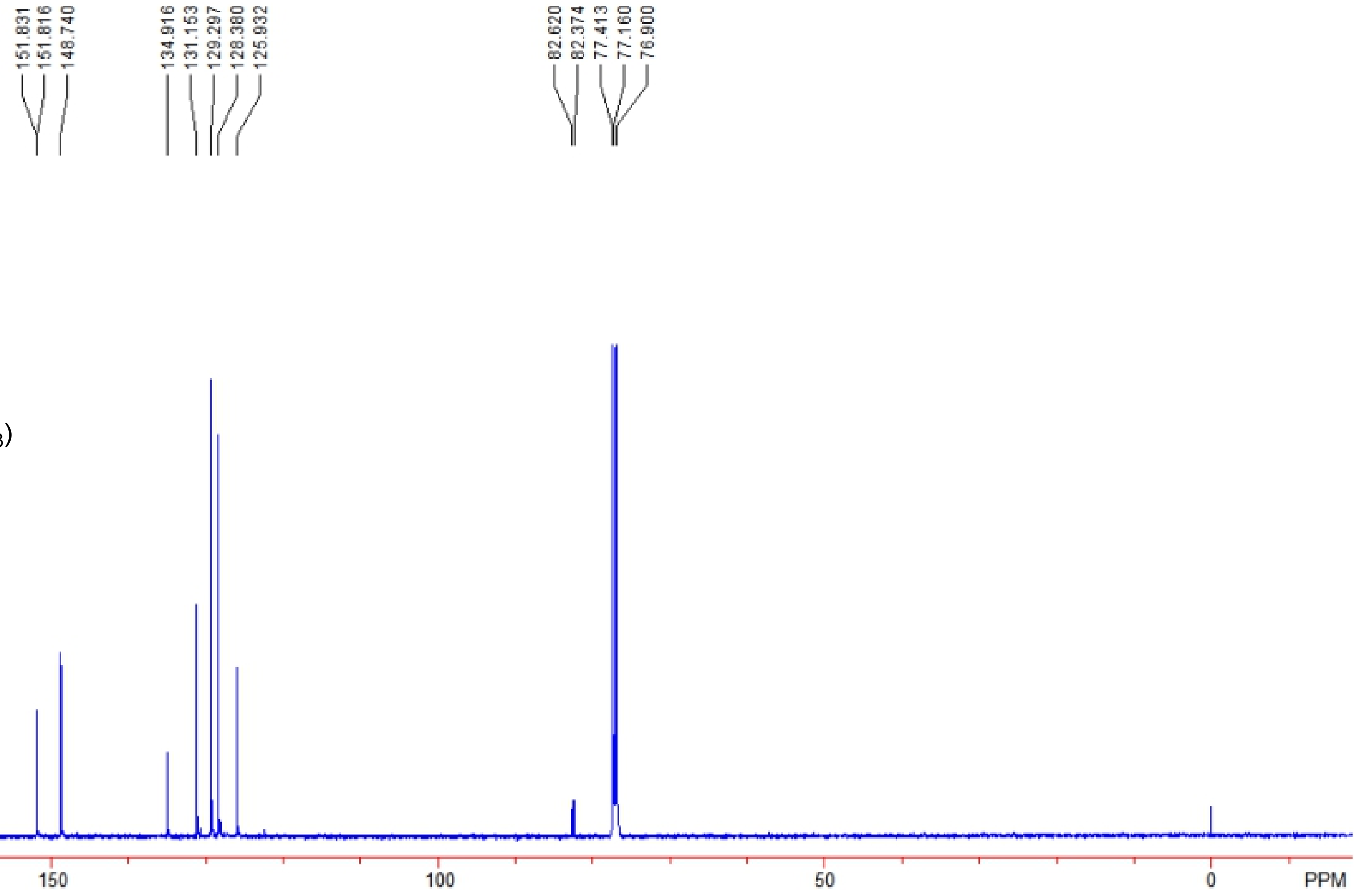

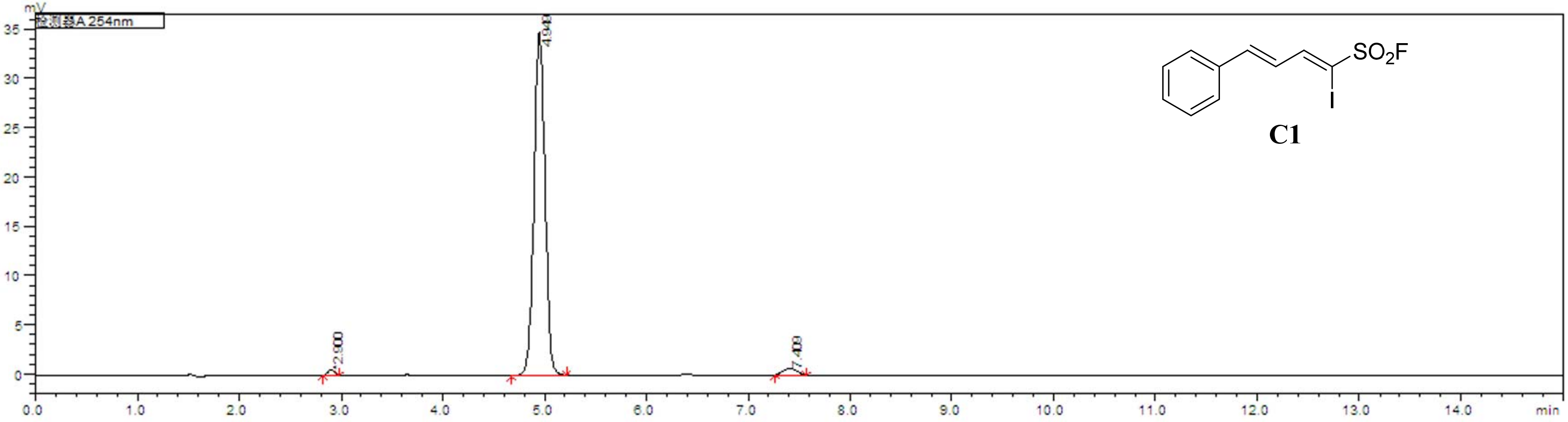

C1

No.	Ret Time (min)	Area (mAU*min)	Rel.Area (%)
1	2.900	2747	1.01%
2	4.949	261657	96.48%
3	7.409	6794	2.51%
Total		271198	

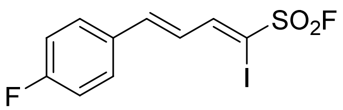

C2

^{1}H NMR (500 MHz, $CDCl_3$)

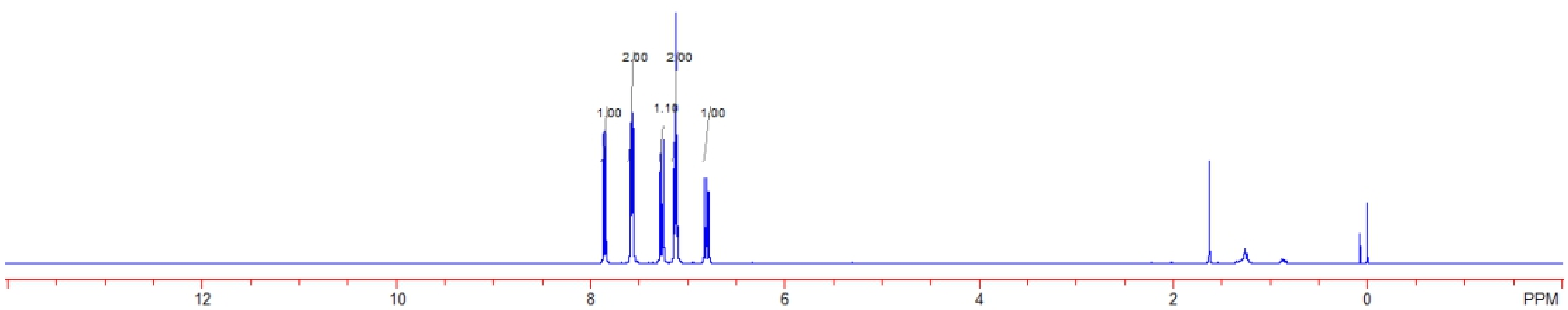

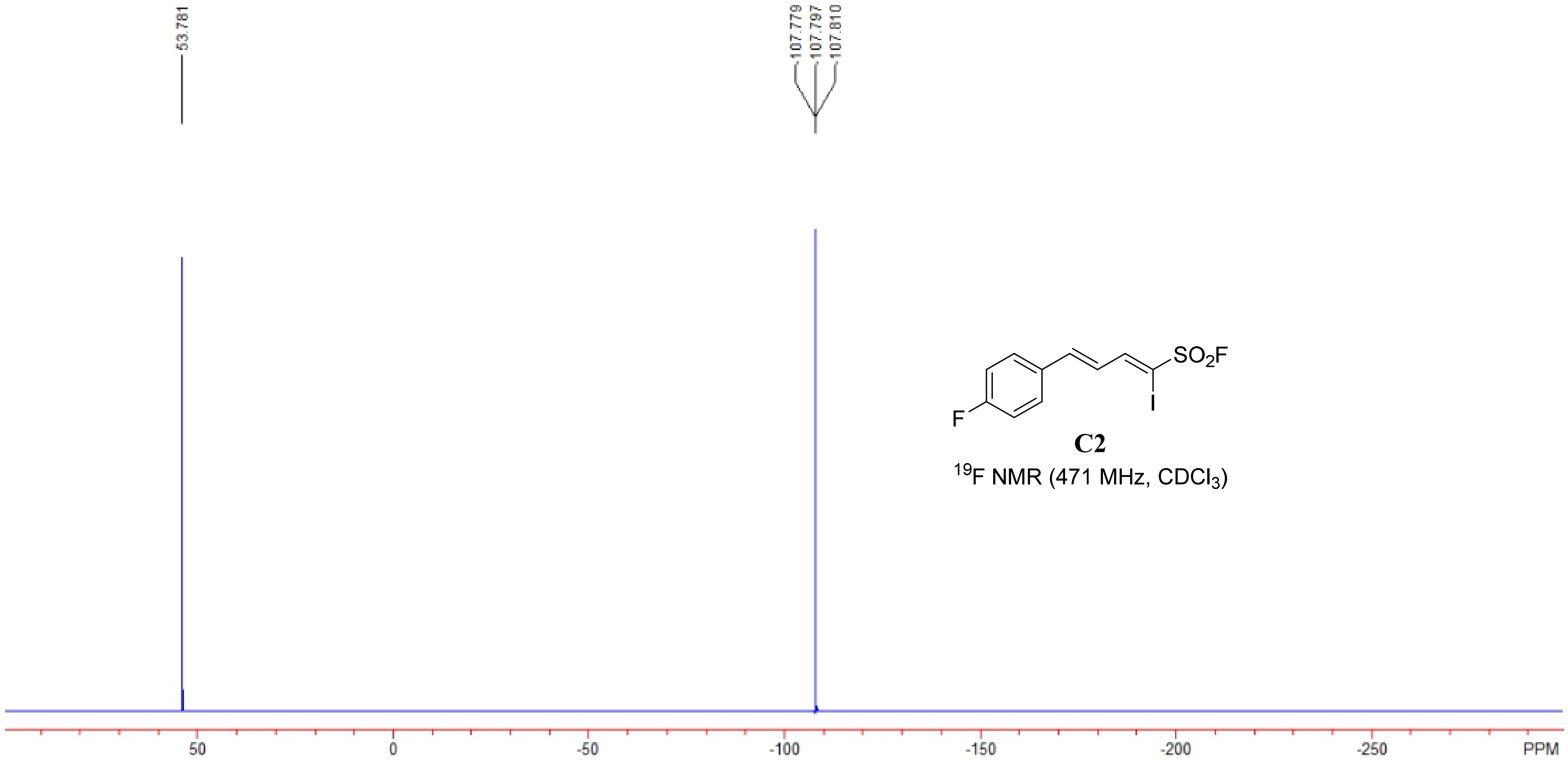

C2

^{19}F NMR (471 MHz, $CDCl_3$)

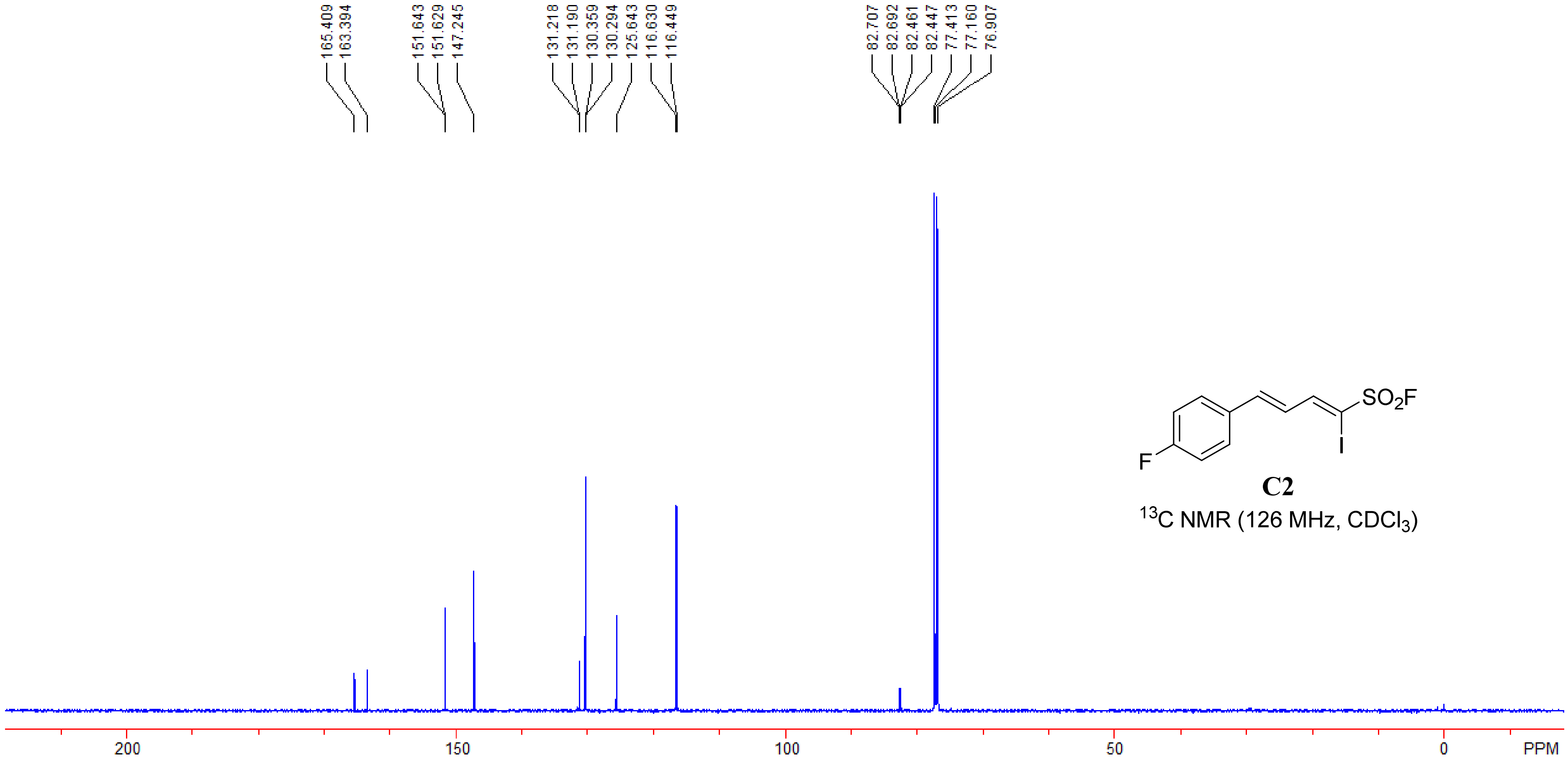

C2

^{13}C NMR (126 MHz, $CDCl_3$)

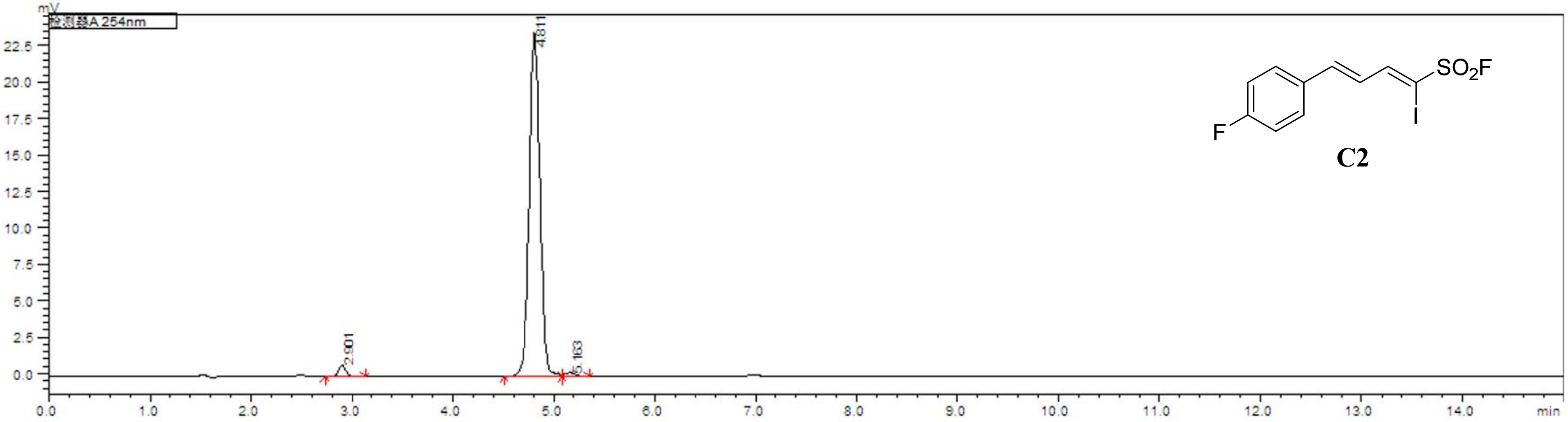

No.	Ret Time (min)	Area (mAU*min)	Rel.Area (%)
1	2.901	4139	2.27%
2	4.811	176221	96.58%
3	5.163	2093	1.15%
Total		182453	

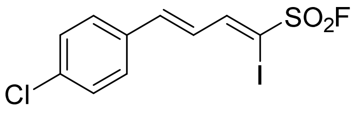

C3

^{1}H NMR (500 MHz, $CDCl_3$)

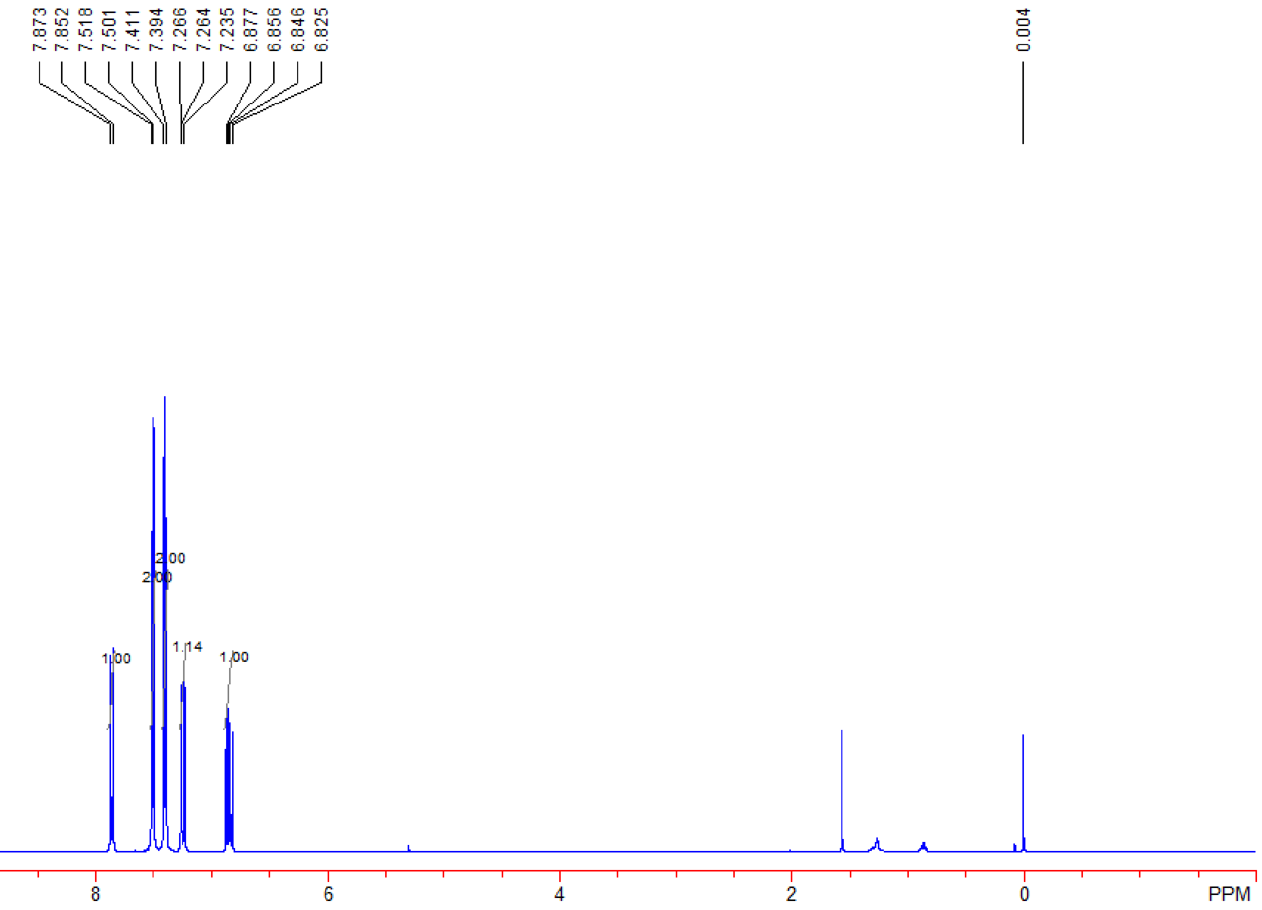

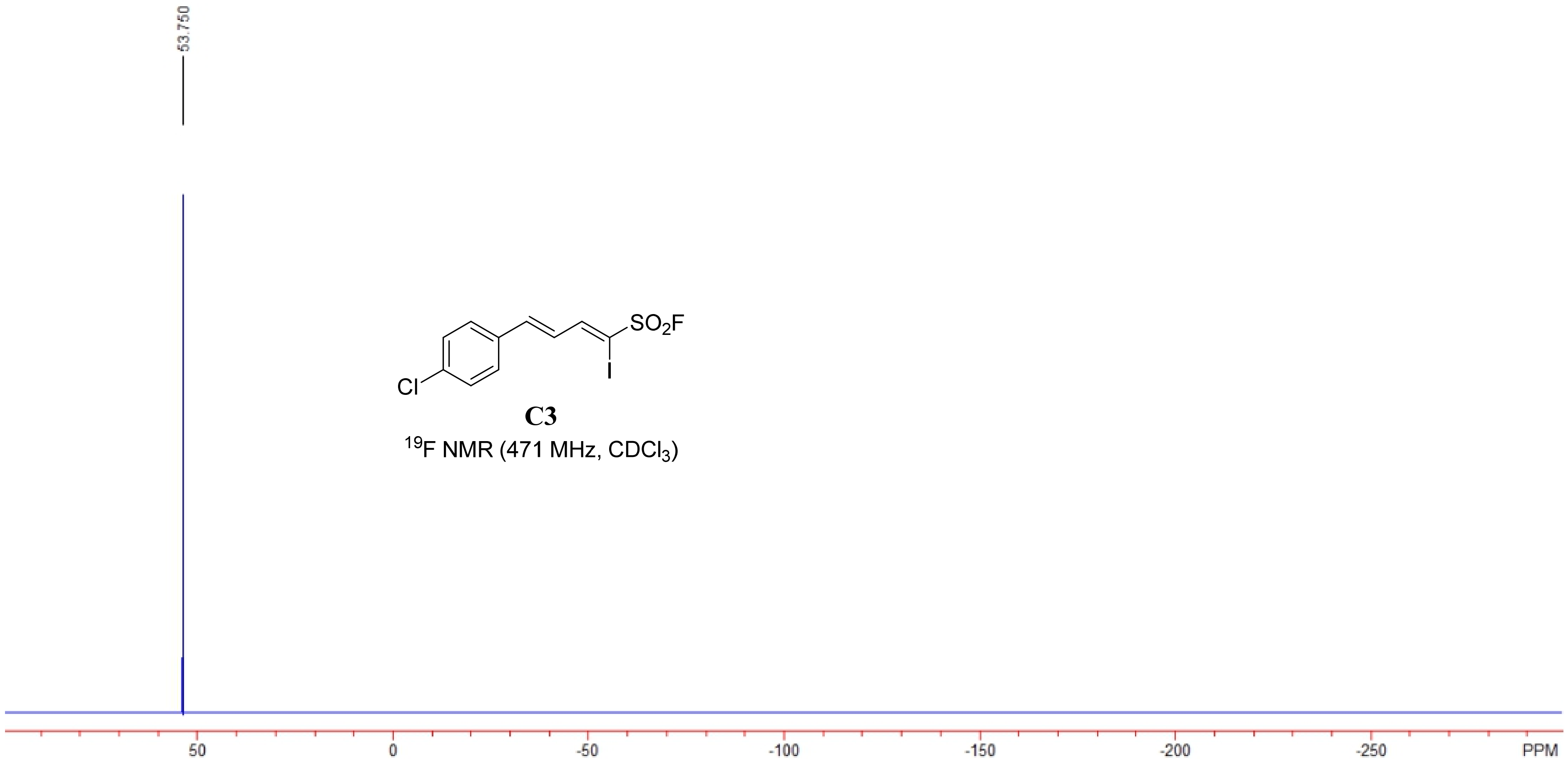

C3

^{19}F NMR (471 MHz, $CDCl_3$)

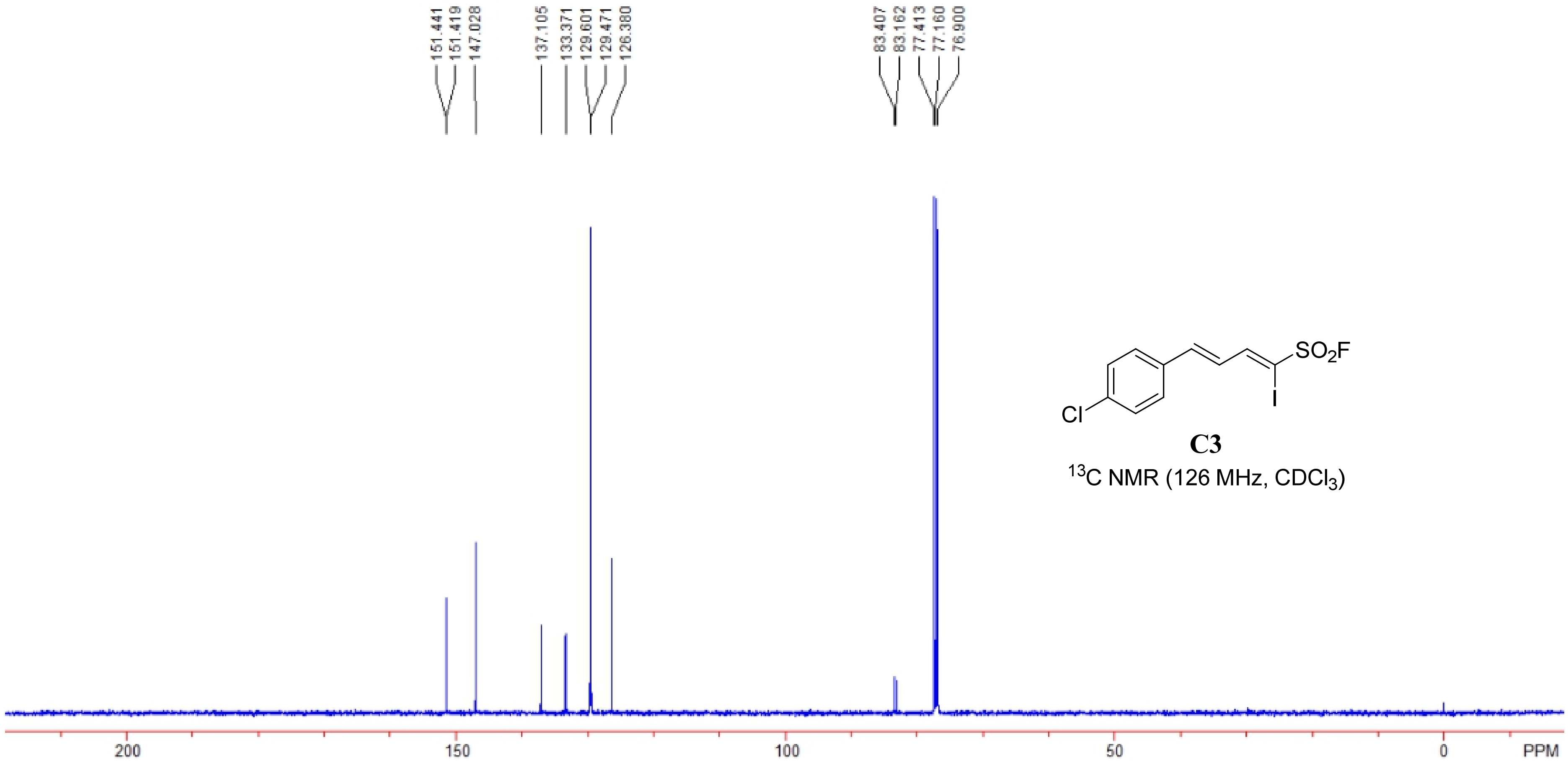

C3

^{13}C NMR (126 MHz, $CDCl_3$)

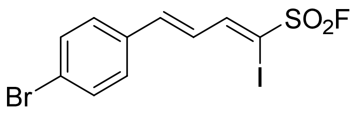

C4

^{1}H NMR (500 MHz, $CDCl_3$)

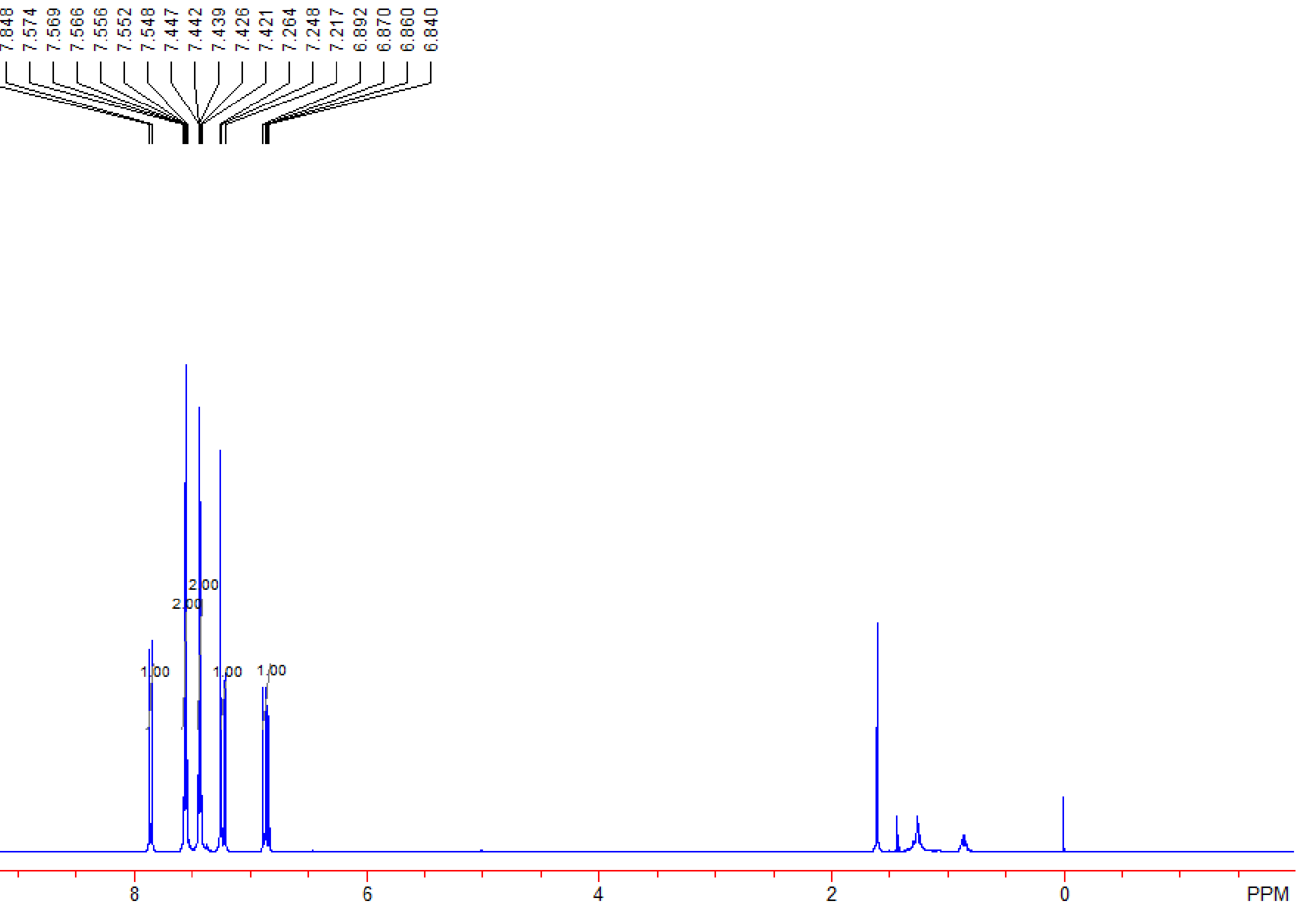

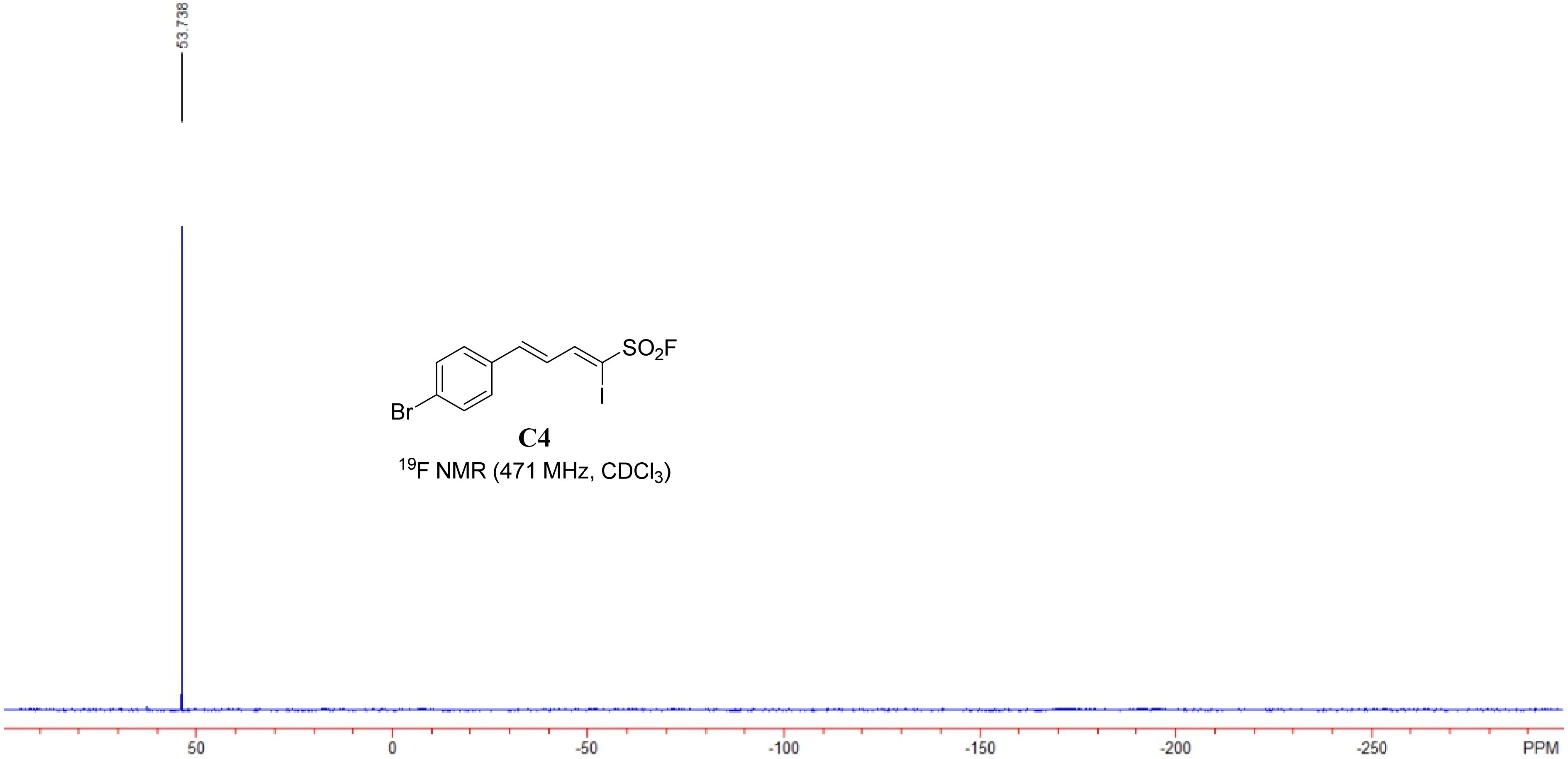

C4

^{19}F NMR (471 MHz, $CDCl_3$)

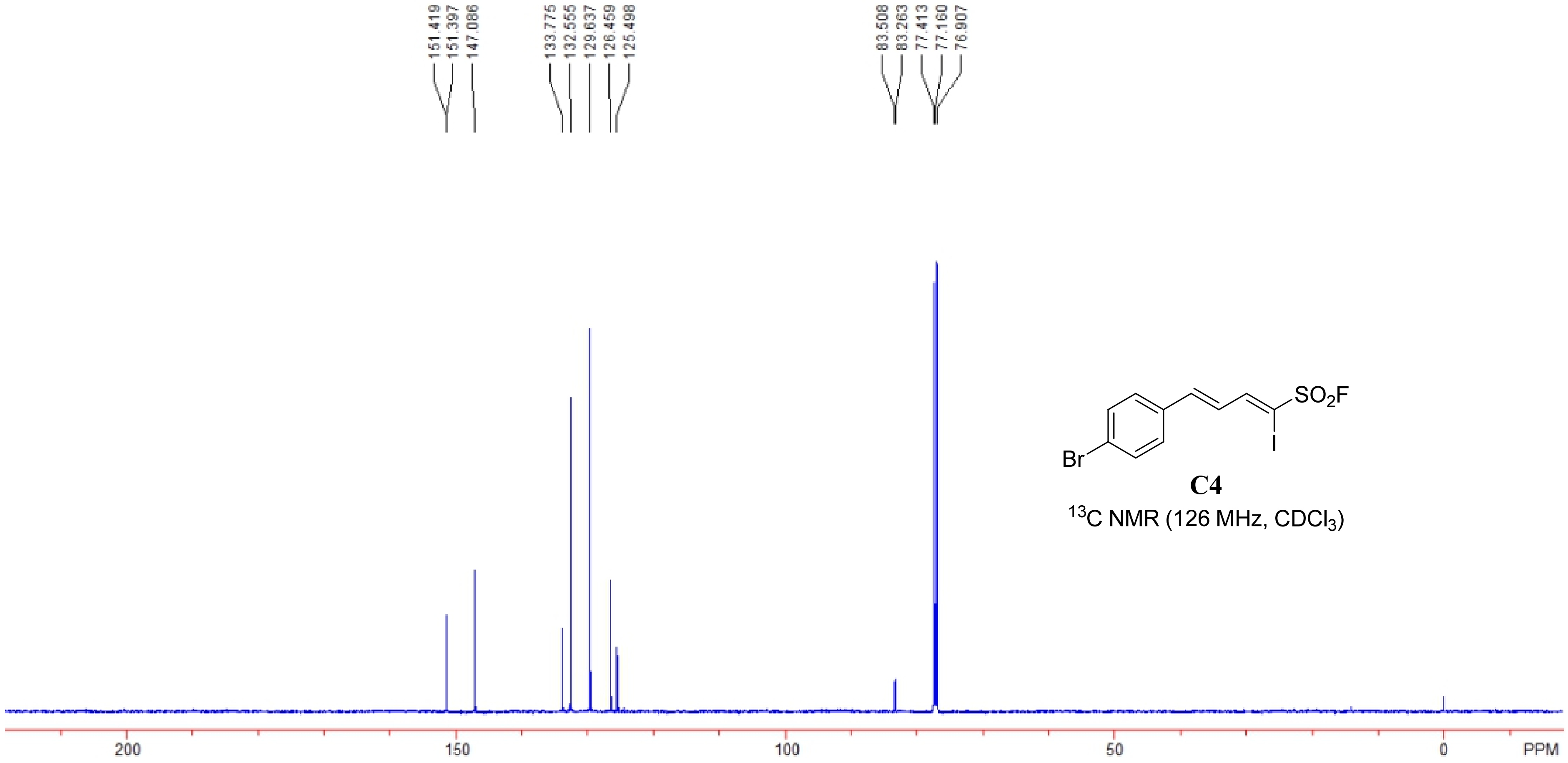

C4

^{13}C NMR (126 MHz, $CDCl_3$)

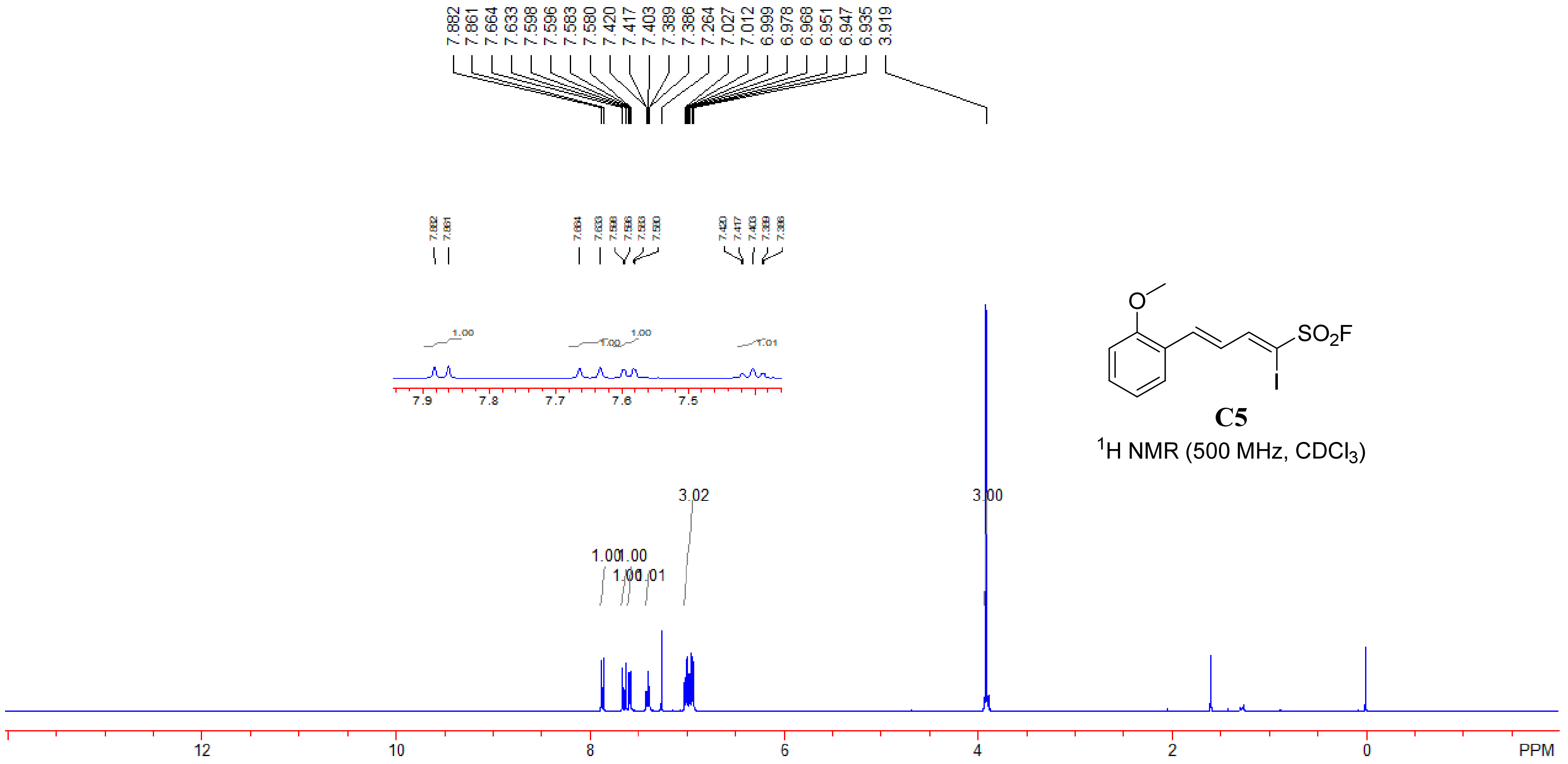

C5

^{1}H NMR (500 MHz, $CDCl_3$)

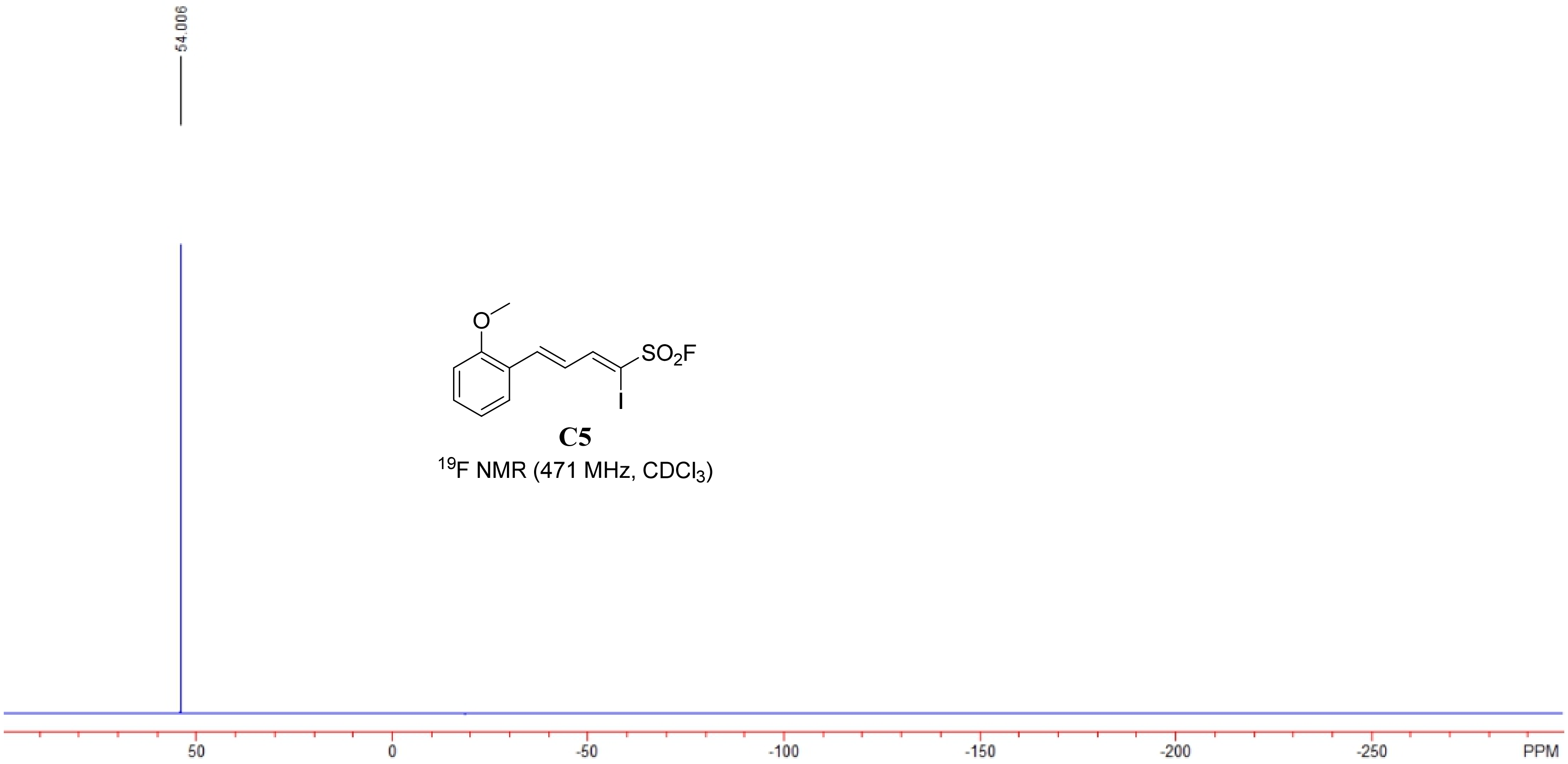

C5

^{19}F NMR (471 MHz, $CDCl_3$)

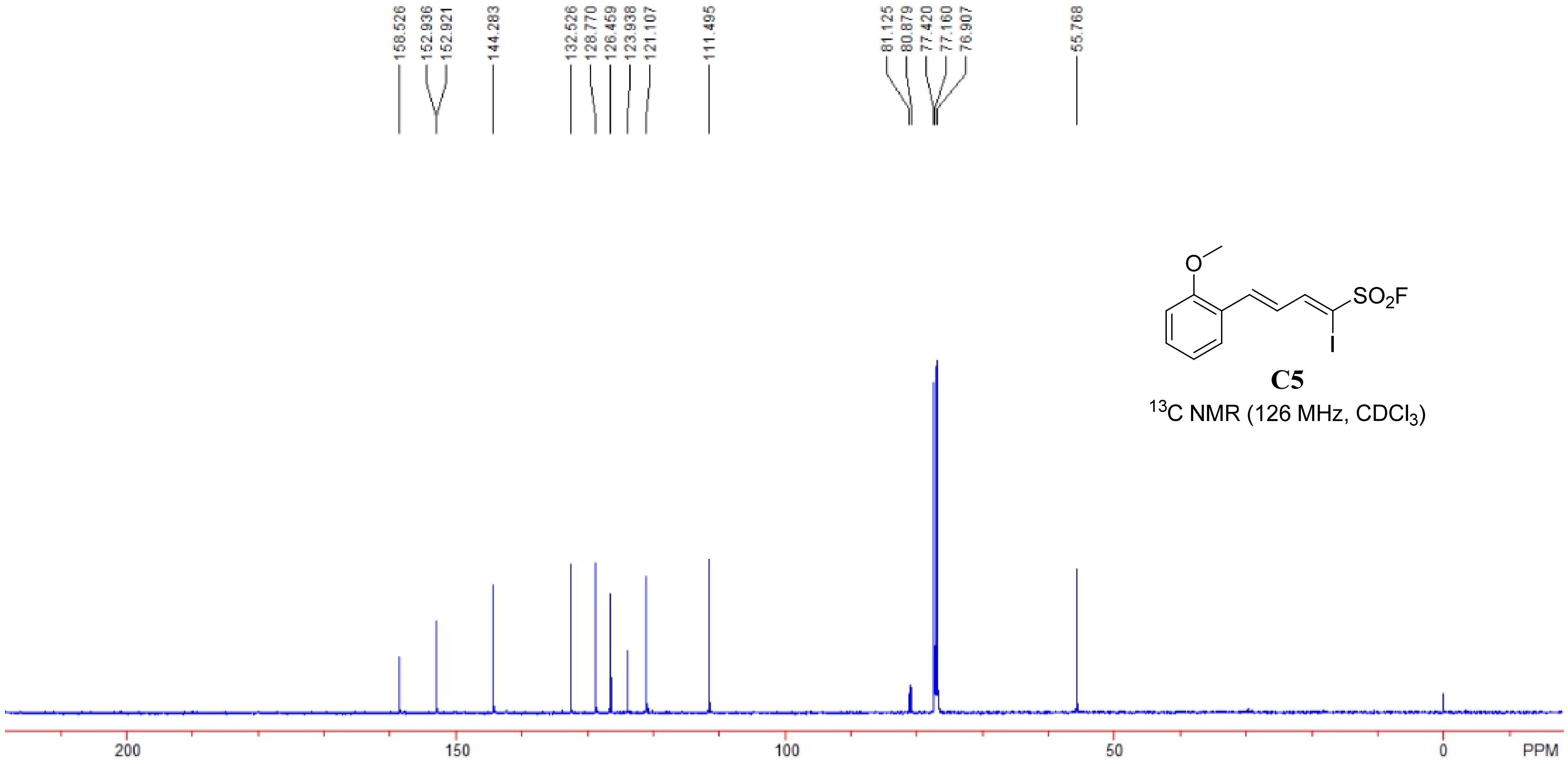

C5

^{13}C NMR (126 MHz, $CDCl_3$)

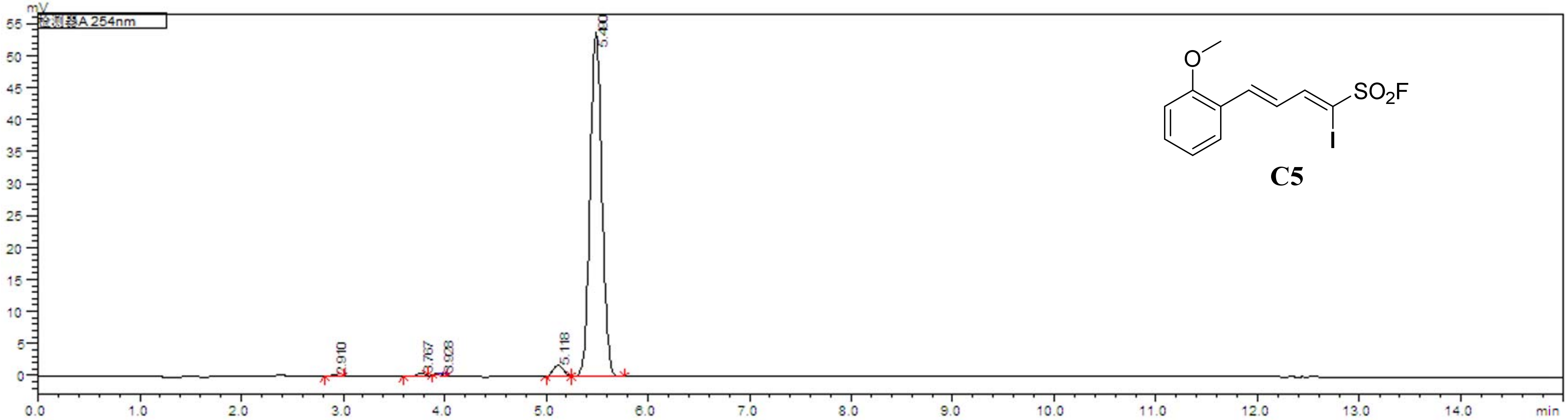

No.	Ret Time (min)	Area (mAU*min)	Rel.Area (%)
1	2.910	1718	0.38%
2	3.767	1871	0.41%
3	3.928	1332	0.29%
4	5.118	11829	2.61%
5	5.490	437026	96.31%
Total		453776	

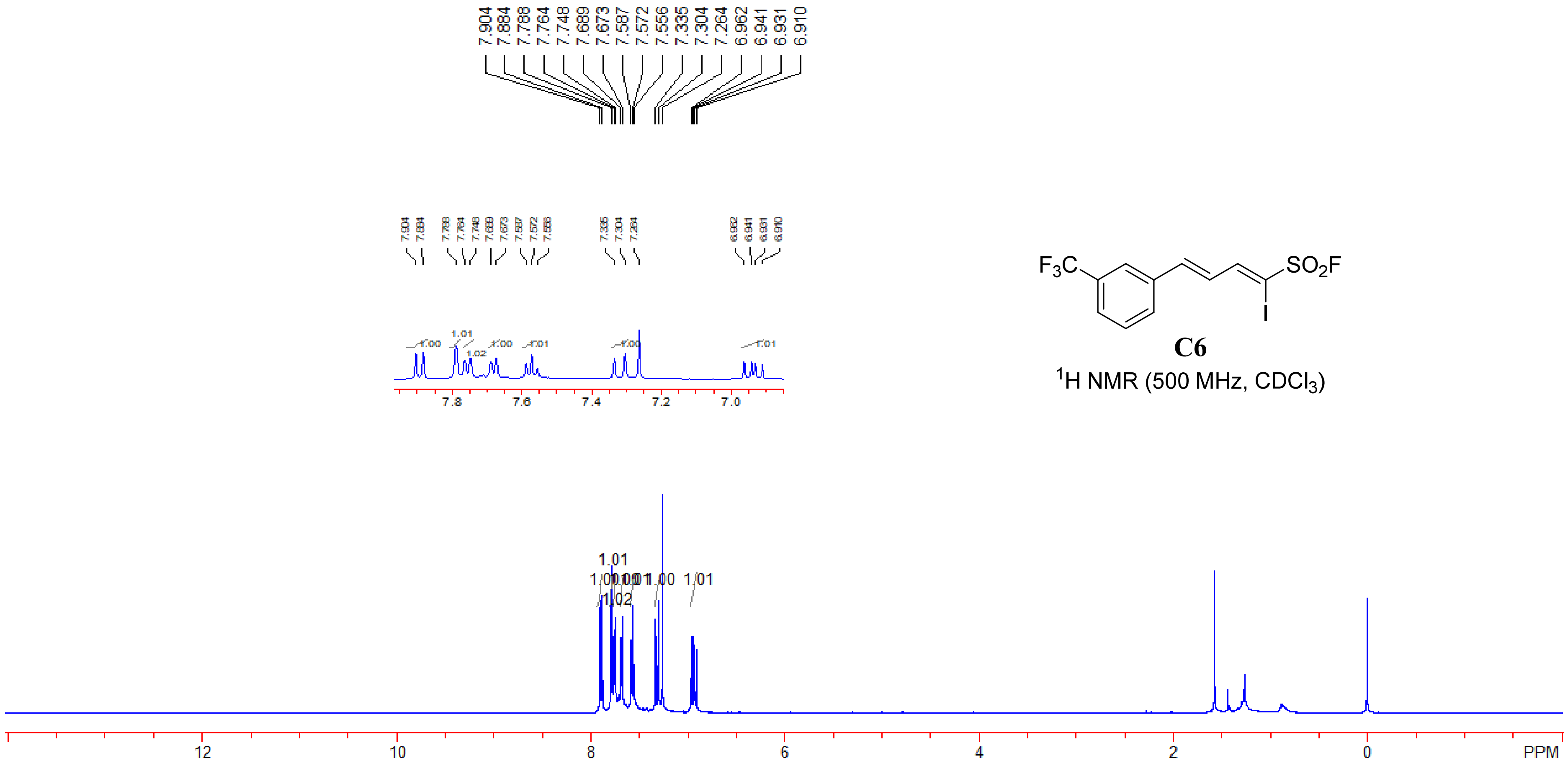

C6

^{1}H NMR (500 MHz, $CDCl_3$)

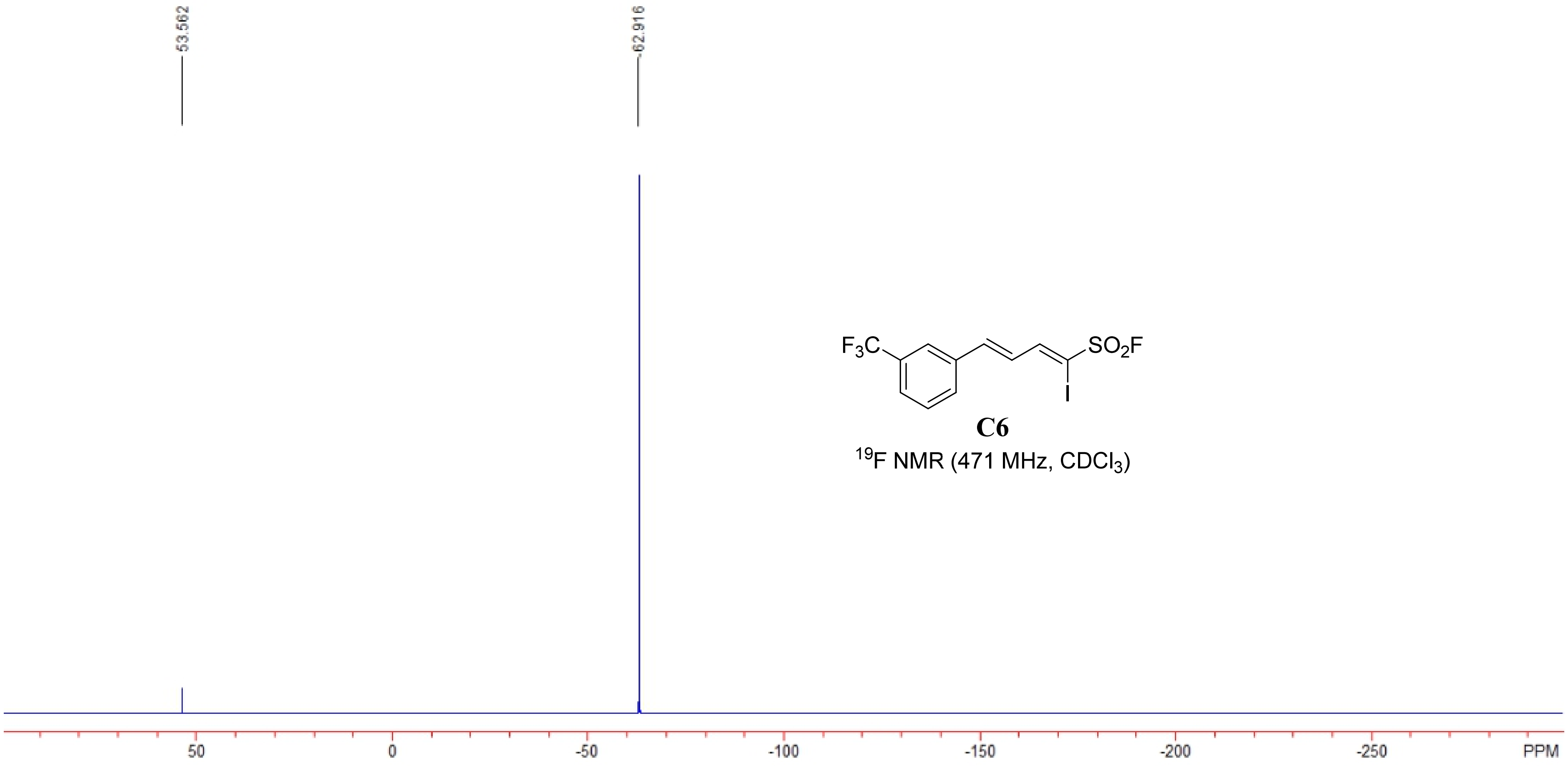

C6

^{19}F NMR (471 MHz, $CDCl_3$)

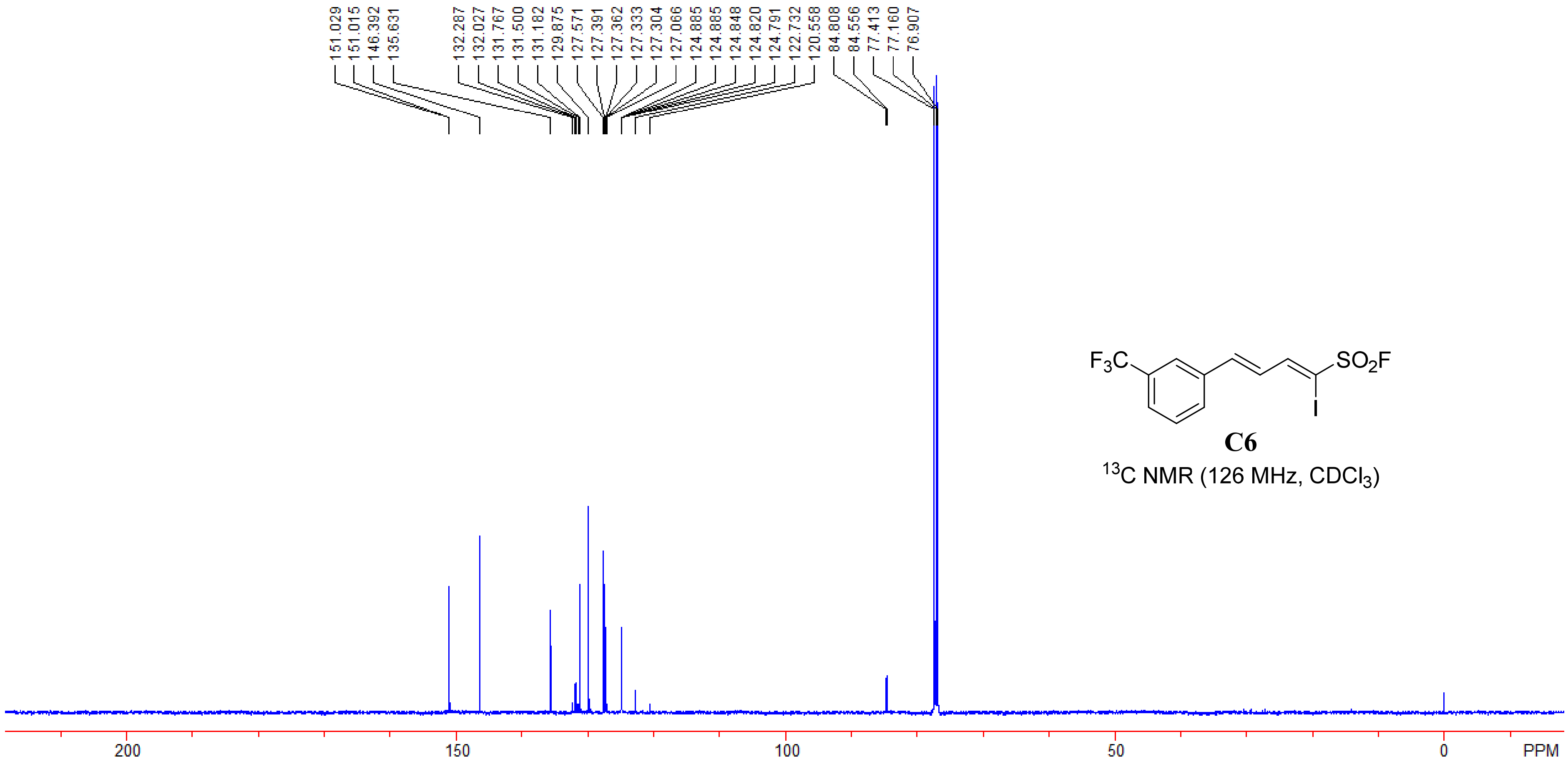

C6

^{13}C NMR (126 MHz, $CDCl_3$)

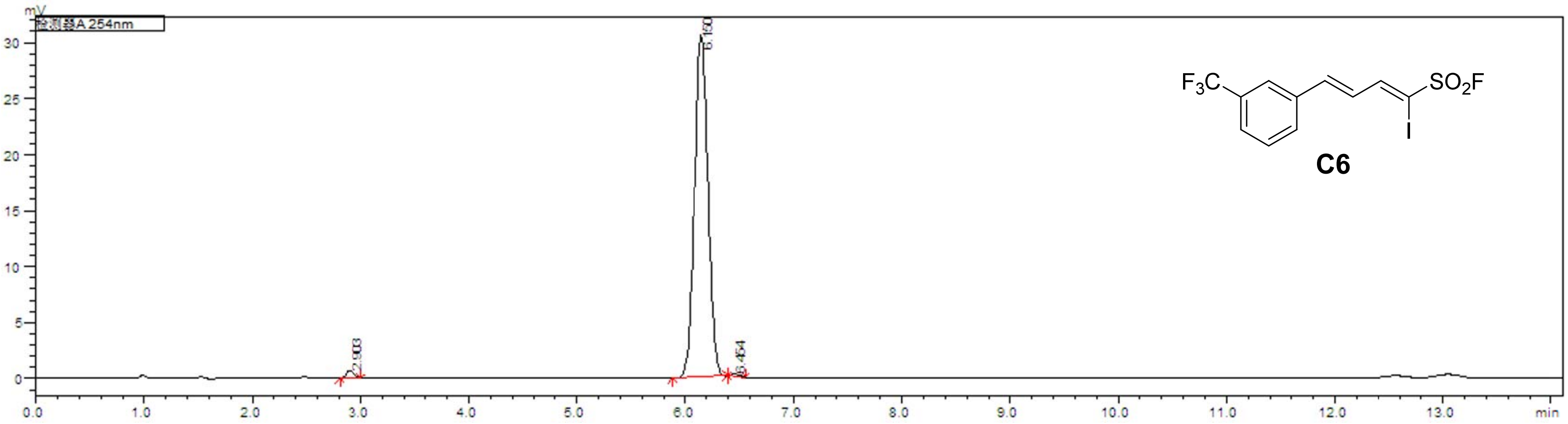

No.	Ret Time (min)	Area (mAU*min)	Rel.Area (%)
1	2.903	3436	1.26%
2	6.150	268654	98.38%
3	6.454	984	0.36%
Total		273074	

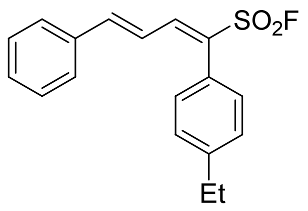

D1

^{1}H NMR (500 MHz, $CDCl_3$)

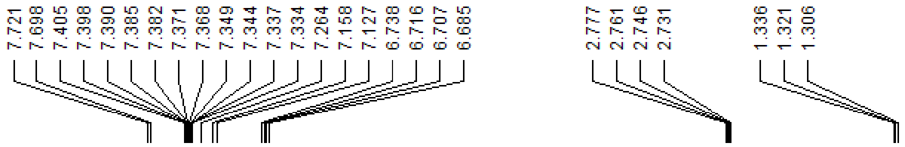

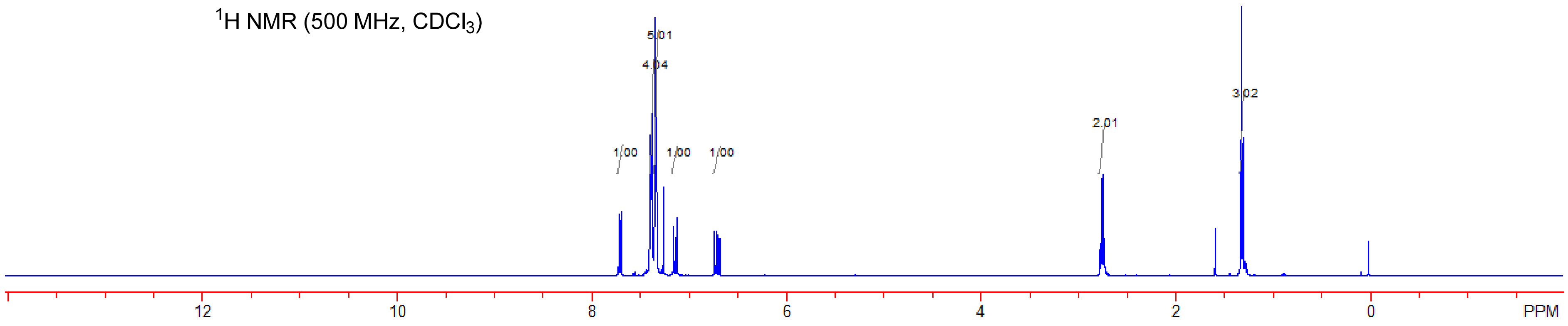

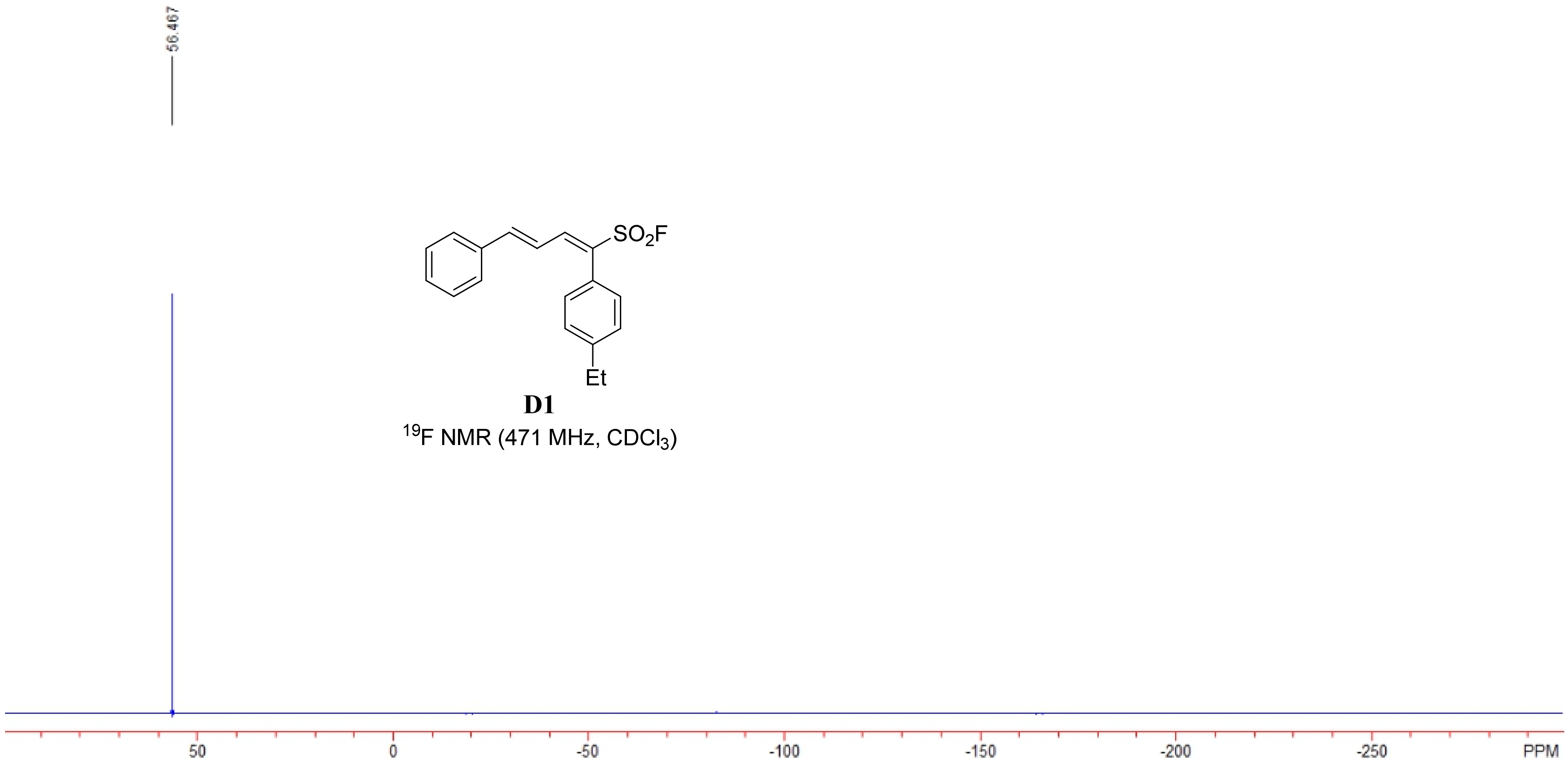
56.467
SO2F
Et
D1
19F NMR (471 MHz, CDCl3)
50
0
-50
-100
-150
-200
-250
PPM

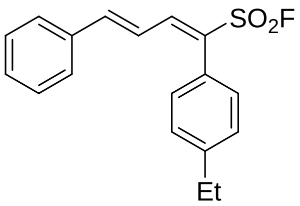

D1

^{13}C NMR (126 MHz, $CDCl_3$)

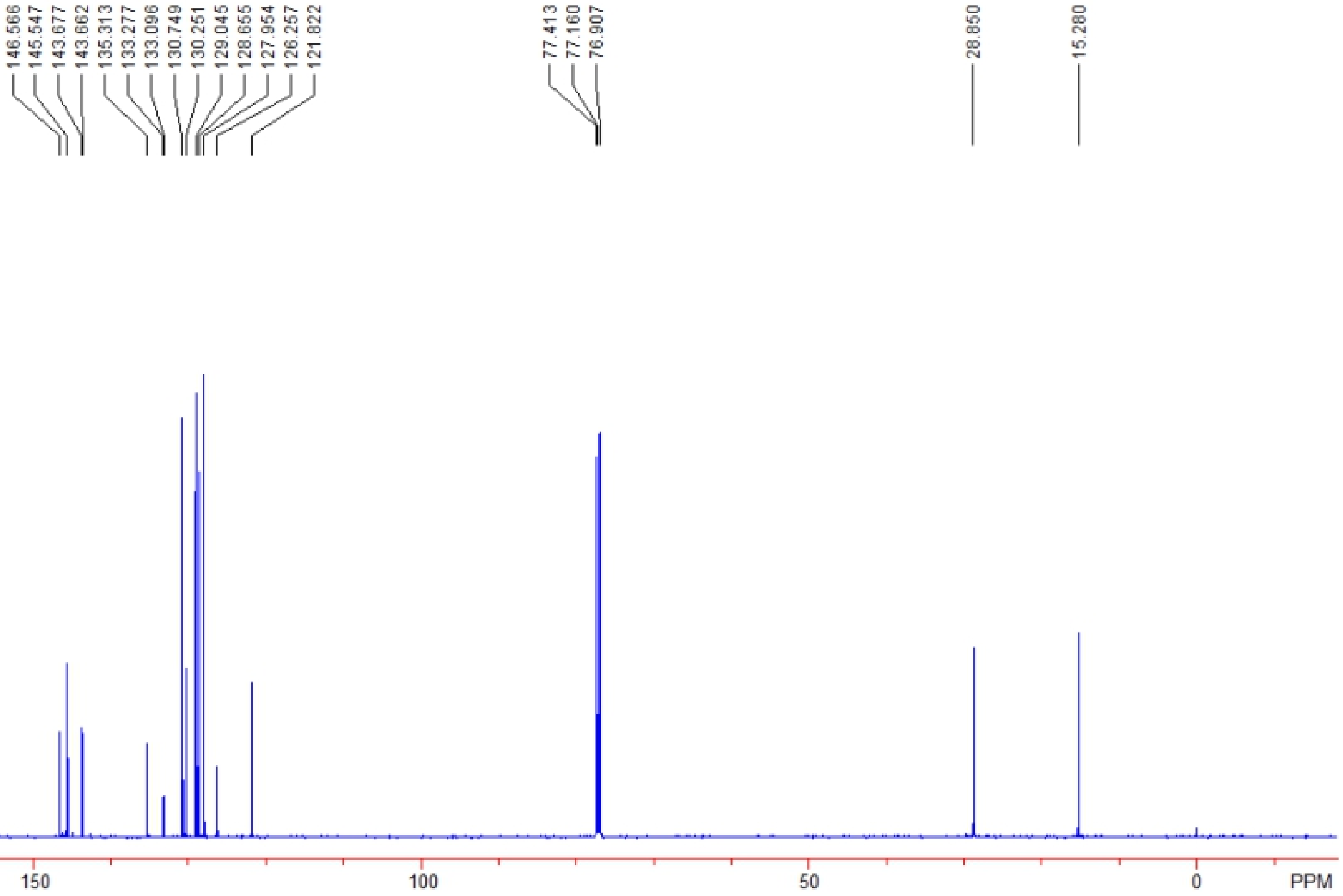

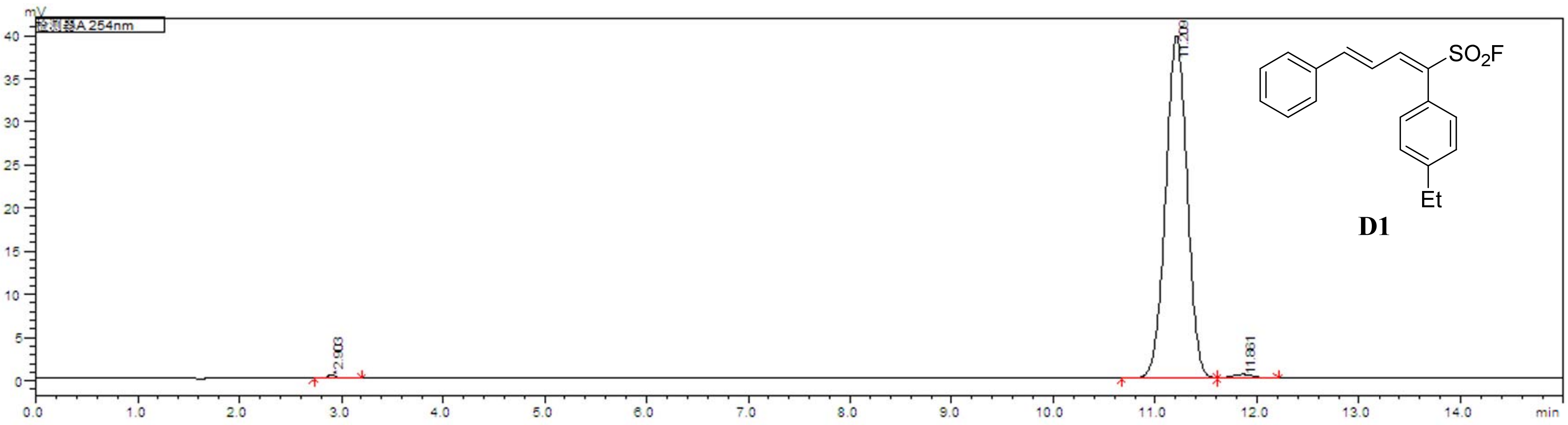

No.	Ret Time (min)	Area (mAU*min)	Rel.Area (%)
1	2.903	2744	0.46%
2	11.209	583841	98.27%
3	11.861	7555	1.27%
Total		594140	

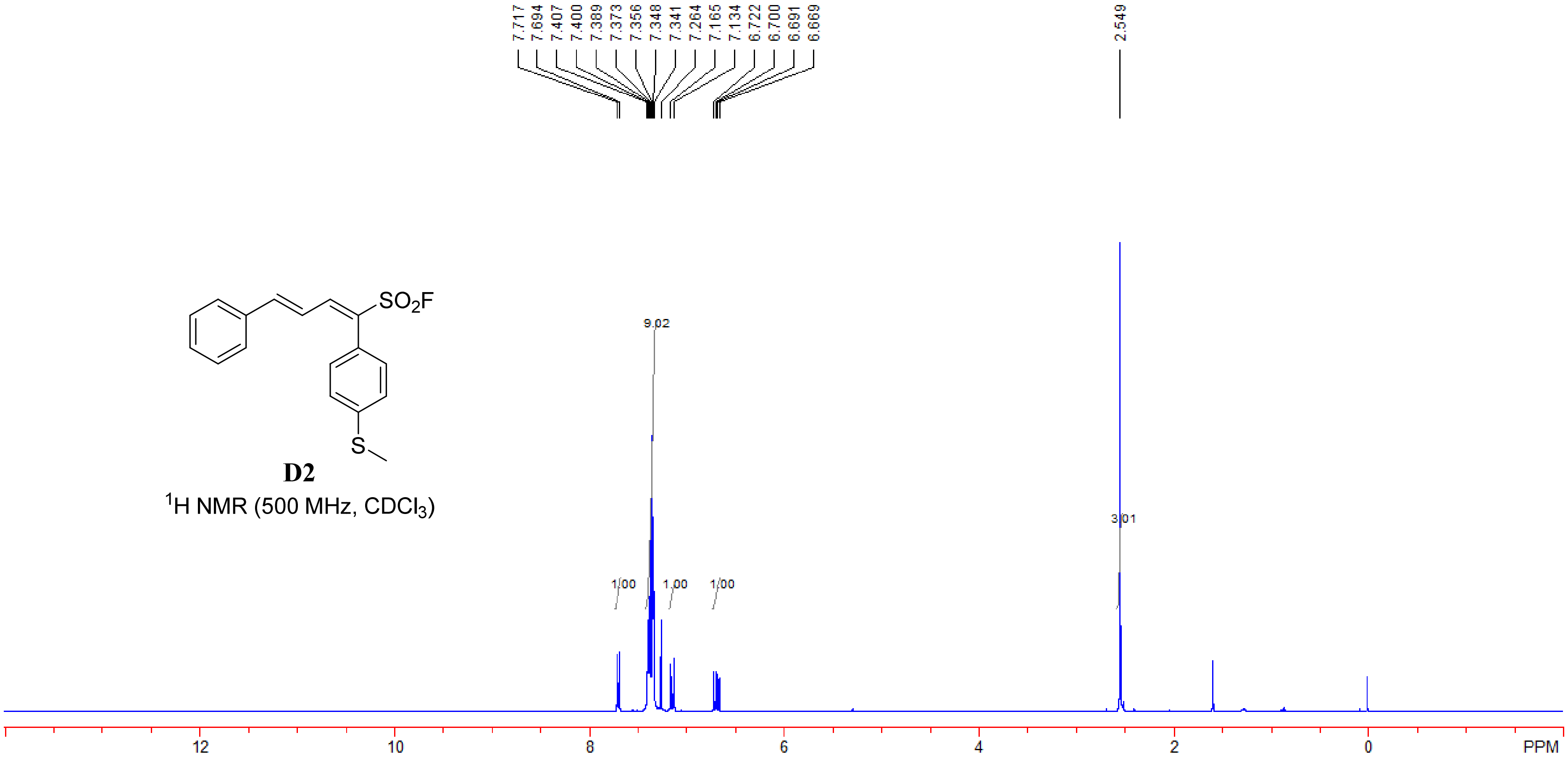

D2

^{1}H NMR (500 MHz, $CDCl_3$)

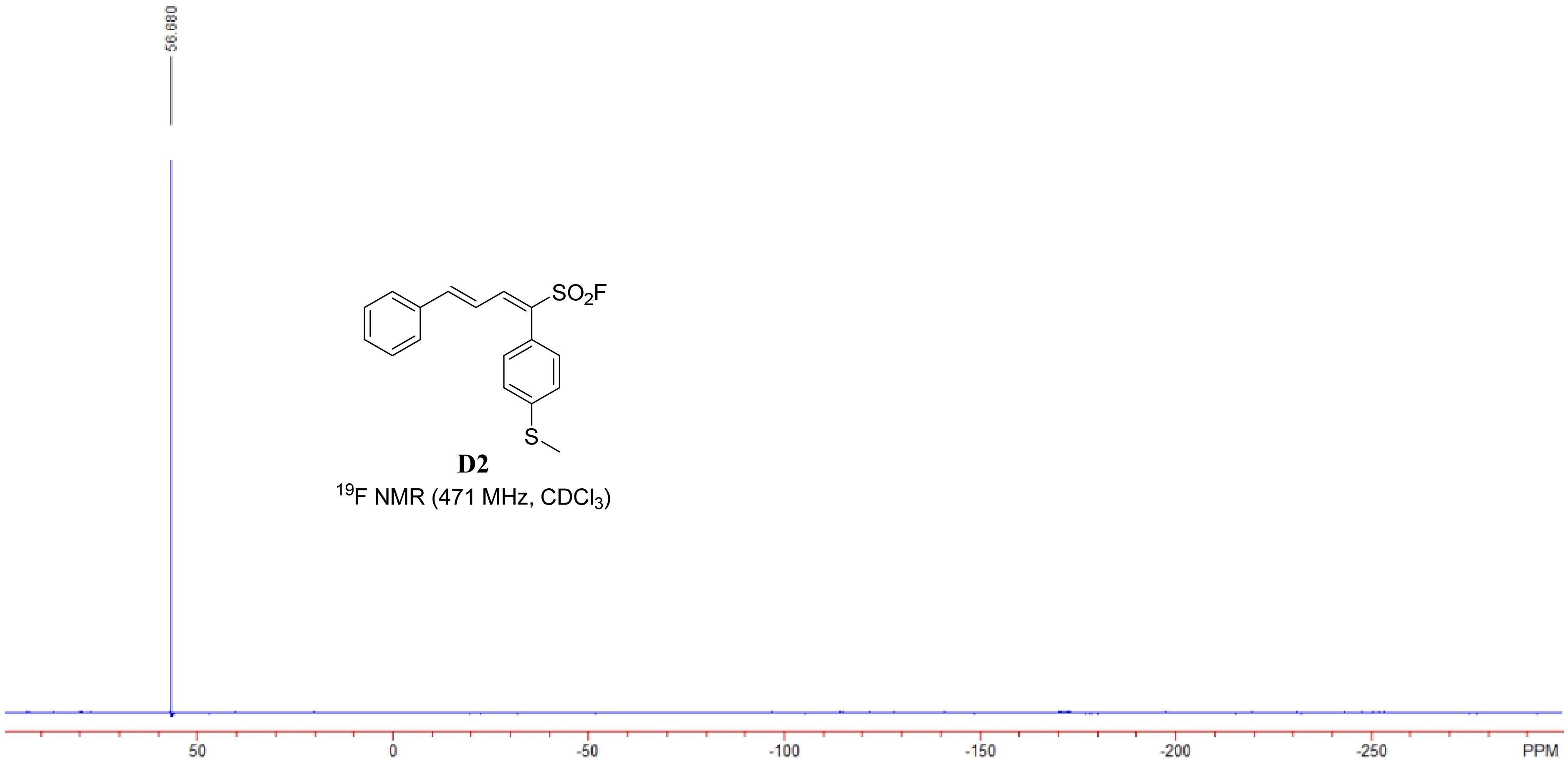

SO2F
S

D2

^{19}F NMR (471 MHz, $CDCl_3$)

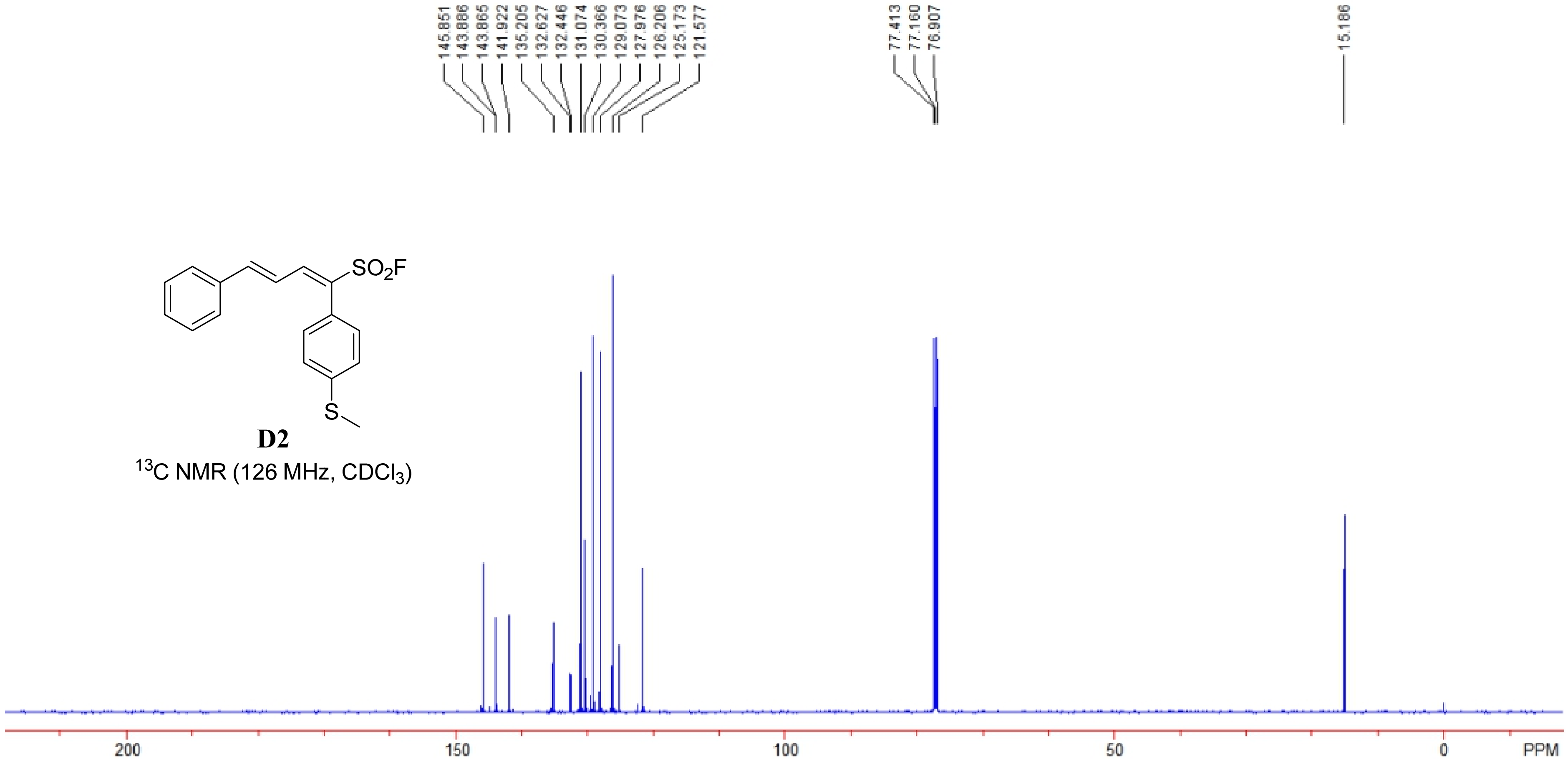
145.851
143.886
143.865
141.922
135.205
132.627
132.446
131.074
130.366
129.073
127.976
126.206
125.173
121.577
77.413
77.160
76.907
15.186
SO2F
S
D2
13C NMR (126 MHz, CDCl3)
200
150
100
50
0
PPM

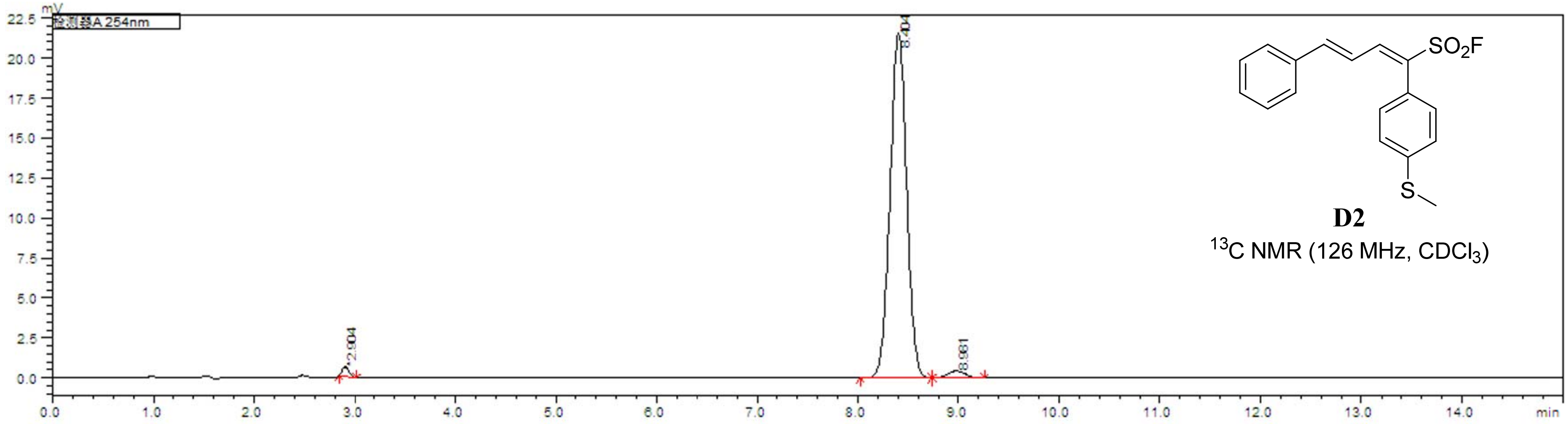

No.	Ret Time (min)	Area (mAU*min)	Rel.Area (%)
1	2.904	2832	1.10%
2	8.404	248067	96.75%
3	8.981	5489	2.14%
Total		256388	

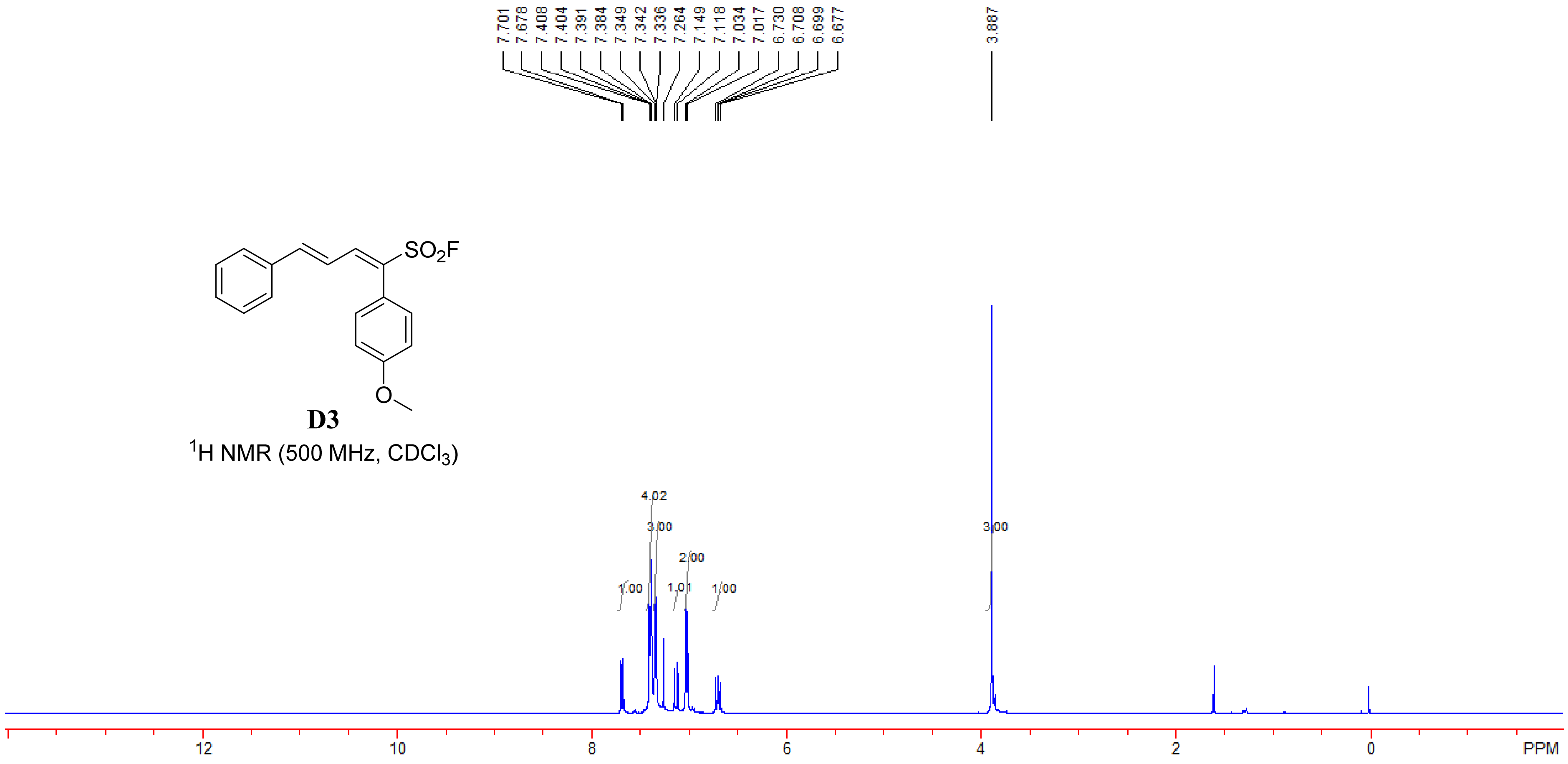

D3

^{1}H NMR (500 MHz, $CDCl_3$)

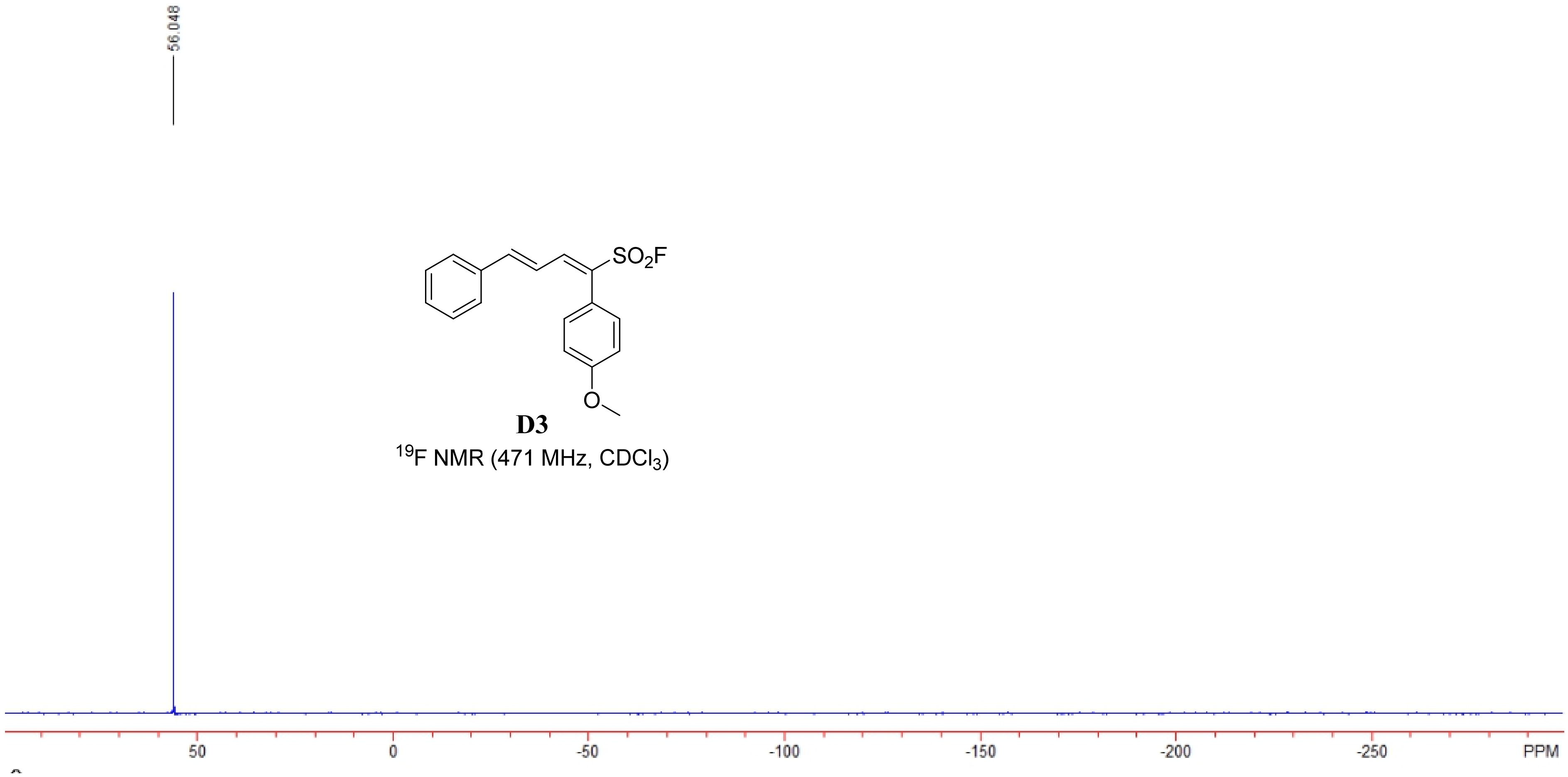

D3

^{19}F NMR (471 MHz, $CDCl_3$)

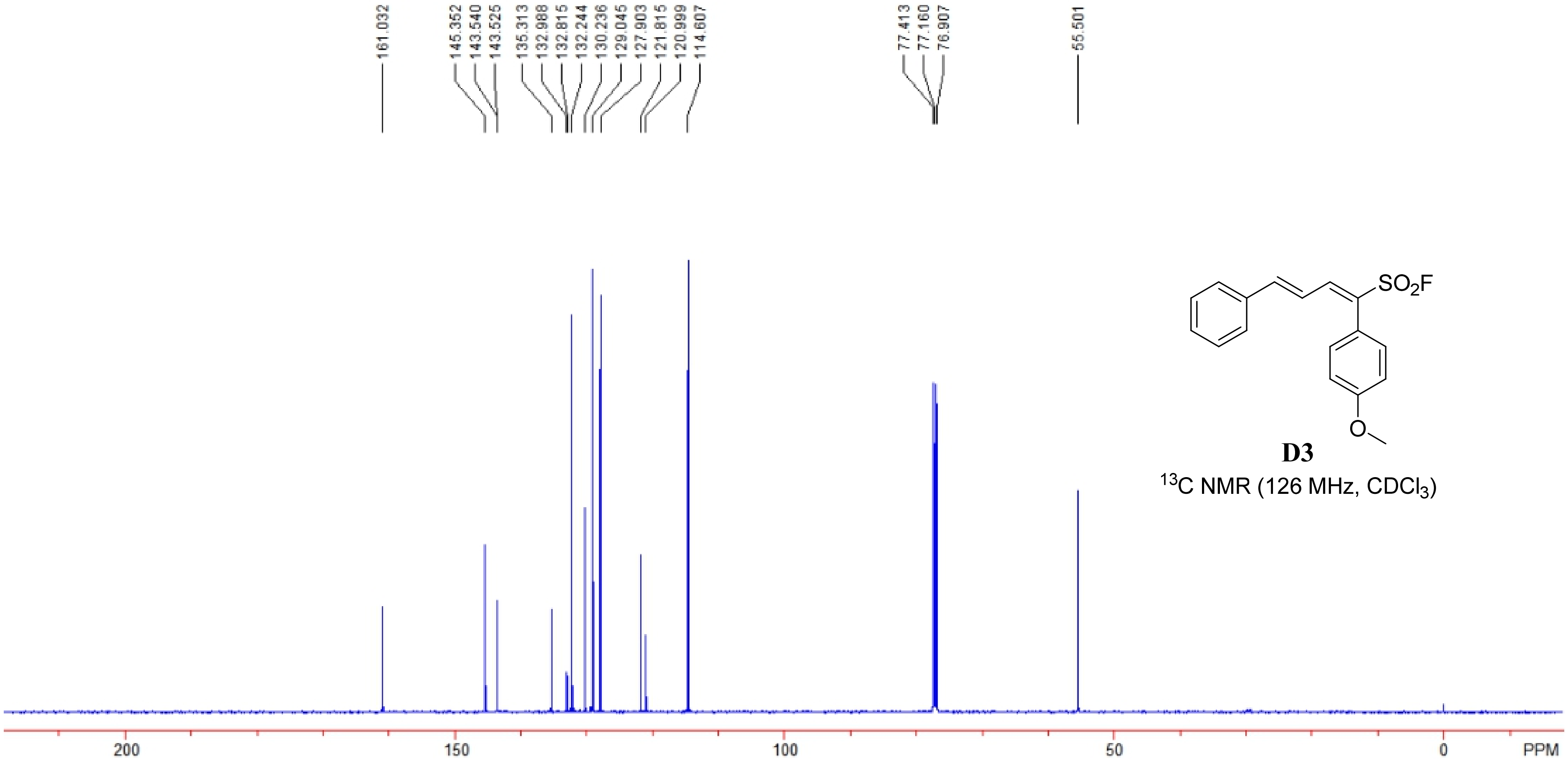

D3

^{13}C NMR (126 MHz, $CDCl_3$)

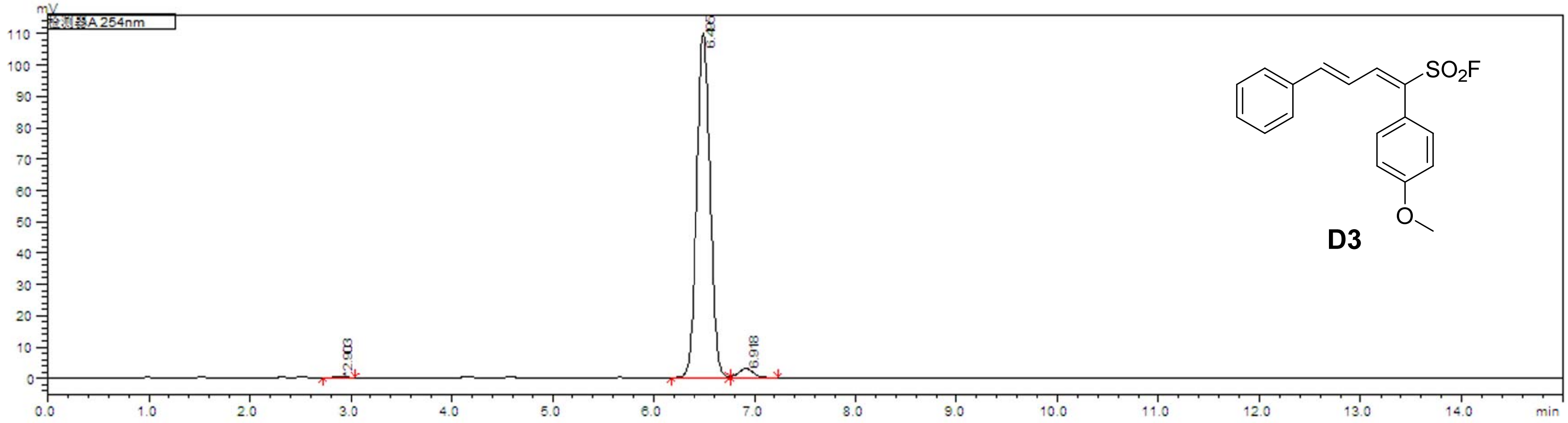

No.	Ret Time (min)	Area (mAU*min)	Rel.Area (%)
1	2.903	2608	0.25%
2	6.495	1026105	97.08%
3	6.918	28250	2.67%
Total		1056963	

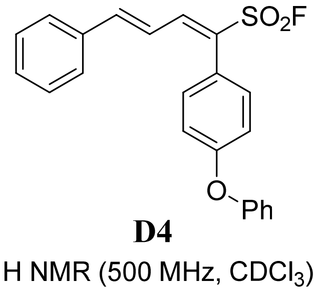

D4

^1H NMR (500 MHz, $CDCl_3$)

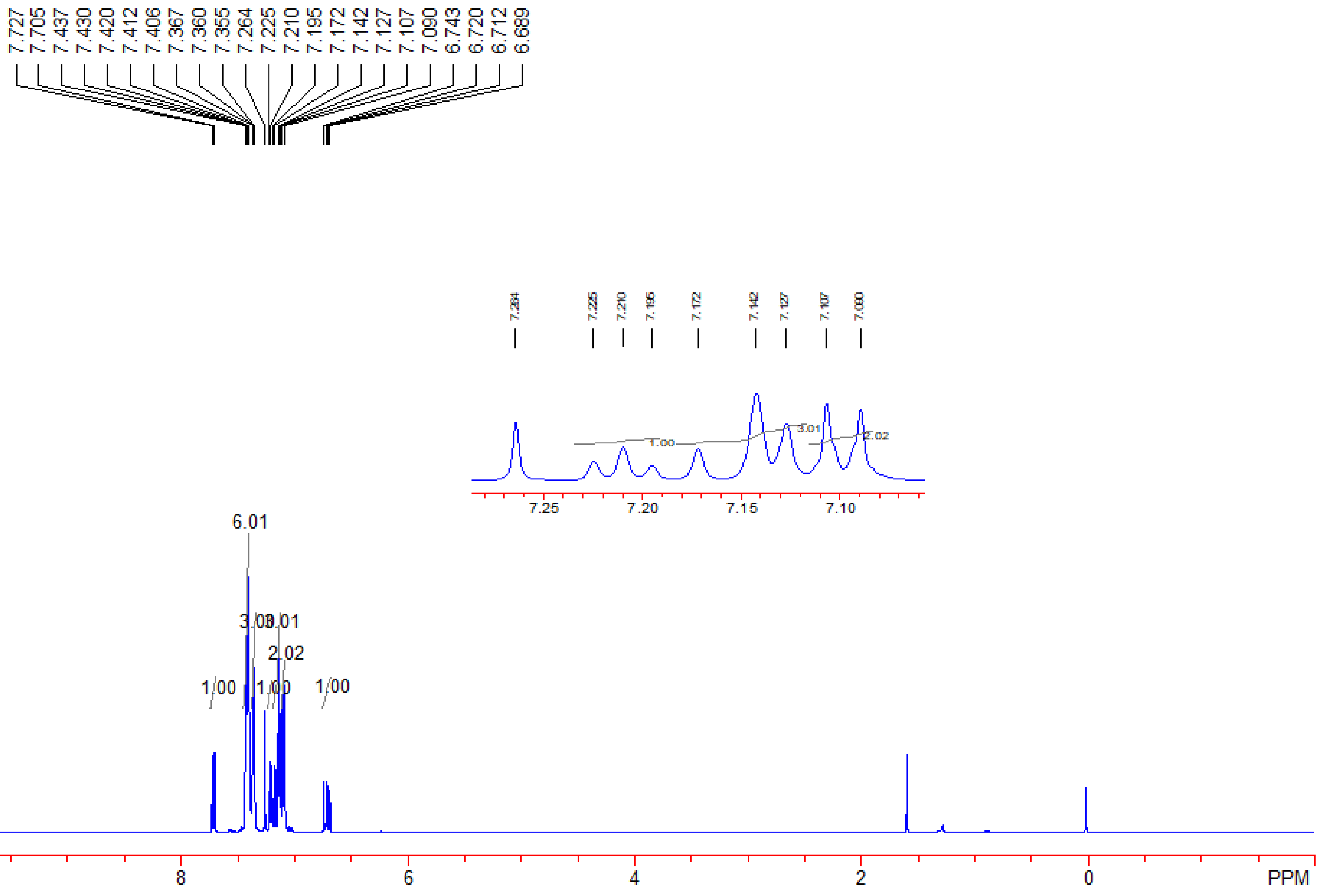

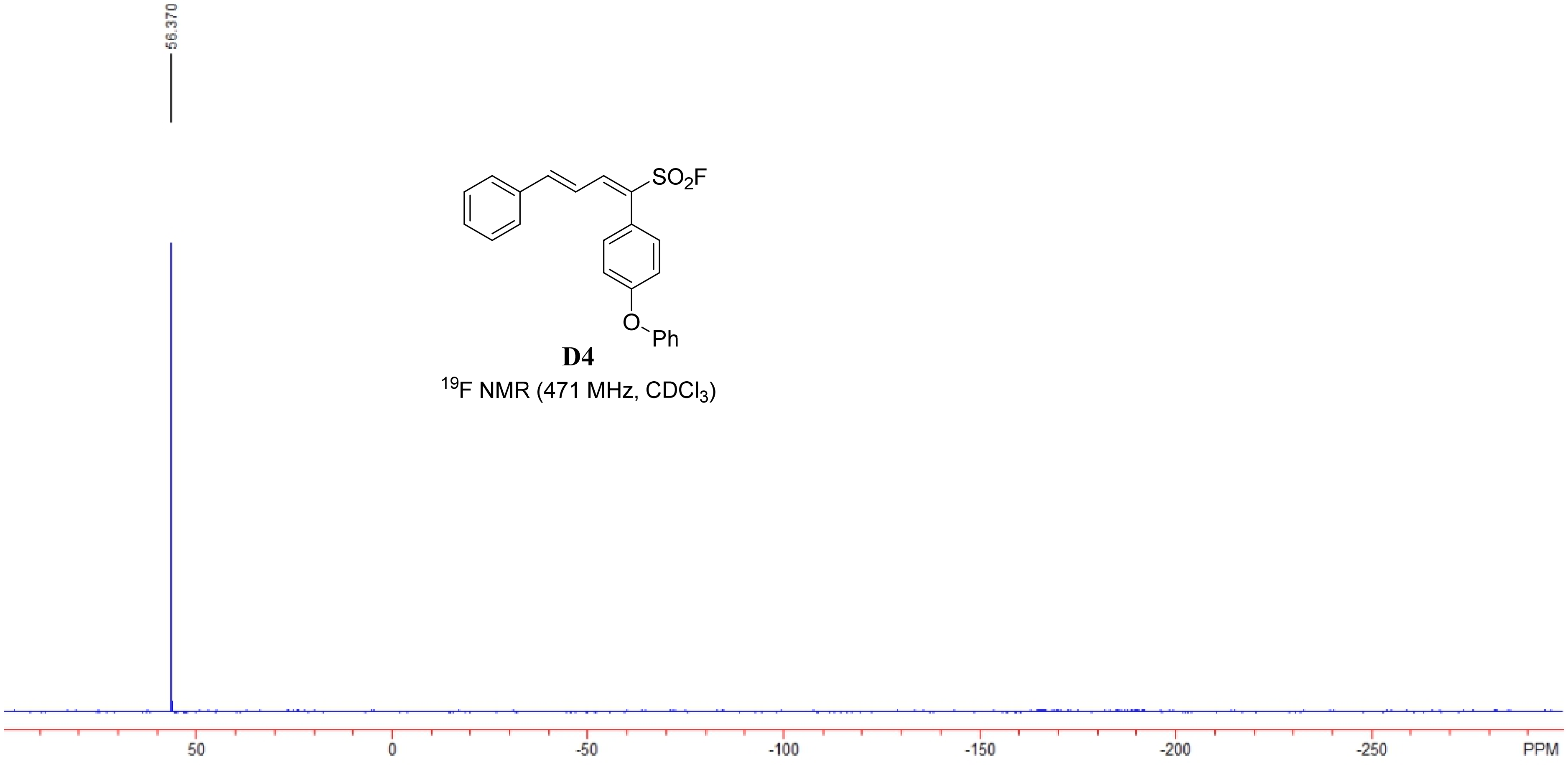

D4

^{19}F NMR (471 MHz, $CDCl_3$)

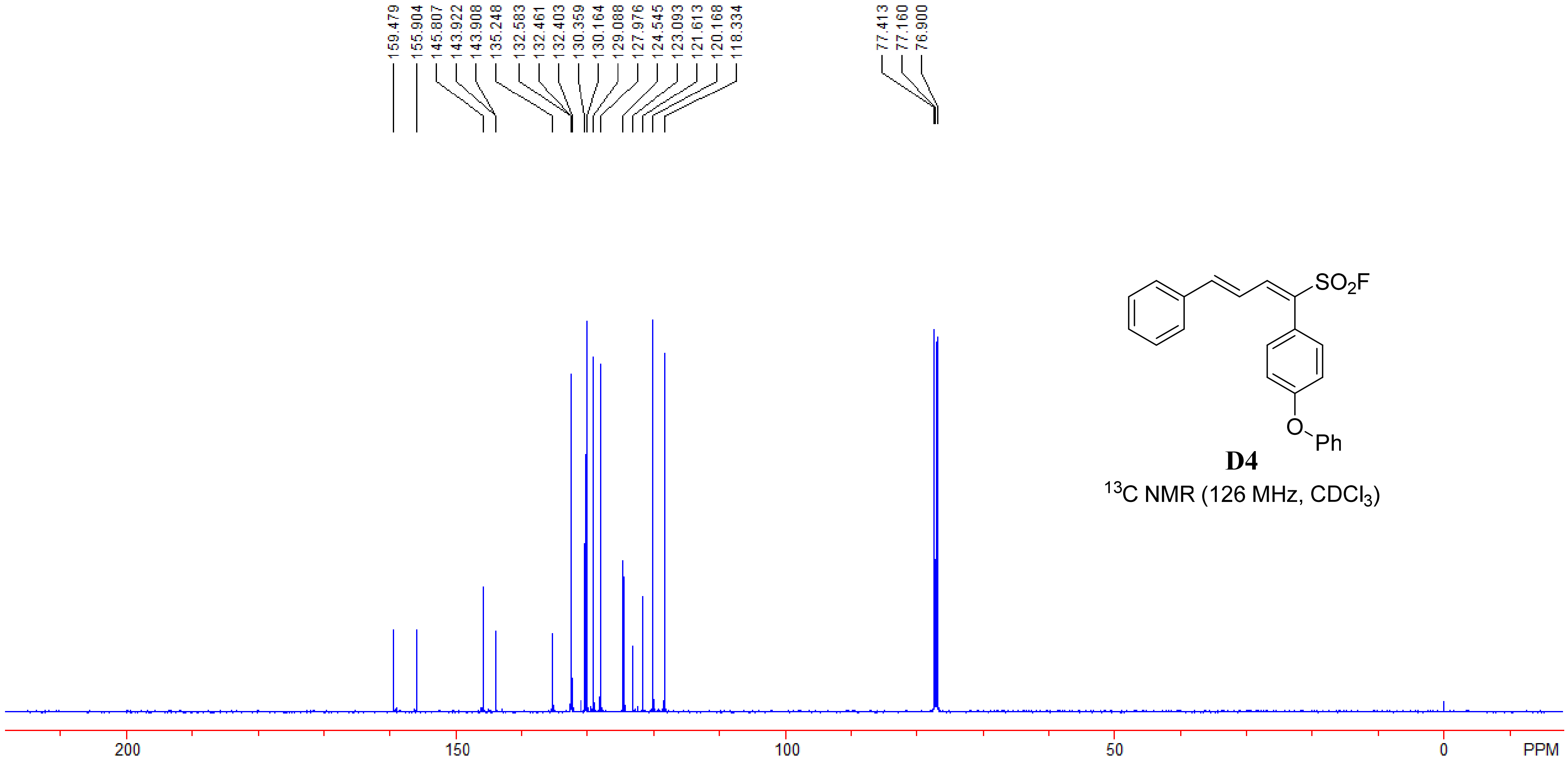

D4

^{13}C NMR (126 MHz, $CDCl_3$)

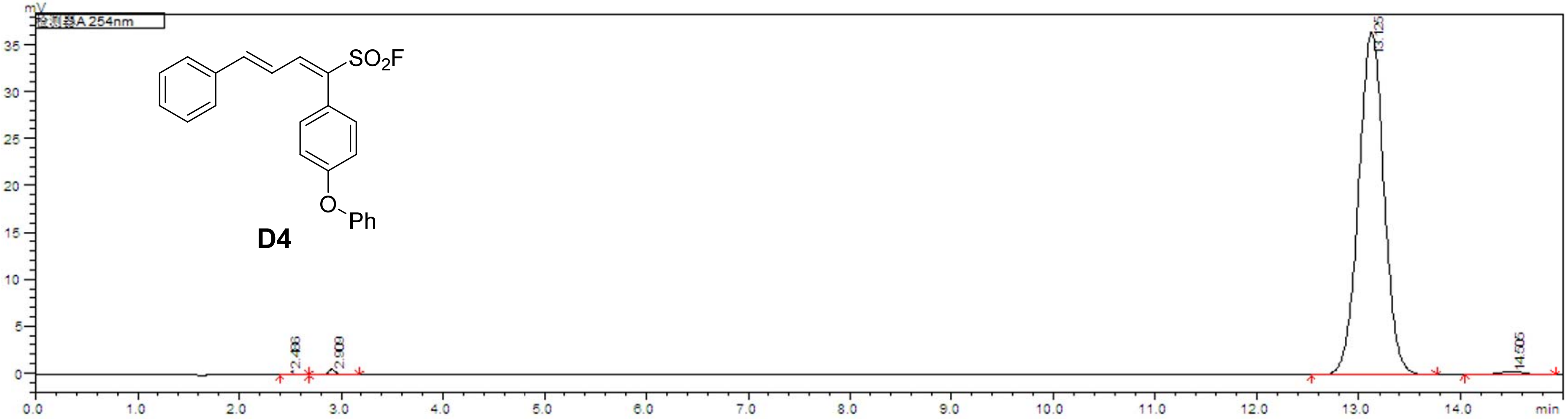

No.	Ret Time (min)	Area (mAU*min)	Rel.Area (%)
1	2.486	1044	0.16%
2	2.909	3644	0.56%
3	13.125	638262	98.17%
4	14.505	7219	1.11%
Total		650169	

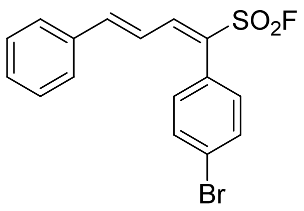

D5

^1H NMR (500 MHz, $CDCl_3$)

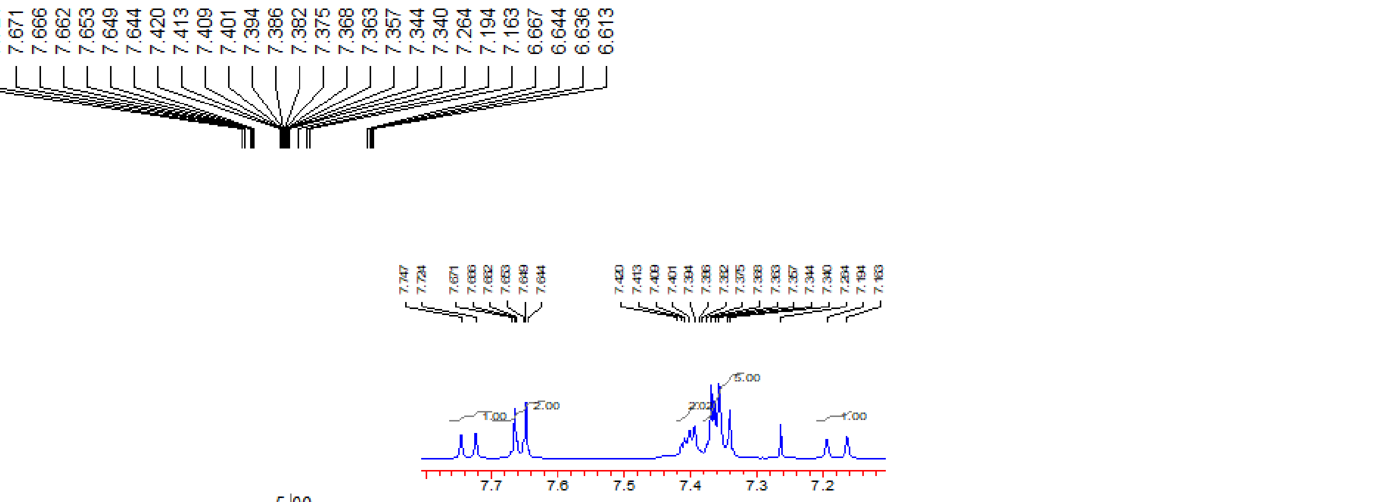

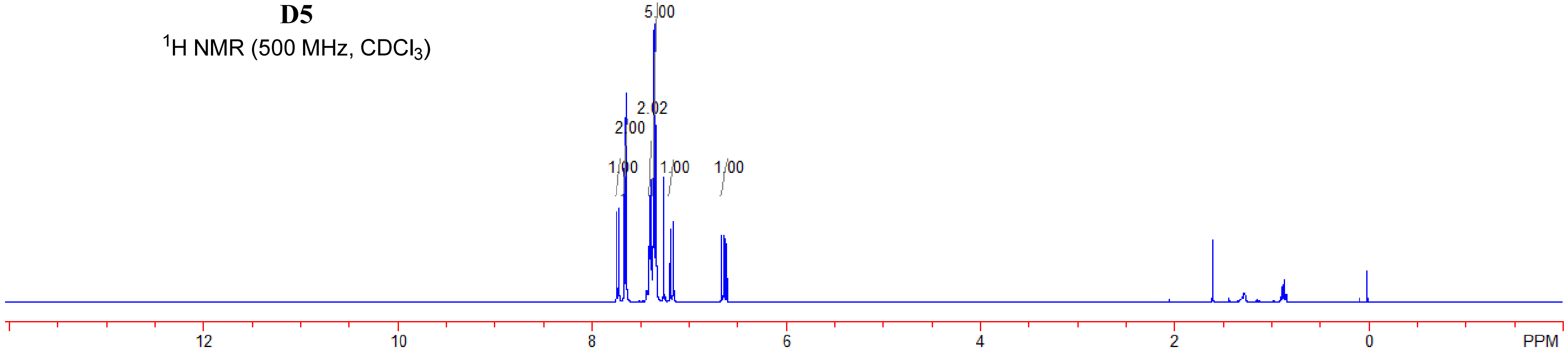

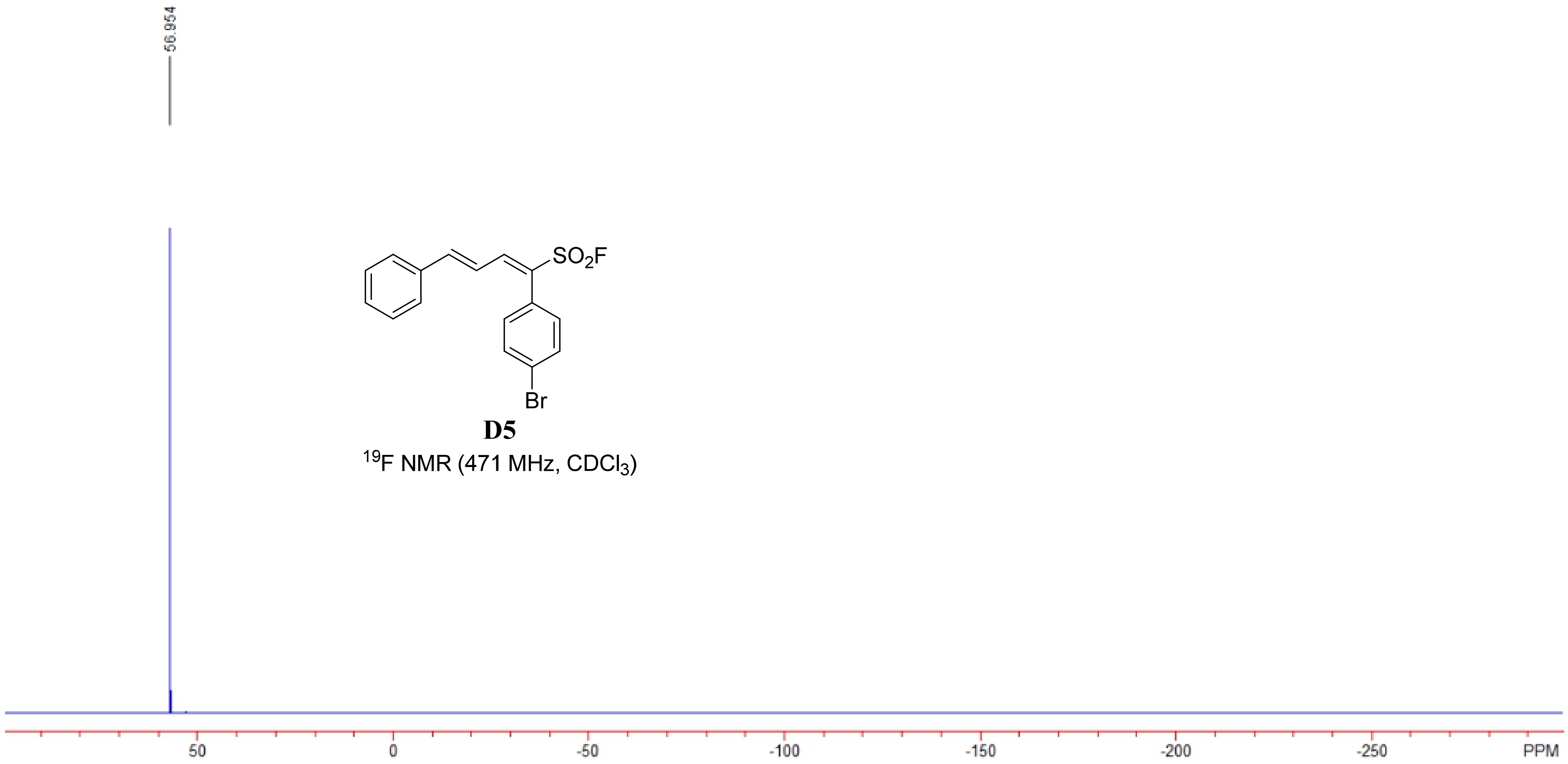
56.954
SO2F
Br
D5
19F NMR (471 MHz, CDCl3)
50
0
-50
-100
-150
-200
-250
PPM

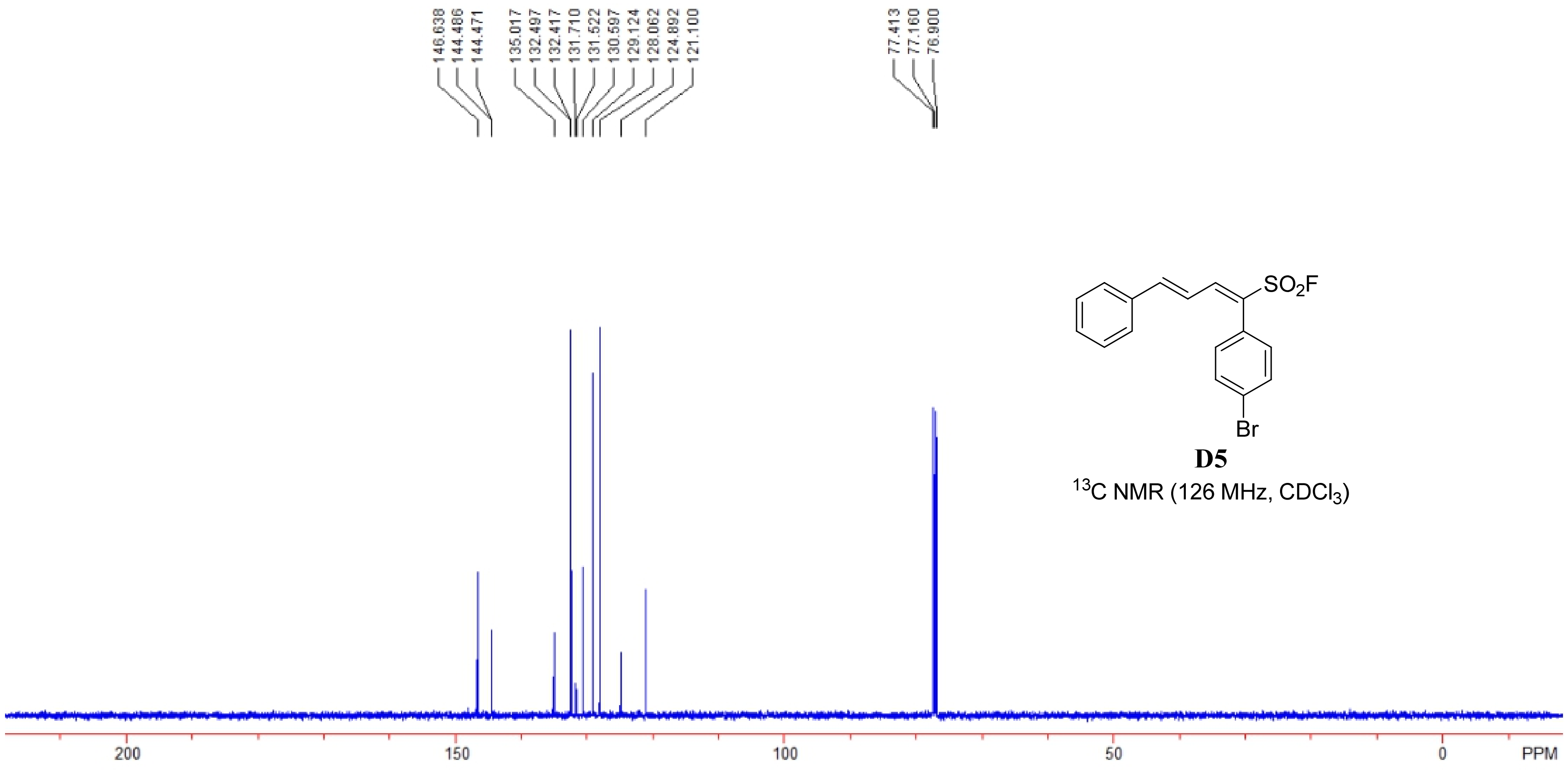

D5

^{13}C NMR (126 MHz, $CDCl_3$)

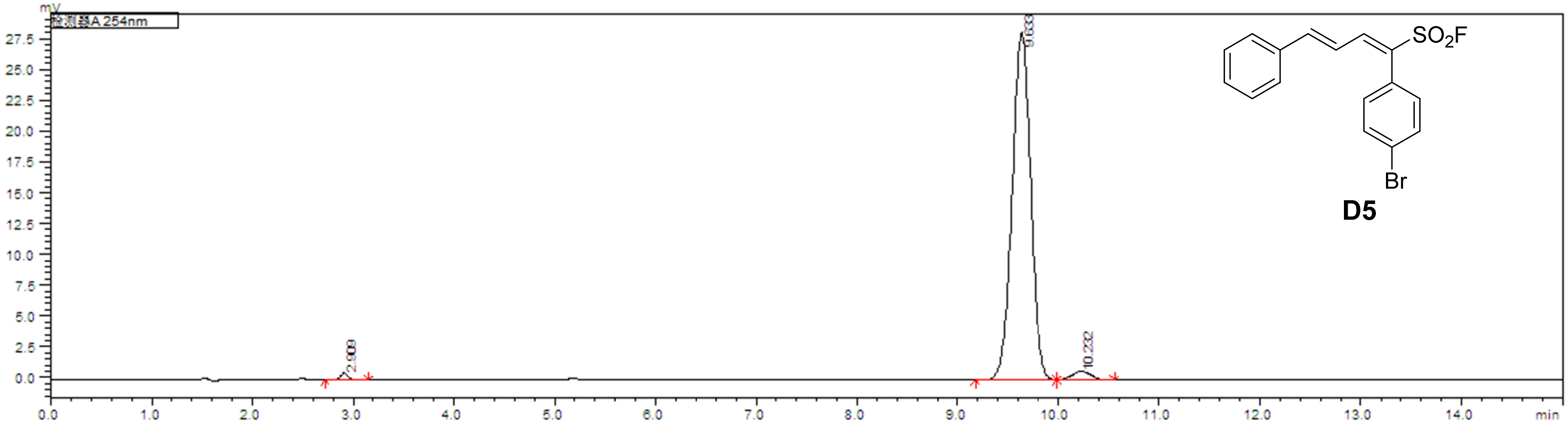

D5

No.	Ret Time (min)	Area (mAU*min)	Rel.Area (%)
1	2.909	3146	0.84%
2	9.633	363419	96.61%
3	10.232	9598	2.55%
Total		376162	

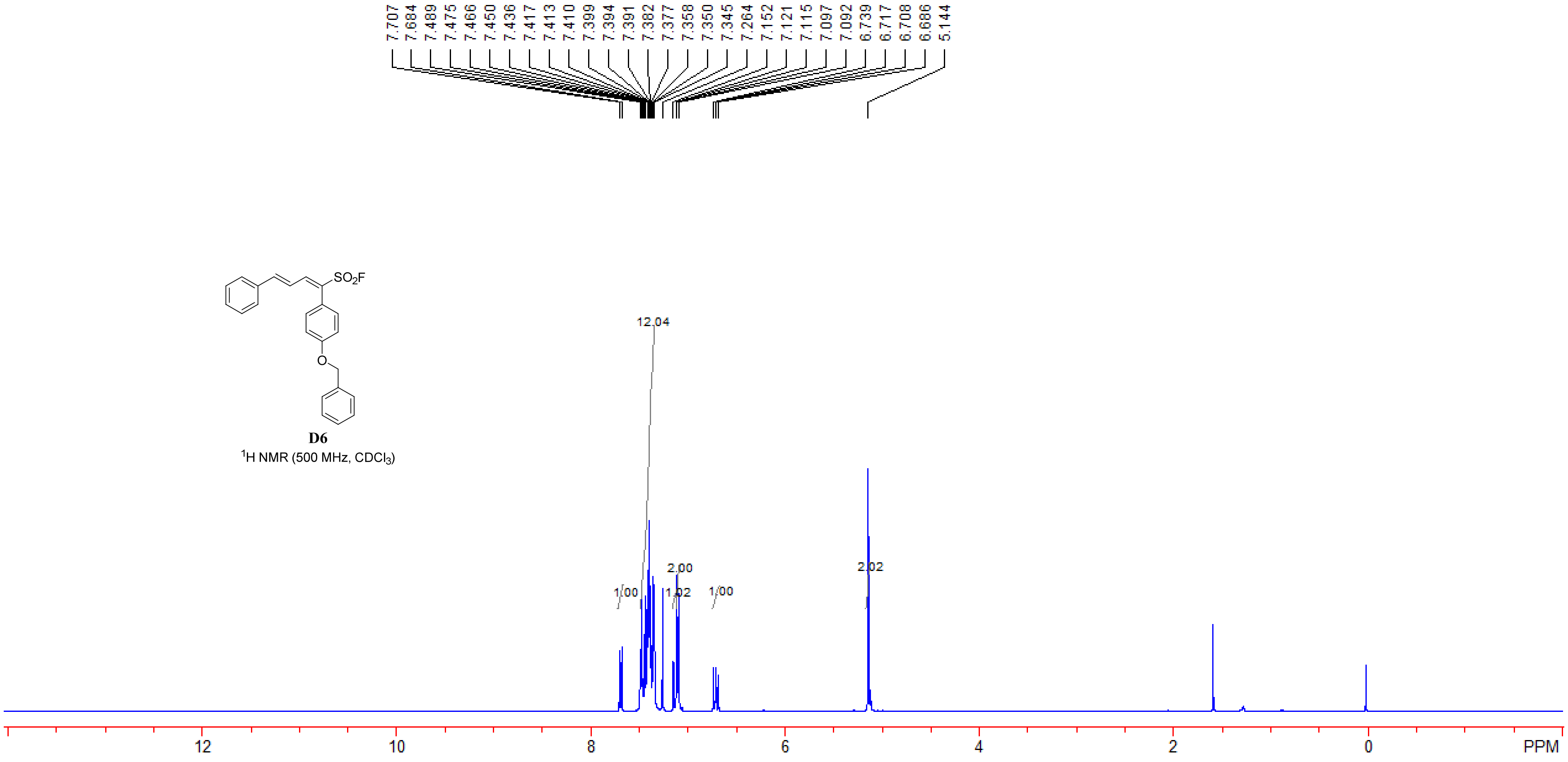

D6

^{1}H NMR (500 MHz, $CDCl_3$)

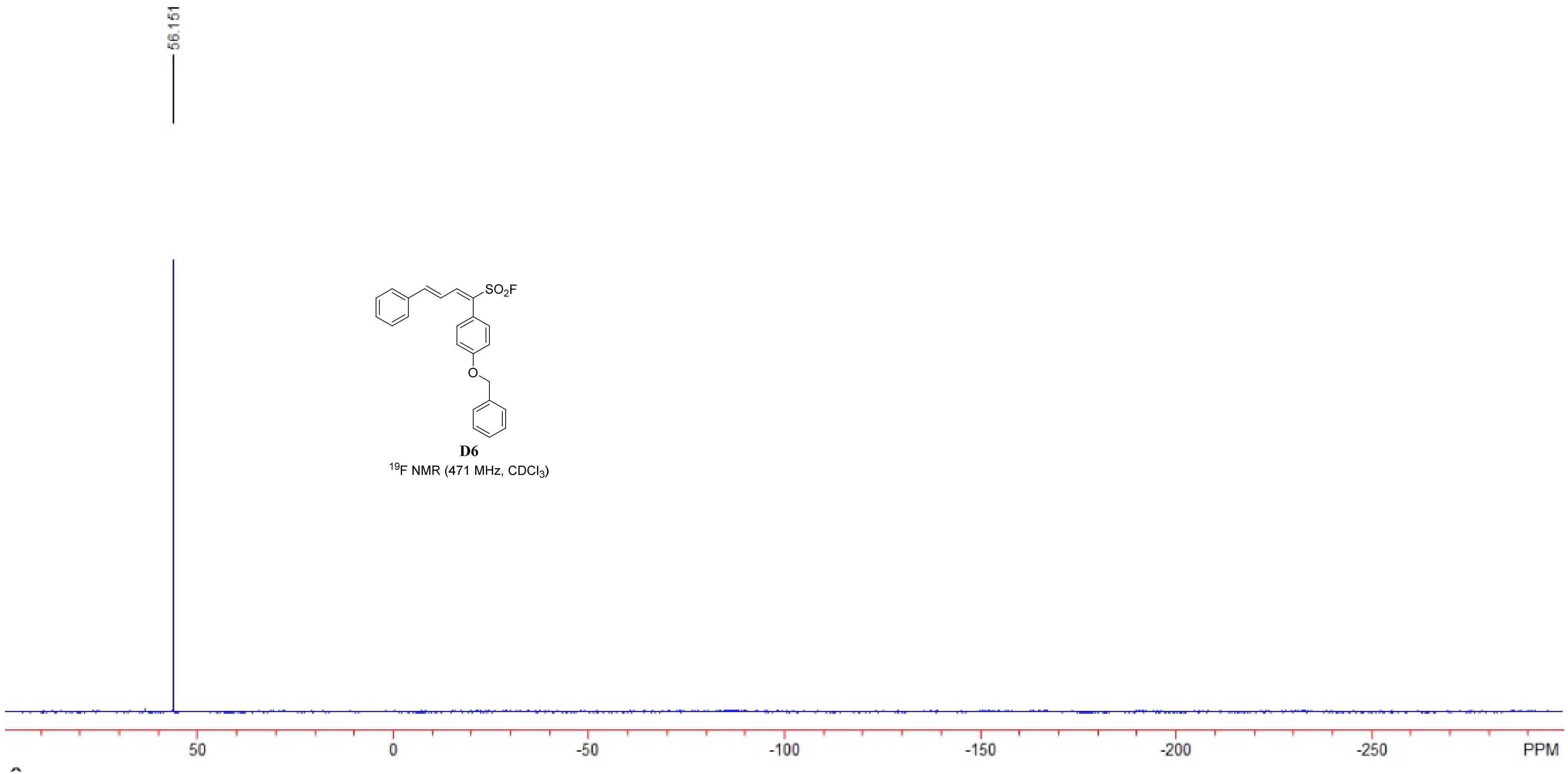
56.151
SO2F
O
D6
19F NMR (471 MHz, CDCl3)
50
0
-50
-100
-150
-200
-250
PPM

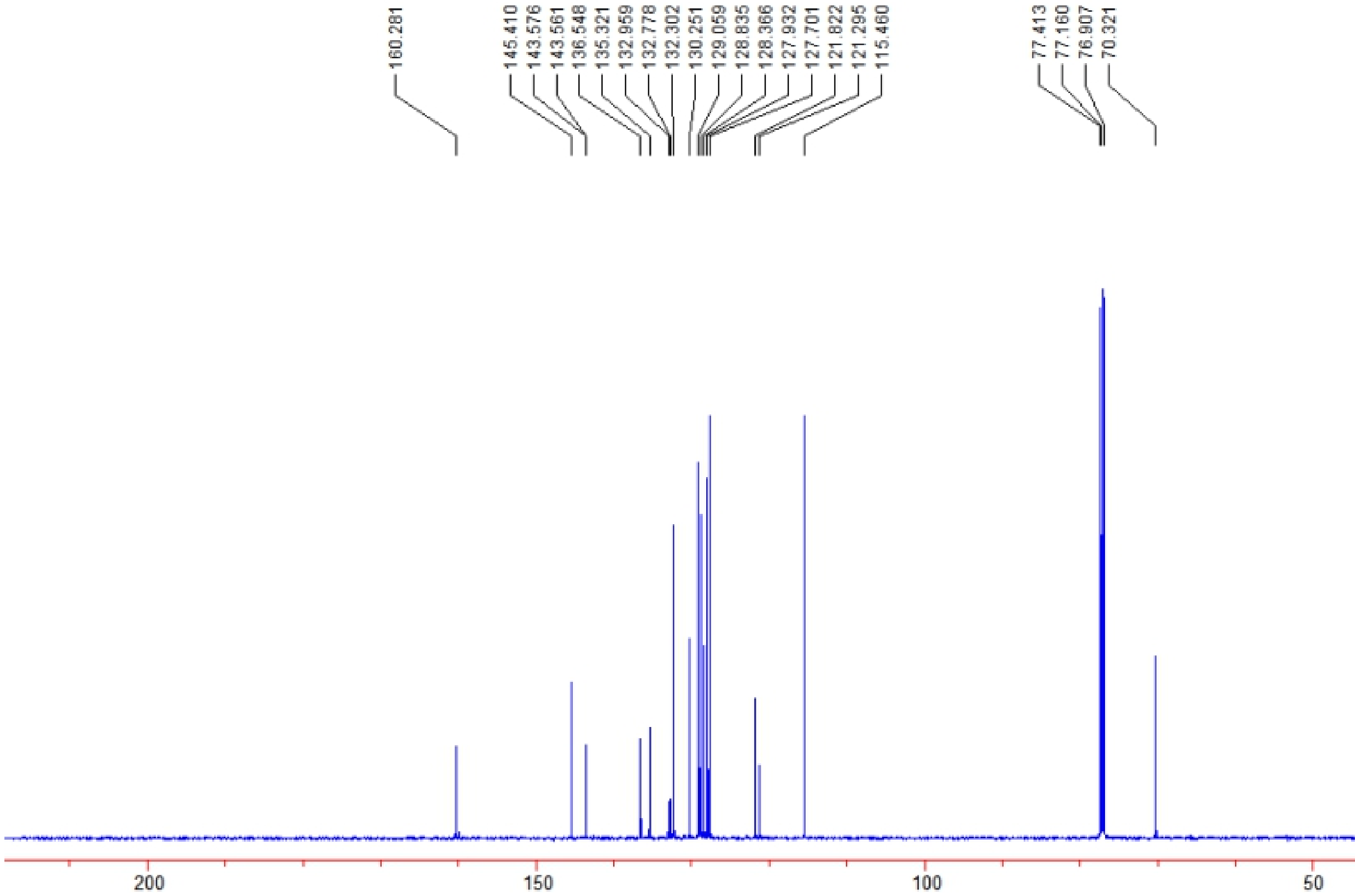

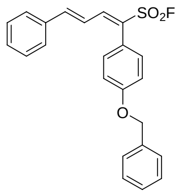

D6

^{13}C NMR (126 MHz, $CDCl_3$)

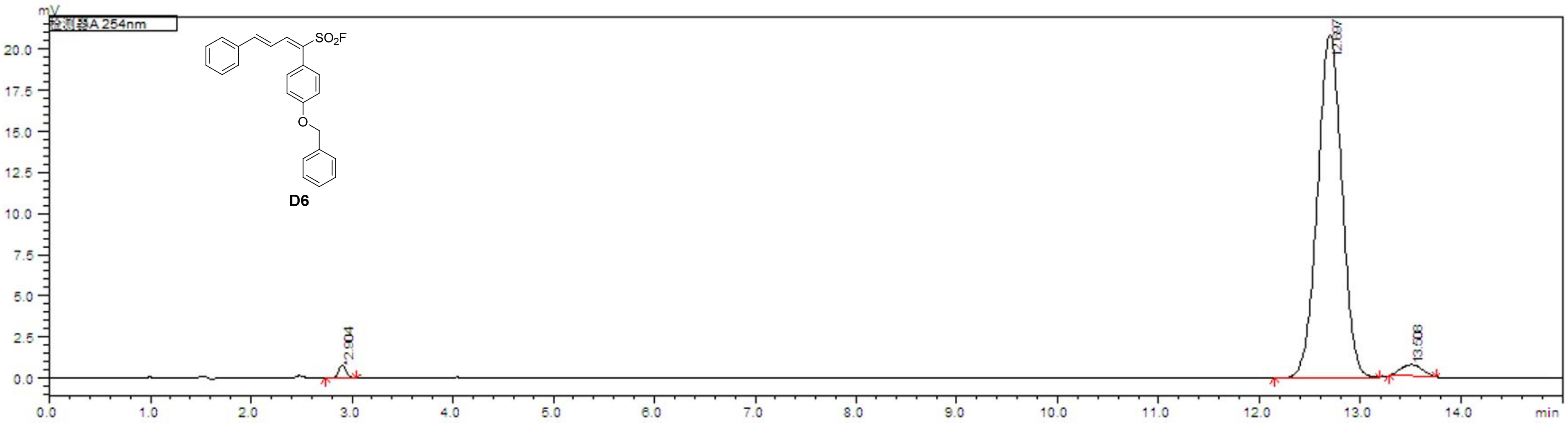

No.	Ret Time (min)	Area (mAU*min)	Rel.Area (%)
1	2.904	4320	1.17%
2	12.697	353327	95.98%
3	13.508	10482	2.85%
Total		368129	

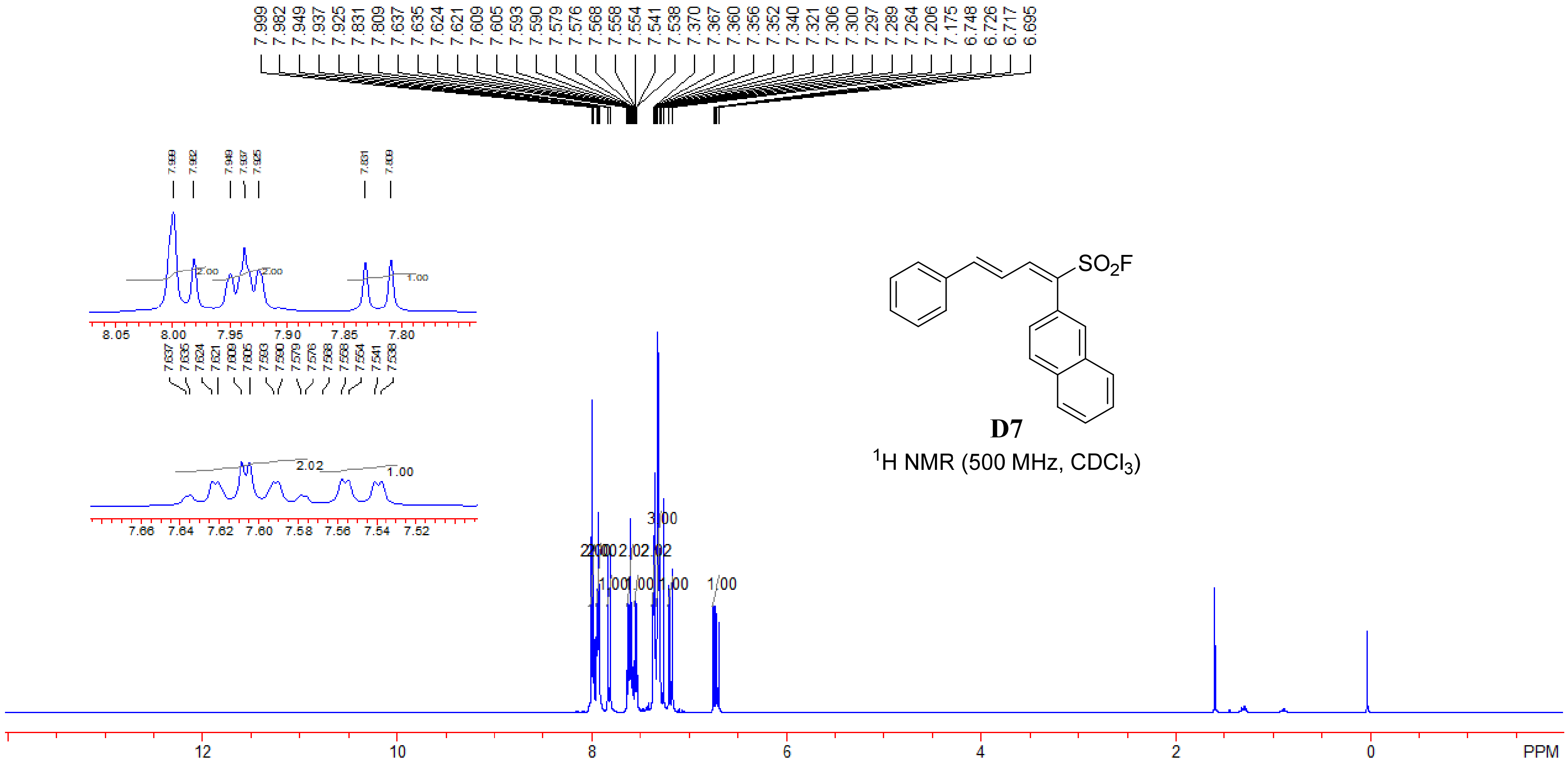

D7

^{1}H NMR (500 MHz, $CDCl_3$)

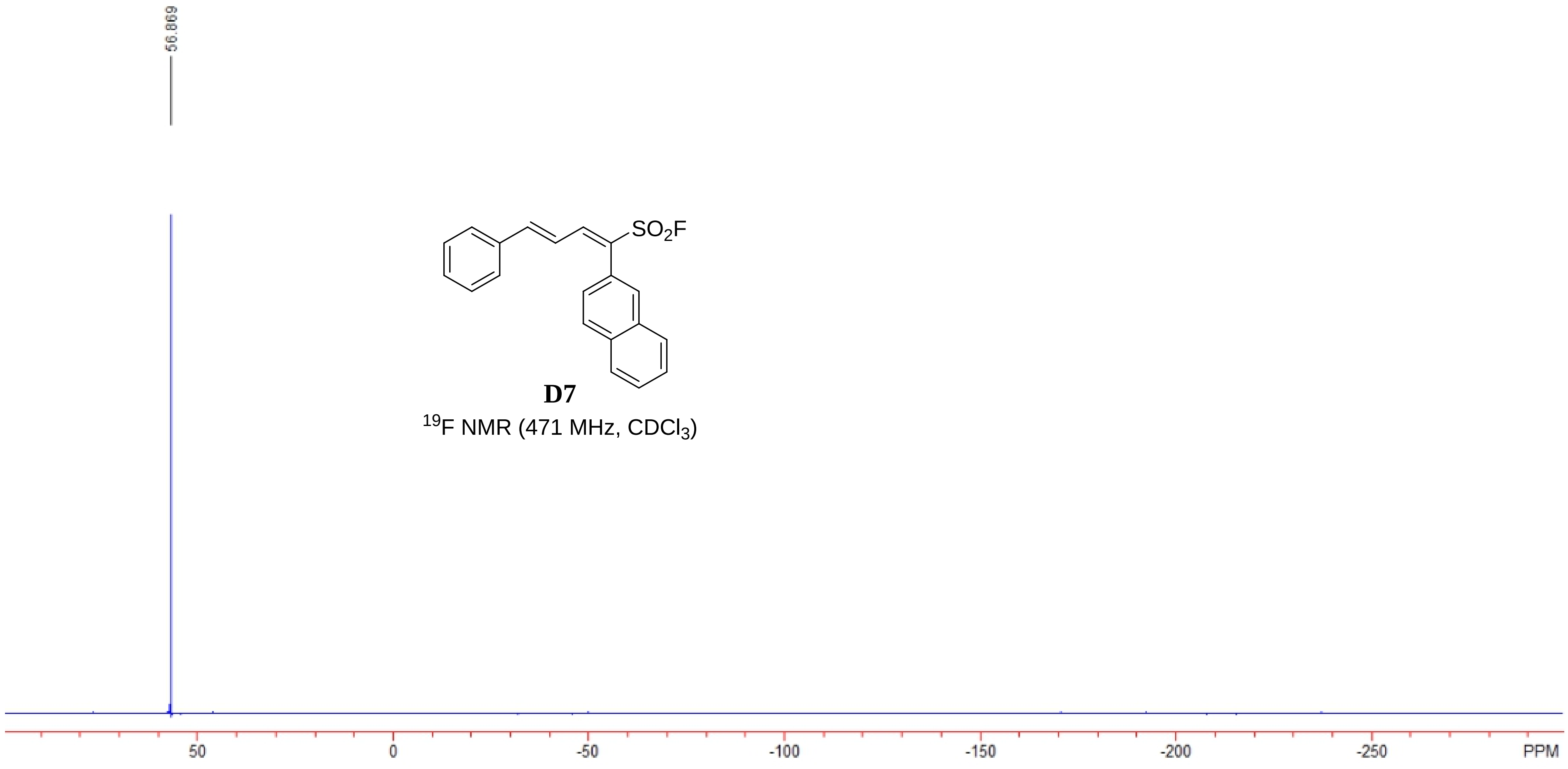

D7

^{19}F NMR (471 MHz, $CDCl_3$)

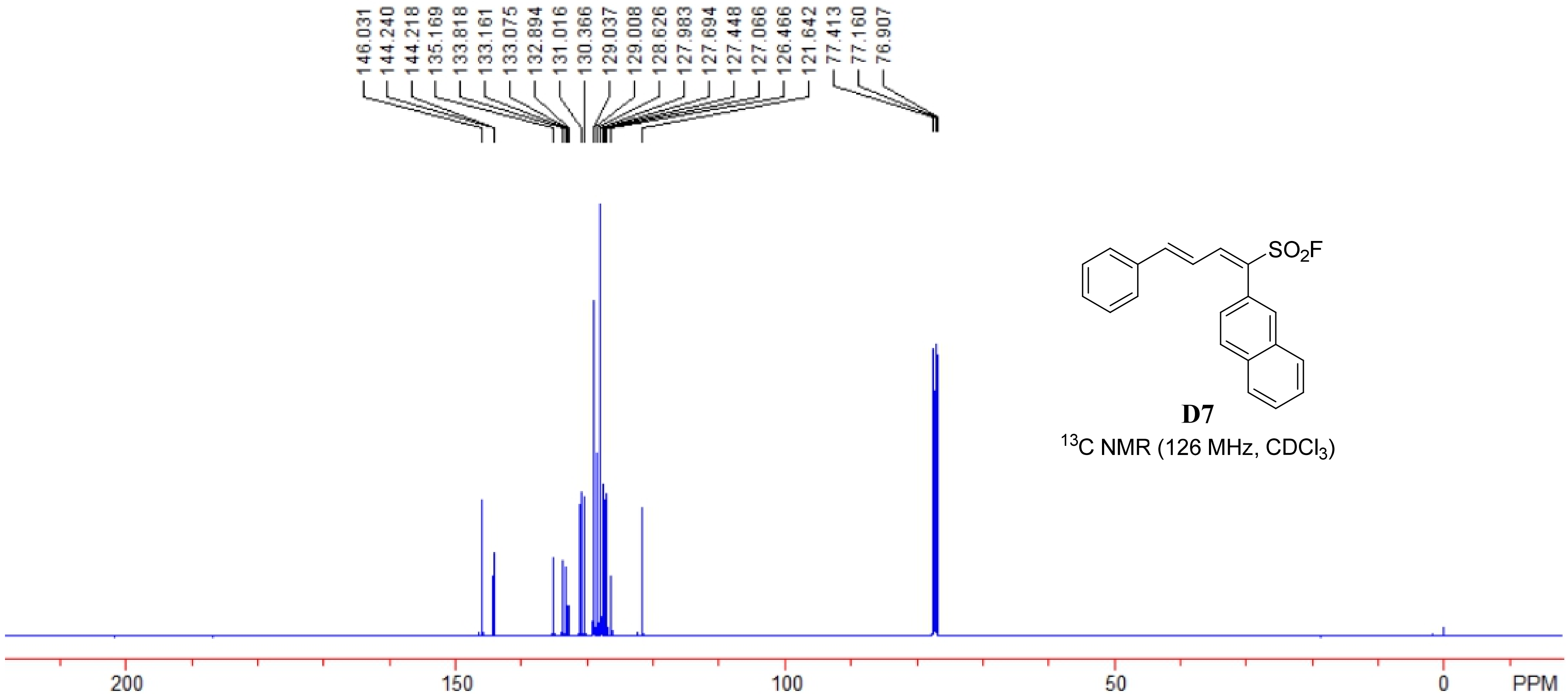

D7

^{13}C NMR (126 MHz, $CDCl_3$)

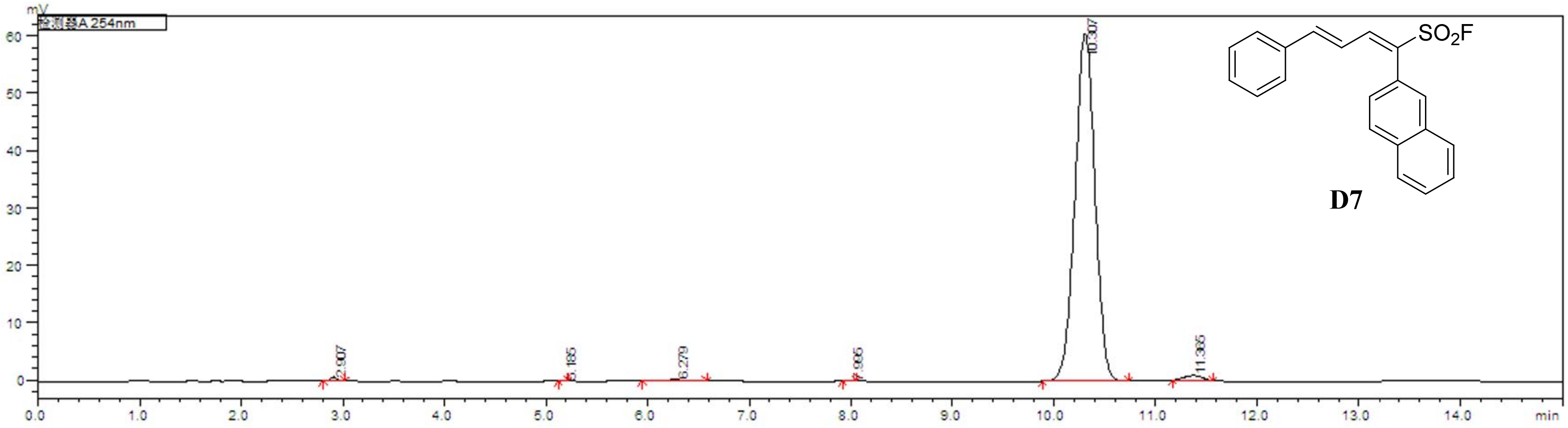

No.	Ret Time (min)	Area (mAU*min)	Rel.Area (%)
1	2.907	3554	0.42%
2	5.185	119	0.01%
3	6.279	4652	0.55%
4	7.995	218	0.03%
5	10.307	827978	97.68%
6	11.365	11090	1.31%
Total		847611	

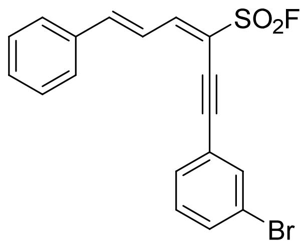

D8

^1H NMR (500 MHz, $CDCl_3$)

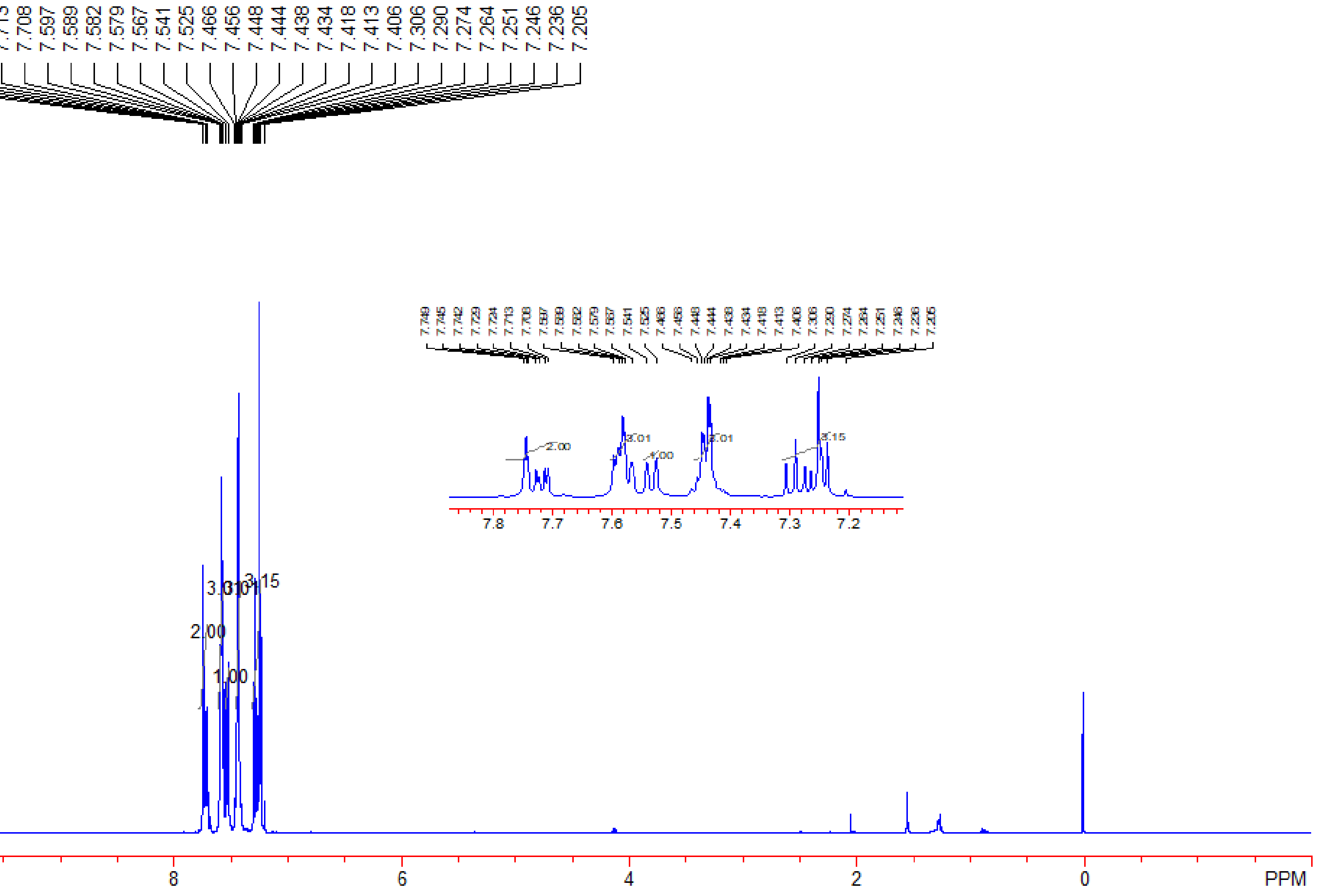

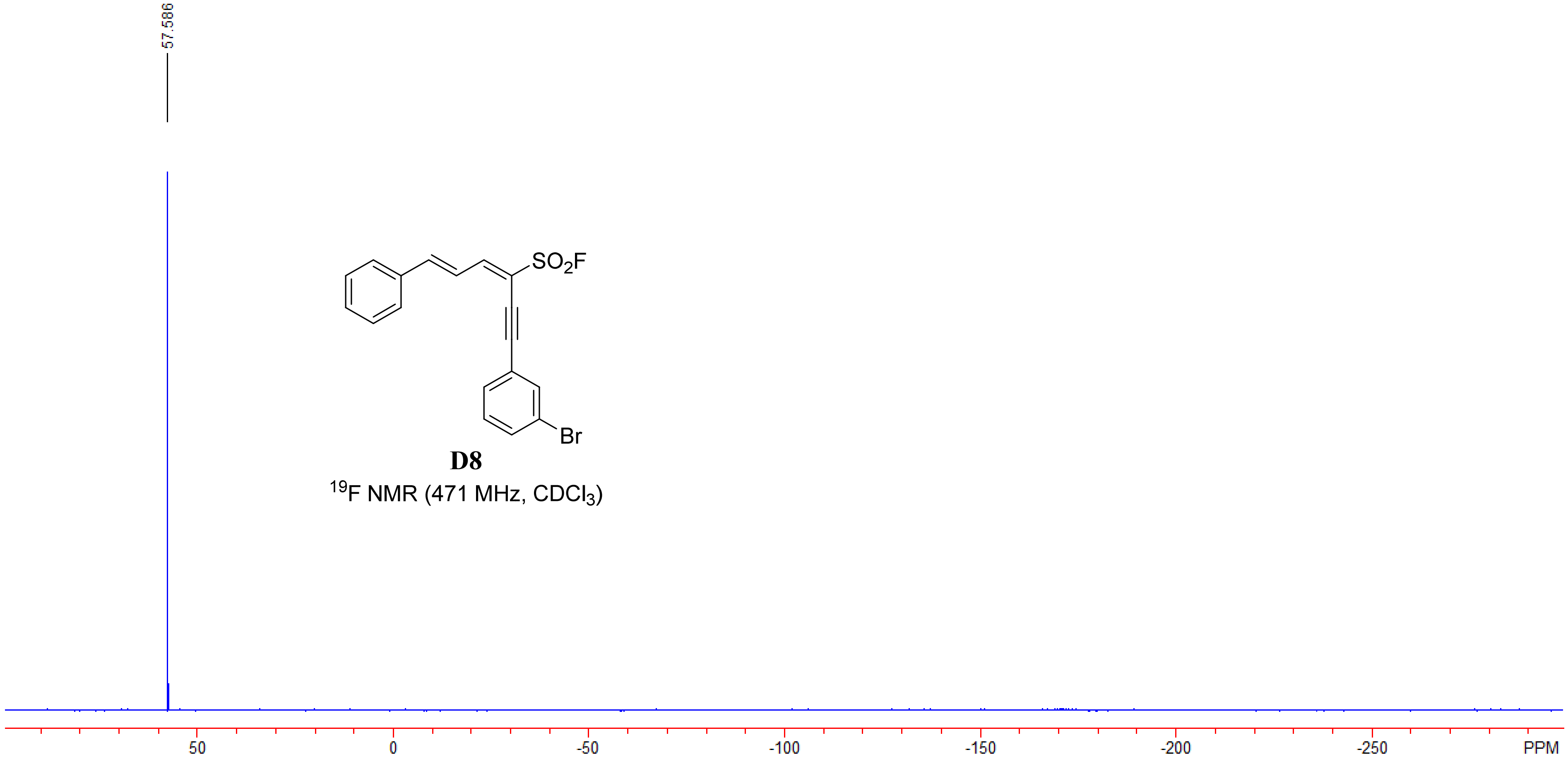

D8

^{19}F NMR (471 MHz, $CDCl_3$)

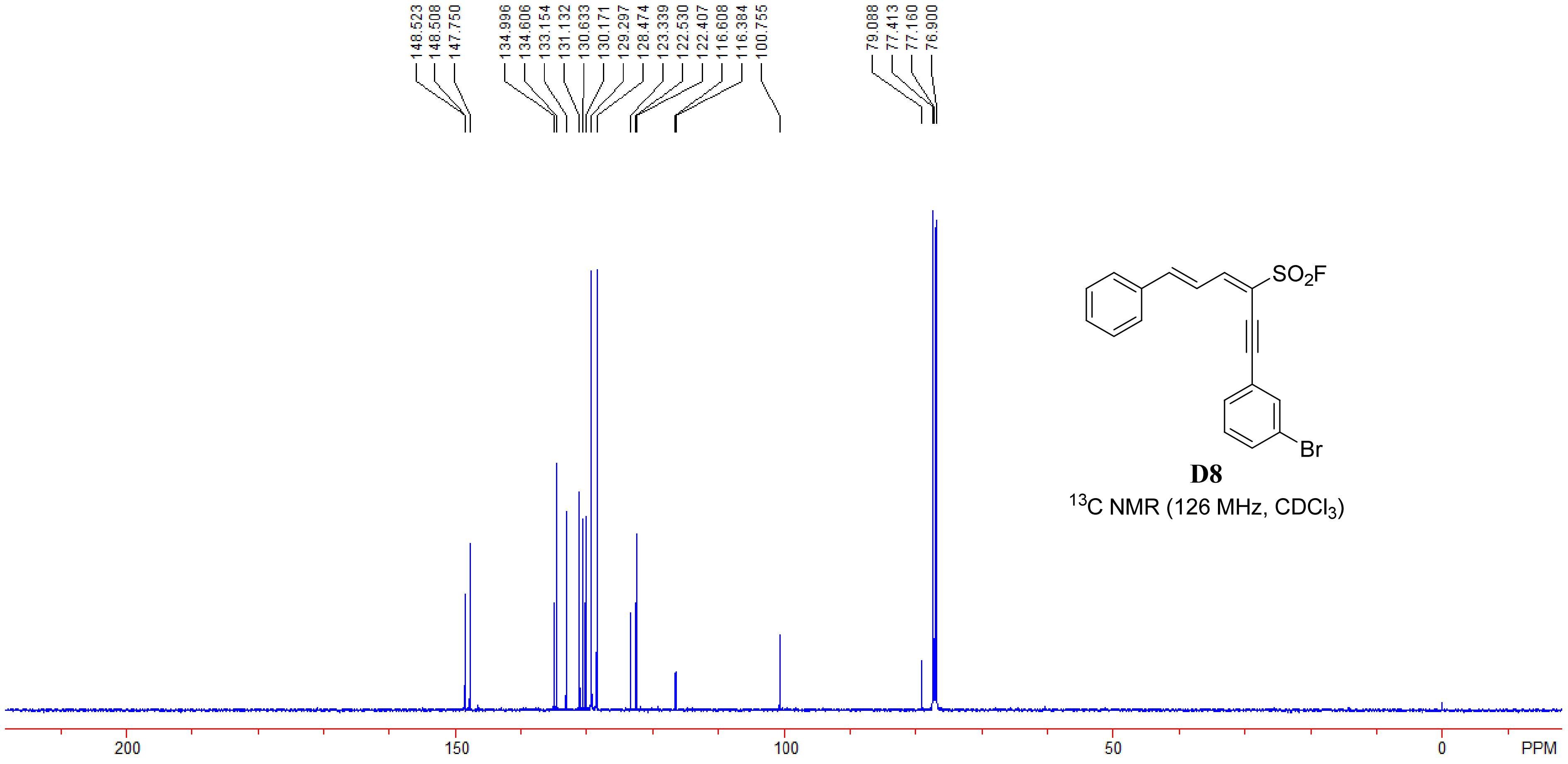
148.523
148.508
147.750
134.996
134.606
133.154
131.132
130.633
130.171
129.297
128.474
123.339
122.530
122.407
116.608
116.384
100.755
79.088
77.413
77.160
76.900
SO2F
Br
D8
13C NMR (126 MHz, CDCl3)
200
150
100
50
0
PPM

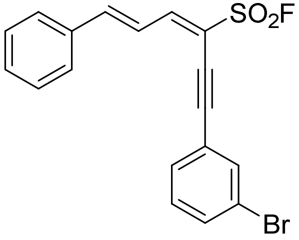
SO2F
Br

D8

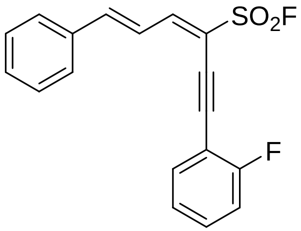

D9

^{1}H NMR (500 MHz, $CDCl_3$)

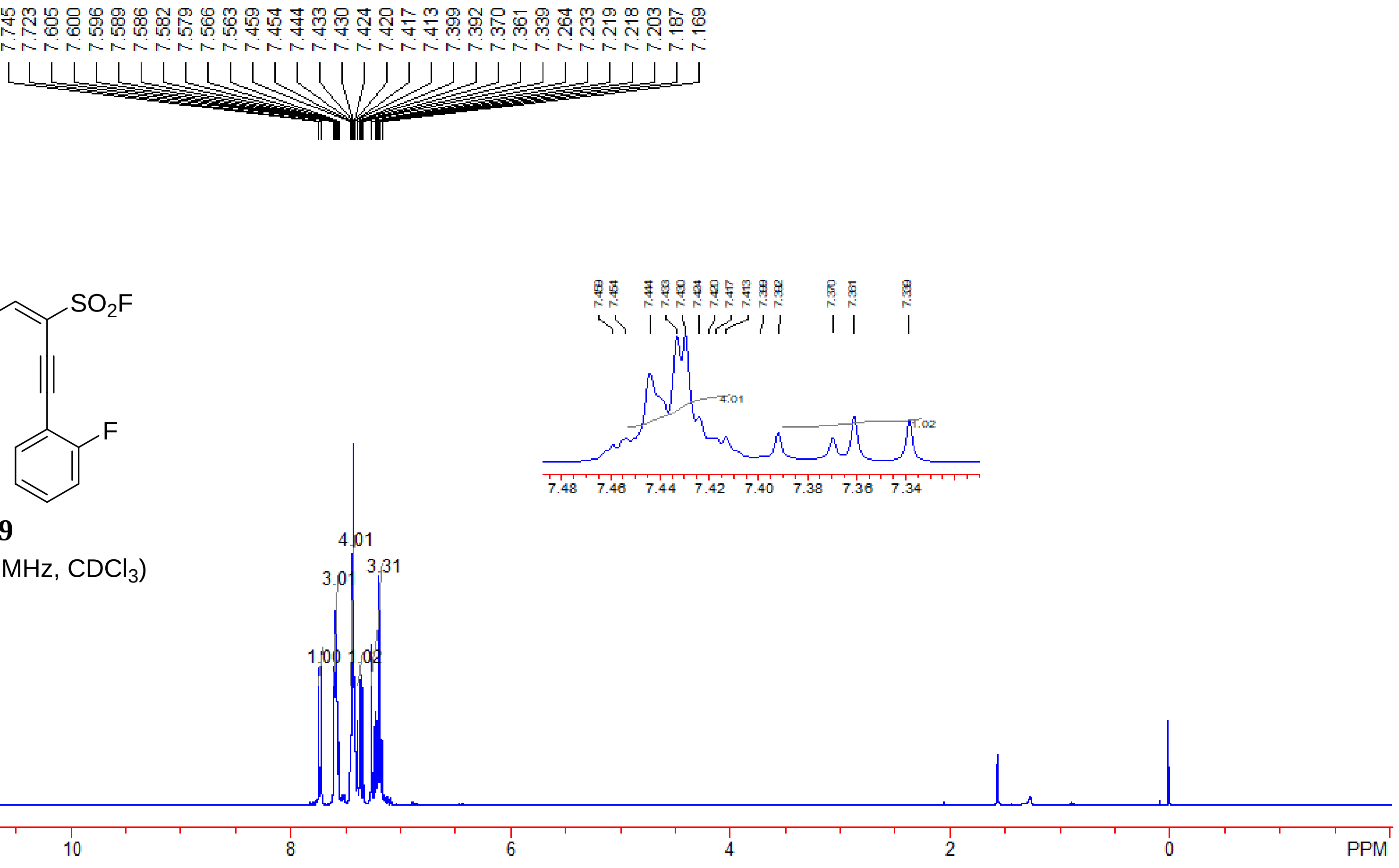

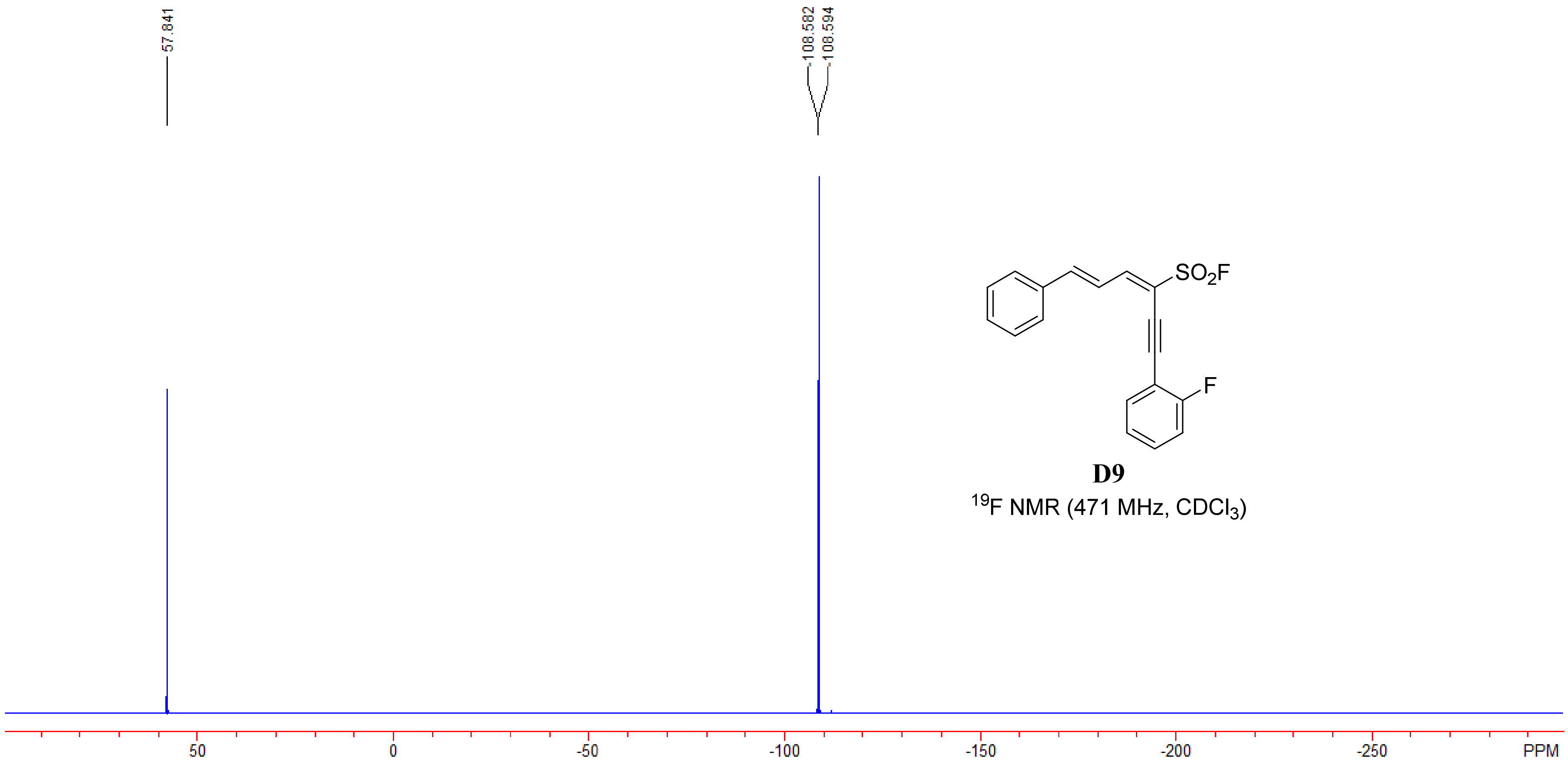

D9

^{19}F NMR (471 MHz, $CDCl_3$)

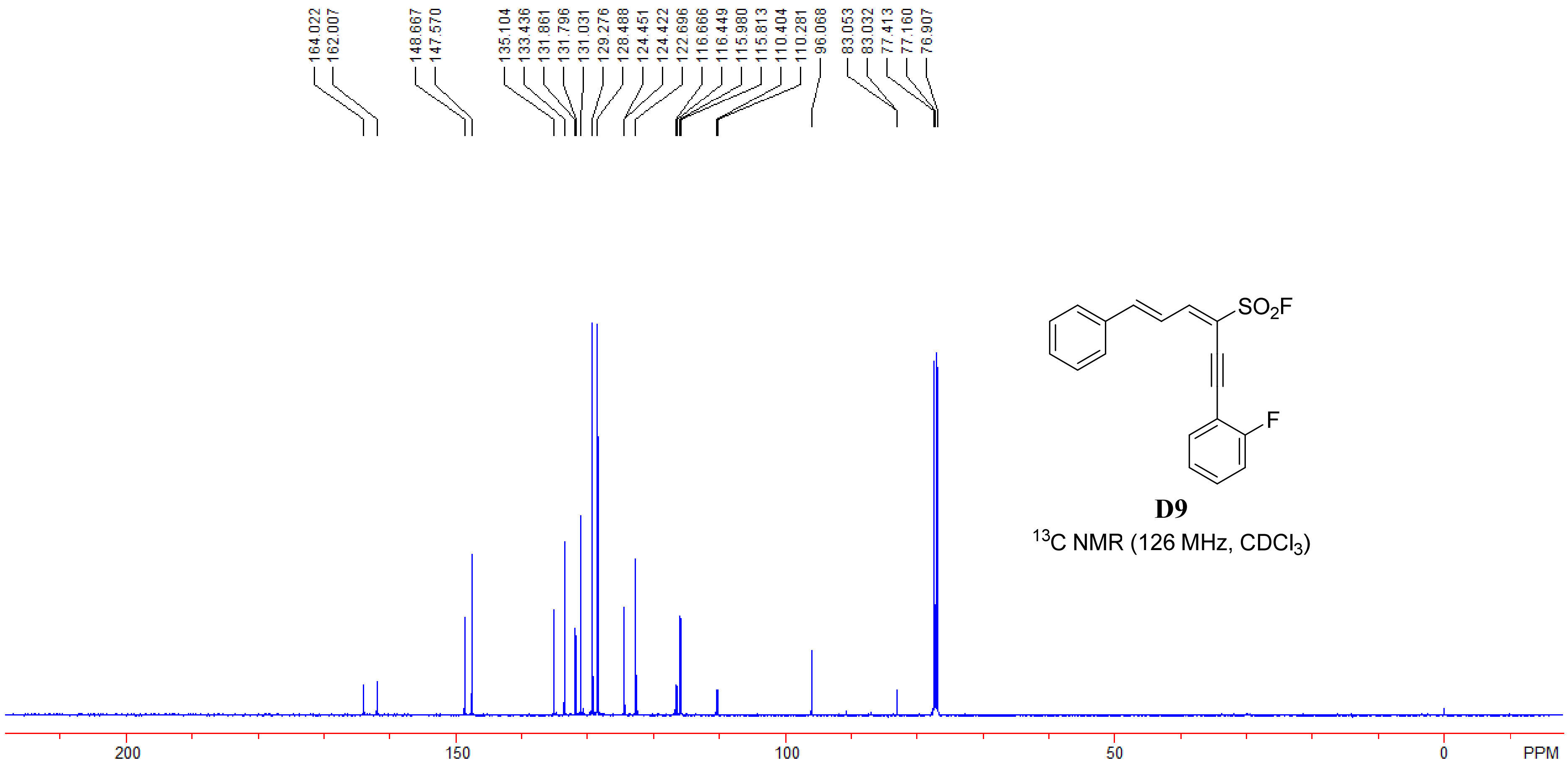

D9

^{13}C NMR (126 MHz, $CDCl_3$)

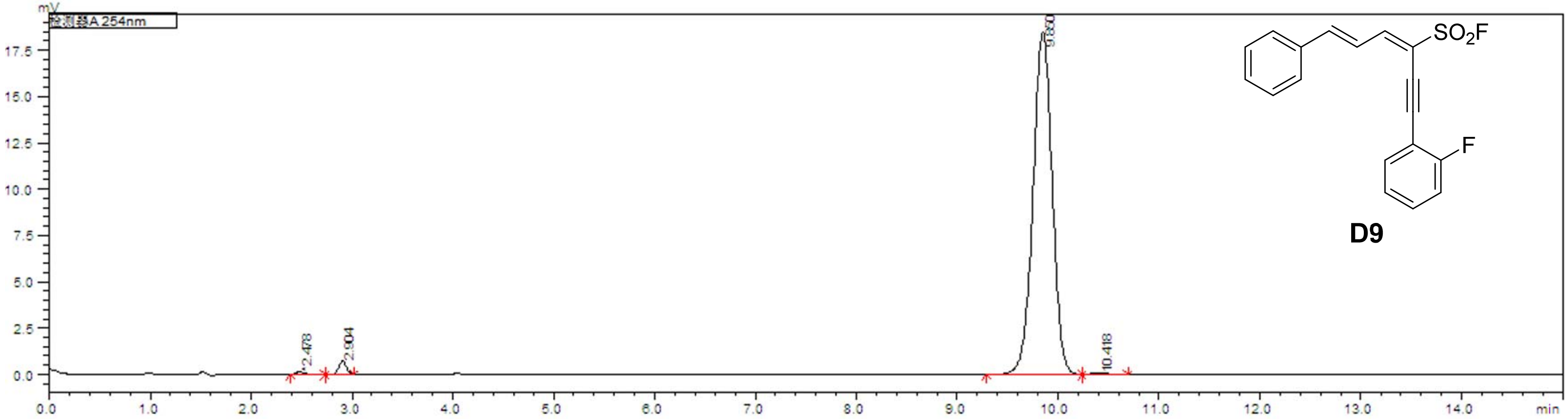

No.	Ret Time (min)	Area (mAU*min)	Rel.Area (%)
1	2.478	1086	0.43%
2	2.904	4149	1.64%
3	9.850	247002	97.42%
4	10.418	1309	0.52%
Total		253545	

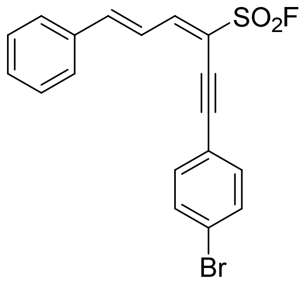

D10

^{1}H NMR (500 MHz, $CDCl_3$)

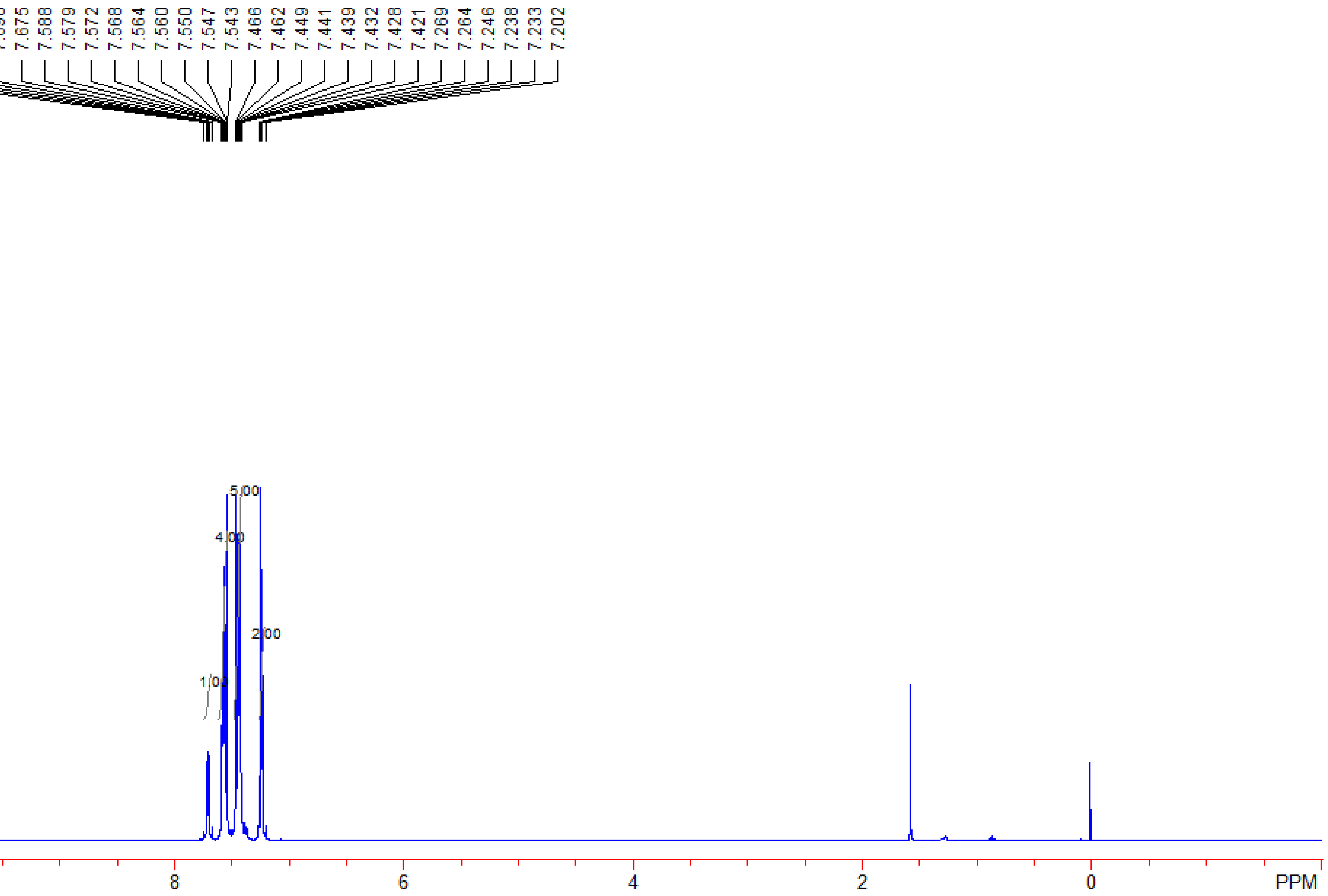

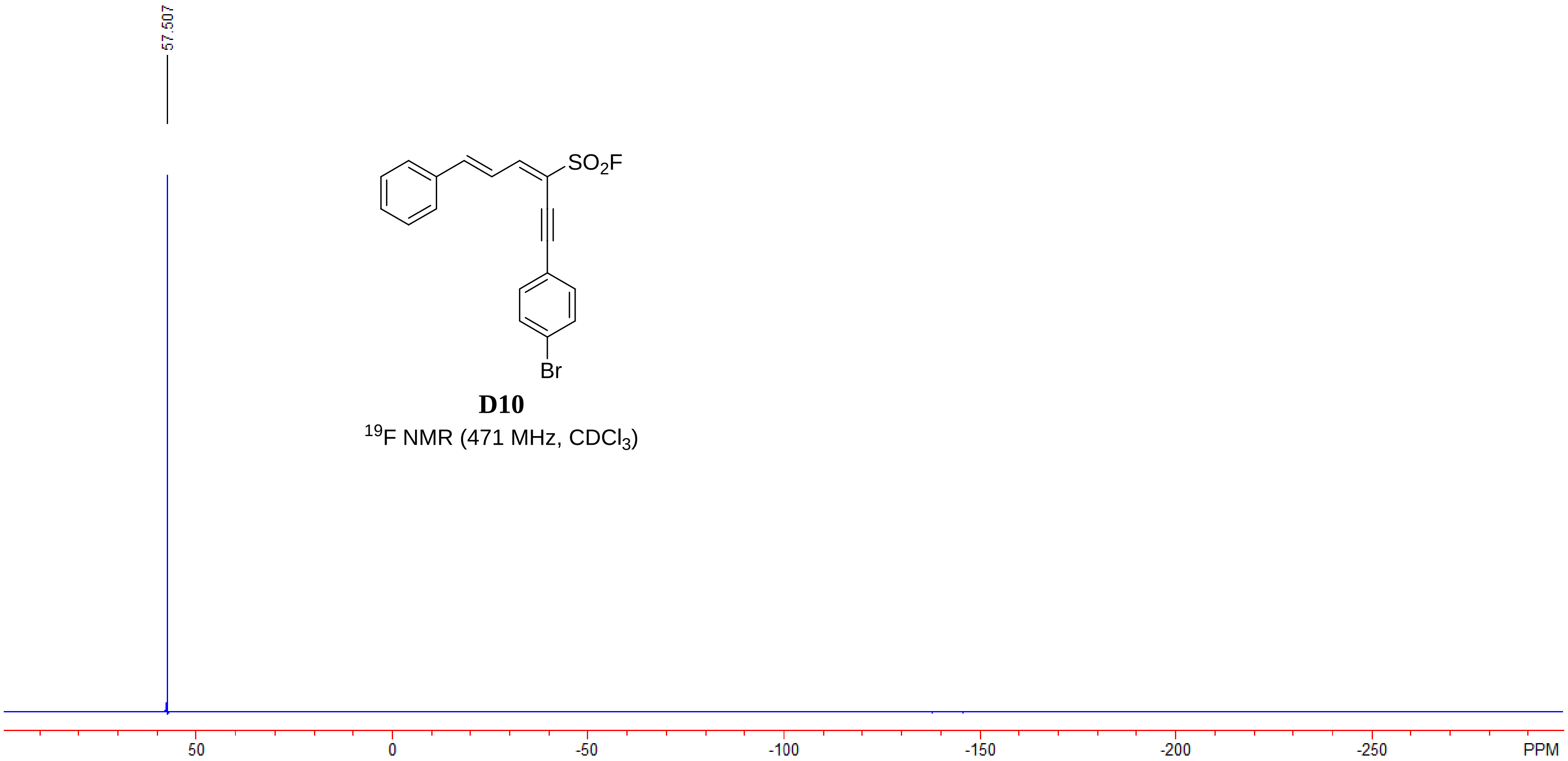
SO2F
Br
D10
19F NMR (471 MHz, CDCl3)
57.507
50
0
-50
-100
-150
-200
-250
PPM

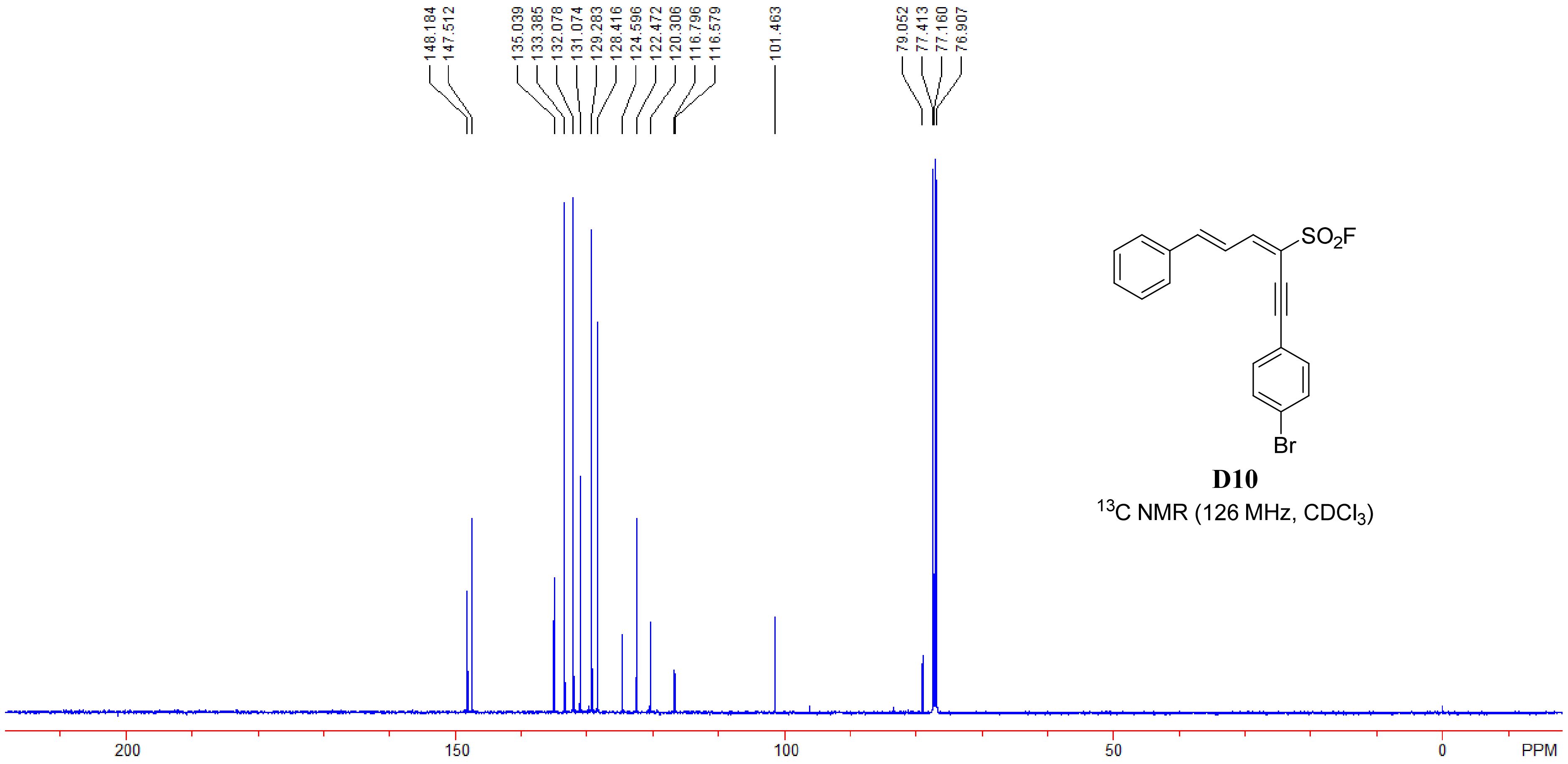

D10

^{13}C NMR (126 MHz, $CDCl_3$)

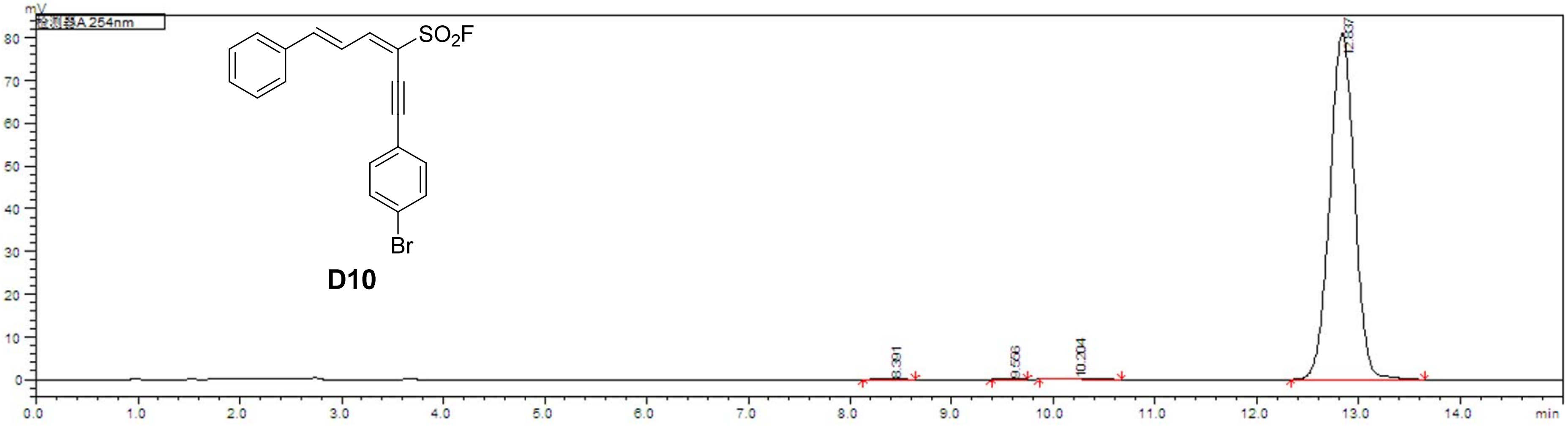

No.	Ret Time (min)	Area (mAU*min)	Rel.Area (%)
1	8.391	3482	0.25%
2	9.556	2683	0.20%
3	10.204	4070	0.30%
4	12.837	1360855	99.25%
Total		1371089	

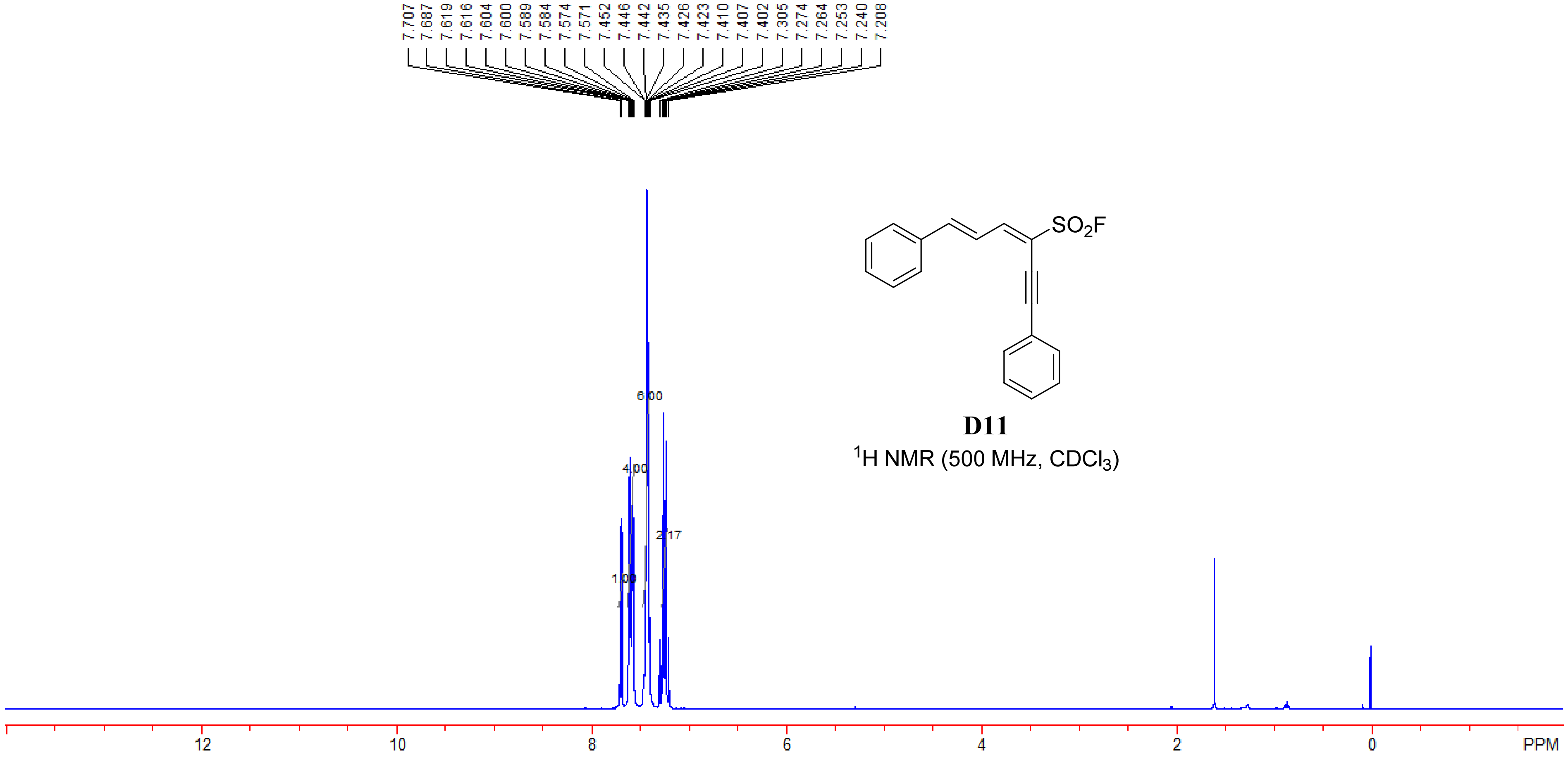

D11

^{1}H NMR (500 MHz, $CDCl_3$)

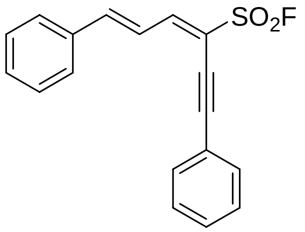

D11

^{19}F NMR (471 MHz, $CDCl_3$)

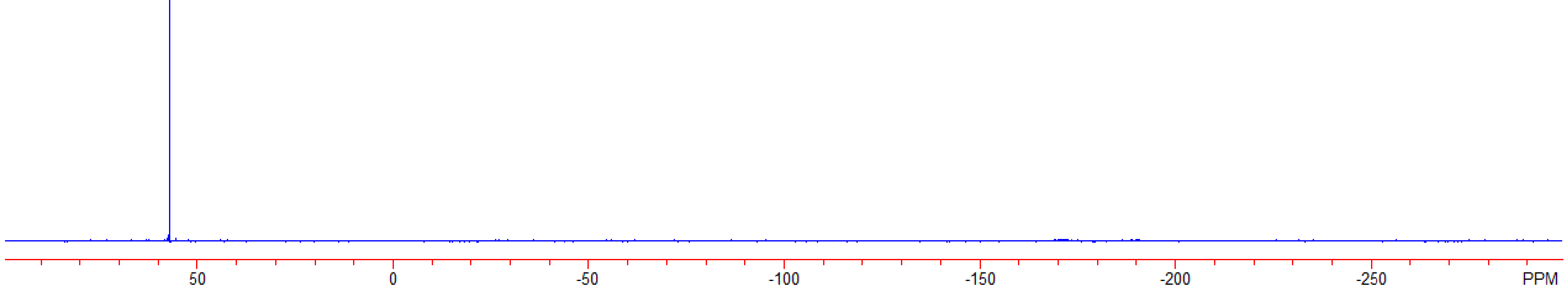

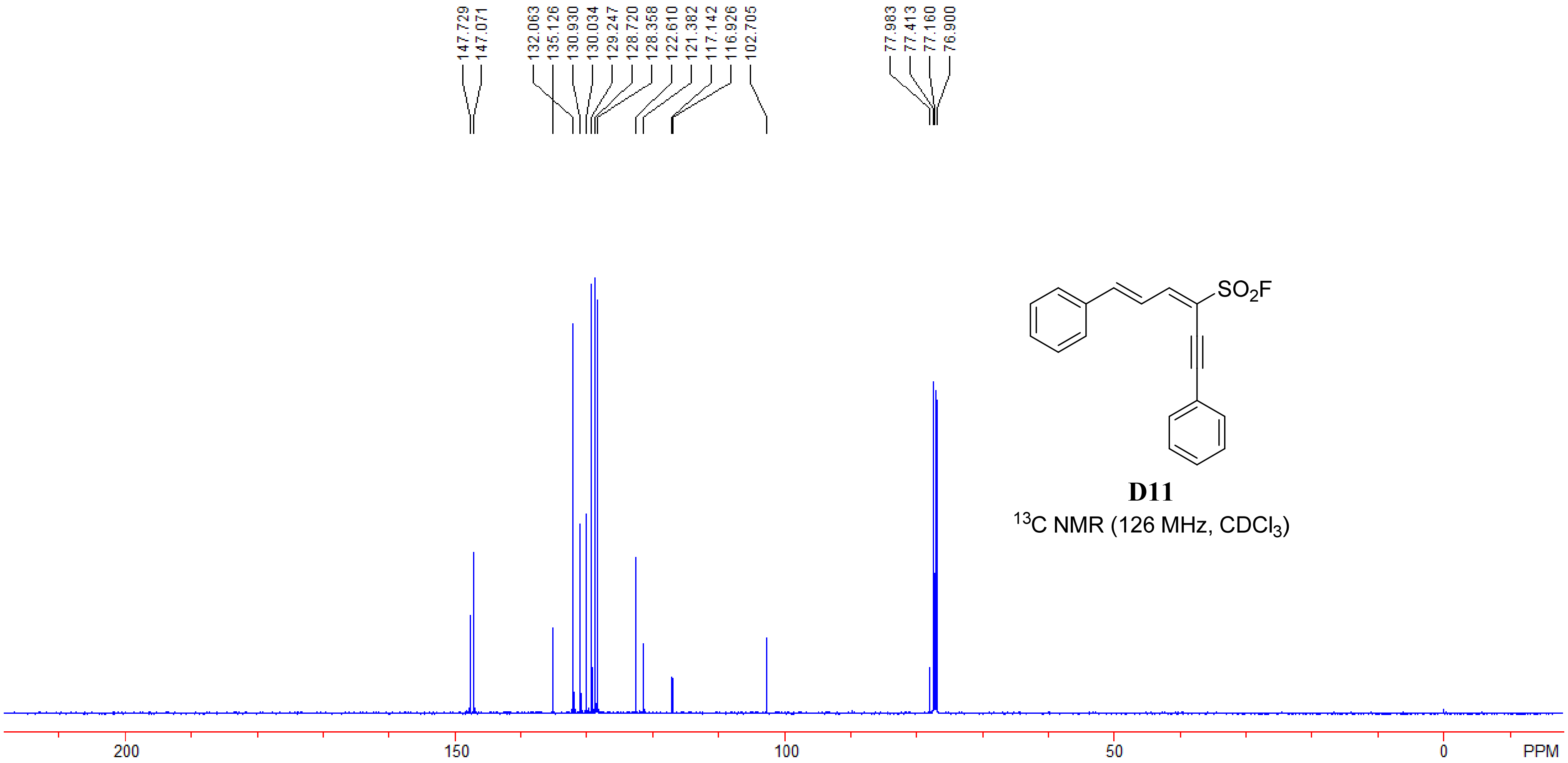

D11

^{13}C NMR (126 MHz, $CDCl_3$)

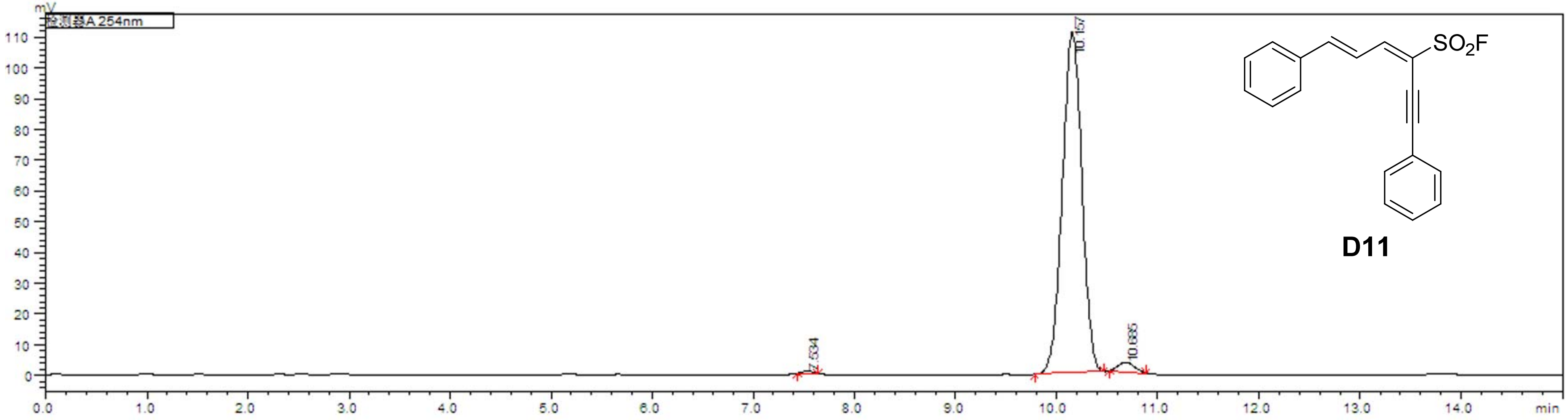

No.	Ret Time (min)	Area (mAU*min)	Rel.Area (%)
1	7.543	5413	0.35%
2	10.157	1484580	97.34%
3	10.685	35201	2.31%
Total		1525194	

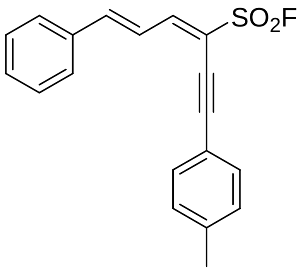

D12

^{1}H NMR (500 MHz, $CDCl_3$)

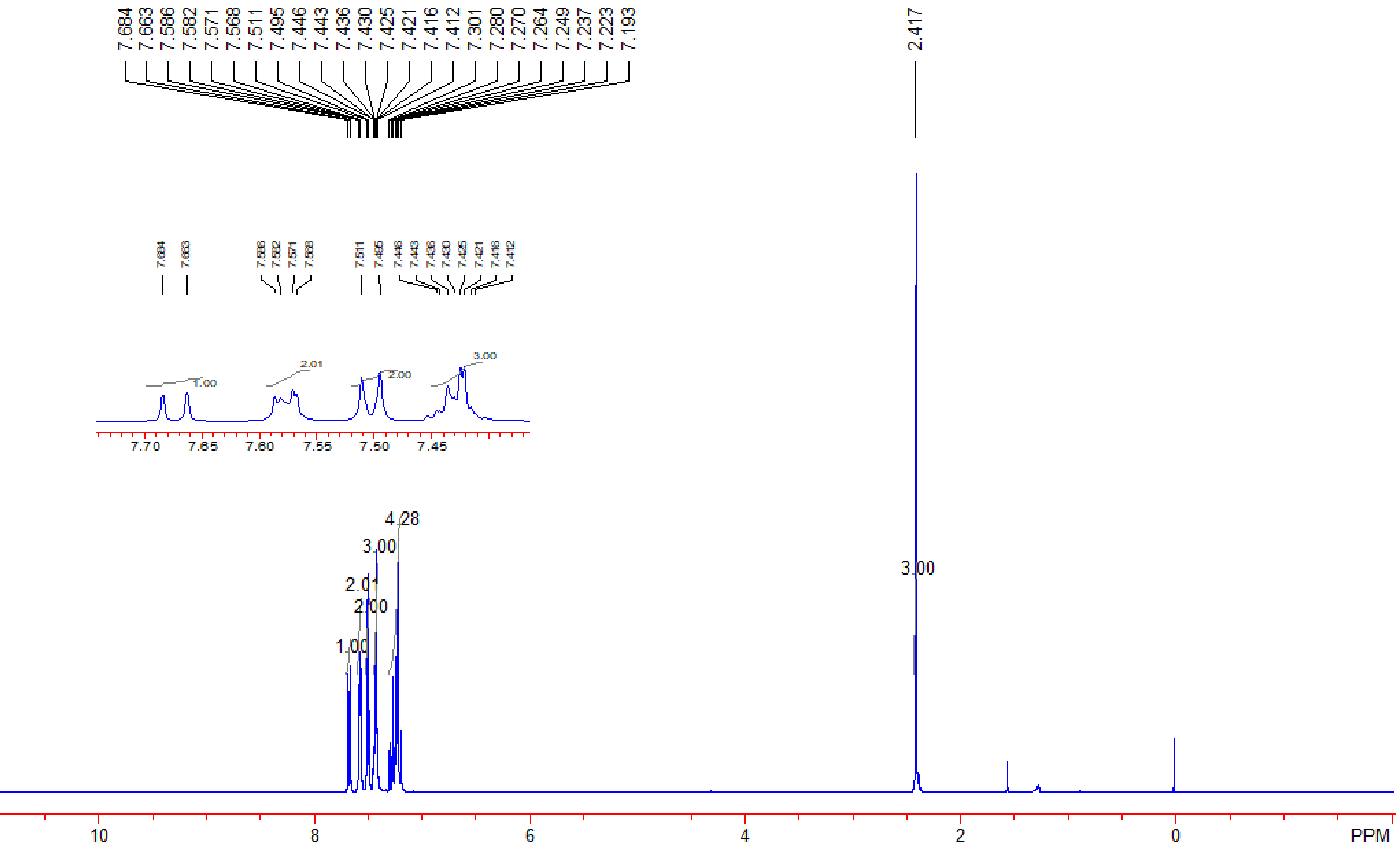

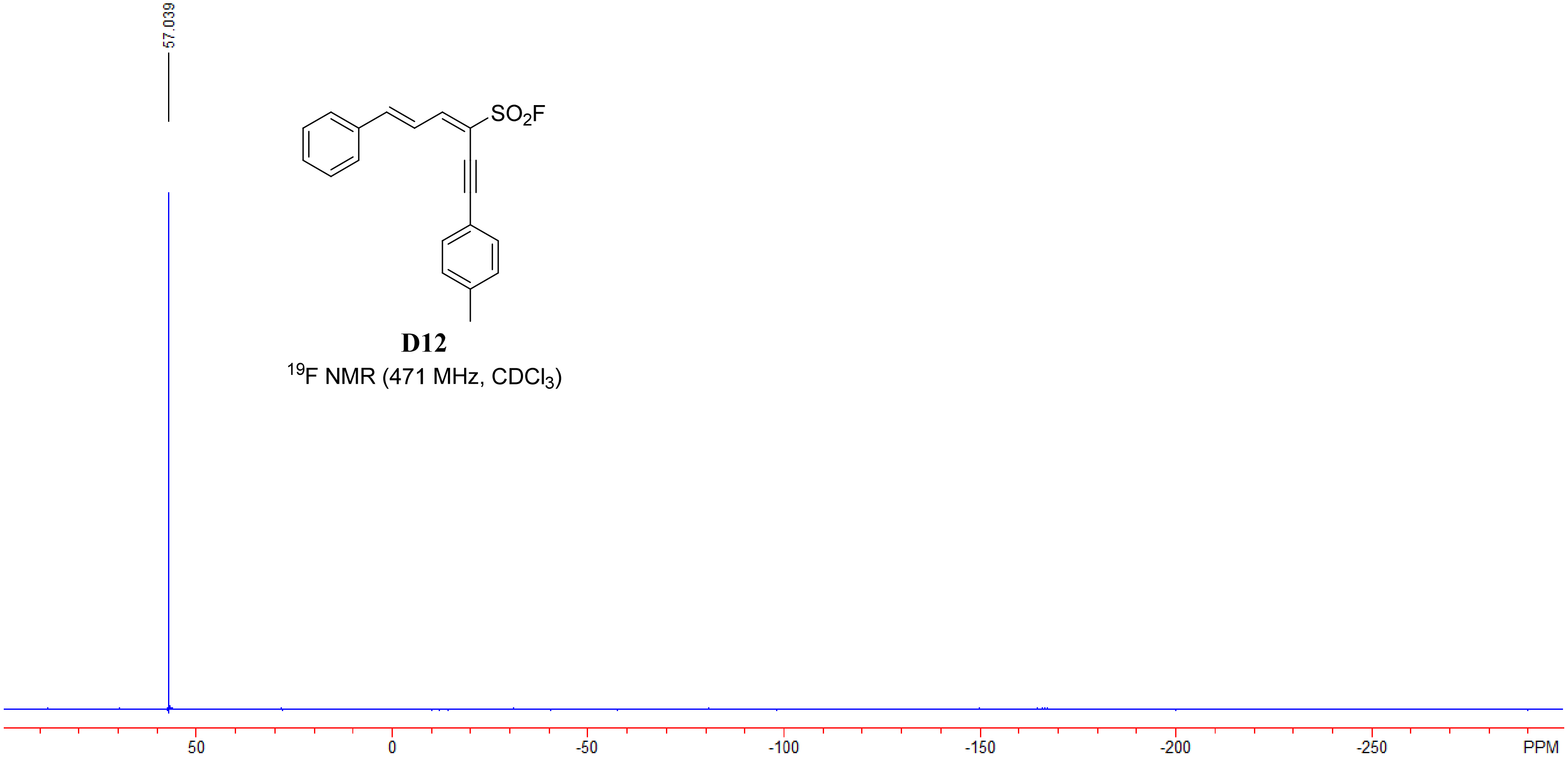

D12

^{19}F NMR (471 MHz, $CDCl_3$)

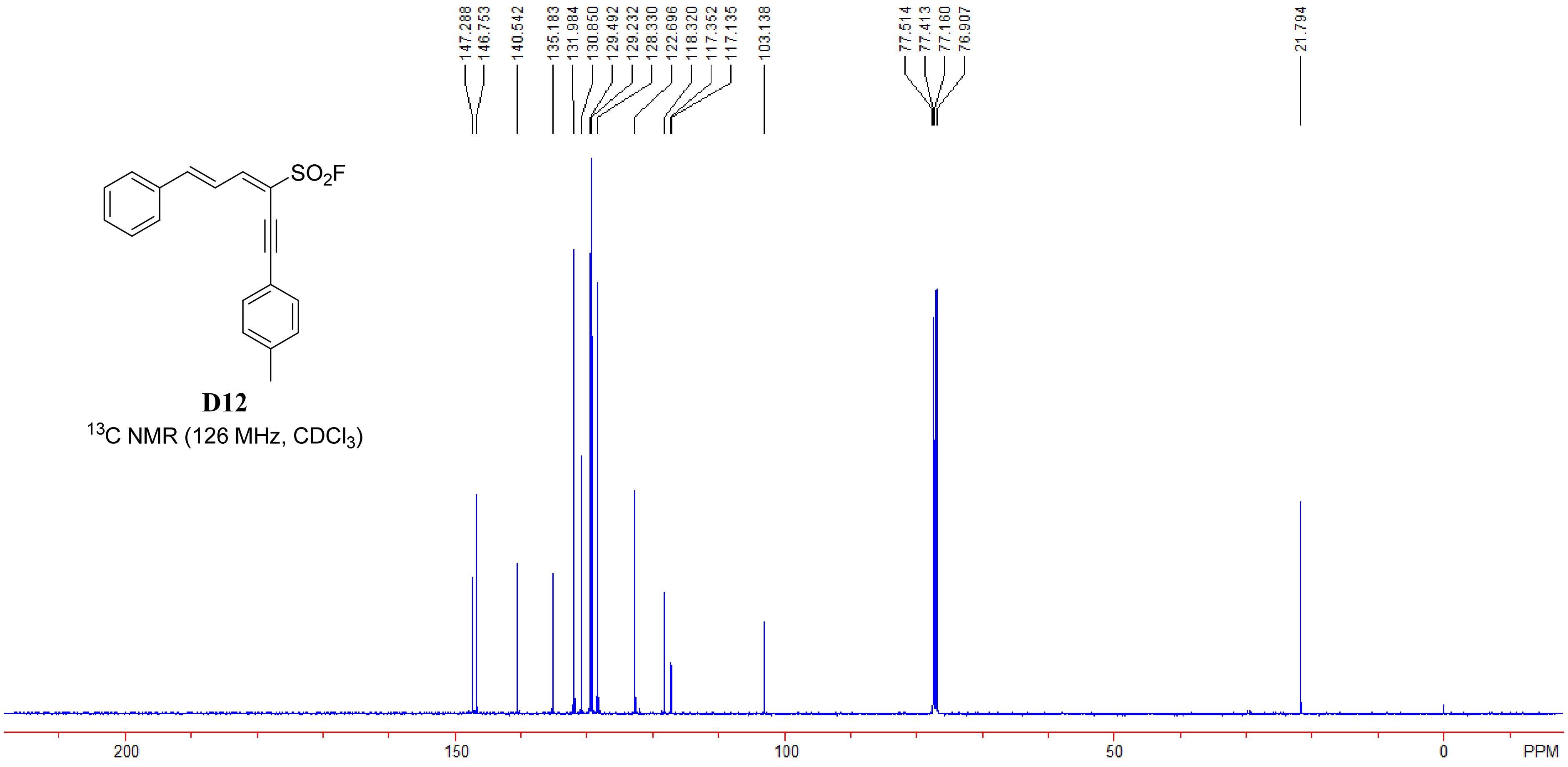

D12

^{13}C NMR (126 MHz, $CDCl_3$)

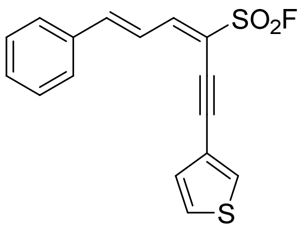

D13

^{1}H NMR (500 MHz, $CDCl_3$)

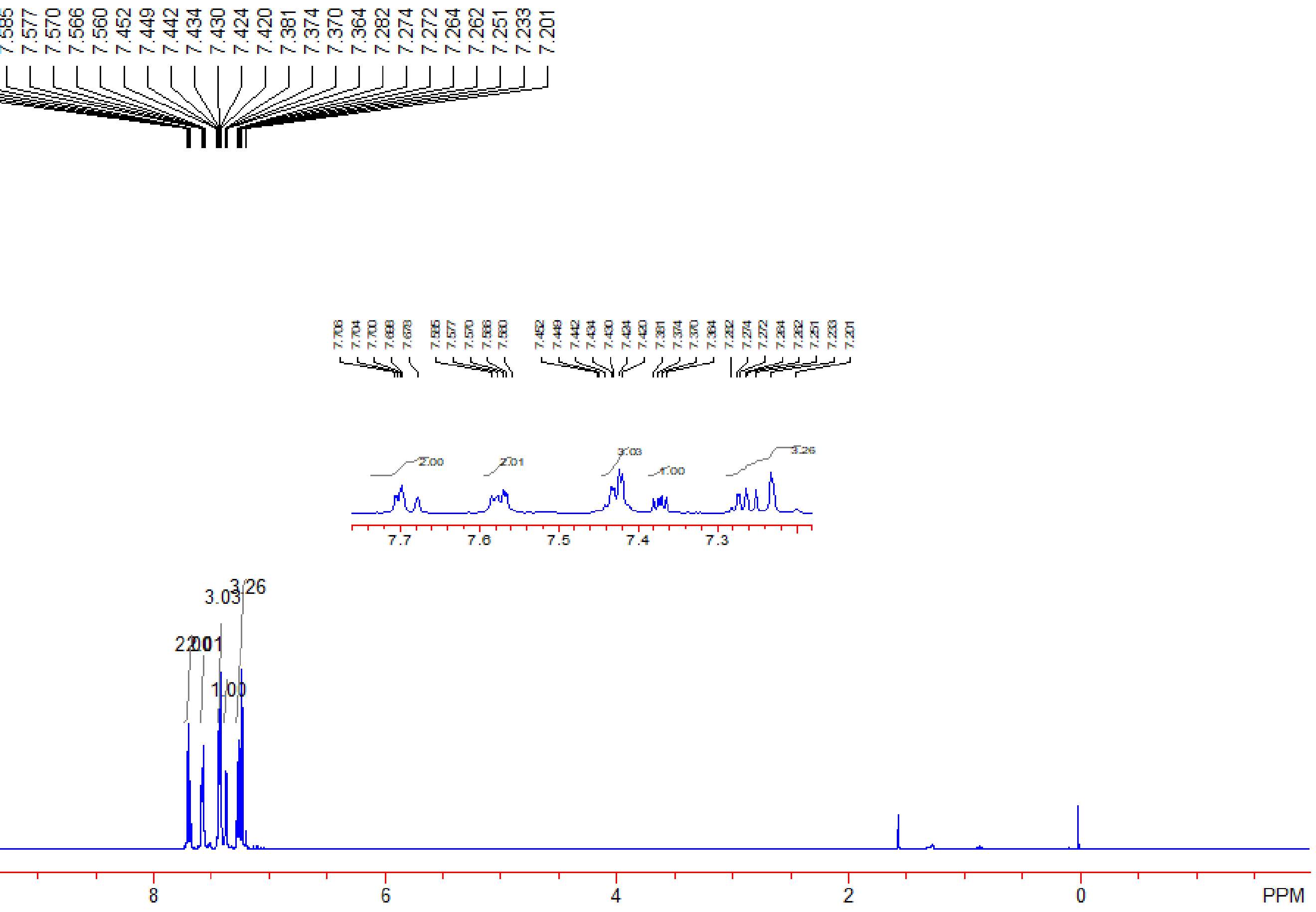

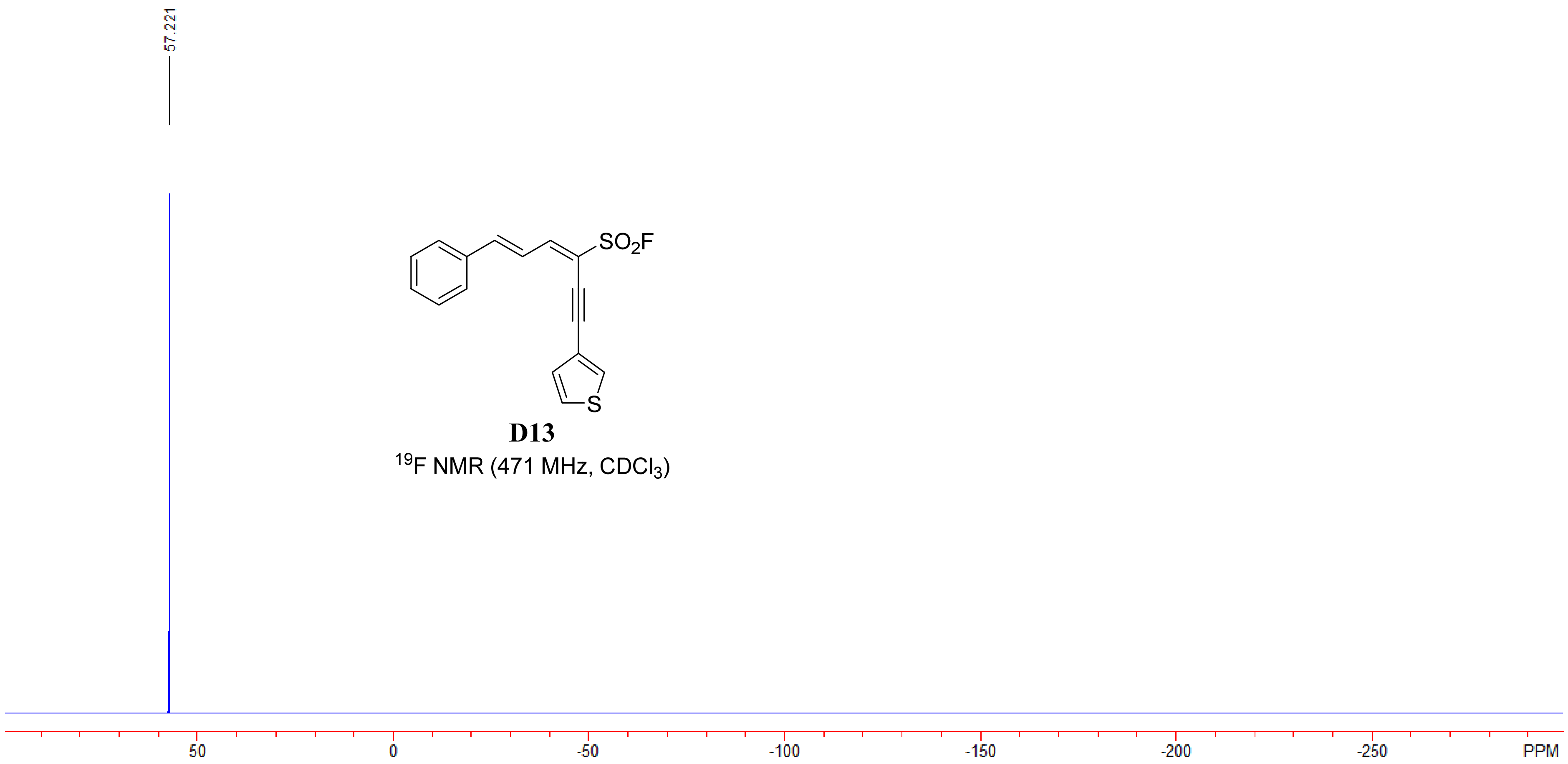

D13

^{19}F NMR (471 MHz, $CDCl_3$)

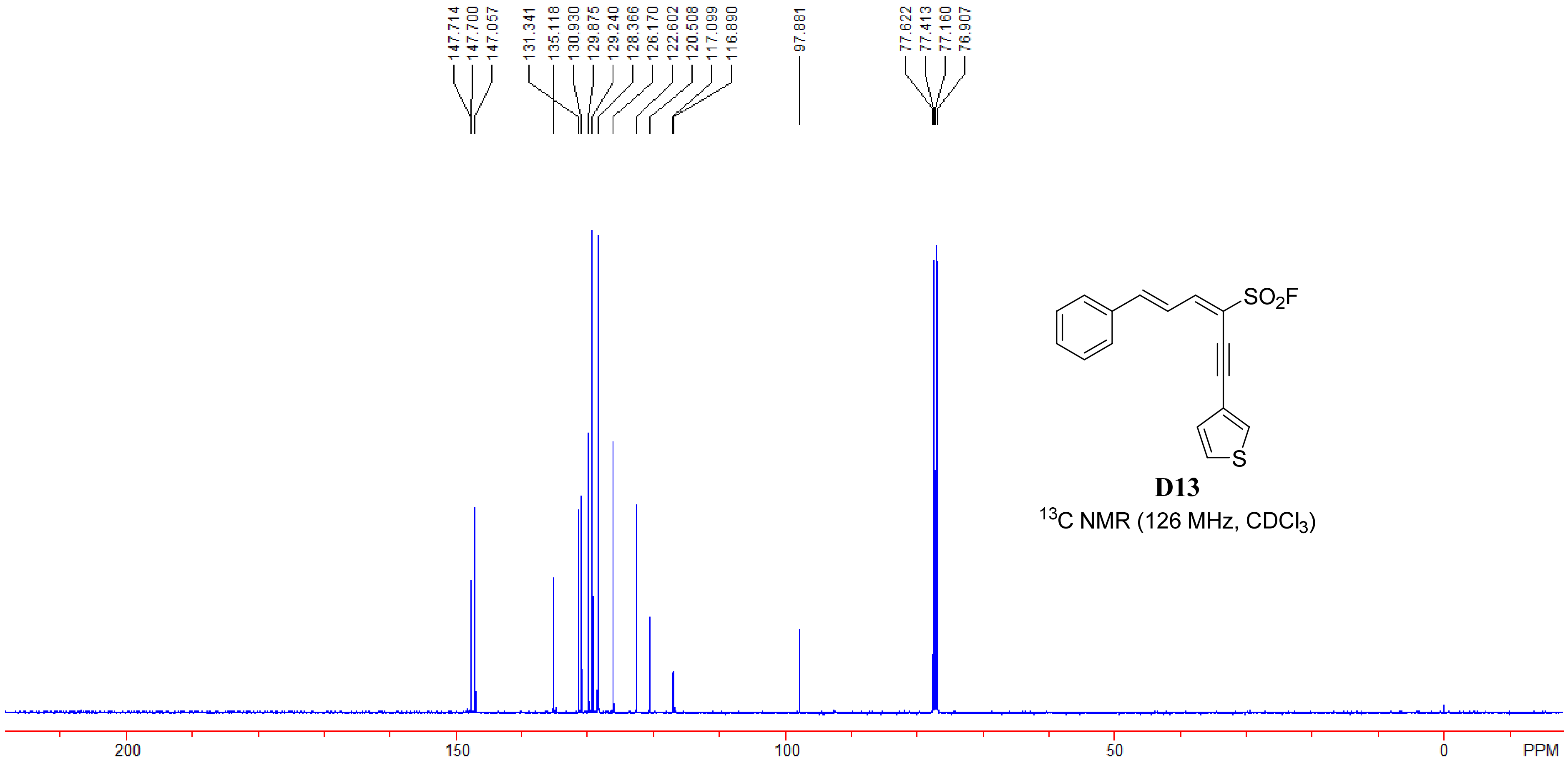
147.714
147.700
147.057
131.341
135.118
130.930
129.875
129.240
128.366
126.170
122.602
120.508
117.099
116.890
97.881
77.622
77.413
77.160
76.907
SO2F
S
D13
13C NMR (126 MHz, CDCl3)
200
150
100
50
0
PPM

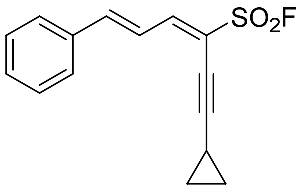

D14

^{1}H NMR (500 MHz, $CDCl_3$)

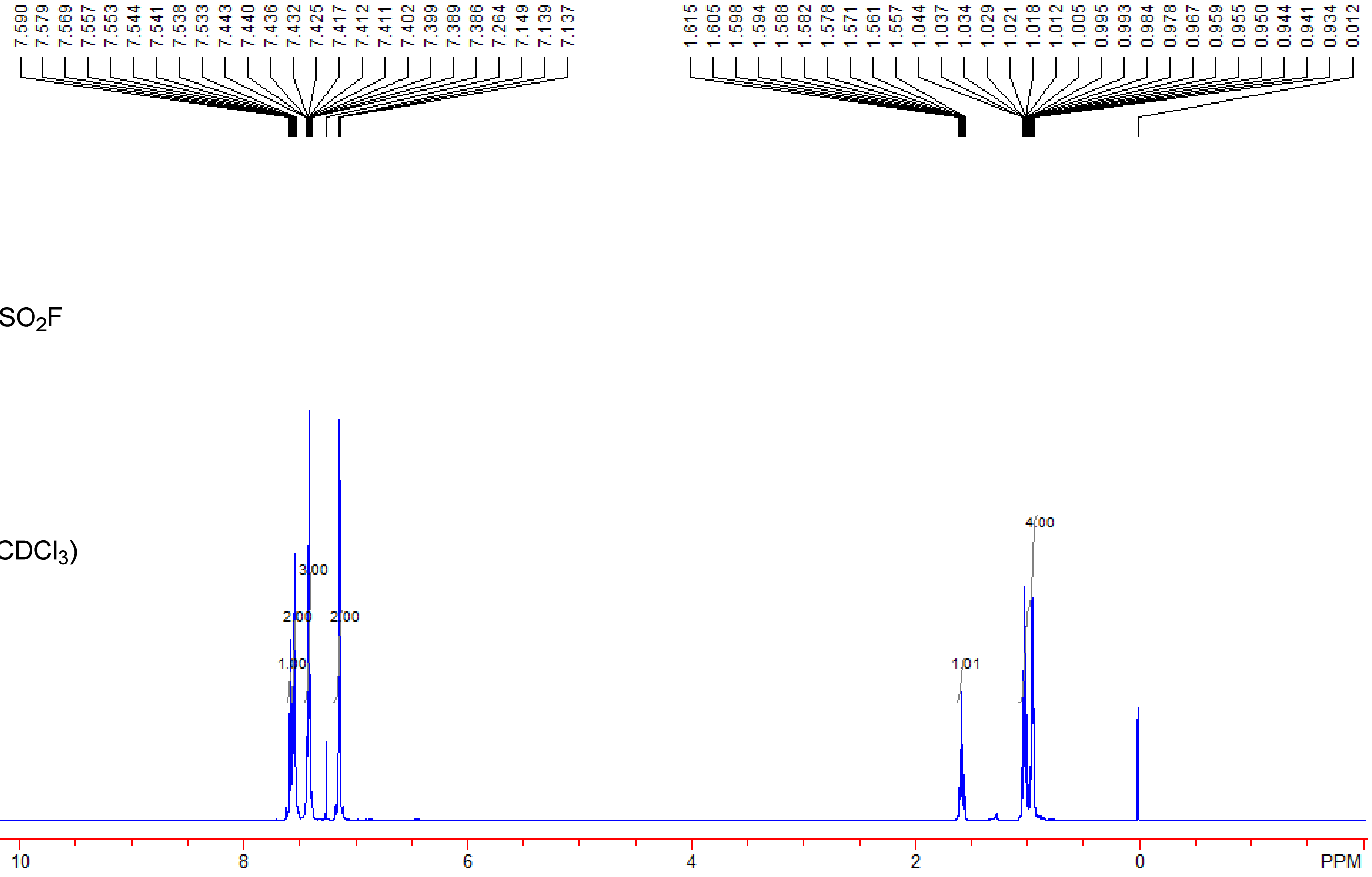

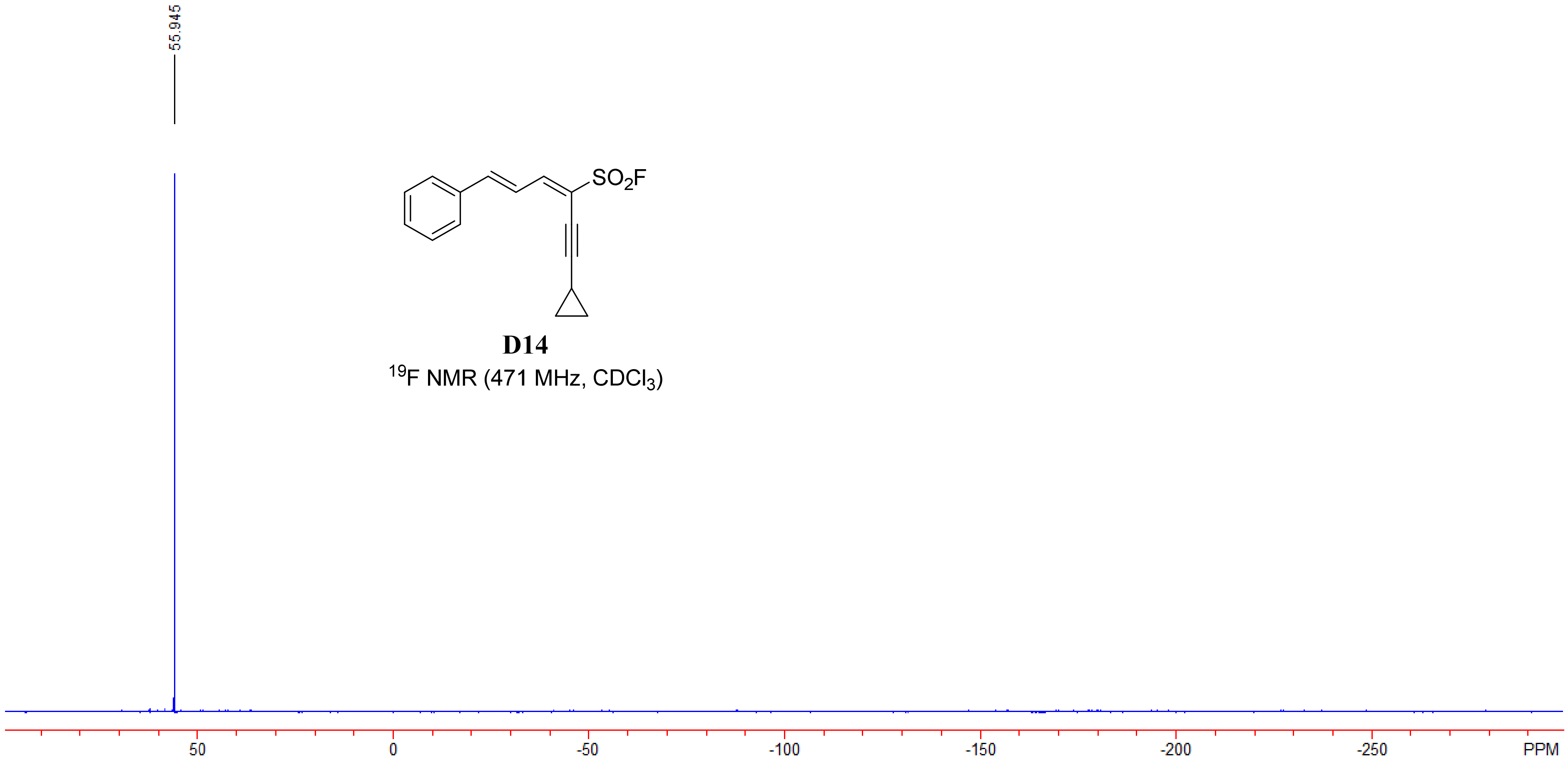

D14

^{19}F NMR (471 MHz, $CDCl_3$)

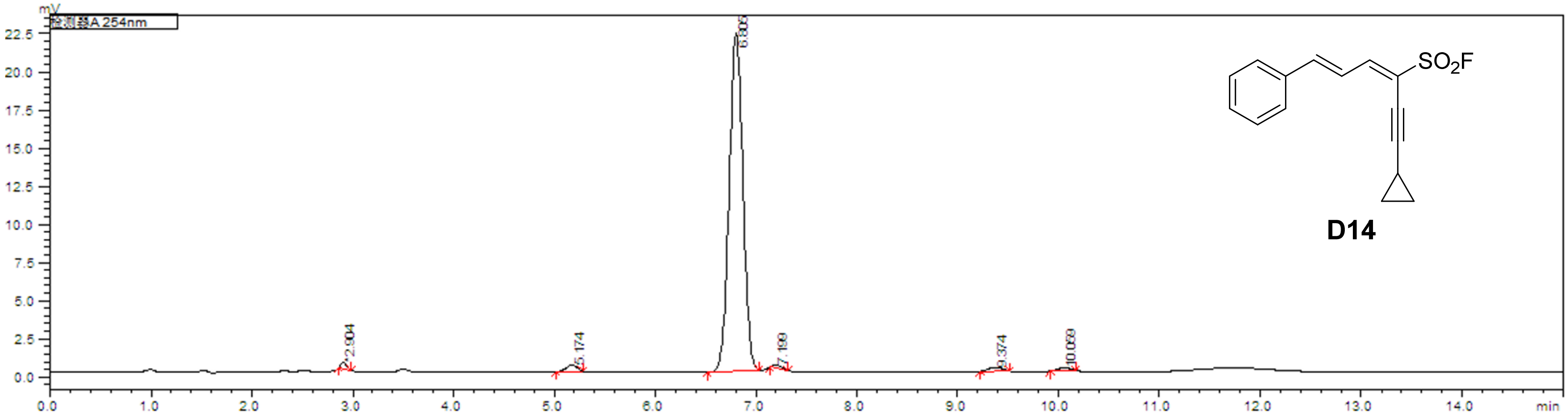

No.	Ret Time (min)	Area (mAU*min)	Rel.Area (%)
1	2.904	1664	0.76%
2	5.174	3076	1.40%
3	6.805	209561	95.50%
4	7.199	1234	0.56%
5	9.374	2419	1.10%
6	10.059	1473	0.67%
Total		219427	
